# Nutrigenomic influence of a curcumin-supplemented high glycemic diet on hippocampal microvasculature in male C57BL/6J mice

**DOI:** 10.3389/fnut.2025.1736964

**Published:** 2026-02-03

**Authors:** Emilio Balbuena, Jennifer M. Rutkowsky, Saivageethi Nuthikattu, Amparo C. Villablanca, Dragan Milenkovic

**Affiliations:** 1 Plants for Human Health Institute, North Carolina State University, Kannapolis, NC, United States; 2 Department of Molecular Biosciences, School of Veterinary Medicine, University of California, Davis, Davis, CA, United States; 3 Division of Cardiovascular Medicine, Department of Internal Medicine, University of California, Davis, Sacramento, CA, United States

**Keywords:** curcumin, high glycemic diet, hippocampus, microarray, nutrigenomics

## Abstract

**Introduction:**

Curcumin, a dietary polyphenol primarily derived from turmeric, has potent antioxidant and anti-inflammatory capabilities against diet-related chronic diseases. A high glycemic diet (HGD) has been shown to contribute to cognitive decline and dysfunction of murine brain microvasculature. The goal of our study was to elucidate the multi-genomic effects of curcumin on hippocampal microvessels in mice during consumption of a high glycemic diet.

**Methods:**

Male C57BL/6J mice were fed a low glycemic diet (LGD, 12% sucrose/weight), a high glycemic diet (HGD, 34% sucrose), or a HGD with 0.2% curcumin (HGD + Curc) for 12 weeks. Global transcriptomic profiles, including protein coding and non-coding genes, of laser-captured endothelial microvessels of the hippocampus were analyzed via microarrays. Bioinformatic tools were utilized to uncover networks and functional pathways of differentially expressed genes modulated by curcumin as well as interactivity between transcription factors and major curcumin metabolites via *in silico* docking analysis.

**Results:**

The HGD + Curc treatment influenced the differential expression of 1887 genes compared to HGD alone, which included messenger RNAs, microRNAs, long noncoding RNAs, and small nucleolar RNAs. Of these modulated genes, 307 overlapped and were negatively correlated with the fold change expression of the HGD versus LGD comparison. These protein coding and non-coding gene targets regulated by HGD+Curc were involved in pathways related to neurodegeneration, oxidative phosphorylation, blood-brain barrier permeability, cell signaling, and cellular metabolism.

**Discussion/conclusion:**

The results from this study show that curcumin induces complex nutrigenomic modifications that could elucidate its neuroprotective effect against hippocampal microvascular dysfunction induced by a high glycemic diet.

## 1 Introduction

Disruptions in the cerebral vascular system contribute to cognitive decline and the progression of dementia, which is the 5th leading cause of death in individuals aged 65 and older in the United States (1). The microvascular network, composed of capillary endothelial cells that maintain the blood brain barrier (BBB), is essential for the delivery of oxygen, nutrients, and hormones to the brain parenchyma (2) and thus dysfunction of this system via hypoperfusion (i.e., decreased cerebral blood flow) and atrophy is detrimental to neurological function (3, 4). Integrity of this BBB is maintained by tight junctions (TJ) composed of the transmembrane proteins such as occludin, claudins, zonula occuldens (ZOs), and junctional adhesion molecules (JAMs) (4, 5). As a key component of the limbic system, the hippocampus is responsible for memory formation, spatial organization, and behavioral regulation (6) though microvascular dysfunction, injury, and senescence (i.e., age-related deterioration) contribute to vascular dementia-related cognitive impairment (3). Nutrition and cognitive health have been strongly linked and consumption of diets that promote obesity development, like those high in sugar and saturated fats, can foster cognitive decline (7–9).

Metabolic syndrome (MetS) is a diet-induced cluster of conditions characterized by increased waist circumference, high triglycerides, reduced high-density lipoprotein cholesterol (HDL-C), raised blood pressure, and elevated fasting blood glucose that can operate in a concerted effort to increase the risk of type 2 diabetes, cardiovascular diseases, and cognitive dysfunction (10–13). Chronic hyperglycemia and insulin resistance, principal features of diabetes, brought on by a high glycemic diet (HGD) or a Western diet (high-fat, high-sugar) have reportedly contributed to cognitive deterioration and dementia characteristics like synaptic degeneration/plasticity impairment, neuroinflammation, memory loss/spatial learning issues, and dysfunction of glial cells (12, 14). Furthermore, hyperglycemia and other consequences of MetS establish a status of low-grade inflammation and mitochondrial oxidative stress that can contribute to endothelial dysfunction and BBB permeability, including within the hippocampus (15). Previous studies, including from members of our research group, have reported that HGD/Western diet is associated with deleterious effects on brain microvasculature due to neurovascular inflammation, apoptosis, and endothelial hyperpermeability (7, 16–19), even inducing a multi-omic effect on protein coding and non-coding genes (16, 20). A HGD and diabetes have also been linked to exacerbation of age-related detriments and major degenerative diseases like Alzheimer’s, Parkinson’s, Huntington’s, and amyotrophic lateral sclerosis (ALS) (10, 21–23) in addition to brain tumor progression (24, 25).

Dietary intervention of bioactives like polyphenols and carotenoids from fruits, vegetables, and other plant sources can act as nutraceutical solutions in alleviating metabolic syndrome-related cognitive impairment due to their antioxidant and anti-inflammatory capabilities (26–30). Curcumin is a dietary polyphenol found in the rhizome of turmeric (*Curcuma longa*), comprising ∼77% of its curcuminoid composition along with demethoxycurcumin (∼17%) and bisdemethoxycurcumin (∼3-6%), and has historically been utilized in South Asian cooking and herbal remedies (31, 32). An average daily consumption of turmeric within the Indian community is about 2–2.5 g per individual (60 kg), corresponding to 60–100 mg of curcumin (30). The three parent curcuminoids share their structure with two aromatic benzenemethoxy rings connected with a conjugated α,β-unsaturated β-diketo linker and curcumin specifically can tautomerize between keto-enol forms and exist as the keto form in neutral and acidic environments while the enol form is found in alkaline environments and the solid state (30, 33). These structural components of curcuminoids all contribute to a flexible yet hydrophobic nature that lead to poor bioavailability (34, 35), though nanoemulisons of curcumin have been developed to help facilitate absorption and bioactivity (36, 37). Once ingested, gut and liver enzymes are capable of degrading curcumin via phase I and II reactions (34). Phase I metabolism generates dihydrocurcumin, hexahydrocurcumin, tetrahydrocurcumin, and octahydrocurcumin via reduction of double bonds while phase II conjugates curcumin and metabolites with glucuronic acid and sulfate (34, 38). Phase II glucuronidation elevates solubility and thus is found predominantly in bodily fluids and organs (34). Interplay with colonic intestinal bacteria has been reported as certain species can modify curcuminoids by reduction, hydroxylation, demethylation, and demethoxylation (39) while consumption of curcumin can modulate the profile of the gut microbiota and alleviate dysbiosis associated with chronic diseases (40, 41), which may help drive the reported biological activities.

Nutraceutical bioactive effects of curcuminoids include antioxidant, anti-inflammatory, antiproliferative, and antimicrobial actions in addition to notable ones relevant to this study such as antidiabetic, cardioprotective and neuroprotective effects (30, 33, 42, 43). Furthermore, several studies have reported curcumin-mediated modulation of neurodegenerative disease severity in animal and human models (43–46). In high glycemic conditions, curcumin treatment has antidiabetic efficacy by improving insulin resistance, fasting blood sugar levels, dyslipidemia, neuropathy, and inflammatory/oxidative status (42, 47, 48). Mechanistic drivers of curcumin bioactivity relate to modulation of nuclear factor kappa B (NF-kB), nuclear factor E2-related factor (Nrf2), Janus kinase/signal transducers and activators of transcription (JAK-STAT), phosphoinositide 3-kinase (PI3K)/v-akt murine thymoma viral oncogene homolog 1 (AKT), and mammalian target of rapamycin (mTOR) pathways (30, 33, 43, 49).

*Goal of study:* The objective of this study was to assess the nutrigenomic effects of curcumin (0.2% in diet) in male wild-type mice on a high glycemic diet (HGD) and its potential impact in neurovascular function of microvessels within the hippocampus.

## 2 Materials and methods

### 2.1 Animals and diet treatments

The research in this study was performed in compliance with the Public Health Service Policy on Humane Care and Use of Laboratory Animals and all animal procedures were approved by the Institutional Animal Care and Use Committee (IACUC) at the University of California, Davis (protocol code 20943 and date of approval: 04/18/2019). Male C57BL/6J mice (*n* = 21, 19 weeks of age) were purchased from The Jackson Laboratory (Bar Harbor, ME, United States) and housed (*n* = 1 per cage) in a 12-h light/dark cycle in a temperature- and humidity-controlled environment within the University of California, Davis Mouse Biology Program. Mice were fed a standard chow diet (Teklad Custom Diets #0915, Madison, WI, United States) during the acclimation period of 1 week and then were randomly assigned to three experimental dietary intervention groups (*n* = 7/group) for 12 weeks: low glycemic diet (LGD, Teklad Custom Diets #TD.08485; 67.9% kcal of carbohydrate, 19.1% protein, 13% fat, with 12% sucrose by weight), high glycemic diet (HGD, #TD.05230, 68.7% kcal of carbohydrate, 18.7% protein, 12.6% fat, with 34% sucrose/weight), and HGD+Curc (#TD.05230 + 0.2% curcumin in diet, equivalent to 1 g/day in humans, achieved by replacing 2.0 g/kg of corn starch content in the HGD formulation with isolated curcumin); the composition of the experimental diets is provided in Supplementary Table 13. The supplementation of 0.2% curcumin has been utilized in animal studies (50, 51) and the human equivalency of 1 g/day falls in the range for recommended supplementation (52, 53). Food and water were administered *ad libitum* and consumption of experimental diets were monitored by lab members.

### 2.2 Body weight and serum tests

Body weight was recorded at the conclusion of the 12-week dietary intervention period and terminal blood was collected via ventricular puncture following an 8-h fasting period and stored at −80°C. Total cholesterol (TC), triglycerides (TG), high-density lipoprotein cholesterol (HDL-C), low-density lipoprotein cholesterol (LDL-C), insulin, and glucose levels were measured from fasted serum samples, which were isolated by centrifuging the blood at 1,500 × g for 10 min at 4°C. Levels of TC, TG, HDL-C, and LDL-C were determined via enzymatic assays from Fisher Scientific (Hampton, NH, United States) and precipitation separation assays from Abcam (Waltham, MA, United States) that were adapted for a microplate format. Serum glucose and insulin levels were measured by an enzymatic assay from Fisher Scientific (Hampton, NH, United States) and electrochemiluminescence from Meso Scale Discovery (Rockville, MD, United States), respectively, in accordance with manufacturer’s instructions. All serum analyses were carried out by the UC Davis Mouse Metabolic Phenotyping Center (MMPC) Metabolic Core.

### 2.3 Isolation, cryosection, and laser capture microdissection of murine hippocampal microvessels

**FIGURE 1:**
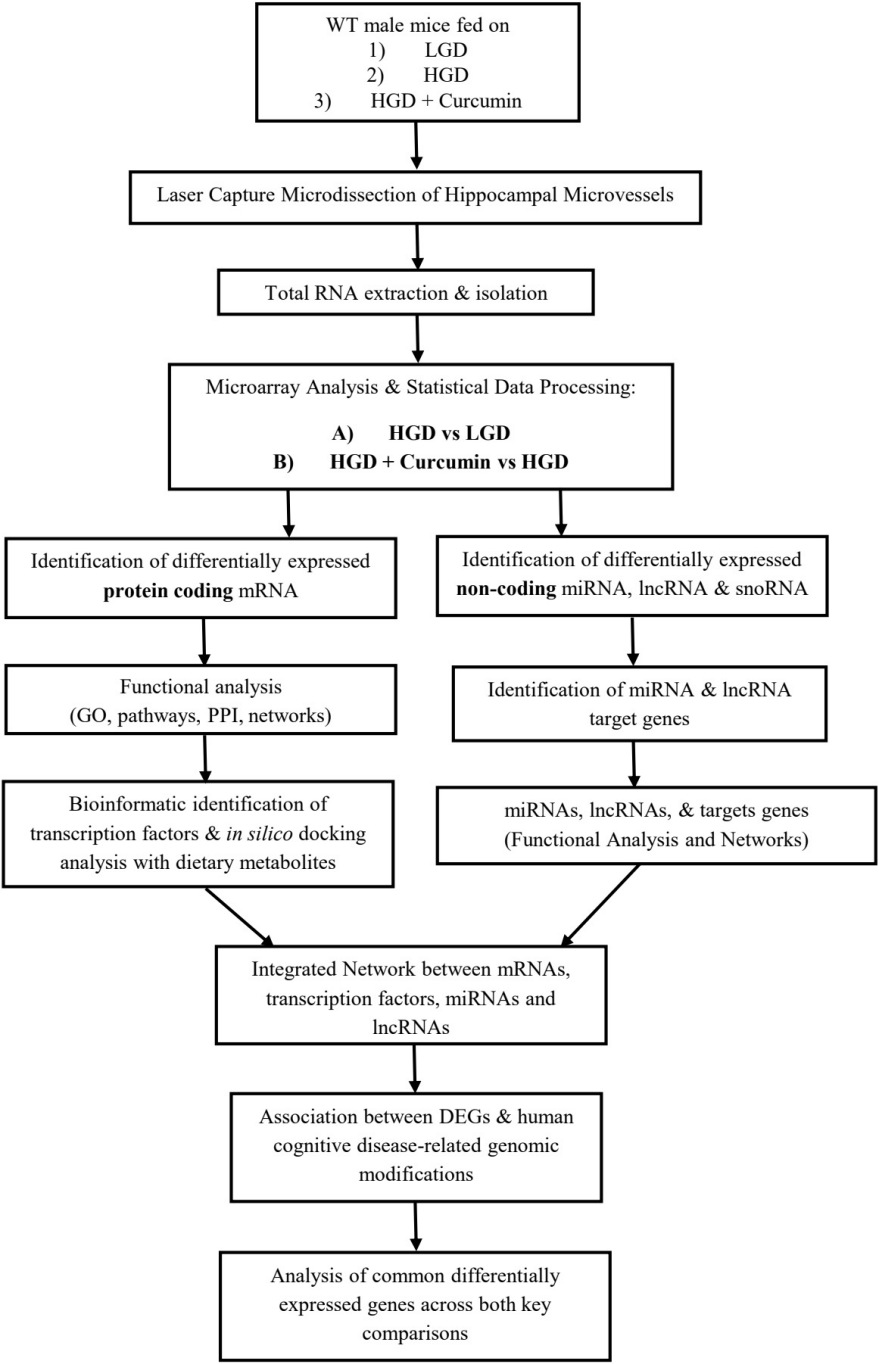
A flowchart depicting the workflow of the study, including microarray and bioinformatic analyses for the two key comparisons (A) HGD vs. LGD and (B) HGD+Curc vs. HGD. LGD, low glycemic diet; HGD, high glycemic diet; mRNA, messenger RNA; miRNA, microRNA; lncRNA, long non-coding RNA; snoRNA, small nucleolar RNA; GO, gene ontology; PPI, protein-protein interactions; DEGs, differentially expressed genes.

At the time of sacrifice following the 12-week study period, mice were anesthetized by intraperitoneal injection of xylazine/ketamine with dosing based on the amount required to achieve the surgical plane of anesthesia (24–25.5 mg/kg xylazine and 216–229.5 mg/kg ketamine) and euthanized by exsanguination. Intact brains were collected rapidly and the region containing the temporal lobe and hippocampus was isolated and embedded with HistoPrep Frozen Tissue Embedding Media (Fisher Scientific, Pittsburgh, PA, United States) under RNase-free conditions. Hematoxylin staining and microscopy visualization of the medial temporal lobe allowed for the identification of the hippocampus and hippocampal neurons, as previously reported (54). The verified hippocampal region was then coronally cryosectioned (8 μm, Leica Frigocut 2800 n Cryostat, Leica Biosystems, Buffalo Grove, IL, United States), bound to charged RNA-free PEN Membrane Glass slides, treated with RNAlater^®^-ICE (Life Technologies, Grand Island, NY, United States) for RNA preservation, and stored at −80°C for further analysis. A flowchart portraying the workflow of subsequent analyses of the hippocampus is provided in Figure 1.

Hippocampal cryosections were immersed in nuclease-free water and dehydrated in desiccant in preparation for the laser capture microdissection (LCM) of endothelial microvessels (<20 μm), which were identified via alkaline phosphatase staining with 5-bromo-4-chloro-3-indolyl phosphate/nitro blue tetrazolium chloride (BCIP/NBT) substrate (55). Extraction of the microvascular endothelium of the cryosectioned hippocampus via LCM was performed with direct microscopic visualization of the entire vessel wall using a Leica LMD6000 Laser Microdissection Microscope (Leica Microsystems, Wetzlar, Germany). The isolated microvessels largely represented the cornu ammonis CA1 and CA3 regions in dorsal segments of the hippocampus, though further regional specification was not conducted.

### 2.4 RNA extraction and microarray transcriptomic analysis of lcm-isolated hippocampal microvessels

Laser-captured hippocampal microvessels (300 microvessels/mouse, *n* = 3 mice/group) were further utilized, starting with total RNA extraction via Arcturus PicoPure™ RNA Isolation Kit (Thermo Fisher Scientific, Santa Clara, CA, United States) following the manufacturer’s instructions. The Affymetix (Santa Clara, CA) protocol for RNA quantification with SYBR Green I and ROX™ Passive Reference Dye was conducted and the RNA quality of the LCM-derived microvessels was assessed with a Nanodrop spectrophotometer.

Once extracted, RNA from the hippocampal microvessels was further processed for transcriptomic analysis with Clariom D Mouse Arrays (one per mouse) that contained over 7 million probes for protein-coding and protein non-coding genes such as microRNAs, long non-coding RNAs, and small nucleolar RNAs (Thermo Fisher, Santa Clara, CA, United States). Extracted RNA (122.3 pg/mouse) was utilized for preparation of complimentary RNA (cRNA) and single-stranded cDNA (sscDNA) with the GeneChip^®^WT Pico Kit (Thermo Fisher, Santa Clara, CA, United States). Subsequently, the sscDNA (5.5 μg) obtained from 20 μg cRNA was fragmented via uracil-DNA glycosylase (UDG) and apurinic/apyrimidinic endonuclease 1 (APE 1) and labeled by terminal deoxynucleotidyl transferase (TdT) with the biotin-linked DNA Labeling Reagent. The UC Davis Genome Center performed the hybridization, staining, and scanning of the arrays in accordance with the Thermo Fisher Scientific WT array hybridization protocol. The fragmented and labeled sscDNA samples were hybridized in the GeneChip™ Hybridization Oven 645 for 16 h at 45°C and then washed and stained with the GeneChip™ Fluidics Station 450. Finally, the microarrays were scanned with the GeneChip™ Scanner 3000 7G (Thermo Fisher Scientific, Santa Clara, CA, United States) and the Thermo Fisher Scientific Transcriptome Analysis Console software was utilized for quality control of the microarrays and data analysis. The gene expression data of the low glycemic diet (LGD) and high glycemic diet (HGD) can be found in Gene Expression Omnibus (GEO) dataset GSE185057 while the genomic data from the HGD+Curc microarrays is deposited under accession number GSE314833.

### 2.5 Bioinformatics methods

#### 2.5.1 RNA classification

The ShinyGO version 0.82 online tool^1^ (56) was utilized for the identification and classification of protein coding and non-coding RNA types (mRNA; miRNA, lncRNA, snoRNA, respectively) present in the microarray analysis with default settings applied (*p*-value cutoff: 0.05; species: mouse).

#### 2.5.2 Pathway analysis

Pathway enrichment analysis of differentially expressed genes (DEGs) was conducted via the Enrichr^2^ online bioinformatic tool (57–59) in conjunction with the Kyoto Encyclopedia of Genes and Genomes (KEGG) 2021 (60) and Wiki Pathways 2024 (61) databases (*p*-value cutoff: 0.05). Pathway histograms and pie charts were created via the horizontal bar plot and 2D pie chart options on the SR Plot website^3^ (62).

#### 2.5.3 Transcription factors and *in silico* docking

Transcription factors (TFs) potentially involved in the modulation of gene expression identified in our study were identified with the Enrichr tool through the Transcriptional Regulatory Relationships Unraveled by Sentence-based Text-mining (TRUUST) (63) and TRANScription FACtor (TRANSFAC) (64) databases (*p*-value cutoff: 0.05; species mouse). For *in silico* docking analysis, the online SwissDock^4^ tool (65, 66) was utilized to assess the molecular complementarity (i.e., binding energies) between these identified TFs and major dietary metabolites of curcumin via the Attracting Cavities approach (binding energy cutoff: < −7.0 kcal/mol) (67, 68). The SwissDock results (.doc4 files) were then imported into UCSF Chimera v1.19 (69) to visualize docking of the interaction with the lowest binding energy or other representative interactions. For curcumin and related dietary metabolites (ligands), the 2D and 3D structures were obtained from ChemSpider^5^ or PubChem^6^ (70), respectively. The 3D structures of TF proteins were acquired from the UniProt^7^ repository and electrostatic maps depicting charge distribution of the TFs were portrayed in the UCSF Chimera software when applicable. Docking analysis with SP1 could not be performed due to the 3D structure reported in UniProt at the time of this publication, which is not fully intact with high confidence in structure prediction.

#### 2.5.4 Non-coding (miRNA and lncRNA)-target gene analysis

Functional strand identification (5p/3p) of differentially expressed miRNAs was achieved through the miRBase^8^ online resource (71). Subsequently, the MicroRNA ENrichment TURned NETwork (MIENTURNET)^9^ bioinformatic tool (72) was used to determine the gene targets regulated by the relevant miRNA strands using prediction from the TargetScan mode; the minimum number of gene-target interactions threshold was set to 1. Additionally, the gene targets of differentially expressed lncRNAs were obtained with the lncRRIsearch^10^ online tool using Ensembl identification numbers (73) (species: mouse; minimum of energy threshold: < −20 kcal/mol). Finally, gene targets of miRNA or lncRNAs were submitted to Enrichr for pathway ontology analysis with the KEGG 2021 and Wiki Pathways 2024 databases.

#### 2.5.5 Comparative analysis and network mapping

Venn diagrams for comparisons between multi-level genomic regulation (i.e., different RNA types) by dietary intervention groups were generated via InteractiVenn^11^ (74). STRING^12^ (Protein-Protein Interaction Networks Functional Enrichment Analysis) software v12.0 (75) was utilized to construct a network of experimentally determined and predicted protein-protein interactions between coding DEGs (high confidence: 0.700). Furthermore, network maps between DEGs (mRNA, miRNA, lncRNAs) and their respective targets and/or functional pathways were assembled in the Cytoscape^13^ v3.10.3 software (76). The heatmap and correlation plot representing the fold change values between the HGD/LGD and HGD+Curc/HGD comparisons were generated through the heatmap with Ward clustering and Spearman scatter plot options, respectively, on the SR plot website.

#### 2.5.6 Human disease association

Significant associations between identified differentially expressed genes and human diseases (nervous system and neurodegenerative) were assessed with the Comparative Toxicogenomics Database^14^ (CTD) (77) (corrected *p-*value cutoff: 0.05) and the Genome-Wide Association Studies (GWAS) Catalog^15^ (78); GWAS catalog numbers for neurodegenerative (EFO_0005772) and nervous system (EFO_0000618) diseases were utilized. The bubble plot for CTD results was generated with the enrichment bubble option in the SR plot website whereas the GWAS Venn diagram was created via InteractiVenn.

### 2.6 Statistical methods

For microarrays, statistical analysis of microvessel transcriptomes was conducted using ANOVA ebayes (Thermo Fisher Scientific Transcriptome Analysis Console software, Santa Clara, CA) with false discovery rate (FDR) correction. Differentially expressed genes (DEGs) from the microarray with significant *p* < 0.05 were considered as significantly differentially expressed. Diet intervention effects on body weight, lipid levels, glucose, and insulin were expressed as means ± standard error of the mean (SEM). Statistical significance (*p* ≤ 0.05) was assessed using unpaired *t*-tests (GraphPad software, La Jolla, CA, United States), or the Mann–Whitney test for non-normally distributed data.

## 3 Results

### 3.1 Biochemical data: effect of diets on body weight and serum parameters

**FIGURE 2:**
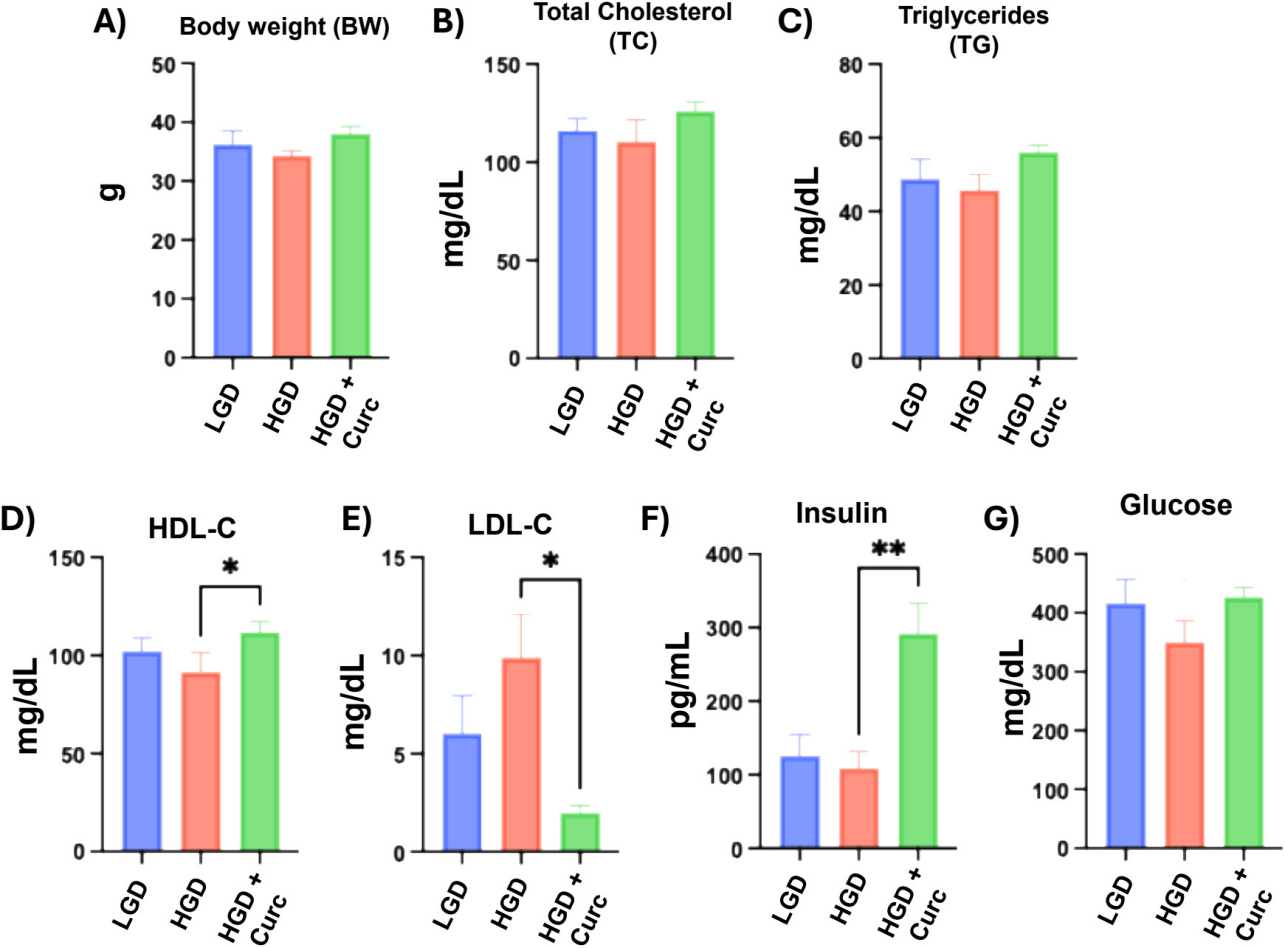
Body weight and circulatory measurements following 12 weeks of diet intervention. **(A)** Mean total body weight (g); **(B–G)** Bar graphs denoting serum levels of **(B)** total cholesterol (TC, mg/dL), **(C)** triglycerides (TG, mg/dL), **(D)** high-density lipoprotein cholesterol (HDL-C, mg/dL), **(E)** low-density lipoprotein cholesterol [LDL-**(C)**, mg/dL], **(F)** insulin (pg/mL), and **(G)** glucose (mg/dL). All endpoints were analyzed by one-way ANOVA with *post-hoc* Tukey HSD. Values are mean ± standard error of the mean (SEM); statistical significance *(*p* < 0.05) and **(*p* < 0.01).

At the conclusion of the 12-week diet intervention period, overall body weight amongst the LGD and HGD controls did not differ and the HGD+Curc treatment did not have a significant effect (Figure 2A). Similarly, serum levels of total cholesterol (Figure 2B) and triglycerides (Figure 2C) were unchanged across the three study groups. Notably, HGD+Curc significantly increased high-density lipoprotein cholesterol (HDL-C) levels (91.39 ± 26.35 vs. 11.83 ± 14.43 mg/dL, *p* < 0.05) (Figure 2D) and decreased low-density lipoprotein cholesterol (LDL-C) (9.89 ± 5.86 vs. 1.98 ± 0.76 mg/dL, *p* < 0.05) compared to HGD alone (Figure 2E). In addition, HGD+Curc elevated circulatory insulin (108.84 ± 61.14 vs. 291.43 ± 112.59 mg/dL, *p* < 0.01) significantly (Figure 2F) but did not affect glucose levels (Figure 2G) in relation to LGD and HGD.

### 3.2 HGD/LGD: effect of the high glycemic diet on male murine hippocampal microvasculature

**FIGURE 3:**
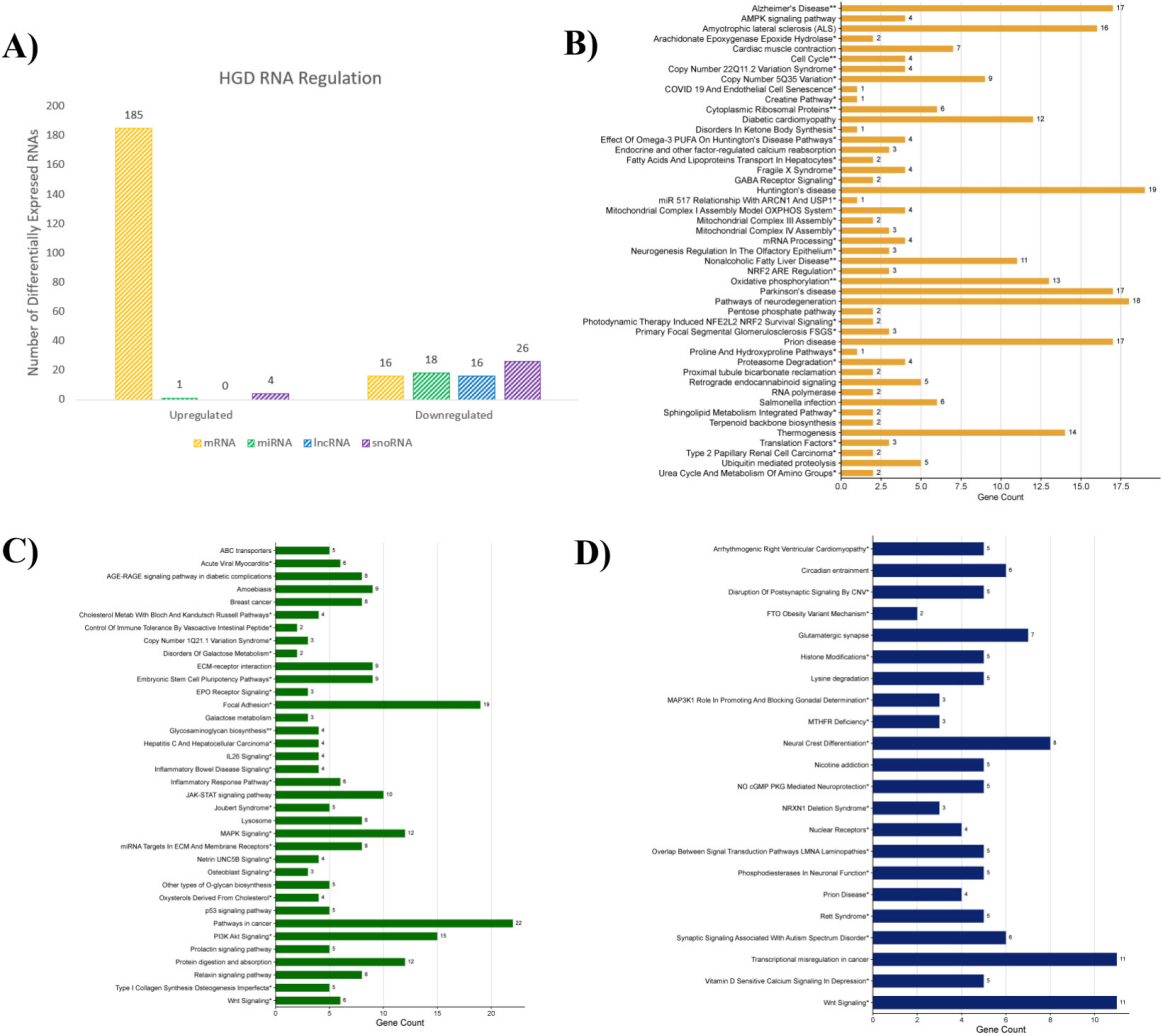
HGD genomic regulation on murine brain hippocampal microvasculature. **(A)** Bar graph depicting the number of differentially expressed coding (mRNA, orange) and non-coding (miRNAs, green; lncRNAs, blue; snoRNAs, purple) genes that were up-/down-regulated by the HGD intervention compared to LGD. **(B–D)** Histograms of functional pathways involving **(B)** coding mRNA genes, **(C)** miRNA gene targets, and **(D)** lncRNA gene targets; statistically regulated pathways (*p* < 0.05) were identified using the Enrichr online database tool- KEGG (no asterisk), Wiki Pathways*, common to both databases**.

Statistical analysis of the microarray data revealed that the HGD treatment compared to LGD modulated 786 differentially expressed genes (DEGs) in male murine hippocampal microvessels (Supplementary Table 1). More specifically, 201 protein-coding genes and 65 non-coding genes were regulated by the HGD treatment; the latter category included 19 microRNAs (miRNAs), 16 long non-coding RNAs (lncRNAs), and 30 small nucleolar RNAs (snoRNAs). Of these characterized genes, 190 were upregulated and 76 were down-regulated by HGD intervention compared to LGD (Figure 3A). Furthermore, the remaining 520 DEGs were categorized as pseudogenes, multi-complex genes, or unassigned genes with symbols (known) or without symbols (unidentified).

Amongst those classified, a total of 201 protein-coding genes were differentially expressed by HGD relative to LGD, which were mostly upregulated (*n* = 185; fold change range 1.5–11.4), while a few underwent downregulation (*n* = 16; fold change range of −4.38 to −1.5) (Supplementary Table 2). Bioinformatic analysis to uncover gene and pathway ontology indicated that the coding DEGs were involved in pathway regulation of major neurodegenerative diseases like Huntington’s, Parkinson’s, Alzheimer’s, and prion diseases and amyotrophic lateral sclerosis (ALS) as well as cellular metabolism (e.g., mitochondrial complex assembly, oxidative phosphorylation, and thermogenesis) (Figure 3B).

The hippocampal microarray analysis indicated that the HGD treatment also differentially expressed non-coding RNAs such as miRNAs, lncRNAs, and snoRNAs in comparison to LGD. Firstly, the 19 modulated miRNAs were primarily downregulated (*n* = 18; fold change (fc) range of −3.93 to −1.5) while only one (*mmu-miR-692*) was upregulated with a fold change of 4.97 (Supplementary Table 3). Targets of the modulated miRNAs totaled 527 genes that were involved in phosphoinositide 3-kinase (PI3K)/v-akt murine thymoma viral oncogene homolog 1 (AKT), mitogen-activated protein kinase (MAPK), and Janus kinase/signal transducer and activator of transcription (JAK/STAT) signaling pathways and extracellular matrix (ECM) maintenance such as ECM-receptor interactions and cytoskeletal focal adhesion (Figure 3C). Furthermore, all 16 differentially expressed lncRNAs were downregulated by HGD relative to LGD with a fold change range of −6.23 to −1.51 (Supplementary Table 4). Identification of 542 genes targeted by differentially expressed lncRNAs were involved in neuronal function pathways like neural crest differentiation, nitric oxide (NO)/cyclic guanosine monophosphate (cGMP)/protein kinase G (PKG) mediated neuroprotection, and phosphodiesterases in addition to synaptic signaling (Wingless-related integration site (Wnt) and glutamatergic synapse regulation) (Figure 3D). All pathways significantly regulated (*p* < 0.05) by HGD intervention relative to LGD, organized by identification with the Kyoto Encyclopedia of Genes and Genomes (KEGG) and Wiki Pathways databases, have been provided in Supplementary Figure 1 for coding genes (1A) as well as gene targets for miRNAs (1B) and lncRNAs (1C). Finally, relative expressions of the 30 snoRNAs were primarily downregulated (*n* = 26; fc −3.27 to −1.51) with some upregulation present (*n* = 4; fc 1.92–19.32) (Supplementary Table 5). Relevant target genes and pathways of these snoRNAs were not unveiled with literature searches and bioinformatic tools.

### 3.3 HGD + curcumin/HGD: nutraceutical effect of the high glycemic diet + curcumin on the hippocampal microvascular genome

**FIGURE 4:**
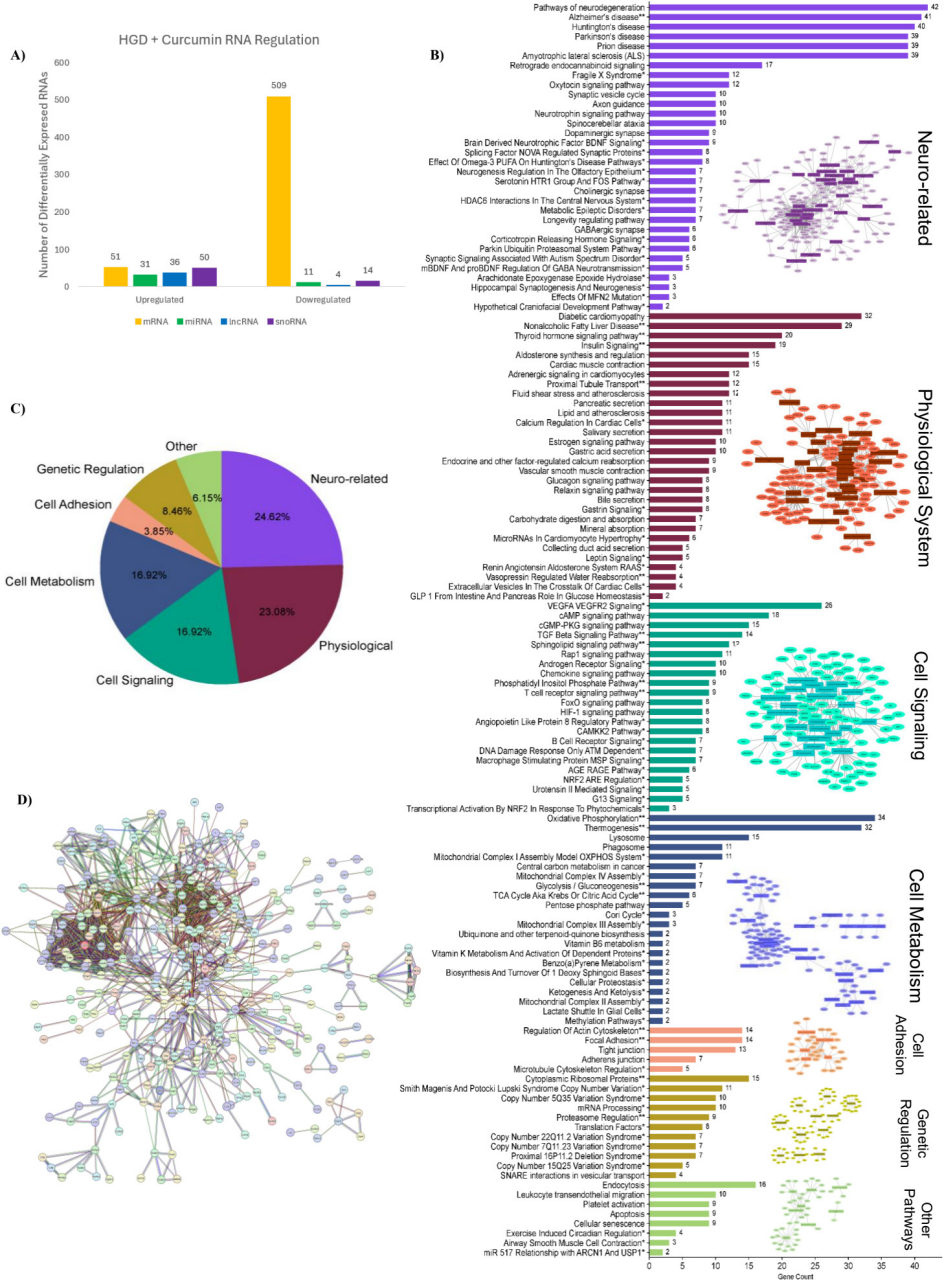
HGD + Curcumin intervention on the murine hippocampal microvascular genome involving protein-coding genes. **(A)** Bar graph portraying the number of differentially expressed protein coding (mRNA, orange) and non-coding (miRNAs, green; lncRNAs, blue; snoRNAs, purple) genes up-/down-regulated by HGD+Curc versus HGD treatment. **(B)** Histogram of pathways for differentially expressed coding genes organized by gene count and pathway type accompanied by Cytoscape network maps of pathways (rectangles) and respective coding genes (circles); neuro-related (violet), physiological system (burgundy), cell signaling (teal), cell metabolism (blue), cell adhesion (peach), genetic regulation (mustard yellow), and other (light green); statistically regulated pathways (*p* < 0.05) were identified using the Enrichr online database tool- KEGG (no asterisk), Wiki Pathways*, common to both databases**. **(C)** Pie chart of pathway types and respective percentages for coding genes. **(D)** STRINGv12 interaction map of coding genes: experimentally determined (pink), curated databases (light blue), gene neighborhood (green), gene co-occurence (dark blue), gene fusions (red), co-expression (black), protein homology (purple), text mining (mentioned together in Pubmed abstracts; yellow).

Microarray analysis was also conducted with the curcumin supplemented group to uncover how this dietary polyphenol influenced the genome of the murine hippocampal microvessels that were exposed to a high glycemic load. Statistical analysis of the microarray data demonstrated that 1,887 DEGs were affected by HGD+Curc treatment relative to the HGD control group (Supplementary Table 6). The DEGs identified with gene symbols included 560 protein coding and 146 non-coding genes (42 miRNAs, 40 lncRNAs, and 64 snoRNAs). Within those classified into these categories, 168 genes were upregulated and 538 downregulated by HGD+Curc in comparison to HGD (Figure 4A). The remaining 1,181 DEGs were categorized as pseudogenes, multi-complex genes, immunoglobulin (Ig) variable chain genes, or unassigned genes with gene symbols (known) or without symbols (unidentified).

#### 3.3.1 Influence of HGD+Curc on coding mRNA

The protein coding genes differentially expressed by HGD+Curc were primarily downregulated (*n* = 509; fold change −32.64 to −1.5) with some upregulation (*n* = 51; fold change 1.51–9.68) compared to the HGD group (Supplementary Table 7). Pathway enrichment analysis demonstrated that coding DEGs influenced by HGD+Curc were involved in various neurological and physiological system associated functions as well as pathways of cellular signaling, metabolism, genetic regulation and adhesion (Figure 4B). Cognitive and neurological related functions, which took up 24.62% of overall significantly regulated pathways (Figure 4C), involving the coding DEGs were predominately associated with neurodegenerative diseases (e.g., Huntington’s, Parkinson’s, and Alzheimer’s diseases; prion disease; ALS) in addition to signaling of hormones (oxytocin, corticotropin) and/or growth factors like brain-derived neurotrophic factor (BDNF). Pathways concerning other physiological processes (23.08%) were identified such as diabetic cardiomyopathy, non-alcoholic fatty liver disease (NAFLD), and signaling of insulin and thyroid hormones. Cellular transduction pathways (16.92%) were involved in vascular endothelial growth factor and receptor VEGF/VEGFR2, cAMP/cGMP-PKG, and transforming growth factor-beta (TGF-β) signaling while functionality of coding DEGs in cellular metabolism (16.92%) were heavily implicated with the regulation of oxidative phosphorylation and thermogenesis. Though a small portion but important for endothelial maintenance, pathways related to cellular adhesion (3.85%) included the maintenance of the actin/microtubule cytoskeleton, focal adhesion, and tight/adherens junctions. Pathways of genetic regulation (8.46%) like mRNA processing, translation factors, and regulation of ribosomal proteins were affected, which is characteristic of this protein coding level of nutrigenomic modulation. Additionally, some identified genes related to chromosomal abnormalities such as copy number variations (CNV) and proximal deletions, influenced by dietary intervention. Finally, coding DEGs modulated by HGD+Curc were also involved in other regulatory pathways (6.15%) related to cellular processes like endocytosis, apoptosis, and senescence.

The STRING map highlights the potential interactions between differentially expressed protein coding genes (Figure 4D). Twenty eight of the 560 coding genes participated in 25 or more interactions (Table 1), the highest amongst them with 38 interactions being ATP synthase subunit alpha (*Atp5a1*). These highly interactive coding genes were involved in pathways of neurodegenerative diseases, diabetic complications (i.e., NAFLD and cardiomyopathy), and cellular metabolic processes of oxidative phosphorylation and thermogenesis.

**TABLE 1:** Genes with 25 or more interactions in the STRING map of HGD+Curc DEGs in Figure 4.

Symbol	Gene name	Count
Atp5a1	ATP synthase subunit alpha, mitochondrial	38
Eef2	Elongation factor 2	34
Cox5a	Cytochrome c oxidase subunit 5A, mitochondrial	33
Rpl4	60S ribosomal protein L4	32
Uqcrc2	Cytochrome b-c1 complex subunit 2, mitochondrial	32
Actb	Actin, cytoplasmic 1, N-terminally processed	31
Cox4i1	Cytochrome c oxidase subunit 4 isoform 1, mitochondria	31
Gm11808	Ubiquitin-60S ribosomal protein L40	31
Uqcrc1	Cytochrome b-c1 complex subunit 1, mitochondrial	31
Cox5b	Cytochrome c oxidase subunit 5B	30
Ndufs3	NADH dehydrogenase [ubiquinone] iron-sulfur protein 3, mitochondrial	30
Rpl8	60S ribosomal protein L8	29
Rps15	40S ribosomal protein S15	29
Sdhb	Succinate dehydrogenase [ubiquinone] iron-sulfur subunit, mitochondrial	29
Hsp90ab1	Heat shock protein HSP 90-beta	28
Cox6a1	Cytochrome c oxidase subunit 6A1, mitochondrial	27
Ndufa4	Cytochrome c oxidase subunit NDUFA4	27
Ndufa9	NADH dehydrogenase [ubiquinone] 1 alpha subcomplex subunit 9, mitochondrial	27
Ndufv2	NADH dehydrogenase [ubiquinone] flavoprotein 2, mitochondrial	27
Uqcr11	Cytochrome b-c1 complex subunit 10	27
Ndufb7	NADH dehydrogenase [ubiquinone] 1 beta subcomplex subunit 7	26
Ndufs4	NADH dehydrogenase [ubiquinone] iron-sulfur protein 4, mitochondrial	26
Rpl3	60S ribosomal protein L3	26
Rplp0	60S acidic ribosomal protein P0	26
Sdha	Succinate dehydrogenase [ubiquinone] flavoprotein subunit, mitochondrial	26
Arl6ip1	ADP-ribosylation factor-like protein 6-interacting protein 1	25
Eif5a	Eukaryotic translation initiation factor 5A-1	25
Ndufa13	NADH dehydrogenase [ubiquinone] 1 alpha subcomplex subunit 13	25

#### 3.3.2 Transcription factors and *in silico* docking of curcumin metabolites

**FIGURE 5:**
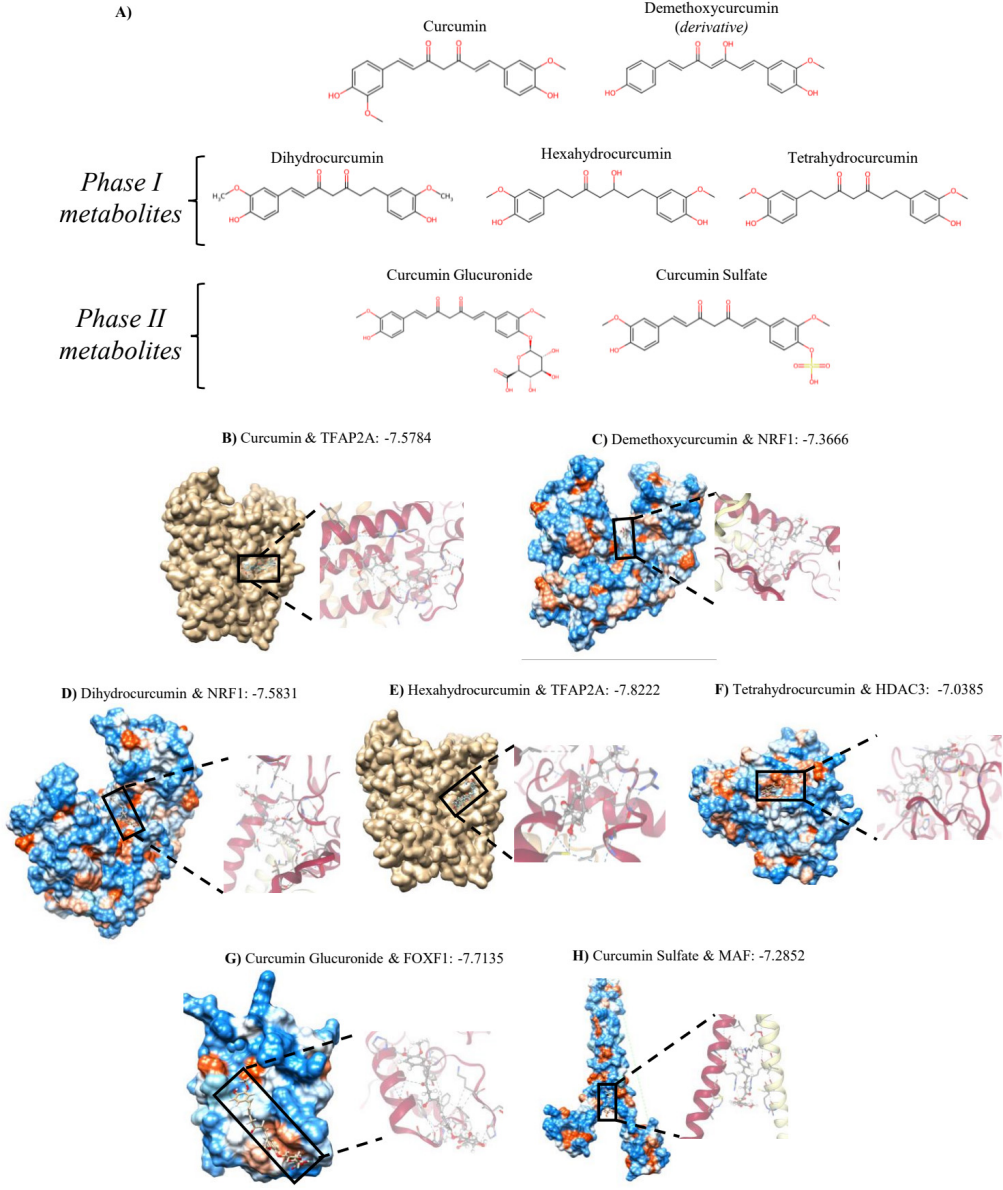
*In silico* docking of curcumin-related metabolites to transcription factors (TFs). **(A)** Skeletal 2D structures of curcumin and major dietary curcumin-derived metabolites. **(B–G)** Representative docking interactions between metabolites and TF with significant binding energies, **(B)** curcumin and TFAP2A, **(C)** demethoxycurcumin and NRF1, **(D)** dihydrocurcumin and NRF1, **(E)** hexahydrocurcumin and TFAP2A, **(F)** tetrahydrocurcumin and HDAC3, **(G)** curcumin Glucuronide and FOXF1, and **(H)** curcumin Sulfate and MAF; 3D visualization via UCSF Chimera v1.19 (left) and SwissDock (right).

Another level of nutrigenomic modulation by curcumin consumption was observed in this study through the interactions between protein coding DEGs modulated by HGD+Curc and potential transcription factors (TFs) whose activity could be affected by curcumin and underlying observed genomic changes. Seven statistically significant TFs were identified (*p* < 0.05) included CREB1, SP1, FOXF1, NRF1, MAF, TCF12, HDAC3, and TFAP2A (Table 2). The largest number of coding DEGs (*n* = 33) was associated with CREB1, while the most significant relationship (*p* = 0.0011) was with MAF, which was likely due to *Maf* itself being a DEG. To further investigate how curcumin consumption potentially influenced TF activity, *in silico* docking analysis was performed to determine binding energies between the identified TFs and major dietary metabolites or derivatives of curcumin. The structures of curcumin and the related compounds of demethoxycurcumin, dihydrocurcumin, hexahydrocurcumin, tetrahydrocurcumin, curcumin glucuronide, and curcumin sulfate are provided in Figure 5A as well as their respective binding energies to the putative TFs in Table 3. Most metabolites had significant docking (<−7 kcal/mol) to one or more TF, with the lowest binding energy consistently being with TFAP2A, with the exception of tetrahydrocurcumin and NRF1. Among the metabolites, demethoxycurcumin and curcumin glucuronide showed potential interactions with the highest number of proteins. Representative depictions of significant docking amongst the dietary compounds and TFs are provided in Figures 5B–H.

**TABLE 2:** Potential transcription factors (TFs) involved in gene regulation by HGD + Curc intervention.

TF (symbol)	Name	UniProt ID	*P*-value	Gene count
CREB1**	cAMP response element-binding protein 1	Q01147	0.036512	33
SP1*	Specificity Protein 1	O89090	0.004082	16
FOXF1**	Forkhead box protein F1	Q61080	0.030939	11
NRF1*	Nuclear respiratory factor 1	Q9WU00	0.002978	4
MAF*	MAF bZIP	P54843	0.001101	3
TCF12*	Transcription factor 12	Q61286	0.014969	2
HDAC3*	Histone deacetylase 3	O88895	0.014969	2
TFAP2A*	Transcription factor AP-2 alpha (Activating enhancer binding Protein 2 alpha)	P34056	0.030344	2

Table organized by number of protein coding genes influenced by TF activity identified using the Enrichr online database tool;

TRRUST*,

TRANSFAC**.

**TABLE 3:** Binding energy of major dietary curcumin-related metabolites to transcription factors (TFs) according to the online SwissDock *in silico* docking tool.

Dietary metabolites	Binding energy to TFs (kcal/mol)						
	CREB1	FOXF1	HDAC3	MAF	NRF1	TCF12	TFAP2A
Curcumin	–6.1655	–6.4461	–7.0956	–7.1561	–7.4551	–7.1376	–7.5784
Demethoxycurcumin	–6.1034	–6.3629	–6.4598	–6.9189	–7.3666	–6.6069	–7.6787
Dihydrocurcumin	–6.136	–6.6939	–6.9701	–6.9603	–7.5831	–6.9597	–7.8057
Hexahydrocurcumin	–5.9812	–6.8071	–6.9923	–6.9079	–7.2298	–6.8901	–7.8222
Tetrahydrocurcumin	–6.1399	–6.4305	–7.0385	–7.151	–7.5008	–6.8022	–7.3203
Curcumin glucuronide	–7.1127	–7.7135	–7.2736	–7.2426	–8.034	–7.1559	–8.8235
Curcumin sulfate	–6.966	–6.7967	–7.0248	–7.2852	–7.9576	–6.833	–8.9096

#### 3.3.3 HGD+Curc non-coding miRNA

**FIGURE 6:**
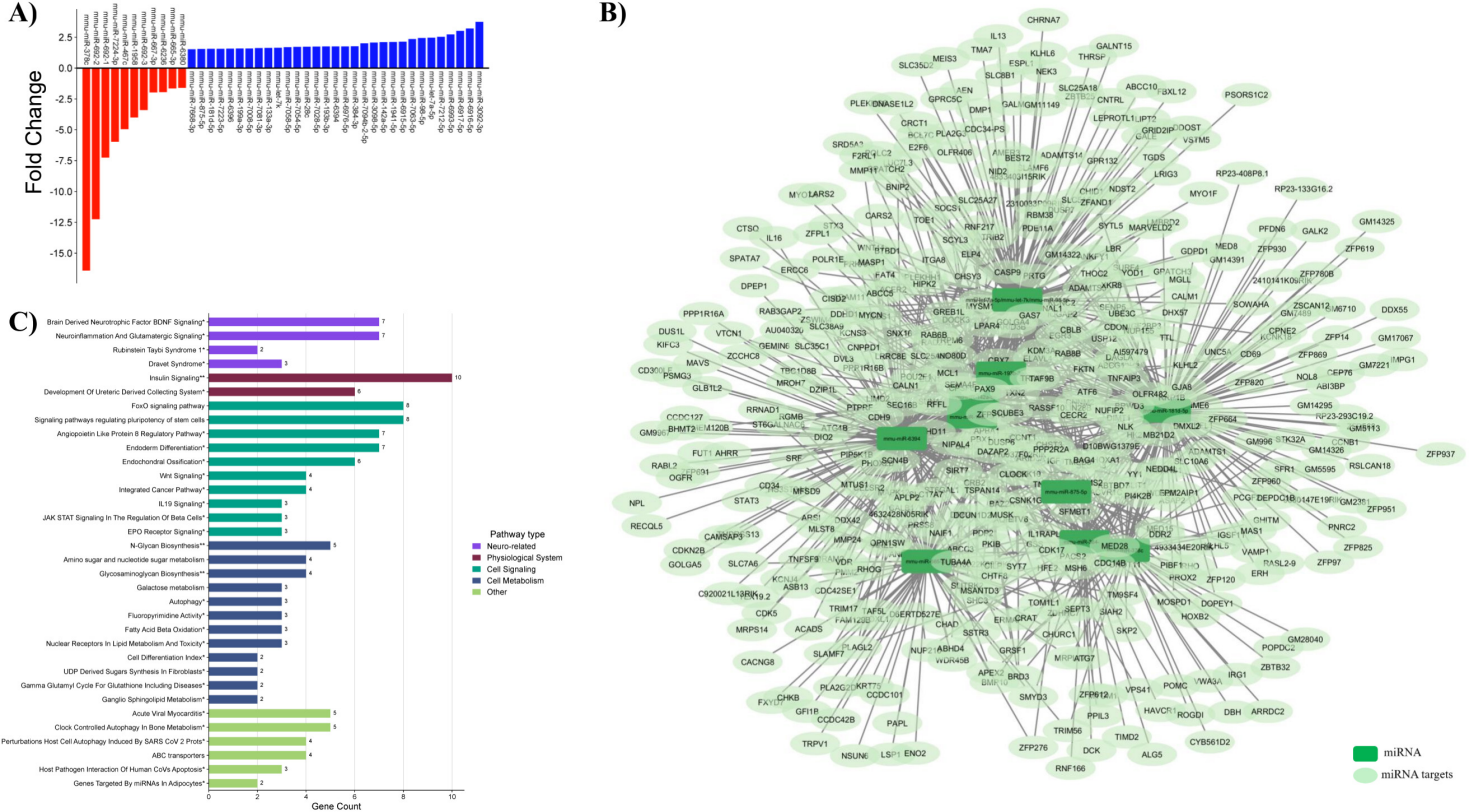
Curcumin modulation of hippocampal non-coding miRNAs and targets. **(A)** Fold changes depicting upregulation or downregulation of differentially expressed miRNAs by HGD+Curc compared to HGD. **(B)** Network map of miRNAs (dark green rectangles) and their respective gene targets (light gene circles) generated via Cytoscape. **(C)** Histogram of functional pathways for miRNA gene targets categorized by gene count and pathway type; statistically regulated pathways (*p* < 0.05) were identified using the Enrichr online database tool KEGG (no asterisk), Wiki Pathways*, common to both**.

As seen with the HGD, several categories of non-coding RNAs were differentially expressed in the hippocampal microvessels by curcumin supplementation. Firstly, 42 miRNAs were modulated by HGD+Curc in comparison to HGD, of which 31 were upregulated (fold change range of 1.53–3.75) and 11 were downregulated (fold change range of −16.41 to −1.6) (Figure 6A and Supplementary Table 8A). Bioinformatic analysis identified 470 target genes for only 12 of the 42 DE miRNAs (*p* < 0.05), 10 of such miRNAs were upregulated (fold change 1.55–2.46) and two were downregulated (fold change −16.41 to −1.65) (Supplementary Figure 4A). A network map of the participating miRNAs and respective gene targets is provided in Figure 6B, demonstrating that genes are associated with up to eight miRNAs. In particular, three miRNAs were grouped together (*mmu-let-7a-5p, -let-7k, and -miR-98-5p*) in a network node as these are members of the *let-7* family of miRNAs, which were upregulated (fold change 2.46, 1.65, 2.44, respectively) and associated with the largest subset (*n* = 179) of target genes (Supplementary Table 8B). Functions of overall genes targeted by the DE miRNAs were related to brain-derived neurotrophic factor (BDNF), forkhead box O (FoxO), and insulin signaling as well as the angiopoietin-like protein-8 regulatory pathway (Figure 6C), which were all pathways in common with the coding genes differentially expressed by HGD+Curc as mentioned previously. Additional pathways that were exclusive to miRNA targets were related to regulation of stem cell pluripotency, neuroinflammation, and glutamatergic signaling as well as biosynthesis of N-glycans and glycoaminoglycans (GAGs) and metabolism of galactose and amino/nucleotide sugars.

#### 3.3.4 HGD+Curc non-coding lncRNA

**FIGURE 7:**
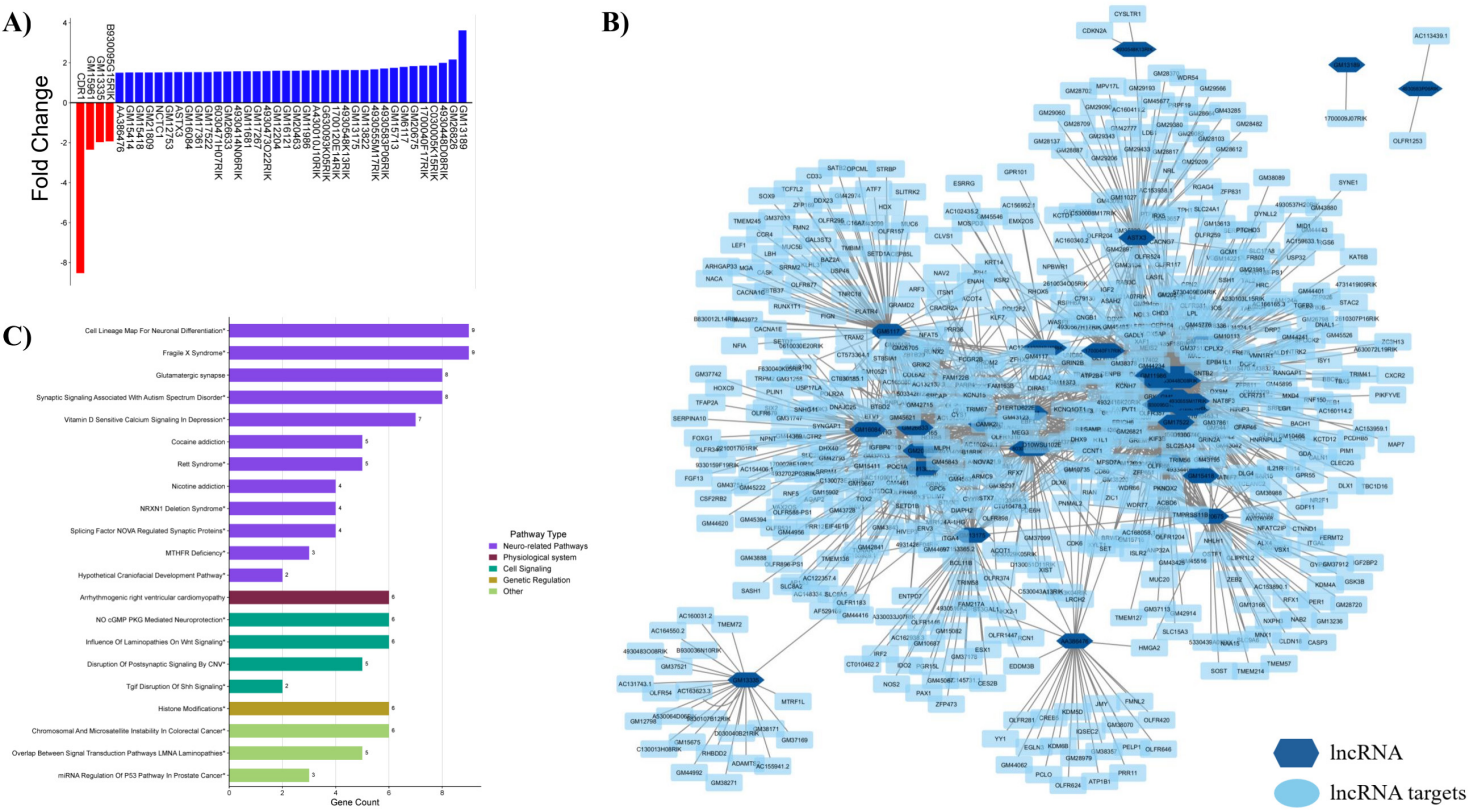
Curcumin modulation of hippocampal non-coding lncRNAs and targets. **(A)** Upregulation or downregulation of differentially expressed lncRNAs by HGD+Curc compared to HGD. **(B)** Cytoscape network map of lncRNAs (dark blue hexagons) and their respective gene targets (light blue rectangles). **(C)** Categorized histogram of lncRNA gene targets organized via gene count and pathway type; statistically regulated pathways (*p* < 0.05) were identified using the Enrichr online database tool- KEGG (no asterisk), Wiki Pathways*.

Curcumin supplementation also modulated 40 lncRNAs in the hippocampal microvessels, the majority of which were upregulated (*n* = 36; fold change 1.5–3.62) while only four were downregulated (fold change: −8.53 to −1.93) (Figure 7A and Supplementary Table 9). The majority of DE lncRNAs (82.5%) were discovered to have a total of 656 gene targets and depicted in the network map provided in Figure 7B. Pathway enrichment analysis shows that these lncRNA targets were predominantly involved with neurological functionalities. However, these genes regulated by lncRNAs were associated with neurodevelopmental disorders, rather than neurodegenerative diseases, such as fragile X syndrome, autism, Rett syndrome, methylenetetrahydrofolate reductase (MTHFR) deficiency, the hypothetical craniofacial development pathway, postsynaptic signaling disruption by copy number variations (CNV), and T-box G-rich interacting factor (*Tgif*) disruption of Sonic Hedgehog (Shh) signaling (Figure 7C). Other significantly regulated pathways of lncRNA target genes included neuronal differentiation, glutamatergic synapse regulation, and NO/cGMP/PKG neuroprotection. Alternatively organized by database (i.e., KEGG and Wiki Pathways) and alphabetically, significantly regulated pathways (*p* < 0.05) by HGD+Curc compared to HGD alone are provided for coding DEGs in addition to gene targets for miRNAs and lncRNAs in Supplementary Figures 2–4, respectively.

#### 3.3.5 HGD+Curc non-coding snoRNA

Finally, non-coding snoRNAs (*n* = 64) were also differentially regulated by HGD+Curc intervention, of which 50 were upregulated (fold change 1.5–4.72) and the other 14 were downregulated (fold change −16.58 to −1.54) (Supplementary Table 10). Target genes and pathways of these snoRNAs were not observed with literature searches and bioinformatic tools, though a few C/D box snoRNAs (*Snords*) were identified. Notably, *Snord16a* and *Snord59a* were downregulated (fc: −4.07) and upregulated (fc: 1.59), respectively, by HGD+Curc.

#### 3.3.6 HGD+Curc: integrative analysis of coding DEGs and non-coding gene targets

**FIGURE 8:**
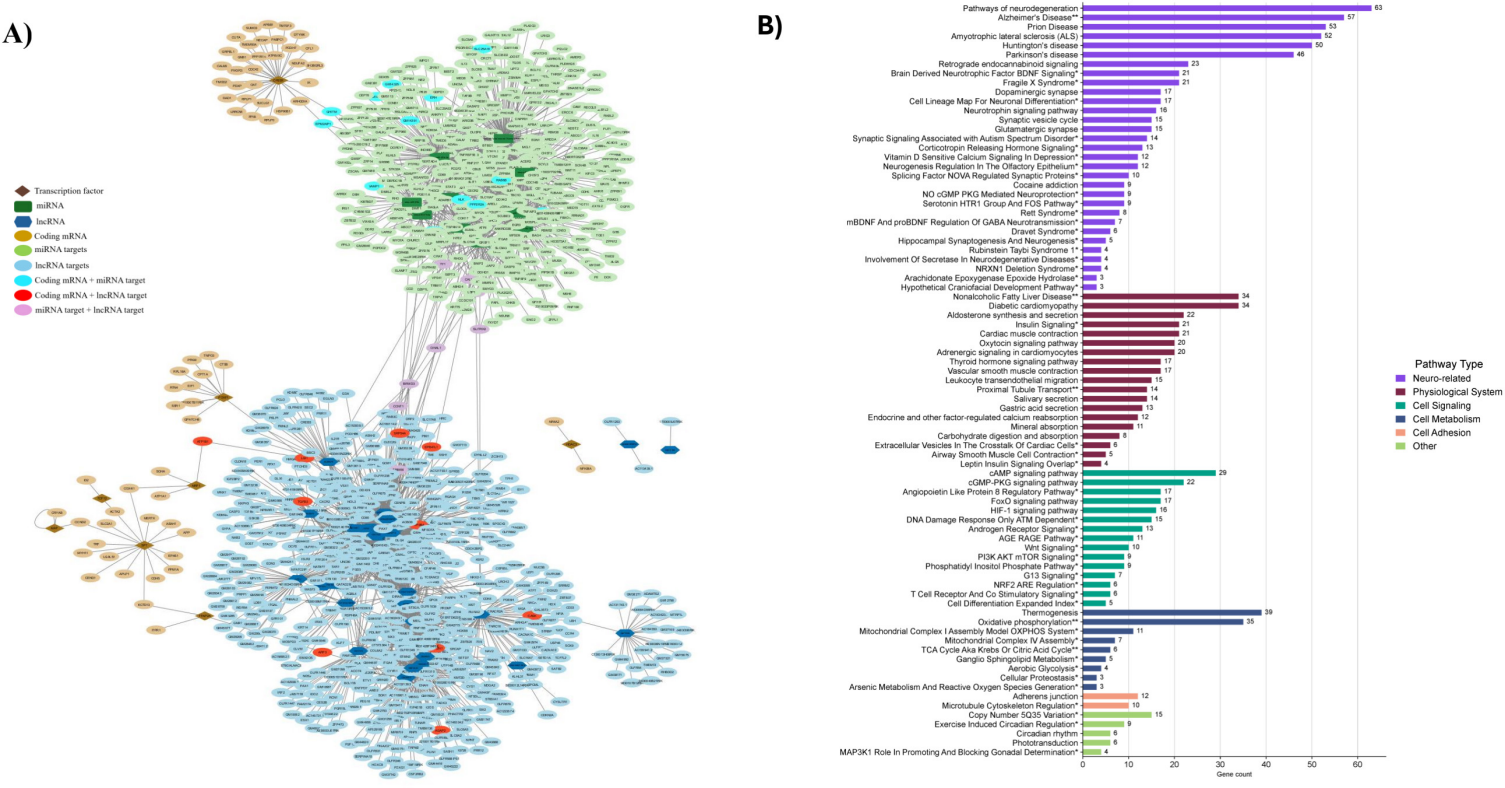
Global interactions and pathways of differentially expressed coding genes and non-coding gene targets by HGD+Curc in the murine hippocampal microvasculature. **(A)** Cytoscape network map of coding mRNA (light brown circles), transcription factors (brown diamonds), miRNAs (dark green rectangles) and miRNA gene targets (light green circles), lncRNAs (dark blue hexagons) and lncRNA gene targets (light blue circles), genes that are coding mRNA + miRNA targets (teal circles), coding mRNA + lncRNA targets (red circles), miRNA targets + lncRNA gene targets (purple circles). **(B)** Histogram of pathways involving coding genes in addition to gene targets of non-coding miRNAs and lncRNA categorized by gene count and pathway type; statistically regulated pathways (*p* < 0.05) were identified using the Enrichr online database tool- KEGG (no asterisk), Wiki Pathways*, common to both databases**.

In order to further look into the multigenomic influence of HGD+Curc dietary intervention on the hippocampal microvasculature, we generated an integrated network of differentially expressed protein coding mRNAs, potential regulatory TFs, and non-coding miRNAs and lncRNAs in addition to their respective gene targets (Figure 8A). Targets were clustered around TFs and non-coding RNAs with some overlap, indicating that some genes undergo multi-level regulation through more than one RNA type. For example, various genes were identified as protein coding DEGs in addition to targets of miRNAs (*n* = 13; *Aplp2, Dram2, Epm2aip1, Erh, Ghitm, Gm14325, Gm14326, Gm14391, Nlk, Ppp2r2a, Rab6b, Slc25a18, Vamp1)* or lncRNAs (*n* = 10; *Agap2, Arf3, Atp1b1, Atp2b4, Camk2n1, Cask, Epb41l1, Lgi1, Srp54a, Tgfb3*) (Supplementary Figure 5A). Likewise, a few genes were targets of both miRNAs and lncRNAs (*n* = 8; *Baz2a, Brwd3, Caln1, Ccnt1, Dnal1, Slitrk2, Trim56, Yy1*) though no genes identified through bioinformatic tools were common amongst coding DEGs and targets of non-coding multi-regulation (i.e., all three groups).

Integrated pathway enrichment of all differentially expressed coding genes as well as gene targets of miRNAs and lncRNAs was conducted to gain insight on the global, multigenomic regulation induced by HGD+Curc intervention on the hippocampal microvessels (Figure 8B). Consistently, a large portion of coding DEGs and non-coding gene targets were involved with pathways of neurodegenerative diseases (Alzheimer’s, Huntington’s, Parksinson’s, ALS, prion disease) as well as BDNF and retrograde endocannabinoid neuronal signaling. Furthermore, diabetic sequelae such as cardiomyopathy, NAFLD, and interactivity of insulin and aldosterone were influenced by HGD+Curc consumption. Several transduction pathways like cAMP/cGMP-PKG, FoxO, hypoxia-inducible factor-1 (HIF-1), and angiopoietin-like protein 8 signaling as well as cellular metabolic processes of thermogenesis and oxidative phosphorylation were significantly modulated. As for endothelial adhesion maintenance, genes involved in microtubule and adherens junction regulation were identified. Finally, some DEGs were involved in other pathways regulating copy number variations, circadian rhythm, and phototransduction. Overlaps between pathways as well as common coding DEGs and/or non-coding gene targets across both HGD and HGD+Curc are provided in Supplementary Figures 5B,C, respectively.

#### 3.3.7 Disease associations with genes differentially expressed by HGD+Curc

**FIGURE 9:**
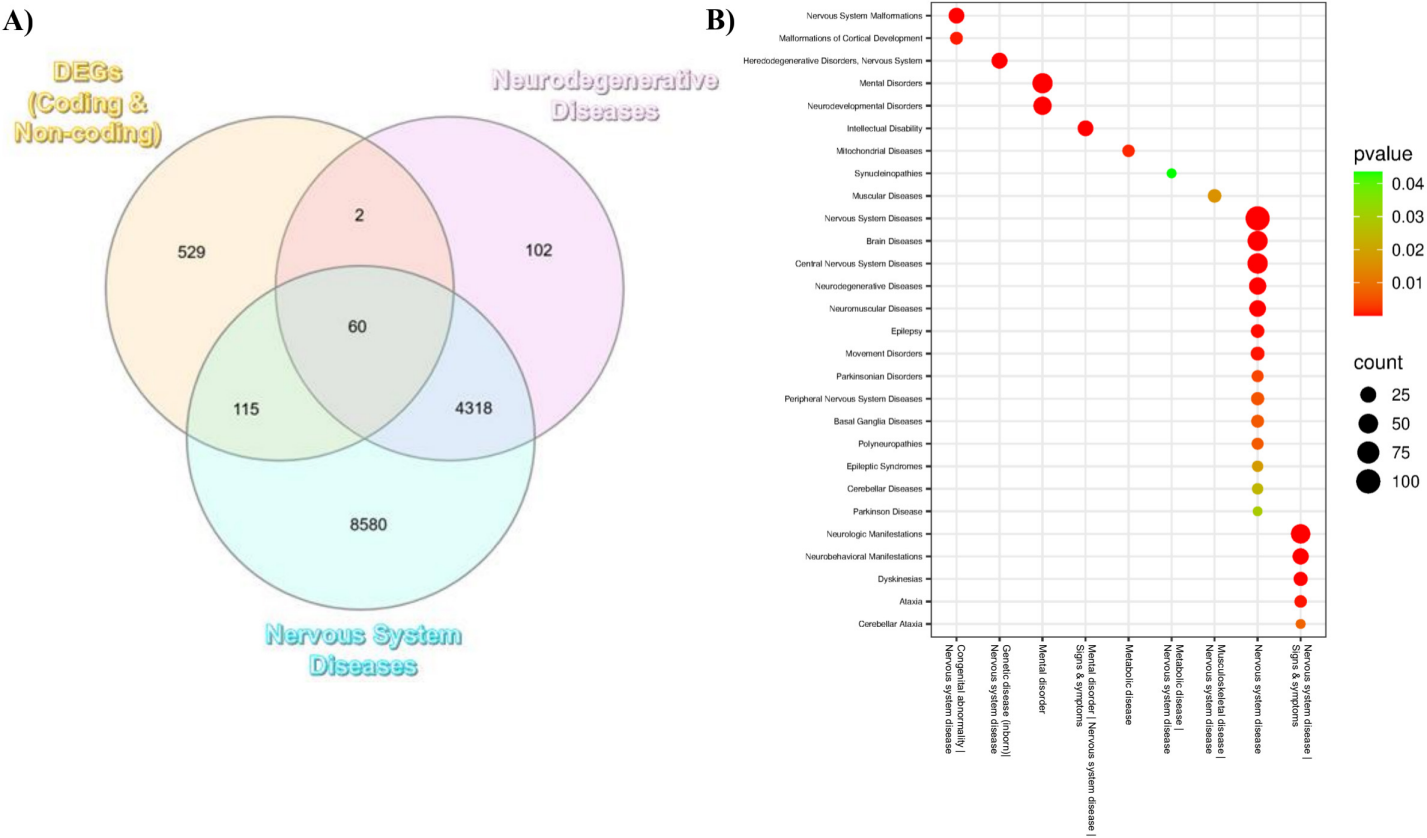
Associations between the differentially expressed genes (DEGs) by HGD+Curc and known human neurological diseases. **(A)** Venn diagram depicting the overlap of identified DEGs (mRNAs, miRNAs, lncRNAs, snoRNAs; orange) with genes associated with neurodegenerative diseases (pink) and nervous system diseases (blue) according to the Genome-Wide Association Study (GWAS) catalog. **(B)** Bubbleplot of disease associations according to the Comparative Toxicogenomics Database; neurological disease (y-axis), disease category (x-axis), number of associated DEGs (node size), corrected *p*-value (node color).

Further investigation was conducted into genomic associations between the differentially expressed genes identified in murine hippocampal genome and genes known to be involved in the development of human neurological diseases due to the consistent involvement of identified DEGs with neurological dysfunction via pathway enrichment analysis. According to the Genome-Wide Association Study (GWAS) catalog, coding and non-coding DEGs regulated by HGD+Curc overlapped with modulated genes seen in human nervous system diseases (*n* = 115), neurodegenerative diseases (*n* = 2), or both (*n* = 60) (Figure 9A). Taking a closer look with the Comparative Toxicogenomics Database (CTD), HGD+Curc modulated DEGs were highly associated with general nervous system disease (corrected *p* = 6.28 ×10^−24^) and several subcategories like congenital abnormalities, mental disorders, and diseases of genetic, metabolic, and musculoskeletal nature (Figure 9B and Supplementary Table 11). Notably, the HGD+Curc-related DEGs were further shown to be associated with neurodegenerative (corrected *p* = 2.48 ×10^−9^)and neurodevelopmental (corrected *p* = 5.02 ×10^−9^)diseases. Associations with signs and symptoms of nervous system diseases such as intellectual disability, neurologic/neurobehavioral manifestations, dyskinesias, and ataxia were also observed.

#### 3.3.8 Curcumin counteracts HGD-induced differential gene expression

**FIGURE 10:**
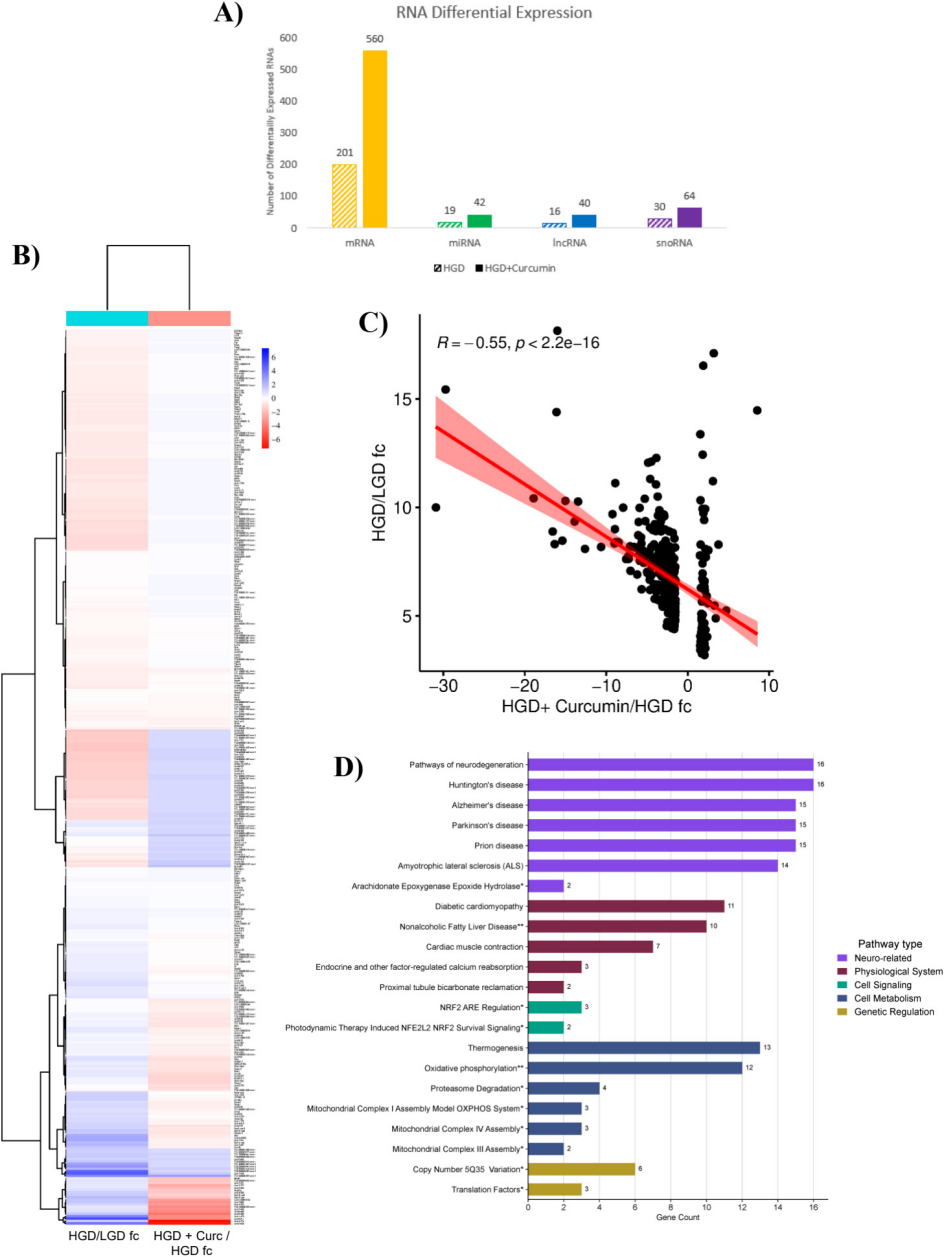
Comparison of the nutrigenomic regulation of HGD + Curc and HGD interventions on murine hippocampal microvasculature. **(A)** Bar graph representing the number of differentially expressed coding (mRNA, orange) and non-coding (miRNAs, green; lncRNAs, blue; snoRNAs, purple) genes that were up-/down-regulated by the HGD intervention compared to LGD (dashed bars) or HGD + Curc versus HGD (solid bars). **(B)** Heatmap of common genes that were differentially regulated in both HGD/LGD (left column) and HGD+Curc/HGD (right column) comparisons; unidentified genes are represented by their Affymetrix ID number. **(C)** Spearman’s correlation plot representing the relationship for the expression of common genes between HGD/LGD and HGD+Curc/HGD fold changes (fc). **(D)** Pathway histogram of common identified genes, organized by gene count and pathway type; statistically regulated pathways (*p* < 0.05) were identified using the Enrichr online database tool- KEGG (no asterisk), Wiki Pathways*, common to both databases**.

The overall goal of this study was to determine the differential genomic regulation of the murine brain microvasculature across the dietary intervention groups, of which the two key comparisons were between HGD versus LGD and HGD+Curc versus HGD. The HGD+Curc supplemented group, relative to HGD as seen in Figure 10A, differentially expressed more than double the number of identified protein coding (560 vs. 201) and non-coding genes (146 vs. 65) in comparison to the DEGs identified between the two control groups of HGD and LGD. There were 376 common DEGs across the two key comparisons, of which were 139 coding mRNAs, 5 miRNAs, 4 lncRNAs, and 14 snoRNAs while the rest were miscellaneous or unidentified genes (Figure 10B and Supplementary Table 12). All of these common DEGs had an upregulated fold change (3.2–18.14) by HGD relative to LGD while 307 of them had reversed expression to downregulation (−30.91 to −1.5), leaving 69 upregulated (1.5 to 8.53) by HGD+Curc compared to HGD. Furthermore, correlation analysis calculated a significant negative correlation (*p* < 2.2 ×10^−16^; *R* = −0.55) between the fold changes of common DEGs across the two key comparisons of HGD/LGD and HGD+Curc/HGD (Figure 10C), suggesting a counteractive effect by curcumin supplementation. Pathway enrichment (Figure 10D) demonstrated that these common DEGs were involved with neurodegenerative diseases (e.g., Huntington’s, Parkinson’s, and Alzheimer’s diseases; prion disease; ALS), diabetic complications (NAFLD and cardiomyopathy) and cellular metabolism (e.g., mitochondrial complex assembly, oxidative phosphorylation, and thermogenesis).

## 4 Discussion

The goal of this study was to assess the nutrigenomic regulation of curcumin on hippocampal microvessels in mice fed on a high glycemic diet. Genes with reversed fold change expression induced HGD+Curc relative to HGD were associated with multiple neurodegenerative diseases (e.g., Alzheimer’s, Huntington’s, Parkinson’s, and prion diseases; ALS), diabetic complications (NAFLD and cardiomyopathy) in addition to the cellular metabolic processes of oxidative phosphorylation and thermogenesis. Consistently shown in this study, these HGD+Curc-modulated pathways were connected via genes regulating the assembly of mitochondrial complexes I (*Ndufa13, Ndufb6, Ndufb7*), II (*Sdhb*), III (*Cox7a2l*, *Uqcr11, Uqcrc1*), and IV (*Cox5a, Cox5b, Cox6a1*) which indicated a switch, from promotion by HGD to reduction by HGD + Curc, in detrimental reactive oxygen species (ROS) production. Overall, this current study offers some of the first evidence that curcumin has potential multigenomic neuroprotective effects on hippocampal microvasculature under the exposure to a high glycemic diet in a wild-type murine model.

### 4.1 HGD/LGD: implications of high glycemic diet consumption and neurodegeneration

In this study following 12 weeks of intervention, HGD induced multi-level genomic changes in the hippocampal microvasculature independently from significant differences in body weight and levels of total cholesterol, HDL-C, LDL-C, triglycerides, insulin, and glucose compared to LGD, which is largely consistent with our previously published study in female mice (20). Under these circumstances, this high glycemic load likely triggered early, tissue-specific cerebrovascular changes that either preceded or occurred independently of systemic alterations in insulin, glucose, and other biochemical markers. Coding genes were primarily upregulated by HGD, mostly for neurodegenerative diseases such as Alzheimer’s, Parkinson’s, Huntington’s, and ALS in addition to cellular metabolic processes of oxidative phosphorylation and thermogenesis and other diabetic issues like cardiomyopathy and NAFLD. The onset of hyperglycemic complications, such as NAFLD, have been closely linked to cognitive impairment, mainly due to the chronic-low grade inflammatory environment as neuroinflammation can be induced via chemokine infiltration across the BBB and activation of microglia (79). Furthermore, development of atherosclerosis and cardiomyopathy can lead to microvascular dysfunction and hippocampal impairment due to decreased cerebral blood flow (80, 81). In line with the reported literature regarding neurological complications brought on by a high-glycemic dietary challenge (10), this study showed that these pathways were linked through genes involved in the assembly of mitochondrial complexes I (*Ndufa13, Ndufb6, Ndufb7, Ndufc1*), II (*Sdhb*), III (*Cox7a2l*, *Uqcr11, Uqcrc1*), and IV (*Cox5a, Cox5b, Cox6a1*). The upregulation of these mitochondrial complex genes induced by HGD suggests possible accelerated production of ROS via oxidative phosphorylation, though not directly studied here, that has been linked to neurodegeneration such as aberrant oxidative damage, protein aggregation (i.e., protein folding), and promotion of neuroinflammatory and apoptotic pathways (82).

Meanwhile non-coding (miRNAs, lncRNAs, and snoRNAs) were primarily downregulated by HGD compared to LGD. Targets genes of HGD-regulated miRNAs were involved in the interplay between kinase phosphorylation (JAK/STAT, MAPK, PI3K-Akt) pathways responsible for influencing the neuroinflammatory and oxidative stress environment of neurological diseases (83). More specifically, JAK/STAT and MAPK signaling has been linked to abnormal accumulation of tau-protein and amyloid-beta (Aβ) characteristic of Alzheimer’s disease (83, 84) while disrupted PI3K/Akt activity impairs neuroplasticity (83, 85). Targets of DE miRNAs were also associated with extracellular matrix (ECM) maintenance like ECM-receptor interactions and cytoskeletal focal adhesion, mainly linked through targeting of collagen subunits. These ECM-interactions, including the perineuronal nets (PNNs) of the central nervous system, are disrupted by increased degradation activity via matrix metalloproteinases (MMPs) (86, 87) and binding of the cell-to-ECM connective integrins to Aβ (86, 87) in contribution to neurodegenerative conditions as these interactions are crucial for long-term potentiation (LTP) in hippocampal neurons (86, 88). Furthermore, all DE lncRNA were downregulated whose gene targets were associated with neuroprotection and neural crest differentiation mediated through NO-cGMP-PKG and Wnt signaling pathways as well as glutamatergic synapse regulation. NO-cGMP-PKG signaling is essential for LTP as NO aids in regulation of neuroplasticity, memory, and hypothalamic responsibilities, but Aβ accumulation impedes NO production and cGMP/PKG downstream activity (89) while Wnt signaling can protect against mitochondrial dysfunction (90). Finally, the structurally modifying ribosomal 2’-O-methylation activity of C/D box snoRNAs has been linked to CNS disorders and neurodegeneration (91), though the *Snord82* downregulated by HGD in this study has not yet been directly implicated. Altogether, the neurodegenerative effect in the hippocampal microvasculature by HGD was once again seen in this study, irrespective of whole-body diabetic markers.

### 4.2 HGD + Curc/HGD: mechanisms of potential nutraceutical protection of hippocampal endothelium

**FIGURE 11:**
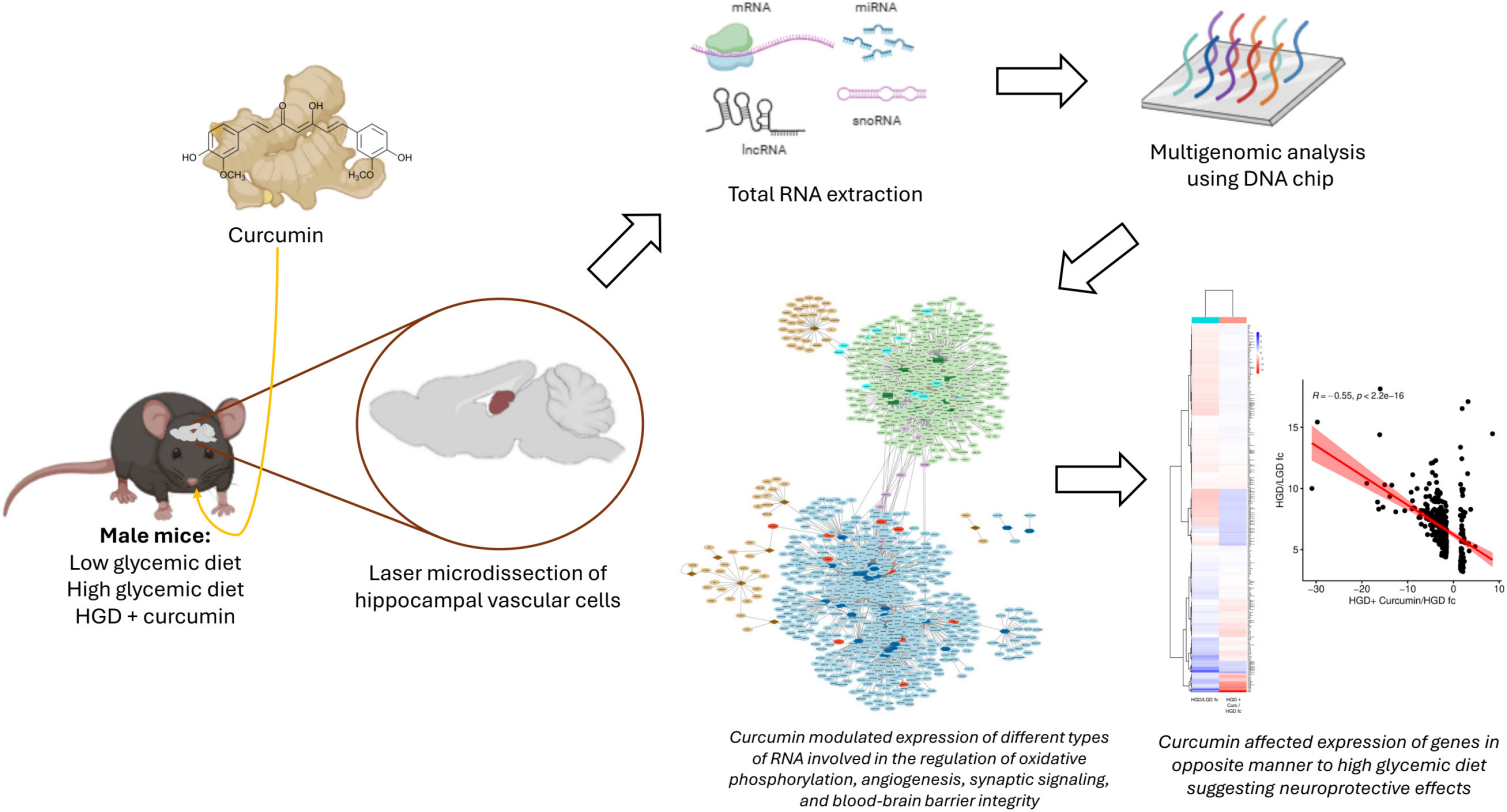
Mechanisms of dietary curcumin regulation in hippocampal microvessels exposed to a high glycemic load.

Curcumin has gained traction in studies of its dietary bioactivity for neuroprotection in the central nervous system due to increasing evidence that it can act as a genomic and epigenetic modulator in multiple diseases like cancer, diabetes, and even neurodegenerative diseases (48, 92–96). Several studies have revealed that curcumin can impact large number of genes simultaneously, such as endothelial cells *in vitro* (97) or within the aorta of ApoE-/- mice (98), however, the multigenomic impact remains largely unknown. These epigenetic mechanisms by curcumin have included regulation of DNA methylation, histone modifications, and expression of non-coding RNAs like microRNAs, lncRNAs, and circular RNAs (92–96, 99–101). While the influence of curcumin has been documented in the brain within models of neurodegeneration (92, 100, 101) and diabetes (42, 92, 102, 103), even specifically in the hippocampus (104–106), they have focused on the temporal and/or hippocampal region as a whole and overall cognitive function. Therefore, these studies have not addressed the effect of curcumin on hippocampal microvessels, especially regarding epigenomic regulation. Notably, the differential expression of snoRNAs by curcumin in the cerebral endothelium has not been previously documented, thus this study highlights a potential new non-coding RNA-related level of epigenomic neuroprotection. Altogether, this current study emphasized the nutrigenomic influence of curcumin on hippocampal endothelial microvasculature subjected to a high glycemic dietary load within a wild-type model through multi-level modulation of coding mRNAs, putative transcription factors, and non-coding RNAs as summarized in Figure 11.

The first indication of the curcumin’s bioactivity in this study was depicted in the circulation as HGD+Curc raised HDL-C and lowered LDL-C level compared to HGD, suggesting a mild corrective effect on dyslipidemia, though there was no overall effect on body weight, TC, and TG. Also, HGD+Curc significantly elevated circulatory insulin compared to HGD, which indicated an insulinotropic (i.e., promotion of insulin secretion) response as curcumin has been reportedly capable of protecting pancreatic β–cell damage under hyperglycemic conditions and diabetic patients (107–110). Thus, the curcumin-induced elevation in serum insulin was likely due to preservation of pancreatic β-cell production/secretory capacity of insulin and reduction of lipotoxic stress on β-cells via improved lipid homeostasis. However, more direct measurements of pancreatic β-cell health, such as the homeostatic model assessment for β-cell function (HOMA-β), need to be performed to further investigate the impact of curcumin consumption on circulatory insulin under the conditions of this study.

The HGD+Curc-induced regulatory pathways of coding genes were predominately linked through the downregulation of oxidative phosphorylation with implications for curcumin-mediated inhibition excessive mitochondrial ROS formation. Structural characteristics of curcumin can directly quench reactive free radicals as the phenolic hydroxyl groups can act as hydrogen or electron donors to form phenoxyl radicals and the keto-enol moiety can promote antioxidant activity via chelation with redox-active metal ions (i.e., *Cu*^2 +^, *Zn*^2 +^, and *Fe*^3 +^) (111, 112). Furthermore, curcumin can indirectly affect ROS levels by promoting the activity of endogenous antioxidant enzymes like superoxide dismutase (SOD), catalase (CAT), and glutathione peroxidase (GPx) via Nrf2 (111); these targets were not assessed in this study but would be important work for future investigations.

Differentially expressed genes related to distinct neurodegenerative diseases in this study leaned toward Alzheimer’s related neurofibril aggregation as HGD+Curc downregulated Aβ precursor protein (*App*), Aβ precursor-like proteins (*Aplp1* and *Aplp2*) and Aβ precursor protein-binding, family B, member 1 (*Apbb1*). Interruption of Aβ aggregation was potentially facilitated by the decreased expression of Rho-associated coiled-coil containing protein kinase 1 (*Rock1*) by HGD+Curc and subsequent phosphorylation of *App*. A more direct inhibition of amyloidogenesis by curcumin was also possibly achieved here as the keto–enol ring and aromatic hydroxyl groups of curcumin reportedly can react covalently with the aromatic rings or form hydrogen bonds with amino acid residues with polar pockets of Aβ, respectively (113). Furthermore, tau-phosphorylation was potentially inhibited due to the decreased expression of calcium/calmodulin-dependent protein kinase IV (*Camk4*), glycogen synthase kinase 3 alpha (*Gsk3a*) as well as the increased expression of *miR-142a-5p* that targeted cyclin-dependent kinase 5 (*Cdk5)*; while the CNS-associated *Gsk3*β isoform (114) was not identified by as a coding DEG, it was targeted by *Gm20675*. *Gsk3* activity is also tied to insulin signaling in a negative-feedback loop as *Gsk3-*overexpression diminishes insulin-mediated glycogen synthesis and glucose homeostasis via pancreatic β-cell dysfunction, leading to peripheral insulin resistance, while insulin-receptor binding inhibits *Gsk3* activation (115). Thus, this downregulation of *Gsk3a* and indirect action on *Gsk3*β may be tied to the potential insulinotropic capability of curcumin in this study. Targeting of caspase signaling, which has been linked to the neuronal apoptosis aspect of dementia (116, 117), was also observed in this study as *Casp3* and *Casp9* were targeted by lncRNA *Gm20675* and *miR-142a-5p/-199a-3p*, respectively. Along with mitochondrial dysfunction, ALS pathology has been mainly linked to aggregation of misfolded SOD1 and transactive response DNA binding protein-43 (TDP-43, encoded by *Tardbp*) (118, 119), which were both downregulated by HGD+Curc.

Regulation of multiple pathways by HGD+Curc in this study indicated potential BBB preservation. Pathological angiogenesis (i.e., formation of new blood vessels), or neovascularization in the context of microvascular networks, has been implicated with the neurodegenerative disease progression and is heavily regulated by VEGF/VEGFR interactions and downstream signaling (120–122). Binding of VEGF to its receptor VEGFR can activate PI3K and Rho GTPases like Ras homolog gene family, member A (*Rhoa*) and cell division cycle 42 (*Cdc42*), which can stimulate actin cytoskeleton/ECM-remodeling in the endothelium by *Rock1* and cofilin-1 (*Cfl1*) (123, 124). This angiogenic process appears to be partially inhibited as genes involved in PI3K signaling (*Pik3ca, Pik3r3, Pik3c2a*), Rho GTPases (*Rhoa, Cdc42/Cdc42ep1*), and cytoskeleton migration (*Rock1, Cfl1)* were downregulated by HGD+Curc. As mentioned previously, permeability of the vascular endothelium/BBB can also be affected by ECM-interactions such as focal adhesion degradation by MMPs and transendothelial migration of leukocytes facilitated by adhesion molecules (86, 125). The miRNA-mediated targeting of *Mmp11* (*let-7a-5p, let-7k, miR-98-5p*, *miR-6394*) and *Mmp24* (*miR-142a-5p, -199a-3p, -665-3p*) along with reduction of neural cell adhesion molecule 1 (*Ncam1*) and aforementioned *Rhoa, Rock1*, and PI3K players by HGD+Curc appear to moderately affect these interactions. Angiogenesis can also be influenced by hypoxia, seen in cases like ischemic stroke (126) and is facilitated by hypoxia-inducible factor-1 (HIF-1) and VEGF/PI3K signaling that all can contribute to a neuroinflammatory environment (125, 127). Hyperglycemia has been linked to upregulation of HIF-1 and loss of endothelial tight junctions in brain microvascular endothelium due to VEGF-overexpression (128). The HIF-1 complex (heterodimer composed of α and β subunits) can be stabilized by heat shock protein 90 (*Hsp90ab1, Hsp90b*) (129), which were reduced by HGD+Curc. Hypoxic signaling can also contribute to Aβ formation and tau hyperphosphorylation (130). Taken together, observed changes in the expression of genes are suggestive of a decrease in BBB permeability, a key factor in neurodegenerative disease development.

Downregulation of these VEGF-mediated angiogenic and BBB-remodeling pathways by HGD+Curc may also indicate a moderate anti-cancer functionality of curcumin by restricting blood flow to tumor cells and reducing cell migration via ECM-degradation related to metastasis (131, 132). PI3k signaling governs cell cycle progression/proliferation, adherence and migration (133, 134) while blunting the cell cycle arrest and apoptotic activities of forkhead box O (FoxO) signaling (134). Therefore, downregulation of players in PI3K pathway (*Pik3ca, Pik3r3, Pik3c2a*) and cell cycle mediators like cyclin D2 (*Ccnd2*, G1 to S), cyclin B1 (*Ccnb1*, G2 to M), and Ras homolog enriched in brain (*Rheb*) by HGD+Curc may indicate an inhibitory effect on the HGD-induced influence toward tumor cell proliferation. Reduction of genes related to glucose transporter type 1 (*Glut1*, encoded by *Slc2a1)*, ATP synthase (*Atp5a1, Atp5b, Atp5g2, Atp5g3*), sodium/potassium (*Na*^+^/*K*^+^) ATPase (*Atp1a1, Atp1a2, Atp1a3, Atp1b1, Atp1b2*), and the vacuolar ATPase (*Atp6v0a1, Atp6v0b, Atp6v0c, Atp6v0e2, Atp6v1c1, Atp6v1d*) as well as non-coding targeting of ATP-binding cassette (ABC) subunits (*Abcc3, Abcc5, Abcc10)* by curcumin in this study may further indicate anti-cancer capabilities due to inhibition by limiting the energy needs of tumorous cells (135–138), though further investigation is warranted.

Curcumin and its derivative demethoxycurcumin as well as related phase I and II metabolites (dihydrocurcumin, hexahydrocurcumin, tetrahydrocurcumin, curcumin glucuronide, and curcumin sulfate) had significant binding interactions with one or more TFs potentially involved in regulation of coding DEGs identified in this study. The largest number of coding DEGs modulated by HGD+Curc interacted with the cAMP-responsive element binding protein (CREB) transcription factor, which has widely reported to be involved with neuroprotection and neuroplasticity (139). Inhibitory phosphorylation of CREB (serine-129) (140) by *Gsk3*β can lead to decreased hippocampal neurogenesis and activity of pro-survival genes like brain-derived neurotrophic factor (BDNF) (141, 142). On the other hand, CREB can be activated by PI3K/Akt at a different site (serine-133) (143, 144) and bind to the promoter region of VEGF (145) and thus is related to tumorigenic vascularization and proliferation. Notably, TFAP2A had the lowest, most significant binding energies with curcumin and related metabolites, except for tetrahydrocurcumin. TFAP2A has a dual role as it is involved in differentiation of cranial neural crest cells and inhibition of this TF can lead to the development of facial clefts, specifically branchio-oculo-facial syndrome (146–148), though it can also influence ECM remodeling via MMPs and promote angiogenesis via VEGF and HIF-1α in oncogenic conditions (149). In another study, curcumin inhibited the oncogenic TFAP2A-induced ECM remodeling in colorectal cancer via downregulation of genes in the ECM-receptor pathway (150). All curcumin-related compounds had significant binding efficacy with nuclear respiratory factor-1 (NRF1), not to be confused with nuclear factor erythroid 2-related factor 1 (Nrf1, encoded by *Nfe2l1*). NRF1 activity reportedly mediates mitochondrial biogenesis and alleviates Aβ-induced degenerative mitochondrial dysfunction (151), which reiterates the potential influence curcumin had on mitochondrial function in the hippocampal microvasculature under high glycemic exposure.

SP1 is another transcription factor that has been seen as a driver of angiogenesis in microvascular endothelial cells under hyperglycemic (152) or hypoxic conditions (153) while another SP family member (SP3) has been associated with regulation of BBB players like transferrin receptor and occludin (152). Though SP1 may be related to neuronal survival and synaptogenesis (154), it is also involved in Alzheimer’s disease through binding to promoter regions of *App* and its cleaving enzyme β-secretase (155). Histone deacetylases (HDACs) are responsible for chromatin compacting that represses gene transcription thus HDAC3, which is the predominantly expressed class I HDAC in the brain and highly expressed in the hippocampus, is involved in the silencing of genes essential for neuronal survival and plasticity (156, 157). Though HDAC3 can be involved in normal brain development by regulating neural progenitor cells, it has been implicated in the progression of neurodegeneration and neurotoxicity largely due to phosphorylation by *Gsk3*β and interactions with HDAC1 (156, 157). HDAC3 can be overexpressed within the hippocampus in diabetic conditions (158), which can promote BBB transendothelial permeability. As curcumin has been reportedly can inhibit activity of multiple classes of HDACs (159), HGD+Curc dietary intervention may have facilitated neuroprotective functionality through the inhibition of HDAC3. TCF12, is a member of the basic helix-loop-helix (bHLH) protein family that has been linked to the promotion of neurogenesis, primarily mesodiencephalic dopaminergic neurons, and heterodimerization with other bHLHs can promote neuronal differentiation during cortical development (160, 161). In an endogenous antioxidant mechanism that is commonly seen with curcumin bioactivity (162), small MAF proteins form heterodimers with nuclear factor erythroid 2-related factor 1 (Nrf2) in order to bind antioxidant response elements (AREs) in the promoters of target genes (163). Finally, FOXF1 is reportedly involved with embryonic development of gut-derived organs such as the intestine, stomach, liver, gallbladder, and lung (164) and has been linked to microvascular and endothelial health, but this was primarily in the context of lung development (165, 166). These coding DEGs regulated by this transcription factor may be involved in modulation of neurodevelopmental disorders like other members of the forkhead box family (167), but it has not yet been directly linked to neuronal function.

Multiple miRNAs differentially expressed by HGD+Curc compared to HGD have been reportedly involved in endothelial health in terms of BBB integrity, angiogenesis, and vascular inflammation. As stated previously, the *let-7* network node of *mmu-let-7a-5p, -let-7k, and -miR-98-5p* connected to the largest subset of target genes which was notable as several members of the *let-7* family have been linked to regulation of cerebrovascular inflammation and angiogenesis (168). Of these, *miR-let-7a* and *miR-98-5p* have been reported to help preserve BBB integrity via prevention of tight junction loss as well as inhibition of proinflammatory cytokine release and immune cell infiltration, even under the case of hyperglycemic stress with *miR-let-7a* (169). Another study found an anti-angiogenic influence of *miR-let-7a* by targeting the TGFβ pathway, particularly *Tgfb3* (170) which was downregulated by HGD+Curc. An additional group of miRNAs involved in angiogenic regulation is the *miR-181* family, of which *miR-181d-5p* was upregulated by HGD+Curc. Particularly, *miR-181d-5p* has been found to negatively regulate hyperglycemia-induced VEGF-mediated angiogenesis in human retinal microvascular endothelial cells (171) and promote blood-tumor barrier permeability in glioma endothelial cells, which may aid in delivery of chemotherapeutic drugs (172). Retinal neovasularization related to diabetic retinopathy was also targeted by *miR-384-3p*, which inhibited this angiogenic process by targeting hexokinase 2 (173). *MiR-384-3p* activity has also shown relevance in the context of Alzheimer’s disease as it has been reported to target *App* and its cleaving enzyme β-secretase (174). Regarding neuroinflammation, NF-κB signaling in the vascular endothelium has been targeted in other studies by *miR-193b-3p* in a direct manner through promoting NF-κB p65 acetylation and inhibition of HDAC3 (175), a TF identified in this study, in addition to an indirect manner through *miR-199a-3p*-mediated targeting of mTOR signaling that reduced NF-κB p65 phosphorylation and adhesion molecule expression related to leukocyte adherence (176). Several other miRNAs outside of this study have also been associated with neurodegeneration (177) as well as endothelial homeostasis and inflammation (178, 179).

In the scope of this study, pathways for targets of DE miRNAs by HGD+Curc participated in conjunction with coding genes for synaptic signaling while exclusive pathways were involved in endothelial ECM maintenance in terms of biosynthesis of N-glycans and glycoaminoglycans (GAGs). BDNF, a type of neurotrophin synthesized in high concentrations within neuronal cell bodies and glia of the hippocampus, is important for synaptogenesis, synaptic plasticity/LTP, and neurotransmitter release (180). Multiple integrative downstream pathways are modulated by BDNF following cleavage and bondage to tropomyosin receptor kinase B (TrkB, *Ntrk2*) receptors, which can recruit SHC-transforming protein 3 (*Shc3*), that are all involved in neurotransmitter release such as PI3K-Akt/mTOR (neuronal survival) and MAPK/ERK (phosphorylation of synaptic vesicles) (180). Additionally, neuroprotective BDNF activation can inhibit *Gsk3*β through phosphorylation by dedicator of cytokinesis 3 (*Dock3*) (181). These BDNF-related processes were influenced by HGD+Curc through non-coding targeting of *Ntrk2/TrkB* by *Gm16121*, *Shc3* by *miR-142a-5p, -384-3p, -665-3p*, and *Dock3* by *let-7a-5p/let-7k/miR-98-5p, miR-6394, miR-665-3p*. However, BDNF is upregulated in a tumor environment due to its influence of pro-survivability cascades like PI3K/Akt/mTOR and MAPK (182).

GAGs and N-glycans are both essential components of the neurovascular ECM that regulate vascular homeostasis and cellular communication as GAGs primarily form the protective endothelial glycocalyx layer (EGL) (183), while N-glycans are attached to transmembrane proteins (i.e., N-glycosylation) to modulate protein folding, cell signaling, and adhesion (184). GAGs involved in the regulation of the PNNs of central nervous system ECM influenced by HGD+Curc were heparan sulfate and chondroitin sulfate as miRNAs (*let-7a-5p, let-7k, miR-98-5p, -142a-5p, -199a-3p, -6394)* targeted genes related to synthesis like chondroitin sulfate synthase 3 (*Chsy3)* and sulfation enzymes such as N-deacetylase/N-sulfotransferase 2 (*Ndst2)*, heparan sulfate-glucosamine 3-O-sulfotransferase 3A1 (*Hs3st3a1*) and carbohydrate sulfotransferase 3 (*Chst3*) which may indicate an influence of curcumin on the glycocalyx aspect of the BBB. Sulfation of GAGs to generate heparan sulfate and chondroitin sulfate contribute to the protective negative charge of the EGL that helps regulate permeability of charged molecules (185), but dysregulation of sulfation patterns are linked to tumor migration and amyloid aggregation (186, 187). Furthermore, miRNAs (*let-7a-5p, let-7k, miR-98-5p, -384-3p, -142a-5p*) targeted N-glycosylation facilitators like dolichyl-diphosphooligosaccharide protein glycosyltransferase (*Ddost*), phosphomannomutase 2 (*Pmm2*), and dolichyl-phosphate beta-glucosyltransferase (*Alg5*). More to the point of regulating cell signaling of glycoproteins, DE miRNAs (*let-7a-5p/let-7k/miR-98-5p, miR-6394*) targeted β-galactoside alpha-2,6-sialyltransferase 1 (*ST6Gal1*) that influences tumorigenic cell adherence by adding sialic acid to glycoproteins (188). Altogether, targets of miRNA differentially expressed by HGD+Curc portrayed a potential versatile regulation of curcumin on aspects like BBB permeability, ECM-regulation and synaptic signaling.

Pathways unique to the gene targets of lncRNAs differentially expressed by HGD+Curc were predominantly involved in pathways regarding neurodevelopmental disorders, rather than neurodegenerative. Such disorders like autism as well as fragile X and Rett syndromes can be associated with mitochondrial dysfunction (189), but findings from this study highlight a link through glutamatergic synaptic signaling as subunits of N-methyl-D-aspartate (NMDA) receptors (*Grin2A, Grin2B, Grin2D*) were targeted by 6–14 lncRNAs differentially expressed by HGD+Curc. Important for synaptic plasticity and LTP for memory, glutamatergic NMDA receptor interactions can be regulated through the NO/cGMP pathway (190) but can be impaired by MTHFR deficiency, which is linked to increased phosphorylation of hippocampal Aβ precursor protein due to dysfunctional folate metabolism (191). Craniofacial development was also associated with DE lncRNAs through TFAP2A, the TF that significantly interacted with all curcumin-related metabolites, which was targeted by *Gm16084*. Again, lncRNA targets complementarity participated in pathways of neurodegeneration through the inclusion of these NMDA receptor subunits and the aforementioned targeting of *Gsk3*β and *Casp3* by *Gm20675.* Regarding the C/D box snoRNAs, *Snord82* has been reported to be a potential tumor suppressor of prostate cancer (192) was upregulated by our HGD+Curc group, which was a reversal of HGD regulation. Additionally, *Snord16a* is a potential biomarker for colon cancer (193) and *Snord59a* is reported to be a tumor immune infiltration-associated snoRNA (194), which were downregulated and upregulated, respectively, by HGD+Curc. However, further studies for relevancy of these *Snords* in the context of brain cancer would be necessary.

This study showed that curcumin supplementation within a high glycemic diet (HGD+Curc) may have a dual role of moderate neuroprotection and potential anti-tumorigenicity in our model of hippocampal microvasculature, though these often involve regulation of the same pathways in opposing directions. Since consequences of high glycemic diet consumption are complex, regulation of curcumin may have acted in a multi-faceted manner and could be elucidated in direct models of neurodegeneration and brain cancer, which has been reviewed previously (38, 43–45, 195–200). Studies investigating curcumin have included multiple models of aging, Alzheimer’s, Huntington’s Parkinson’s, multiple sclerosis, and ischemic/hemorrhagic stroke (43–45, 195–197). Additionally, these studies have included direct measurements of Aβ aggregation, mitochondrial function, and antioxidant/anti-inflammatory markers as well as cognitive tests (43–45, 195–197). Furthermore, some clinical studies have been conducted to assess the effect curcumin on cognitive function in healthy/non-demented older adults (201) as well as Alzheimer’s and Parkinson’s patients (43, 201). Studies of neuroprotection have also been conducted and included in these reviews that involved curcumin nanoparticles (45) and other curcumin related metabolites mentioned in this study (202) with an emphasis on tetrahydrocurcumin (203), demethoxycurcumin (202, 204), and hexahydrocurcumin (205). Finally, the anti-diabetic effect of turmeric and curcumin have been widely reviewed in multiple models (38, 47, 107).

Some limitations should be addressed as the results displayed are representative of the bioinformatics resources utilized and available at the time of the study and subsequent identification of target genes and pathways may vary with the use of other databases and gene ontology analysis tools. Large amounts of DEGs found in the hippocampal microarrays were either miscellaneous (i.e., pseudogenes, multi-complex, immunoglobulin (Ig) variable chain genes) or unidentified. Some of the DEGs common to both HGD/LGD and HGD+Curc comparisons represented in Figure 10 were unidentified, as indicated by only their Affymetrix IDs, leaving pathway enrichment analysis to be done with the remaining identified genes. It is important to note that conclusions about full regulation of the identified pathways cannot be made definitively as the DEGs and non-coding targets characterized within this study via microarray analysis and bioinformatic tools were not totally comprehensive in their respective pathways. Regarding the overall model, the HGD in this study did not appear to induce a hyperglycemic status systemically as changes in body weight and serum TG, TC/HDL-C/LDL-C, TG, insulin and glucose levels compared to LGD were not observed. Studies with endpoints of hyperglycemia and insulin resistance in mice have utilized a high fat diet alone or combined high-fat, high-sucrose diets for short-term studies (e.g., < 16 weeks) (206–209) or high-sucrose diets alone for extended study periods (e.g., 55 weeks) (210). As the diets in this study were isocaloric (∼3.6–3.7 kcal/g) with similar fat content (12.6–13.0 % kcals), the lack of changes in serum metabolic markers may indicate that the consumption of a high-sucrose diet alone at the starting age of 20 weeks and a duration of 12 weeks without the additional stressor of excess calories/fat were not sufficient to induce systemic hyperglycemia and insulin resistance. Additionally, the study was conducted with only male mice so further experimentation to account for sex differences in the analysis for bioactivity of curcumin alongside a high glycemic diet would be needed. Lastly, no direct measurements of abnormal protein aggregation associated with neurological diseases like Aβ, SOD1, α-synuclein were measured.

## 5 Conclusion

Overall, this study showed that dietary intervention of curcumin induced a multi-level, nutrigenomic effect on the hippocampal microvasculature as differentially expressed coding genes and targets of non-coding miRNAs and lncRNAs were involved in numerous pathways, mainly involving key signaling pathways regulating the interplay between neurodegeneration, mitochondrial dysfunction and thermogenesis, in addition to endothelial remodeling induced by the high glycemic diet challenge.

## Data Availability

The datasets presented in this study can be found in online repositories. The names of the repository/repositories and accession number(s) can be found in the article/Supplementary material.

## Footnotes

1
https://bioinformatics.sdstate.edu/go/

2
https://maayanlab.cloud/Enrichr/

3
https://www.bioinformatics.com.cn/en

4
https://www.swissdock.ch/

5
https://www.chemspider.com/

6
https://pubchem.ncbi.nlm.nih.gov/

7
https://www.uniprot.org/

8
https://www.mirbase.org

9
http://userver.bio.uniroma1.it/apps/mienturnet/

10
http://rtools.cbrc.jp/LncRRIsearch/

11
https://www.interactivenn.net/

12
https://string-db.org/

13
https://cytoscape.org/

14
https://ctdbase.org/

15
https://www.ebi.ac.uk/gwas/

## References

[1] Alzheimers Dementia. 2024 Alzheimer’s disease facts and figures. *Alzheimers Dement.* (2024) 20:3708–821. 10.1002/alz.13809 38689398 PMC11095490

[2] Clark LR Berman SE Rivera-Rivera LA Hoscheidt SM Darst BF Engelman CD Macrovascular and microvascular cerebral blood flow in adults at risk for Alzheimer’s disease. *Alzheimers Dement*. (2017) 7:48–55. 10.1016/j.dadm.2017.01.002 28239641 PMC5318539

[3] Han F. Cerebral microvascular dysfunction and neurodegeneration in dementia. *Stroke Vasc Neurol*. (2019) 4:105–7. 10.1136/svn-2018-000213 31338222 PMC6613876

[4] Wang J Chen Y Chen S Mu Z Chen J. How endothelial cell metabolism shapes blood-brain barrier integrity in neurodegeneration. *Front Mol Neurosci*. (2025) 18:1623321. 10.3389/fnmol.2025.1623321 40636522 PMC12238010

[5] Abbott NJ Patabendige AA Dolman DE Yusof SR Begley DJ. Structure and function of the blood-brain barrier. *Neurobiol Dis*. (2010) 37:13–25. 10.1016/j.nbd.2009.07.030 19664713

[6] Anand KS Dhikav V. Hippocampus in health and disease: an overview. *Ann Indian Acad Neurol*. (2012) 15:239–46. 10.4103/0972-2327.104323 23349586 PMC3548359

[7] Fu Z Wu J Nesil T Li MD Aylor KW Liu Z. Long-term high-fat diet induces hippocampal microvascular insulin resistance and cognitive dysfunction. *Am J Physiol Endocrinol Metab*. (2017) 312:E89–97. 10.1152/ajpendo.00297.2016 27899343 PMC5336564

[8] Puri S Shaheen M Grover B. Nutrition and cognitive health: a life course approach. *Front Public Health*. (2023) 11:1023907. 10.3389/fpubh.2023.1023907 37050953 PMC10083484

[9] Caracciolo B Xu W Collins S Fratiglioni L. Cognitive decline, dietary factors and gut-brain interactions. *Mech Ageing Dev*. (2014) 136-137:59–69. 10.1016/j.mad.2013.11.011 24333791

[10] Chavda V Yadav D Patel S Song M. Effects of a diabetic microenvironment on neurodegeneration: special focus on neurological cells. *Brain Sci*. (2024) 14:284. 10.3390/brainsci14030284 38539672 PMC10969071

[11] Grundy SM Cleeman JI Daniels SR Donato KA Eckel RH Franklin BA Diagnosis and management of the metabolic syndrome: an American Heart Association/National Heart, Lung, and Blood Institute Scientific Statement. *Circulation*. (2005) 112:2735–52. 10.1161/CIRCULATIONAHA.105.169404 16157765

[12] Allen KV Frier BM Strachan MW. The relationship between type 2 diabetes and cognitive dysfunction: longitudinal studies and their methodological limitations. *Eur J Pharmacol*. (2004) 490(1–3):169–75. 10.1016/j.ejphar.2004.02.054 15094083

[13] Alberti KG Eckel RH Grundy SM Zimmet PZ Cleeman JI Donato KA Harmonizing the metabolic syndrome: a joint interim statement of the International Diabetes Federation Task Force on Epidemiology and Prevention; National Heart, Lung, and Blood Institute; American Heart Association; World Heart Federation; International Atherosclerosis Society; and International Association for the Study of Obesity. *Circulation*. (2009) 120:1640–5. 10.1161/CIRCULATIONAHA.109.192644 19805654

[14] Duarte JM. Metabolic alterations associated to brain dysfunction in diabetes. *Aging Dis*. (2015) 6:304–21. 10.14336/AD.2014.1104 26425386 PMC4567214

[15] Buie JJ Watson LS Smith CJ Sims-Robinson C. Obesity-related cognitive impairment: the role of endothelial dysfunction. *Neurobiol Dis*. (2019) 132:104580. 10.1016/j.nbd.2019.104580 31454547 PMC6834913

[16] Nuthikattu S Milenkovic D Rutledge J Villablanca A. The western diet regulates hippocampal microvascular gene expression: an integrated genomic analyses in female mice. *Sci Rep*. (2019) 9:19058. 10.1038/s41598-019-55533-9 31836762 PMC6911042

[17] Luo N Guo Y Peng L Deng F. High-fiber-diet-related metabolites improve neurodegenerative symptoms in patients with obesity with diabetes mellitus by modulating the hippocampal-hypothalamic endocrine axis. *Front Neurol*. (2022) 13:1026904. 10.3389/fneur.2022.1026904 36733447 PMC9888315

[18] Niu Y Chang P Liu T Shen X Zhao H Zhang M Obese mice induced by high-fat diet have differential expression of circular RNAs involved in endoplasmic reticulum stress and neuronal synaptic plasticity of hippocampus leading to obesity-associated cognitive impairment. *Front Mol Neurosci*. (2022) 15:1000482. 10.3389/fnmol.2022.1000482 36263377 PMC9574125

[19] Pinto BA Melo TM Flister KF França LM Kajihara D Tanaka LY Early and sustained exposure to high-sucrose diet triggers hippocampal ER stress in young rats. *Metab Brain Dis*. (2016) 31:917–27. 10.1007/s11011-016-9830-1 27154727

[20] Nuthikattu S Milenkovic D Norman JE Rutledge J Villablanca A. High glycemia and soluble epoxide hydrolase in females: differential multiomics in murine brain microvasculature. *Int J Mol Sci*. (2022) 23:13044. 10.3390/ijms232113044 36361847 PMC9655872

[21] Taylor MK Sullivan DK Morris JK Vidoni, Honea RA Mahnken JD High glycemic diet is related to brain amyloid accumulation over one year in preclinical Alzheimer’s Disease. *Front Nutr*. (2021) 8:741534. 10.3389/fnut.2021.741534 34646853 PMC8502814

[22] Santiago JA Karthikeyan M Lackey M Villavicencio D Potashkin JA. Diabetes: a tipping point in neurodegenerative diseases. *Trends Mol Med*. (2023) 29:1029–44. 10.1016/j.molmed.2023.09.005 37827904 PMC10844978

[23] Baduini IR Castro Vildosola JE Kavehmoghaddam S Kiliç F Nadeem SA Nizama JJ Type 2 diabetes mellitus and neurodegenerative disorders: the mitochondrial connection. *Pharmacol Res*. (2024) 209:107439. 10.1016/j.phrs.2024.107439 39357690

[24] Kim YK Song J. Metabolic imbalance and brain tumors: the interlinking metabolic pathways and therapeutic actions of antidiabetic drugs. *Pharmacol Res*. (2025) 215:107719. 10.1016/j.phrs.2025.107719 40174814

[25] Bao Z Chen K Krepel S Tang P Gong W Zhang M High glucose promotes human glioblastoma cell growth by increasing the expression and function of chemoattractant and growth factor receptors. *Transl Oncol*. (2019) 12:1155–63. 10.1016/j.tranon.2019.04.016 31207546 PMC6580091

[26] Ullah H Dacrema M Buccato DG Fayed MAA De Lellis LF Morone MV A narrative review on plant extracts for metabolic syndrome: efficacy, safety, and technological advances. *Nutrients*. (2025) 17:877. 10.3390/nu17050877 40077747 PMC11901876

[27] Wang L Zhao T Zhu X Jiang Q. Low blood carotenoid status in dementia and mild cognitive impairment: a systematic review and meta-analysis. *BMC Geriatr*. (2023) 23:195. 10.1186/s12877-023-03900-7 36997905 PMC10064563

[28] Yang W Cui K Li X Zhao J Zeng Z Song R Effect of polyphenols on cognitive function: evidence from population-based studies and clinical trials. *J Nutr Health Aging*. (2021) 25:1190–204. 10.1007/s12603-021-1685-4 34866146 PMC12275588

[29] Silveira AC Dias JP Santos VM Oliveira PF Alves MG Rato L The action of polyphenols in diabetes mellitus and Alzheimer’s Disease: a common agent for overlapping pathologies. *Curr Neuropharmacol*. (2019) 17:590–613. 10.2174/1570159X16666180803162059 30081787 PMC6712293

[30] Amalraj A Pius A Gopi S Gopi S. Biological activities of curcuminoids, other biomolecules from turmeric and their derivatives - A review. *J Tradit Complement Med*. (2017) 7:205–33. 10.1016/j.jtcme.2016.05.005 28417091 PMC5388087

[31] Prasad S Gupta SC Tyagi AK Aggarwal BB. Curcumin, a component of golden spice: from bedside to bench and back. *Biotechnol Adv*. (2014) 32:1053–64. 10.1016/j.biotechadv.2014.04.004 24793420

[32] Iweala EJ Uche ME Dike, Etumnu LR Dokunmu TM Oluwapelumi AE Curcuma longa (Turmeric): Ethnomedicinal uses, phytochemistry, pharmacological activities and toxicity profiles—A review. *Pharmacol Res Modern Chin Med.* (2023) 6:100222. 10.1016/j.prmcm.2023.100222

[33] Sharifi-Rad J Rayess YE Rizk AA Sadaka C Zgheib R Zam W Turmeric and its major compound curcumin on health: bioactive effects and safety profiles for food, pharmaceutical, biotechnological and medicinal applications. *Front Pharmacol*. (2020) 11:01021. 10.3389/fphar.2020.01021 33041781 PMC7522354

[34] Anand P Kunnumakkara AB Newman RA Aggarwal BB. Bioavailability of curcumin: problems and promises. *Mol Pharm*. (2007) 4:807–18. 10.1021/mp700113r 17999464

[35] Dei Cas M Ghidoni R. Dietary Curcumin: correlation between Bioavailability and Health Potential. *Nutrients*. (2019) 11:2147. 10.3390/nu11092147 31500361 PMC6770259

[36] Chopra H Dey PS Das D Bhattacharya T Shah M Mubin S Curcumin nanoparticles as promising therapeutic agents for drug targets. *Molecules*. (2021) 26:4998. 10.3390/molecules26164998 34443593 PMC8402133

[37] Stohs SJ Chen O Ray SD Ji J Bucci LR Preuss HG. Highly bioavailable forms of curcumin and promising avenues for curcumin-based research and application: a review. *Molecules*. (2020) 25:1397. 10.3390/molecules25061397 32204372 PMC7144558

[38] Cerullo M Armeli F Mengoni B Menin M Crudeli ML Businaro R. Curcumin modulation of the gut-brain axis for neuroinflammation and metabolic disorders prevention and treatment. *Nutrients*. (2025) 17:1430. 10.3390/nu17091430 40362738 PMC12073396

[39] Niwa T Yokoyama S-I Mochizuki M Osawa T. Curcumin metabolism by human intestinal bacteria in vitro. *J Funct Foods.* (2019) 61:103463. 10.1016/j.jff.2019.103463

[40] Enayati A Soghi A Butler AE Rizzo M Sahebkar A. The effect of curcumin on the gut-brain axis: therapeutic implications. *J Neurogastroenterol Motil*. (2023) 29:409–18. 10.5056/jnm23065 37814431 PMC10577457

[41] Hassaninasab A Hashimoto Y Tomita-Yokotani K Kobayashi M. Discovery of the curcumin metabolic pathway involving a unique enzyme in an intestinal microorganism. *Proc Natl Acad Sci U S A*. (2011) 108:6615–20. 10.1073/pnas.1016217108 21467222 PMC3080977

[42] Zhang DW Fu M Gao SH Liu JL. Curcumin and diabetes: a systematic review. *Evid Based Complement Alternat Med*. (2013) 2013:636053. 10.1155/2013/636053 24348712 PMC3857752

[43] Lehoczki A Fekete M Jarecsny T Zábó V Szappanos Á Csípő T The neuroprotective role of curcumin: from molecular pathways to clinical translation-a narrative review. *Nutrients*. (2025) 17:2884. 10.3390/nu17172884 40944272 PMC12430471

[44] Islam MR Rauf A Akter S Akter H Al-Imran MIK Fakir MNH Neuroprotective potential of curcumin in neurodegenerative diseases: clinical insights into cellular and molecular signaling pathways. *J Biochem Mol Toxicol*. (2025) 39:e70369. 10.1002/jbt.70369 40678834 PMC12326297

[45] Genchi G Lauria G Catalano A Carocci A Sinicropi MS. Neuroprotective effects of curcumin in neurodegenerative diseases. *Foods*. (2024) 13:1774. 10.3390/foods13111774 38891002 PMC11172163

[46] Jafarisavari Z Hasanzadeh E Tayebi L Asadpour S. Curcumin delivery in regenerative medicine for Alzheimer’s Disease. *Regen Eng Transl Med.* (2025). 10.1007/s40883-025-00482-1

[47] Marton LT Pescinini-E-Salzedas LM Camargo MEC Barbalho SM Haber JFDS Sinatora RV The effects of curcumin on diabetes mellitus: a systematic review. *Front Endocrinol*. (2021) 12:669448. 10.3389/fendo.2021.669448 34012421 PMC8126655

[48] Barati S Yadegari A Shahmohammadi M Azami F Tahmasebi F Rouhani MR Curcumin as a promising therapeutic agent for diabetic neuropathy: from molecular mechanisms to functional recovery. *Diabetol Metab Syndr*. (2025) 17:314. 10.1186/s13098-025-01884-5 40764946 PMC12323284

[49] Zheng X Zhu J Haedi AR Zhou M. The effect of curcumin supplementation on glycemic indices in adults: a meta-analysis of meta-analyses. *Prostaglandins Other Lipid Mediat*. (2024) 175:106908. 10.1016/j.prostaglandins.2024.106908 39270815

[50] Kawamori T Lubet R Steele VE Kelloff GJ Kaskey RB Rao CV Chemopreventive effect of curcumin, a naturally occurring anti-inflammatory agent, during the promotion/progression stages of colon cancer. *Cancer Res.* (1999) 59:597–601. 9973206

[51] Asai A Miyazawa T. Dietary curcuminoids prevent high-fat diet-induced lipid accumulation in rat liver and epididymal adipose tissue. *J Nutr*. (2001) 131:2932–5. 10.1093/jn/131.11.2932 11694621

[52] Zeng L Yu G Hao W Yang K Chen H. The efficacy and safety of Curcuma longa extract and curcumin supplements on osteoarthritis: a systematic review and meta-analysis. *Biosci Rep*. (2021) 41:BSR20210817. 10.1042/BSR20210817 34017975 PMC8202067

[53] Chandan S Mohan BP Chandan OC Ahmad R Challa A Tummala H Curcumin use in ulcerative colitis: is it ready for prime time? A systematic review and meta-analysis of clinical trials. *Ann Gastroenterol*. (2020) 33:53–8. 10.20524/aog.2019.0439 31892798 PMC6928475

[54] Aung HH Altman R Nyunt T Kim J Nuthikattu S Budamagunta M Lipotoxic brain microvascular injury is mediated by activating transcription factor 3-dependent inflammatory and oxidative stress pathways. *J Lipid Res*. (2016) 57:955–68. 10.1194/jlr.M061853 27087439 PMC4878181

[55] Ball HJ McParland B Driussi C Hunt NH. Isolating vessels from the mouse brain for gene expression analysis using laser capture microdissection. *Brain Res Brain Res Protoc*. (2002) 9:206–13. 10.1016/s1385-299x(02)00147-2 12113780

[56] Ge SX Jung D Yao R. ShinyGO: a graphical gene-set enrichment tool for animals and plants. *Bioinformatics*. (2019) 36:2628–9. 10.1093/bioinformatics/btz931 31882993 PMC7178415

[57] Chen EY Tan CM Kou Y Duan Q Wang Z Meirelles GV Enrichr: interactive and collaborative HTML5 gene list enrichment analysis tool. *BMC Bioinformatics*. (2013) 14:128. 10.1186/1471-2105-14-128 23586463 PMC3637064

[58] Kuleshov MV Jones MR Rouillard AD Fernandez NF Duan Q Wang Z Enrichr: a comprehensive gene set enrichment analysis web server 2016 update. *Nucleic Acids Res.* (2016) 44:W90–7. 10.1093/nar/gkw377 27141961 PMC4987924

[59] Xie Z Bailey A Kuleshov MV Clarke DJB Evangelista JE Jenkins SL Gene set knowledge discovery with enrichr. *Curr Protoc*. (2021) 1:e90. 10.1002/cpz1.90 33780170 PMC8152575

[60] Kanehisa M Furumichi M Sato Y Kawashima M Ishiguro-Watanabe M. KEGG for taxonomy-based analysis of pathways and genomes. *Nucleic Acids Res.* (2023) 51:D587–92. 10.1093/nar/gkac963 36300620 PMC9825424

[61] Agrawal A Balcı H Hanspers K Coort SL Martens M Slenter DN WikiPathways 2024: next generation pathway database. *Nucleic Acids Res.* (2024) 52:D679–89. 10.1093/nar/gkad960 37941138 PMC10767877

[62] Tang D Chen M Huang X Zhang G Zeng L Zhang G SRplot: a free online platform for data visualization and graphing. *PLoS One*. (2023) 18:e0294236. 10.1371/journal.pone.0294236 37943830 PMC10635526

[63] Han H Cho JW Lee S Yun A Kim H Bae D TRRUST v2: an expanded reference database of human and mouse transcriptional regulatory interactions. *Nucleic Acids Res.* (2018) 46:D380–6. 10.1093/nar/gkx1013 29087512 PMC5753191

[64] Matys V Fricke E Geffers R Gössling E Haubrock M Hehl R TRANSFAC: transcriptional regulation, from patterns to profiles. *Nucleic Acids Res*. (2003) 31:374–8. 10.1093/nar/gkg108 12520026 PMC165555

[65] Bugnon M Röhrig UF Goullieux M Perez MAS Daina A Michielin O SwissDock 2024: major enhancements for small-molecule docking with Attracting Cavities and AutoDock Vina. *Nucleic Acids Res.* (2024) 52:W324–32. 10.1093/nar/gkae300 38686803 PMC11223881

[66] Grosdidier A Zoete V Michielin O. SwissDock, a protein-small molecule docking web service based on EADock DSS. *Nucleic Acids Res.* (2011) 39(Web Server issue):W270–7. 10.1093/nar/gkr366 21624888 PMC3125772

[67] Röhrig UF Goullieux M Bugnon M Zoete V. Attracting cavities 2.0: improving the flexibility and robustness for small-molecule docking. *J Chem Inf Model*. (2023) 63:3925–40. 10.1021/acs.jcim.3c00054 37285197 PMC10305763

[68] Zoete V Schuepbach T Bovigny C Chaskar P Daina A Röhrig UF Attracting cavities for docking. Replacing the rough energy landscape of the protein by a smooth attracting landscape. *J Comput Chem*. (2016) 37:437–47. 10.1002/jcc.24249 26558715 PMC4738475

[69] Pettersen EF Goddard TD Huang CC Couch GS Greenblatt DM Meng EC UCSF Chimera–a visualization system for exploratory research and analysis. *J Comput Chem*. (2004) 25(13):1605–12. 10.1002/jcc.20084 15264254

[70] Kim S Chen J Cheng T Gindulyte A He J He S PubChem 2025 update. *Nucleic Acids Res.* (2025) 53:D1516–25. 10.1093/nar/gkae1059 39558165 PMC11701573

[71] Kozomara A Birgaoanu M Griffiths-Jones S. miRBase: from microRNA sequences to function. *Nucleic Acids Res.* (2018) 47:D155–62. 10.1093/nar/gky1141 30423142 PMC6323917

[72] Licursi V Conte F Fiscon G Paci P. MIENTURNET: an interactive web tool for microRNA-target enrichment and network-based analysis. *BMC Bioinformatics*. (2019) 20:545. 10.1186/s12859-019-3105-x 31684860 PMC6829817

[73] Fukunaga T Iwakiri J Ono Y Hamada M. LncRRIsearch: a Web Server for lncRNA-RNA interaction prediction integrated with tissue-specific expression and subcellular localization data. *Front Genet*. (2019) 10:462. 10.3389/fgene.2019.00462 31191601 PMC6546843

[74] Heberle H Meirelles GV da Silva FR Telles GP Minghim R. InteractiVenn: a web-based tool for the analysis of sets through Venn diagrams. *BMC Bioinformatics*. (2015) 16:169. 10.1186/s12859-015-0611-3 25994840 PMC4455604

[75] Szklarczyk D Nastou K Koutrouli M Kirsch R Mehryary F Hachilif R The STRING database in 2025: protein networks with directionality of regulation. *Nucleic Acids Res.* (2025) 53:D730–7. 10.1093/nar/gkae1113 39558183 PMC11701646

[76] Shannon P Markiel A Ozier O Baliga NS Wang JT Ramage D Cytoscape: a software environment for integrated models of biomolecular interaction networks. *Genome Res*. (2003) 13:2498–504. 10.1101/gr.1239303 14597658 PMC403769

[77] Davis AP Wiegers TC Johnson RJ Sciaky D Wiegers J Mattingly CJ. Comparative Toxicogenomics Database (CTD): update 2023. *Nucleic Acids Res.* (2023) 51:D1257–62. 10.1093/nar/gkac833 36169237 PMC9825590

[78] Cerezo M Sollis E Ji Y Lewis E Abid A Bircan KO The NHGRI-EBI GWAS Catalog: standards for reusability, sustainability and diversity. *Nucleic Acids Res.* (2025) 53:D998–1005. 10.1093/nar/gkae1070 39530240 PMC11701593

[79] D’Mello C Le T Swain MG. Cerebral microglia recruit monocytes into the brain in response to tumor necrosis factoralpha signaling during peripheral organ inflammation. *J Neurosci*. (2009) 29:2089–102. 10.1523/JNEUROSCI.3567-08.2009 19228962 PMC6666330

[80] Miao Y Zhang B Sun X Ma X Fang D Zhang W The presence and severity of NAFLD are associated with cognitive impairment and hippocampal damage. *J Clin Endocrinol Metab*. (2023) 108:3239–49. 10.1210/clinem/dgad352 37310344

[81] Kjærgaard K Mikkelsen ACD Wernberg CW Grønkjær LL Eriksen PL Damholdt MF Cognitive dysfunction in non-alcoholic fatty liver disease-current knowledge, mechanisms and perspectives. *J Clin Med*. (2021) 10:673. 10.3390/jcm10040673 33572481 PMC7916374

[82] Dash UC Bhol NK Swain SK Samal RR Nayak PK Raina V Oxidative stress and inflammation in the pathogenesis of neurological disorders: mechanisms and implications. *Acta Pharm Sin B*. (2025) 15:15–34. 10.1016/j.apsb.2024.10.004 40041912 PMC11873663

[83] Wu X Yang Z Zou J Gao H Shao Z Li C Protein kinases in neurodegenerative diseases: current understandings and implications for drug discovery. *Signal Transduct Target Ther*. (2025) 10:146. 10.1038/s41392-025-02179-x 40328798 PMC12056177

[84] Rusek M Smith J El-Khatib K Aikins K Czuczwar SJ Pluta R. The Role of the JAK/STAT Signaling Pathway in the Pathogenesis of Alzheimer’s Disease: new Potential Treatment Target. *Int J Mol Sci*. (2023) 24:864. 10.3390/ijms24010864 36614305 PMC9821184

[85] Guo N Wang X Xu M Bai J Yu H Le Zhang. PI3K/AKT signaling pathway: molecular mechanisms and therapeutic potential in depression. *Pharmacol Res*. (2024) 206:107300. 10.1016/j.phrs.2024.107300 38992850

[86] Soles A Selimovic A Sbrocco K Ghannoum F Hamel K Moncada EL Extracellular matrix regulation in physiology and in brain disease. *Int J Mol Sci*. (2023) 24:7049. 10.3390/ijms24087049 37108212 PMC10138624

[87] Sun Y Xu S Jiang M Liu X Yang L Bai Z Role of the extracellular matrix in Alzheimer’s Disease. *Front Aging Neurosci*. (2021) 13:707466. 10.3389/fnagi.2021.707466 34512308 PMC8430252

[88] Caltagarone J Jing Z Bowser R. Focal adhesions regulate Abeta signaling and cell death in Alzheimer’s disease. *Biochim Biophys Acta*. (2007) 1772:438–45. 10.1016/j.bbadis.2006.11.007 17215111 PMC1876750

[89] Tropea MR Gulisano W Vacanti V Arancio O Puzzo D Palmeri A. Nitric oxide/cGMP/CREB pathway and amyloid-beta crosstalk: from physiology to Alzheimer’s disease. *Free Radic Biol Med*. (2022) 193(Pt 2):657–68. 10.1016/j.freeradbiomed.2022.11.022 36400326

[90] Arrázola MS Silva-Alvarez C Inestrosa NC. How the Wnt signaling pathway protects from neurodegeneration: the mitochondrial scenario. *Front Cell Neurosci*. (2015) 9:166. 10.3389/fncel.2015.00166 25999816 PMC4419851

[91] Verma AK Roy B Dwivedi Y. Decoding the molecular script of 2’-O-ribomethylation: implications across CNS disorders. *Heliyon*. (2024) 10:e39036. 10.1016/j.heliyon.2024.e39036 39524798 PMC11550049

[92] Jiao H Wang X Zhang D Zhou S Gao F. Curcumin and neuroplasticity: epigenetic mechanisms underlying cognitive enhancement in aging and neurodegenerative disorders. *Front Aging Neurosci*. (2025) 17:1592280. 10.3389/fnagi.2025.1592280 40851668 PMC12367793

[93] Ming T Tao Q Tang S Zhao H Yang H Liu M Curcumin: an epigenetic regulator and its application in cancer. *Biomed Pharmacother*. (2022) 156:113956. 10.1016/j.biopha.2022.113956 36411666

[94] Gowhari Shabgah A Hejri Zarifi S Mazloumi Kiapey SS Ezzatifar F Pahlavani N Soleimani D Curcumin and cancer; are long non-coding RNAs missing link? *Prog Biophys Mol Biol*. (2021) 164:63–71. 10.1016/j.pbiomolbio.2021.04.001 33894206

[95] Rismanchi H Malek Mohammadi M Mafi A Khalilzadeh P Farahani N Mirzaei S The role of curcumin in modulating circular RNAs and long non-coding RNAs in cancer. *Clin Transl Oncol*. (2025) 27:2416–36. 10.1007/s12094-024-03782-0 39623194

[96] McCubrey JA Lertpiriyapong K Steelman LS Abrams SL Yang LV Murata RM Effects of resveratrol, curcumin, berberine and other nutraceuticals on aging, cancer development, cancer stem cells and microRNAs. *Aging*. (2017) 9:1477–536. 10.18632/aging.101250 28611316 PMC5509453

[97] Monfoulet LE Mercier S Bayle D Tamaian R Barber-Chamoux N Morand C Curcumin modulates endothelial permeability and monocyte transendothelial migration by affecting endothelial cell dynamics. *Free Radic Biol Med*. (2017) 112:109–20. 10.1016/j.freeradbiomed.2017.07.019 28739530

[98] Coban D Milenkovic D Chanet A Khallou-Laschet J Sabbe L Palagani A Dietary curcumin inhibits atherosclerosis by affecting the expression of genes involved in leukocyte adhesion and transendothelial migration. *Mol Nutr Food Res*. (2012) 56:1270–81. 10.1002/mnfr.201100818 22753158

[99] Fathima A Ameer SF Kerzabi RI Giordo R Nasrallah GK Zayed H Natural antioxidants as regulators of circular RNA expression and function. *Wiley Interdiscip Rev RNA*. (2025) 16:e70023. 10.1002/wrna.70023 40796179 PMC12343168

[100] Wang X Zhang S Li Y Zhang Y. The regulation of miRNAs using curcumin and other polyphenols during the prevention and treatment of Alzheimer’s disease. *Hum Mol Genet*. (2025) 34:117–27. 10.1093/hmg/ddae154 39561994

[101] Abdul-Rahman T Awuah WA Mikhailova T Kalmanovich J Mehta A Ng JC Antioxidant, anti-inflammatory and epigenetic potential of curcumin in Alzheimer’s disease. *Biofactors*. (2024) 50:693–708. 10.1002/biof.2039 38226733

[102] Kumar PT George N Antony S Paulose CS. Curcumin restores diabetes induced neurochemical changes in the brain stem of Wistar rats. *Eur J Pharmacol*. (2013) 702:323–31. 10.1016/j.ejphar.2013.01.012 23380686

[103] Zheng Y Zhang J Zhao Y Zhang Y Zhang X Guan J Curcumin protects against cognitive impairments in a rat model of chronic cerebral hypoperfusion combined with diabetes mellitus by suppressing neuroinflammation, apoptosis, and pyroptosis. *Int Immunopharmacol*. (2021) 93:107422. 10.1016/j.intimp.2021.107422 33548579

[104] Faheem NM El Askary A. Neuroprotective role of curcumin on the hippocampus against the structural and serological alterations of streptozotocin-induced diabetes in Sprague Dawely rats. *Iran J Basic Med Sci*. (2017) 20:690–9. 10.22038/IJBMS.2017.8839 28868124 PMC5569451

[105] Denizci E Altun G Kaplan S. Morphological evidence for the potential protective effects of curcumin and Garcinia kola against diabetes in the rat hippocampus. *Brain Res*. (2024) 1839:149020. 10.1016/j.brainres.2024.149020 38788929

[106] Sun G Miao Z Ye Y Zhao P Fan L Bao Z Curcumin alleviates neuroinflammation, enhances hippocampal neurogenesis, and improves spatial memory after traumatic brain injury. *Brain Res Bull*. (2020) 162:84–93. 10.1016/j.brainresbull.2020.05.009 32502596

[107] Ghorbani Z Hekmatdoost A Mirmiran P. Anti-hyperglycemic and insulin sensitizer effects of turmeric and its principle constituent curcumin. *Int J Endocrinol Metab*. (2014) 12:e18081. 10.5812/ijem.18081 25745485 PMC4338652

[108] Yaikwawong M Jansarikit L Jirawatnotai S Chuengsamarn S. Curcumin extract improves beta cell functions in obese patients with type 2 diabetes: a randomized controlled trial. *Nutr J*. (2024) 23:119. 10.1186/s12937-024-01022-3 39354480 PMC11445938

[109] Seo KI Choi MS Jung UJ Kim HJ Yeo J Jeon SM Effect of curcumin supplementation on blood glucose, plasma insulin, and glucose homeostasis related enzyme activities in diabetic db/db mice. *Mol Nutr Food Res*. (2008) 52:995–1004. 10.1002/mnfr.200700184 18398869

[110] Aziz MT El-Asmar MF Rezq AM Wassef MA Fouad H Roshdy NK Effects of a novel curcumin derivative on insulin synthesis and secretion in streptozotocin-treated rat pancreatic islets in vitro. *Chin Med*. (2014) 9:3. 10.1186/1749-8546-9-3 24422903 PMC3896850

[111] Avendaño-Briseño KA Escutia-Martínez J Hernández-Cruz EY Pedraza-Chaverri J. Antioxidant effect of curcumin and its impact on mitochondria: evidence from biological models. *J Xenobiot*. (2025) 15:139. 10.3390/jox15050139 40981350 PMC12452413

[112] Borra SK Mahendra J Gurumurthy P Jayamathi, Iqbal SS Mahendra L. Effect of curcumin against oxidation of biomolecules by hydroxyl radicals. *J Clin Diagn Res*. (2014) 8:CC01–5. 10.7860/JCDR/2014/8517.4967 25478334 PMC4253152

[113] den Haan J Morrema THJ Rozemuller AJ Bouwman FH Hoozemans JJM. Different curcumin forms selectively bind fibrillar amyloid beta in post mortem Alzheimer’s disease brains: implications for in-vivo diagnostics. *Acta Neuropathol Commun*. (2018) 6:75. 10.1186/s40478-018-0577-2 30092839 PMC6083624

[114] Lei P Ayton S Bush AI Adlard PA. GSK-3 in Neurodegenerative Diseases. *Int J Alzheimers Dis*. (2011) 2011:189246. 10.4061/2011/189246 21629738 PMC3100544

[115] Rayasam GV Tulasi VK Sodhi R Davis JA Ray A. Glycogen synthase kinase 3: more than a namesake. *Br J Pharmacol*. (2009) 156:885–98. 10.1111/j.1476-5381.2008.00085.x 19366350 PMC2697722

[116] Khan S Ahmad K Alshammari EM Adnan M Baig MH Lohani M Implication of Caspase-3 as a common therapeutic target for multineurodegenerative disorders and its inhibition using nonpeptidyl natural compounds. *Biomed Res Int*. (2015) 2015:379817. 10.1155/2015/379817 26064904 PMC4434175

[117] Espinosa-Oliva AM García-Revilla J Alonso-Bellido IM Burguillos MA. Brainiac Caspases: beyond the Wall of Apoptosis. *Front Cell Neurosci*. (2019) 13:500. 10.3389/fncel.2019.00500 31749689 PMC6848387

[118] Tsekrekou M Giannakou M Papanikolopoulou K Skretas G. Protein aggregation and therapeutic strategies in SOD1- and TDP-43- linked ALS. *Front Mol Biosci*. (2024) 11:1383453. 10.3389/fmolb.2024.1383453 38855322 PMC11157337

[119] Balendra R Sreedharan J Hallegger M Luisier R Lashuel HA Gregory JM Amyotrophic lateral sclerosis caused by TARDBP mutations: from genetics to TDP-43 proteinopathy. *Lancet Neurol*. (2025) 24:456–70. 10.1016/S1474-4422(25)00109-7 40252666 PMC7617675

[120] Yuan Y Sun J Dong Q Cui M. Blood-brain barrier endothelial cells in neurodegenerative diseases: signals from the “barrier”. *Front Neurosci*. (2023) 17:1047778. 10.3389/fnins.2023.1047778 36908787 PMC9998532

[121] Kim S Jung UJ Kim SR. The crucial role of the blood-brain barrier in neurodegenerative diseases: mechanisms of disruption and therapeutic implications. *J Clin Med*. (2025) 14:386. 10.3390/jcm14020386 39860392 PMC11765772

[122] Gao F Zhan Y Wang Q Zhang M Dai L Shen Y. Pathological angiogenesis was associated with cerebrovascular lesion and neurodegeneration in Alzheimer’s disease. *Alzheimers Dement*. (2025) 21:e14521. 10.1002/alz.14521 39777972 PMC11848169

[123] Ma LL Guo LL Luo Y Liu GL Lei Y Jing FY Cdc42 subcellular relocation in response to VEGF/NRP1 engagement is associated with the poor prognosis of colorectal cancer. *Cell Death Dis*. (2020) 11:171. 10.1038/s41419-020-2370-y 32139668 PMC7058620

[124] Bryan BA Dennstedt E Mitchell DC Walshe TE Noma K Loureiro R RhoA/ROCK signaling is essential for multiple aspects of VEGF-mediated angiogenesis. *FASEB J*. (2010) 24:3186–95. 10.1096/fj.09-145102 20400538 PMC2923346

[125] Zhang W Xiao D Mao Q Xia H. Role of neuroinflammation in neurodegeneration development. *Signal Transduct Target Ther*. (2023) 8:267. 10.1038/s41392-023-01486-5 37433768 PMC10336149

[126] Merelli A Rodríguez JCG Folch J Regueiro MR Camins A Lazarowski A. Understanding the role of hypoxia inducible factor during neurodegeneration for new therapeutics opportunities. *Curr Neuropharmacol*. (2018) 16:1484–98. 10.2174/1570159X16666180110130253 29318974 PMC6295932

[127] Zhang Z Yan J Chang Y ShiDu Yan S Shi H. Hypoxia inducible factor-1 as a target for neurodegenerative diseases. *Curr Med Chem*. (2011) 18:4335–43. 10.2174/092986711797200426 21861815 PMC3213300

[128] Yan J Zhang Z Shi H. HIF-1 is involved in high glucose-induced paracellular permeability of brain endothelial cells. *Cell Mol Life Sci*. (2012) 69:115–28. 10.1007/s00018-011-0731-5 21617913 PMC11115066

[129] Katschinski DM Le L Heinrich D Wagner KF Hofer T Schindler SG Heat induction of the unphosphorylated form of hypoxia-inducible factor-1alpha is dependent on heat shock protein-90 activity. *J Biol Chem*. (2002) 277:9262–7. 10.1074/jbc.M110377200 11779866

[130] Lin TK Huang CR Lin KJ Hsieh YH Chen SD Lin YC Potential roles of hypoxia-inducible factor-1 in Alzheimer’s Disease: beneficial or Detrimental? *Antioxidants*. (2024) 13:1378. 10.3390/antiox13111378 39594520 PMC11591038

[131] Ghalehbandi S Yuzugulen J Pranjol MZI Pourgholami MH. The role of VEGF in cancer-induced angiogenesis and research progress of drugs targeting VEGF. *Eur J Pharmacol*. (2023) 949:175586. 10.1016/j.ejphar.2023.175586 36906141

[132] Ahir BK Engelhard HH Lakka SS. Tumor Development and Angiogenesis in Adult Brain Tumor: glioblastoma. *Mol Neurobiol*. (2020) 57:2461–78. 10.1007/s12035-020-01892-8 32152825 PMC7170819

[133] Deng S Leong HC Datta A Gopal V Kumar AP Yap CT. PI3K/AKT signaling tips the balance of cytoskeletal forces for cancer progression. *Cancers*. (2022) 14:1652. 10.3390/cancers14071652 35406424 PMC8997157

[134] Mantamadiotis T. Towards Targeting PI3K-dependent regulation of gene expression in brain cancer. *Cancers*. (2017) 9:60. 10.3390/cancers9060060 28556811 PMC5483879

[135] Wang T Ma F Qian HL. Defueling the cancer: atp synthase as an emerging target in cancer therapy. *Mol Ther Oncolytics*. (2021) 23:82–95. 10.1016/j.omto.2021.08.015 34703878 PMC8517097

[136] Shen Q Pan X Li Y Li J Zhang C Jiang X Lysosomes, curcumin, and anti-tumor effects: how are they linked? *Front Pharmacol*. (2023) 14:1220983. 10.3389/fphar.2023.1220983 37484013 PMC10359997

[137] Mahmmoud YA. Curcumin modulation of Na,K-ATPase: phosphoenzyme accumulation, decreased K+ occlusion, and inhibition of hydrolytic activity. *Br J Pharmacol*. (2005) 145:236–45. 10.1038/sj.bjp.0706185 15753945 PMC1576134

[138] Zhang X Chen Q Wang Y Peng W Cai H. Effects of curcumin on ion channels and transporters. *Front Physiol*. (2014) 5:94. 10.3389/fphys.2014.00094 24653706 PMC3949287

[139] Sakamoto K Karelina K Obrietan K. CREB: a multifaceted regulator of neuronal plasticity and protection. *J Neurochem*. (2011) 116:1–9. 10.1111/j.1471-4159.2010.07080.x 21044077 PMC3575743

[140] DaRocha-Souto B Coma M Pérez-Nievas BG Scotton TC Siao M Sánchez-Ferrer P Activation of glycogen synthase kinase-3 beta mediates β-amyloid induced neuritic damage in Alzheimer’s disease. *Neurobiol Dis*. (2012) 45:425–37. 10.1016/j.nbd.2011.09.002 21945540 PMC3694284

[141] Tang M Shi S Guo Y Xu W Wang L Chen Y GSK-3/CREB pathway involved in the gx-50’s effect on Alzheimer’s disease. *Neuropharmacology*. (2014) 81:256–66. 10.1016/j.neuropharm.2014.02.008 24565641

[142] Amidfar M de Oliveira J Kucharska E Budni J Kim YK. The role of CREB and BDNF in neurobiology and treatment of Alzheimer’s disease. *Life Sci*. (2020) 257:118020. 10.1016/j.lfs.2020.118020 32603820

[143] Hong J Wu Y Li M Man KF Song D Koh SB. Camp response element-binding protein: a credible cancer drug target. *J Pharmacol Exp Ther*. (2025) 392:103529. 10.1016/j.jpet.2025.103529 40157009 PMC12060161

[144] Du K Montminy M. CREB is a regulatory target for the protein kinase Akt/PKB. *J Biol Chem*. (1998) 273:32377–9. 10.1074/jbc.273.49.32377 9829964

[145] Lee HT Chang YC Tu YF Huang CC. CREB activation mediates VEGF-A’s protection of neurons and cerebral vascular endothelial cells. *J Neurochem*. (2010) 113:79–91. 10.1111/j.1471-4159.2010.06584.x 20067582

[146] Zainolabidin N Kamath SP Thanawalla AR Chen AI. Distinct Activities of Tfap2A and Tfap2B in the Specification of GABAergic Interneurons in the Developing Cerebellum. *Front Mol Neurosci*. (2017) 10:281. 10.3389/fnmol.2017.00281 28912684 PMC5583517

[147] Ma M Liao Y Huang X Zou C Chen L Liang L Identification of Alzheimer’s Disease molecular subtypes based on parallel large-scale sequencing. *Front Aging Neurosci*. (2022) 14:770136. 10.3389/fnagi.2022.770136 35592696 PMC9112923

[148] Al-Sabri MH Nikpour M Clemensson LE Attwood MM Williams MJ Rask-Anderson M The regulatory role of AP-2β in monoaminergic neurotransmitter systems: insights on its signalling pathway, linked disorders and theragnostic potential. *Cell Biosci*. (2022) 12:151. 10.1186/s13578-022-00891-7 36076256 PMC9461128

[149] Jin C Luo Y Liang Z Li X Kołat D Zhao L Crucial role of the transcription factors family activator protein 2 in cancer: current clue and views. *J Transl Med*. (2023) 21:371. 10.1186/s12967-023-04189-1 37291585 PMC10249218

[150] Mao X Zhang X Zheng X Chen Y Xuan Z Huang P. Curcumin suppresses LGR5(+) colorectal cancer stem cells by inducing autophagy and via repressing TFAP2A-mediated ECM pathway. *J Nat Med*. (2021) 75:590–601. 10.1007/s11418-021-01505-1 33713277 PMC8159825

[151] Massaro M Baudo G Lee H Liu H Blanco E. Nuclear respiratory factor-1 (NRF1) induction drives mitochondrial biogenesis and attenuates amyloid beta-induced mitochondrial dysfunction and neurotoxicity. *Neurotherapeutics*. (2025) 22:e00513. 10.1016/j.neurot.2024.e00513 39730291 PMC12014405

[152] Xie J Gong Q Liu X Liu Z Tian R Cheng Y Transcription factor SP1 mediates hyperglycemia-induced upregulation of roundabout4 in retinal microvascular endothelial cells. *Gene*. (2017) 616:31–40. 10.1016/j.gene.2017.03.027 28341181

[153] Ai L Lin S Huang C Gao L Zhou J Chen C Simultaneous interference of SP1 and HIF1α retarding the proliferation, migration, and invasion of human microvascular endothelial cells (HMEC-1) under hypoxia. *J Cell Biochem*. (2019) 120:17912–25. 10.1002/jcb.29059 31135072

[154] Hung CY Hsu TI Chuang JY Su TP Chang WC Hung JJ. Sp1 in Astrocyte Is Important for Neurite Outgrowth and Synaptogenesis. *Mol Neurobiol*. (2020) 57:261–77. 10.1007/s12035-019-01694-7 31317491 PMC7269153

[155] Brock B Basha R DiPalma K Anderson A Harry GJ Rice DC Co-localization and distribution of cerebral APP and SP1 and its relationship to amyloidogenesis. *J Alzheimers Dis*. (2008) 13:71–80. 10.3233/jad-2008-13108 18334759 PMC5862394

[156] Rosete C Ciernia AV. The two faces of HDAC3: neuroinflammation in disease and neuroprotection in recovery. *Epigenomics*. (2024) 16:1373–88. 10.1080/17501911.2024.2419357 39513228 PMC11728336

[157] D’Mello SR. Histone deacetylase-3: friend and foe of the brain. *Exp Biol Med*. (2020) 245:1130–41. 10.1177/1535370220928278 32486848 PMC7400723

[158] Zhao Q Zhang F Yu Z Guo S Liu N Jiang Y HDAC3 inhibition prevents blood-brain barrier permeability through Nrf2 activation in type 2 diabetes male mice. *J Neuroinflammation*. (2019) 16:103. 10.1186/s12974-019-1495-3 31101061 PMC6525453

[159] Soflaei SS Momtazi-Borojeni AA Majeed M Derosa G Maffioli P Sahebkar A. Curcumin: a Natural Pan-HDAC Inhibitor in Cancer. *Curr Pharm Des*. (2018) 24:123–9. 10.2174/1381612823666171114165051 29141538

[160] Singh A Mahesh A Noack F Cardoso de Toledo B Calegari F Tiwari VK. Tcf12 and NeuroD1 cooperatively drive neuronal migration during cortical development. *Development*. (2022) 149:dev200250. 10.1242/dev.200250 35147187 PMC8918803

[161] Mesman S Smidt MP. Tcf12 Is involved in early cell-fate determination and subset specification of midbrain dopamine neurons. *Front Mol Neurosci*. (2017) 10:353. 10.3389/fnmol.2017.00353 29163030 PMC5671939

[162] Shahcheraghi SH Salemi F Peirovi N Ayatollahi J Alam W Khan H Nrf2 regulation by curcumin: molecular aspects for therapeutic prospects. *Molecules*. (2021) 27:167. 10.3390/molecules27010167 35011412 PMC8746993

[163] He F Ru X Wen T. NRF2, a transcription factor for stress response and beyond. *Int J Mol Sci*. (2020) 21:4777. 10.3390/ijms21134777 32640524 PMC7369905

[164] Kim IM Zhou Y Ramakrishna S Hughes DE Solway J Costa RH Functional characterization of evolutionarily conserved DNA regions in forkhead box f1 gene locus. *J Biol Chem*. (2005) 280:37908–16. 10.1074/jbc.M506531200 16144835

[165] Ren X Ustiyan V Pradhan A Cai Y Havrilak JA Bolte CS FOXF1 transcription factor is required for formation of embryonic vasculature by regulating VEGF signaling in endothelial cells. *Circ Res*. (2014) 115:709–20. 10.1161/CIRCRESAHA.115.304382 25091710 PMC4810682

[166] Cai Y Bolte C Le T Goda C Xu Y Kalin TV FOXF1 maintains endothelial barrier function and prevents edema after lung injury. *Sci Signal*. (2016) 9:ra40. 10.1126/scisignal.aad1899 27095594

[167] Han L Chen M Wang Y Wu H Quan Y Bai T Pathogenic missense mutation pattern of forkhead box genes in neurodevelopmental disorders. *Mol Genet Genomic Med*. (2019) 7:e00789. 10.1002/mgg3.789 31199603 PMC6625093

[168] Bernstein DL Jiang X Rom S. let-7 microRNAs: their role in cerebral and cardiovascular diseases, inflammation, cancer, and their regulation. *Biomedicines*. (2021) 9:606. 10.3390/biomedicines9060606 34073513 PMC8227213

[169] Song J Yoon SR Kim OY. miR-Let7A controls the cell death and tight junction density of brain endothelial cells under high glucose condition. *Oxid Med Cell Longev*. (2017) 2017:6051874. 10.1155/2017/6051874 28680530 PMC5478855

[170] Wang S Zhou H Wu D Ni H Chen Z Chen C MicroRNA let-7a regulates angiogenesis by targeting TGFBR3 mRNA. *J Cell Mol Med*. (2019) 23:556–67. 10.1111/jcmm.13960 30467960 PMC6307798

[171] Wang F Yu C. In vitro protective effect of miR-181d-5p in high glucose-induced human retinal microvascular endothelial cells by targeting the angiogenic factor VEGFA. *Eur Rev Med Pharmacol Sci*. (2022) 26:6199–207. 10.26355/eurrev_202209_29637 36111920

[172] Guo J Cai H Zheng J Liu X Liu Y Ma J Long non-coding RNA NEAT1 regulates permeability of the blood-tumor barrier via miR-181d-5p-mediated expression changes in ZO-1, occludin, and claudin-5. *Biochim Biophys Acta Mol Basis Dis*. (2017) 1863:2240–54. 10.1016/j.bbadis.2017.02.005 28185956

[173] Xia F Sun JJ Jiang YQ Li CF. MicroRNA-384-3p inhibits retinal neovascularization through targeting hexokinase 2 in mice with diabetic retinopathy. *J Cell Physiol*. (2018) 234:721–30. 10.1002/jcp.26871 30191948

[174] Liu CG Wang JL Li L Wang PC. MicroRNA-384 regulates both amyloid precursor protein and β-secretase expression and is a potential biomarker for Alzheimer’s disease. *Int J Mol Med*. (2014) 34:160–6. 10.3892/ijmm.2014.1780 24827165

[175] Lai N Wu D Liang T Pan P Yuan G Li X Systemic exosomal miR-193b-3p delivery attenuates neuroinflammation in early brain injury after subarachnoid hemorrhage in mice. *J Neuroinflammation*. (2020) 17:74. 10.1186/s12974-020-01745-0 32098619 PMC7041199

[176] Gu X Weng R Hou J Liu S. Endothelial miR-199a-3p regulating cell adhesion molecules by targeting mTOR signaling during inflammation. *Eur J Pharmacol*. (2022) 925:174984. 10.1016/j.ejphar.2022.174984 35489420

[177] Li S Lei Z Sun T. The role of microRNAs in neurodegenerative diseases: a review. *Cell Biol Toxicol*. (2023) 39:53–83. 10.1007/s10565-022-09761-x 36125599 PMC9486770

[178] Fernández-Hernando C Suárez Y. MicroRNAs in endothelial cell homeostasis and vascular disease. *Curr Opin Hematol*. (2018) 25:227–36. 10.1097/MOH.0000000000000424 29547400 PMC6175704

[179] Zhong L Simard MJ Huot J. Endothelial microRNAs regulating the NF-κB pathway and cell adhesion molecules during inflammation. *FASEB J*. (2018) 32:4070–84. 10.1096/fj.201701536R 29565737

[180] Wang CS Kavalali ET Monteggia LM. BDNF signaling in context: from synaptic regulation to psychiatric disorders. *Cell*. (2022) 185:62–76. 10.1016/j.cell.2021.12.003 34963057 PMC8741740

[181] Namekata K Harada C Guo X Kimura A Kittaka D Watanabe H Dock3 stimulates axonal outgrowth via GSK-3β-mediated microtubule assembly. *J Neurosci*. (2012) 32:264–74. 10.1523/JNEUROSCI.4884-11.2012 22219288 PMC6621311

[182] Lei M Liu Q Nie J Huang R Mei Y Pan D Impact and mechanisms of Action of BDNF on neurological disorders, cancer, and cardiovascular diseases. *CNS Neurosci Ther*. (2024) 30:e70138. 10.1111/cns.70138 39648800 PMC11626086

[183] Logsdon AF Rhea EM Reed M Banks WA Erickson MA. The neurovascular extracellular matrix in health and disease. *Exp Biol Med*. (2021) 246:835–44. 10.1177/1535370220977195 33302738 PMC8719034

[184] Esmail S Manolson MF. Advances in understanding N-glycosylation structure, function, and regulation in health and disease. *Eur J Cell Biol*. (2021) 100:151186. 10.1016/j.ejcb.2021.151186 34839178

[185] Walter FR Santa-Maria AR Mészáros M Veszelka S Dér A Deli MA. Surface charge, glycocalyx, and blood-brain barrier function. *Tissue Barriers*. (2021) 9:1904773. 10.1080/21688370.2021.1904773 34003072 PMC8489908

[186] Wang Q Chi L. The alterations and roles of glycosaminoglycans in human diseases. *Polymers*. (2022) 14:5014. 10.3390/polym14225014 36433141 PMC9694910

[187] Wade A Robinson AE Engler JR Petritsch C James CD Phillips JJ. Proteoglycans and their roles in brain cancer. *FEBS J*. (2013) 280:2399–417. 10.1111/febs.12109 23281850 PMC3644380

[188] Gc S Bellis SL Hjelmeland AB. ST6Gal1: oncogenic signaling pathways and targets. *Front Mol Biosci*. (2022) 9:962908. 10.3389/fmolb.2022.962908 36106023 PMC9465715

[189] Anitha A Thanseem I Iype M Thomas SV. Mitochondrial dysfunction in cognitive neurodevelopmental disorders: cause or effect? *Mitochondrion*. (2023) 69:18–32. 10.1016/j.mito.2023.01.002 36621534

[190] Huang CC Chan SH Hsu KS. cGMP/protein kinase G-dependent potentiation of glutamatergic transmission induced by nitric oxide in immature rat rostral ventrolateral medulla neurons in vitro. *Mol Pharmacol*. (2003) 64:521–32. 10.1124/mol.64.2.521 12869658

[191] Hoffman A Taleski G Qian H Wasek B Arning E Bottiglieri T Methylenetetrahydrofolate reductase deficiency deregulates regional brain amyloid-β protein precursor expression and phosphorylation levels. *J Alzheimers Dis*. (2018) 64:223–37. 10.3233/JAD-180032 29865064

[192] Ho SM Cheong A Lam HM Hu WY Shi GB Zhu X Exposure of human prostaspheres to bisphenol a epigenetically regulates SNORD family noncoding RNAs via histone modification. *Endocrinology*. (2015) 156:3984–95. 10.1210/en.2015-1067 26248216 PMC4606748

[193] He JY Liu X Qi ZH Wang Q Lu WQ Zhang QT Small nucleolar RNA, C/D Box 16 (SNORD16) acts as a potential prognostic biomarker in colon cancer. *Dose Response*. (2020) 18:1559325820917829. 10.1177/1559325820917829 32704240 PMC7359415

[194] Cai C Peng Y Shen E Wan R Gao L Gao Y Identification of tumour immune infiltration-associated snoRNAs (TIIsno) for predicting prognosis and immune landscape in patients with colon cancer via a TIIsno score model. *EBioMedicine*. (2022) 76:103866. 10.1016/j.ebiom.2022.103866 35144219 PMC8844792

[195] Hu S Maiti P Ma Q Zuo X Jones MR Cole GM Clinical development of curcumin in neurodegenerative disease. *Expert Rev Neurother*. (2015) 15:629–37. 10.1586/14737175.2015.1044981 26035622 PMC6800094

[196] Benameur T Giacomucci G Panaro MA Ruggiero M Trotta T Monda V New promising therapeutic avenues of curcumin in brain diseases. *Molecules*. (2021) 27:236. 10.3390/molecules27010236 35011468 PMC8746812

[197] Zia A Farkhondeh T Pourbagher-Shahri AM Samarghandian S. The role of curcumin in aging and senescence: molecular mechanisms. *Biomed Pharmacother*. (2021) 134:111119. 10.1016/j.biopha.2020.111119 33360051

[198] Klinger NV Mittal S. Therapeutic potential of curcumin for the treatment of brain tumors. *Oxid Med Cell Longev*. (2016) 2016:9324085. 10.1155/2016/9324085 27807473 PMC5078657

[199] Qi X Jha SK Jha NK Dewanjee S Dey A Deka R Antioxidants in brain tumors: current therapeutic significance and future prospects. *Mol Cancer*. (2022) 21:204. 10.1186/s12943-022-01668-9 36307808 PMC9615186

[200] Garodia P Hegde M Kunnumakkara AB Aggarwal BB. Curcumin, inflammation, and neurological disorders: how are they linked? *Integr Med Res*. (2023) 12:100968. 10.1016/j.imr.2023.100968 37664456 PMC10469086

[201] Francis AJ Sreenivasan C Parikh A AlQassab O Kanthajan T Pandey M Curcumin and cognitive function: a systematic review of the effects of curcumin on adults with and without neurocognitive disorders. *Cureus*. (2024) 16:e67706. 10.7759/cureus.67706 39318960 PMC11421876

[202] Di Meo F Margarucci S Galderisi U Crispi S Peluso G. Curcumin. Gut Microbiota, and Neuroprotection. *Nutrients*. (2019) 11:2426. 10.3390/nu11102426 31614630 PMC6835970

[203] Zeng A Quan Y Tao H Dai Y Song L Zhao J. The role of tetrahydrocurcumin in tumor and neurodegenerative diseases through anti-inflammatory effects. *Int J Mol Sci*. (2025) 26:3561. 10.3390/ijms26083561 40332041 PMC12027286

[204] Ramkumar M Rajasankar S Gobi VV Dhanalakshmi C Manivasagam T Justin Thenmozhi A Neuroprotective effect of Demethoxycurcumin, a natural derivative of Curcumin on rotenone induced neurotoxicity in SH-SY 5Y Neuroblastoma cells. *BMC Complement Altern Med*. (2017) 17:217. 10.1186/s12906-017-1720-5 28420370 PMC5395846

[205] Huang Y Cao S Zhang Q Zhang H Fan Y Qiu F Biological and pharmacological effects of hexahydrocurcumin, a metabolite of curcumin. *Arch Biochem Biophys*. (2018) 646:31–7. 10.1016/j.abb.2018.03.030 29596797

[206] Surwit RS Feinglos MN Rodin J Sutherland A Petro AE Opara EC Differential effects of fat and sucrose on the development of obesity and diabetes in C57BL/6J and A/J mice. *Metabolism*. (1995) 44:645–51. 10.1016/0026-0495(95)90123-x 7752914

[207] Fisher-Wellman KH Ryan TE Smith CD Gilliam LA Lin CT Reese LR A Direct comparison of metabolic responses to high-fat diet in C57BL/6J and C57BL/6NJ Mice. *Diabetes*. (2016) 65:3249–61. 10.2337/db16-0291 27495226 PMC5079634

[208] Turner N Kowalski GM Leslie SJ Risis S Yang C Lee-Young RS Distinct patterns of tissue-specific lipid accumulation during the induction of insulin resistance in mice by high-fat feeding. *Diabetologia*. (2013) 56:1638–48. 10.1007/s00125-013-2913-1 23620060

[209] Baranowski BJ Bott KN MacPherson REK. Evaluation of neuropathological effects of a high-fat high-sucrose diet in middle-aged male C57BL6/J mice. *Physiol Rep*. (2018) 6:e13729. 10.14814/phy2.13729 29890051 PMC5995310

[210] Sumiyoshi M Sakanaka M Kimura Y. Chronic intake of high-fat and high-sucrose diets differentially affects glucose intolerance in mice. *J Nutr*. (2006) 136:582–7. 10.1093/jn/136.3.582 16484528

