# Supplementary material for: Nutrigenomic influence of a curcumin-supplemented high glycemic diet on hippocampal microvasculature in male C57BL/6J mice
Source: Front Nutr. 2026 Feb 3;12:1736964. doi: 10.3389/fnut.2025.1736964 (PMC12909243; doi:10.3389/fnut.2025.1736964)
Supplement: Supplementary file 1 [file Data_Sheet_1.pdf]

Supplementary Material: Nutrigenomic Influence of a Curcumin-Supplemented High Glycemic Diet on Hippocampal Microvasculature in Male C57BL/6J Mice

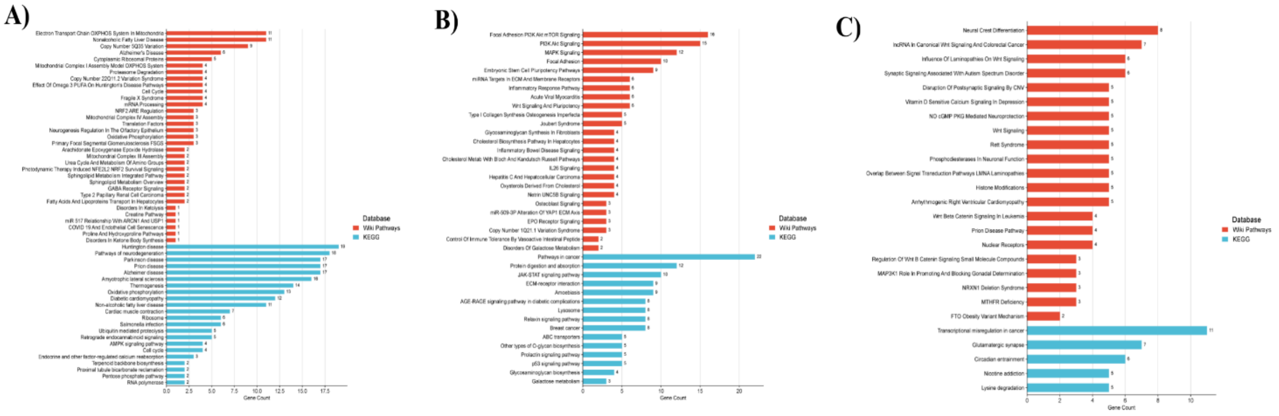

Supplementary Figure 1: HGD coding and non-coding pathways. Histograms of statistically regulated pathways ($p < 0.05$) for **A)** coding mRNAs and **B,C)** gene targets of differentially expressed non-coding **B)** miRNAs and **C)** lncRNAs for the HGD/LGD comparison**;** organized by KEGG or Wiki Pathways databases identified using the Enrichr online database tool.

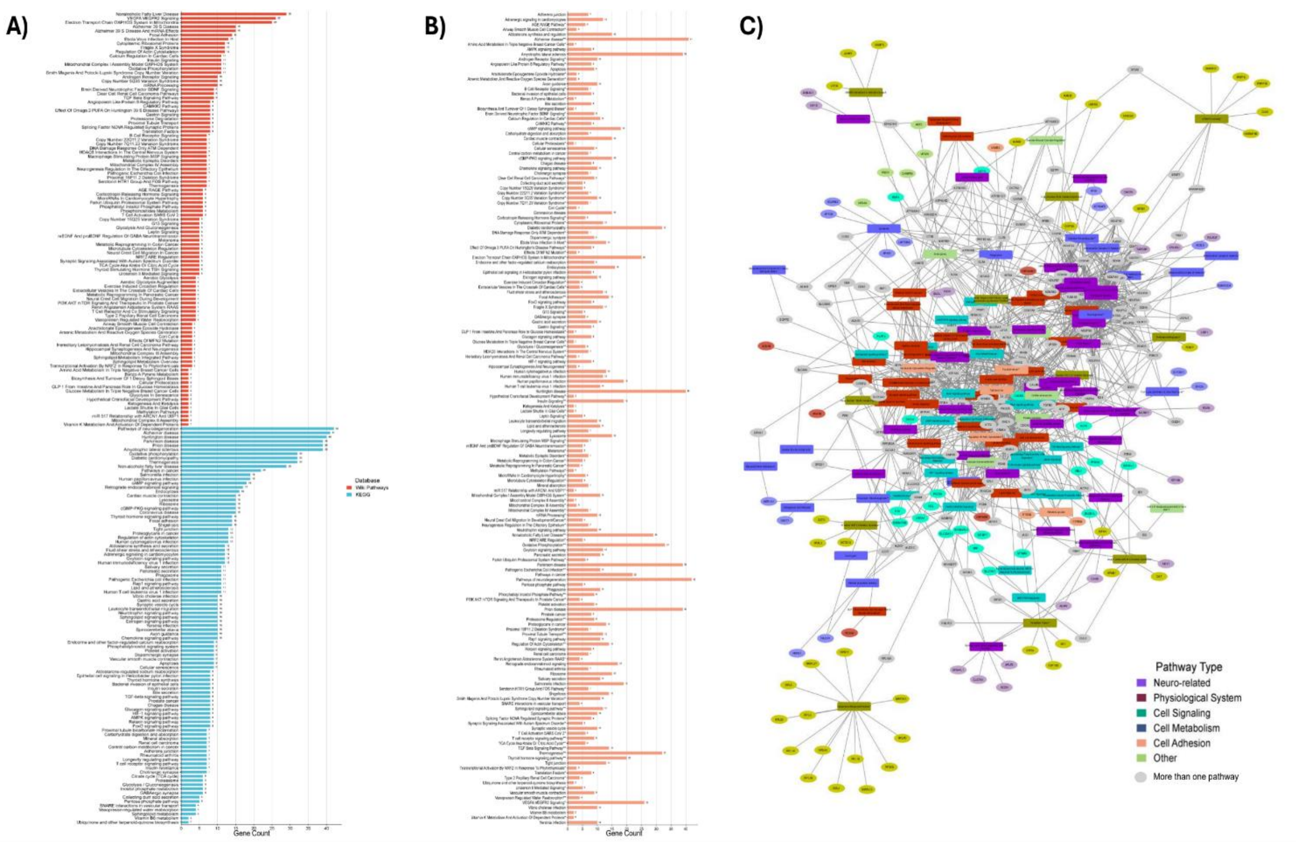

Supplementary Figure 2: HGD + Curcumin coding pathways, alternate organization. Histograms for gene targets of differentially expressed mRNAs, organized by **A)** database or **B)** alphabetically; statistically regulated pathways ($p < 0.05$) were identified using the Enrichr online database tool (KEGG (no asterisk), Wiki Pathways*, common to both**. **C)** Network map of DE coding genes and their respective pathways.

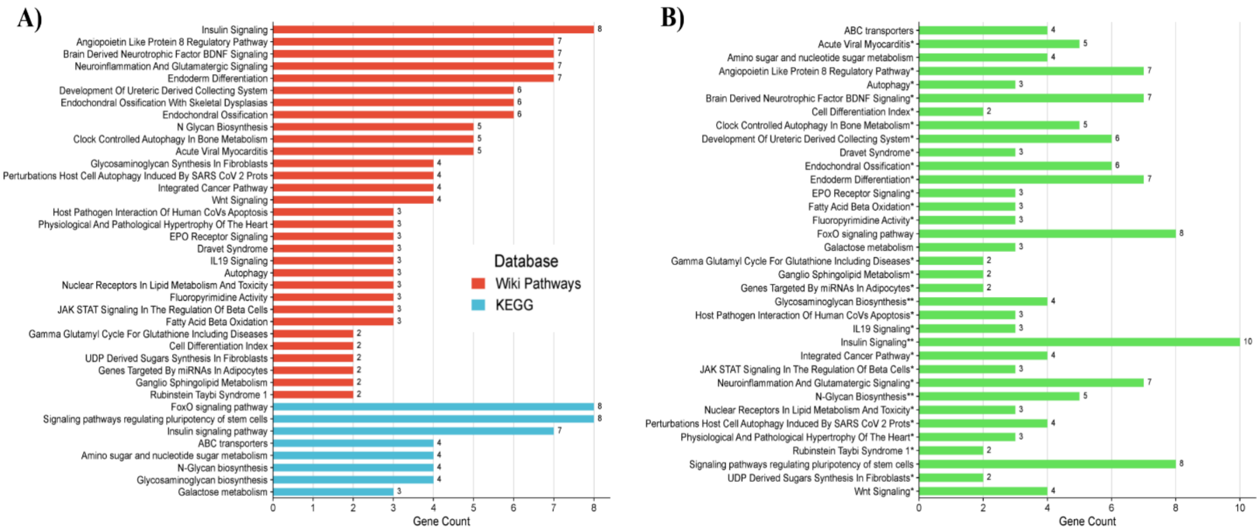

Supplementary Figure 3: HGD + Curcumin miRNA Pathways. Pathway histograms for gene targets of differentially expressed miRNAs, organized by **A)** database or **B)** alphabetically; statistically regulated pathways ($p < 0.05$) were identified using the Enrichr online database tool (KEGG (no asterisk), Wiki Pathways*, common to both**.

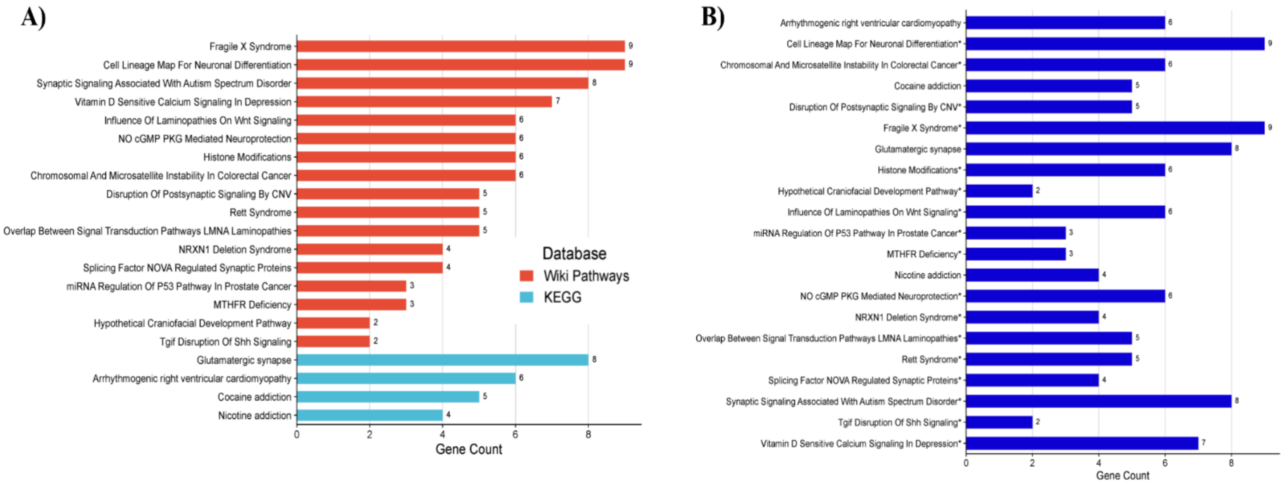

Supplementary Figure 4: HGD + Curcumin lncRNA Pathways. Pathway histograms for gene targets of differentially expressed lncRNAs, organized by **A)** database or **B)** alphabetically; statistically regulated pathways ($p < 0.05$) were identified using the Enrichr online database tool- KEGG (no asterisk), Wiki Pathways*, common to both**.

frontiers

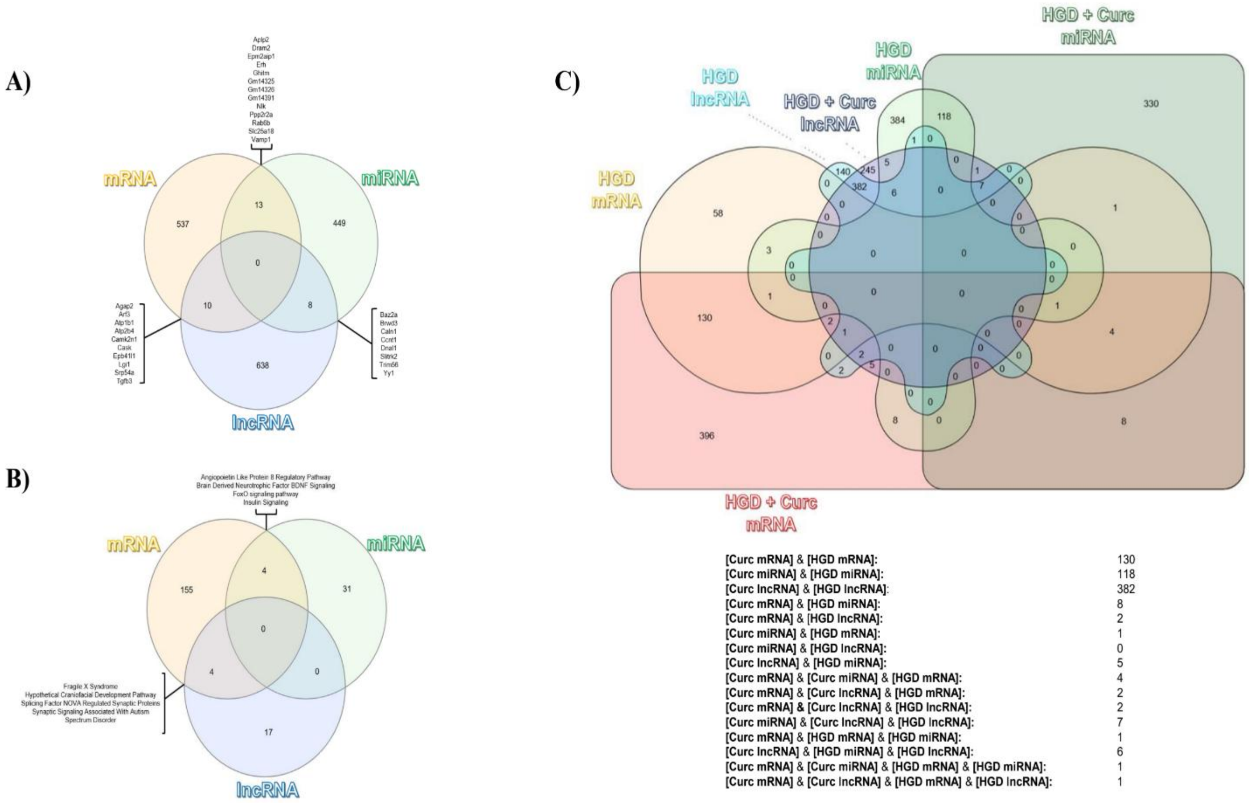

Supplementary Figure 5: HGD + Curcumin multi-genomic comparisons and global pathway network of hippocampal microvascular genome. **A,B)** Venn diagrams depicting overlap of differentially expressed **A)** genes and **B)** pathways amongst protein coding mRNAs and gene targets of non-coding miRNAs and lncRNAs. **C)** Venn diagram depicting the overlap between coding genes and non-coding gene targets of miRNAs and lncRNAs of both HGD+Curc and HGD.

Supplementary Table 1: All HGD/LGD DEGs: All differentially expressed genes (DEGs) of high glycemic diet (HGD) compared to low-glycemic diet (LGD)

Gene Symbol	Description	Fold Change	P-val
Dnajc4	DnaJ (Hsp40) homolog, subfamily C, member 4	1.97	3.16E-05
Gm16209	predicted gene 16209 [Source:MGI Symbol;Acc:MGI:3802095]	3.07	0.0001
TC0100001979.mm.1		-1.53	0.0003
Tubg1	tubulin, gamma 1	1.74	0.0003
TC0600002682.mm.1		2.79	0.0003
TC0900002041.mm.1		-1.54	0.0004
Gm25732	predicted gene, 25732 [Source:MGI Symbol;Acc:MGI:5455509]	1.97	0.0004
Gm24783	predicted gene, 24783 [Source:MGI Symbol;Acc:MGI:5454560]	-1.87	0.0005
Slc25a18	solute carrier family 25 (mitochondrial carrier), member 18	1.78	0.0005
Gm15665	predicted gene 15665 [Source:MGI Symbol;Acc:MGI:3783107]	-4.09	0.0006
Gm6254	predicted gene 6254	-1.62	0.0006
Jag1	jagged 1	1.95	0.0006
TC0200001598.mm.1		-1.85	0.0009
Gm6161	predicted gene 6161 [Source:MGI Symbol;Acc:MGI:3644104]	-1.62	0.0009
Scp2-ps2	sterol carrier protein 2, pseudogene 2 [Source:MGI Symbol;Acc:MGI:107679]	3.09	0.0009
Gm10171	predicted gene 10171	3.89	0.0009
Snord16a	small nucleolar RNA, C/D box 16A	4.77	0.0009
Ppp1r14b	protein phosphatase 1, regulatory (inhibitor) subunit 14B	1.84	0.001
Mir5125	microRNA 5125	-3.93	0.0012
Gm23387	predicted gene, 23387 [Source:MGI Symbol;Acc:MGI:5453164]	-1.71	0.0012
LOC100862392	uncharacterized LOC100862392 (LOC100862392), miscRNA.	1.82	0.0013
Gm24621	predicted gene, 24621 [Source:MGI Symbol;Acc:MGI:5454398]	-1.62	0.0015
Gast	Gastrin	-1.54	0.0015
LOC100861862	uncharacterized LOC100861862 (LOC100861862), miscRNA.	3.84	0.0017
TC1000001002.mm.1		-4.74	0.0018
TC0200001109.mm.1		-1.57	0.0018
TC1100002806.mm.1		-1.57	0.0018
TC0100003360.mm.1		-1.8	0.0019

TC1600001123.mm.1		2.4	0.0019
Gm19974	predicted gene, 19974 (Gm19974), miscRNA.	3.08	0.0019
Gm24400	predicted gene, 24400 [Source:MGI Symbol;Acc:MGI:5454177]	19.32	0.0019
TC1700001452.mm.1		-2.28	0.002
Brms1l	breast cancer metastasis-suppressor 1-like	2.76	0.002
TC1000002836.mm.1		-8.15	0.0021
Gm12565	predicted gene 12565 [Source:MGI Symbol;Acc:MGI:3652206]	1.54	0.0021
Gm6397	predicted gene 6397 [Source:MGI Symbol;Acc:MGI:3648542]	-1.55	0.0022
Pcyox1	prenylcysteine oxidase 1	1.54	0.0022
TC0900001583.mm.1		-1.62	0.0023
Triqk	triple QxxK/R motif containing	1.74	0.0023
Gm9103	predicted gene 9103 [Source:MGI Symbol;Acc:MGI:3645364]	1.83	0.0024
TC0100000555.mm.1		10.28	0.0024
Gm25153	predicted gene, 25153 [Source:MGI Symbol;Acc:MGI:5454930]	-2.03	0.0025
Gm25418	predicted gene, 25418 [Source:MGI Symbol;Acc:MGI:5455195]	-1.56	0.0025
TC1400000138.mm.1		1.59	0.0025
Mrpl49	mitochondrial ribosomal protein L49	1.62	0.0025
Cox6a1	cytochrome c oxidase subunit VIa polypeptide 1	3.07	0.0026
Abhd3	abhydrolase domain containing 3	2.01	0.0027
TC0900003046.mm.1		-3.07	0.0028
Tgfb3	transforming growth factor, beta 3	1.98	0.0028
TC1700001359.mm.1		-1.6	0.0029
Gm17651	predicted gene, 17651 [Source:MGI Symbol;Acc:MGI:4937285]	-4.38	0.0031
TC1800000696.mm.1		-1.55	0.0031
TC0300001750.mm.1		-2.27	0.0032
TC0500003017.mm.1		2.1	0.0032
Hnrnpa1l2-ps	heterogeneous nuclear ribonucleoprotein A1-like 2, pseudogene	-1.9	0.0033
Gm1947	predicted pseudogene 1947 [Source:MGI Symbol;Acc:MGI:3037805]	-1.65	0.0033
AY036118	cDNA sequence AY036118	1.8	0.0033
TC1200001378.mm.1		-1.63	0.0035
Gm23098	predicted gene, 23098	-15.71	0.0036
Gm25938	predicted gene, 25938 [Source:MGI Symbol;Acc:MGI:5455715]	-1.74	0.0036
TC0600000945.mm.1		-1.55	0.0036

Gabra3	gamma-aminobutyric acid (GABA) A receptor, subunit alpha 3	2.11	0.004
TC0700004096.mm.1		3.85	0.004
TC0300002909.mm.1		-1.56	0.0043
TC1300001294.mm.1		-1.99	0.0044
TC0800001779.mm.1		-1.55	0.0044
Gm12226	predicted pseudogene 12226 [Source:MGI Symbol;Acc:MGI:3649742]	1.67	0.0044
Arhgdia	Rho GDP dissociation inhibitor (GDI) alpha	1.58	0.0045
TC1700000557.mm.1		-1.78	0.0046
Ttc9b	tetratricopeptide repeat domain 9B	1.67	0.0046
Gm14516	predicted gene 14516 [Source:MGI Symbol;Acc:MGI:3705695]	1.88	0.0047
Camk2n1	calcium/calmodulin-dependent protein kinase II inhibitor 1	1.51	0.0048
Gm25992	predicted gene, 25992 [Source:MGI Symbol;Acc:MGI:5455769]	1.79	0.0048
Gm11522	predicted gene 11522 [Source:MGI Symbol;Acc:MGI:3650746]	-1.52	0.0049
TC0800001957.mm.1		-1.79	0.005
TC0400002885.mm.1		-1.6	0.0051
TC1500001757.mm.1		-1.53	0.0053
TC1100000243.mm.1		-1.95	0.0054
Gm23221	predicted gene, 23221	-1.8	0.0054
Ndufb6	NADH dehydrogenase (ubiquinone) 1 beta subcomplex, 6	2.58	0.0054
TC0600000156.mm.1		-1.63	0.0058
Ppm1d	protein phosphatase 1D magnesium-dependent, delta isoform	-1.61	0.0058
LOC100861787	uncharacterized LOC100861787, transcript variant 1 (LOC100861787), miscRNA.	1.5	0.0058
Tnfaip1	tumor necrosis factor, alpha-induced protein 1 (endothelial)	1.56	0.0059
TC0400003138.mm.1		1.81	0.0059
Gm11336	predicted gene 11336 [Source:MGI Symbol;Acc:MGI:3651860]	1.58	0.0061
Ost4	oligosaccharyltransferase 4 homolog (S. cerevisiae)	1.77	0.0061
Gm7381	predicted gene 7381 [Source:MGI Symbol;Acc:MGI:3643743]	6.73	0.0062
TC1900000003.mm.1		2.67	0.0063
LOC100861832	PREDICTED: uncharacterized LOC100861832 (LOC100861832), miscRNA.	2.76	0.0063
TC0600000837.mm.1		-1.55	0.0065
Gm26347	predicted gene, 26347 [Source:MGI Symbol;Acc:MGI:5456124]	-1.51	0.0065
TC1000001582.mm.1		-1.6	0.0066
Mir7048	microRNA 7048	-1.75	0.0067

Apoa1bp	apolipoprotein A-I binding protein	2.18	0.0067
TC0400003261.mm.1		2.58	0.0068
Lonp2	lon peptidase 2, peroxisomal	2.32	0.0069
Higd2a	HIG1 domain family, member 2A	11.4	0.007
Gstp1	glutathione S-transferase, pi 1	2.51	0.0071
TC1200001107.mm.1		-2.04	0.0073
Rpl10-ps4	ribosomal protein L10, pseudogene 4	1.74	0.0073
Mir8095	microRNA 8095	-2.28	0.0074
TC0X00000908.mm.1		-3.72	0.0075
Uck2	uridine-cytidine kinase 2	2.28	0.0076
TC0400003735.mm.1		-2.06	0.0077
Gm15590; Gm22774	predicted gene 15590 [Source:MGI Symbol;Acc:MGI:3831433]; predicted gene, 22774 [Source:MGI Symbol;Acc:MGI:5452551]	2.59	0.0077
Gm7332	predicted gene 7332	2.06	0.0078
Gm19933	predicted gene, 19933, transcript variant 2 (Gm19933), miscRNA.;	3.77	0.0078
TC1900001713.mm.1		1.79	0.0079
TC0200000028.mm.1		-6.25	0.008
LOC432823	similar to hypothetical protein MGC37588	2.74	0.008
Gatad1	GATA zinc finger domain containing 1	1.92	0.0081
TC1800000958.mm.1		1.73	0.0082
Gm25911	predicted gene, 25911 [Source:MGI Symbol;Acc:MGI:5455688]	4.16	0.0082
TC0600001961.mm.1		-2.56	0.0083
Gm15724; RP23-246B24.3	predicted gene 15724; novel pseudogene	-2.67	0.0084
TC1000000724.mm.1		-1.77	0.0084
TC0800002360.mm.1		1.57	0.0084
Gm22965	predicted gene, 22965 [Source:MGI Symbol;Acc:MGI:5452742]	-2.19	0.0085
Gm20701	predicted gene 20701 [Source:MGI Symbol;Acc:MGI:5313148]	-1.98	0.0086
Gm20391	predicted gene 20391 [Source:MGI Symbol;Acc:MGI:5141856]	-1.91	0.009
TC0900000944.mm.1		1.69	0.009
TC0900000740.mm.1		1.8	0.009
Gm14730	predicted gene 14730 [Source:MGI Symbol;Acc:MGI:3705542]	-1.51	0.0092
Gm19767	PREDICTED: predicted gene, 19767 (Gm19767), miscRNA.	2.06	0.0093
Mir5099	microRNA 5099	-1.99	0.0094
Mir5099	microRNA 5099	-1.99	0.0094

Gm14951; RP23-83P7.2	predicted gene 14951 [Source:MGI Symbol;Acc:MGI:3705251]; putative novel transcript	-1.57	0.0094
TC0500002367.mm.1		2.9	0.0096
Gm12074	predicted gene 12074 [Source:MGI Symbol;Acc:MGI:3650755]	1.95	0.0097
TC0700001319.mm.1		-2.84	0.0098
TC1200002058.mm.1		-1.79	0.0098
Ubc; Uba52	ubiquitin C; ubiquitin A-52 residue ribosomal protein fusion product 1	5.41	0.0098
Myl9	myosin, light polypeptide 9, regulatory	1.69	0.0099
Gm14165	predicted gene 14165 [Source:MGI Symbol;Acc:MGI:3650537]	2.04	0.0099
TC0600001048.mm.1		-1.51	0.01
TC0800002476.mm.1		1.89	0.01
Atp1b2	ATPase, Na+/K+ transporting, beta 2 polypeptide	2.15	0.01
Hist1h2aj	histone cluster 1, H2aj	6.81	0.0102
TC0500003033.mm.1		-2.04	0.0103
TC0900002318.mm.1		-1.66	0.0103
Dctn2	dynactin 2	1.85	0.0105
TC1400002488.mm.1		-1.58	0.0106
Gm24628	predicted gene, 24628	-1.57	0.0106
Gm15377	predicted gene 15377 [Source:MGI Symbol;Acc:MGI:3705615]	-1.5	0.0106
Gm13132	predicted gene 13132 [Source:MGI Symbol;Acc:MGI:3651366]	-4.42	0.0108
TC1700000547.mm.1		-2.03	0.0108
Pcna	proliferating cell nuclear antigen	2.29	0.0109
Gm1866	predicted gene 1866 [Source:MGI Symbol;Acc:MGI:3037724]	2.51	0.011
Plxnc1	plexin C1	1.68	0.0112
Prdx6-ps2	peroxiredoxin 6 pseudogene 2	-1.54	0.0114
TC1300001501.mm.1		-1.7	0.0115
TC0700001104.mm.1		1.58	0.0115
4930466F19RIK	Protein Gm10592; Protein Gm21968	2.56	0.0116
Gm10592	predicted gene 10592	2.56	0.0116
Gm21968	predicted gene, 21968	2.56	0.0116
TC1000000965.mm.1		-2.43	0.0117
Rab24	RAB24, member RAS oncogene family	1.9	0.0117
TC1300002570.mm.1		1.93	0.0117
Ranbp2-ps2	RAN binding protein 2, pseudogene 2	-1.55	0.0118
Wdr74	WD repeat domain 74	1.82	0.0118
TC0600003473.mm.1		-1.53	0.0121

n-R5s86	nuclear encoded rRNA 5S 86 [Source:MGI Symbol;Acc:MGI:4421934]	-2.27	0.0122
TC0600003458.mm.1		-2.57	0.0124
Eif2b5	eukaryotic translation initiation factor 2B, subunit 5 epsilon	1.51	0.0124
Gm5943	predicted pseudogene 5943 [Source:MGI Symbol;Acc:MGI:3646004]	1.8	0.0125
Gm19738	PREDICTED: predicted gene, 19738, transcript variant 1 (Gm19738), miscRNA.	3.99	0.0125
TC1800000844.mm.1		4.59	0.0126
TC1500001138.mm.1		-1.52	0.0127
Cox7a2l	cytochrome c oxidase subunit VIIa polypeptide 2-like	1.64	0.0127
Gm14777	predicted gene 14777 [Source:MGI Symbol;Acc:MGI:3705662]	1.7	0.0127
TC1200000556.mm.1		-1.68	0.0129
TC0500002868.mm.1		-1.61	0.0129
TC0800002291.mm.1		-1.63	0.013
TC0100000029.mm.1		-1.63	0.013
Mir7076	microRNA 7076	-1.6	0.0132
Gm8420	predicted gene 8420 [Source:MGI Symbol;Acc:MGI:3645594]	2.41	0.0133
TC0500002872.mm.1		-1.55	0.0134
Gm8730	predicted pseudogene 8730 [Source:MGI Symbol;Acc:MGI:3644565]	1.95	0.0135
Gm12254	predicted gene 12254 [Source:MGI Symbol;Acc:MGI:3650324]	3.21	0.0135
Gm15749; AC125183.7	predicted gene 15749 [Source:MGI Symbol;Acc:MGI:3783191]; Novel transcript	-1.58	0.0137
TC0100000178.mm.1		2.63	0.0138
TC1700002364.mm.1		2.63	0.0138
TC1400002268.mm.1		-1.57	0.014
Hist1h2af	histone cluster 1, H2af	4.05	0.0141
Hist1h2an	histone cluster 1, H2an	6.24	0.0141
TC1100000194.mm.1		-2.85	0.0142
TC1600000471.mm.1		-1.76	0.0142
Gm11037	predicted gene 11037 [Source:MGI Symbol;Acc:MGI:3779261]	-2.91	0.0144
TC1300000125.mm.1		-1.57	0.0144
Gm11633	predicted gene 11633 [Source:MGI Symbol;Acc:MGI:3650676]	1.67	0.0144
TC0900000475.mm.1		-1.56	0.0145
Copb2	coatomer protein complex, subunit beta 2 (beta prime)	1.56	0.0148
Gm19774	predicted gene, 19774 (Gm19774), mRNA.	4.52	0.0148
TC1500000845.mm.1		-1.62	0.0149

TC0700002348.mm.1		-1.57	0.0149
Gm22289	predicted gene, 22289 [Source:MGI Symbol;Acc:MGI:5452066]	12.31	0.0149
Plpp3	phospholipid phosphatase 3	1.91	0.015
LOC100861650	uncharacterized LOC100861650	3.12	0.015
TC0900002148.mm.1		3.78	0.015
TC1300000326.mm.1		-1.58	0.0151
TC0500000205.mm.1		1.64	0.0151
Ndufb7	NADH dehydrogenase (ubiquinone) 1 beta subcomplex, 7	2.12	0.0152
Rps4x	ribosomal protein S4, X-linked; ribosomal protein S4, X-linked (Rps4x), mRNA.	5.12	0.0152
TC0900000516.mm.1		1.88	0.0153
TC1200000390.mm.1		-1.56	0.0154
Crry-ps	complement receptor related protein, pseudogene [Source:MGI Symbol;Acc:MGI:88514]	2.66	0.0154
TC1800000734.mm.1		-1.63	0.0155
TC0X00003160.mm.1		-1.51	0.0156
TC0400001310.mm.1		-1.91	0.0157
Lage3	L antigen family, member 3	-1.67	0.0157
Gm7497	predicted gene 7497 [Source:MGI Symbol;Acc:MGI:3646548]	3	0.0157
TC1100000304.mm.1		-2.41	0.0159
Gm17051	predicted gene 17051 [Source:MGI Symbol;Acc:MGI:4937878]	-1.62	0.016
Nlk	nemo like kinase	1.97	0.016
Olfr692	olfactory receptor 692	-1.5	0.0162
TC1700000441.mm.1		-2.34	0.0163
Gm15680; AC103397.2	predicted gene 15680 [Source:MGI Symbol;Acc:MGI:3783122]; putative novel transcript	-2.09	0.0163
TC1200001016.mm.1		-1.59	0.0163
Hey2	hairy/enhancer-of-split related with YRPW motif 2	1.51	0.0163
TC1200000713.mm.1		1.8	0.0163
Ik	IK cytokine	1.84	0.0163
TC1500000820.mm.1		-1.52	0.0164
Rpsa-ps2	ribosomal protein SA, pseudogene 2 [Source:MGI Symbol;Acc:MGI:3643356]	7.19	0.0165
Gm4804	predicted gene 4804 [Source:MGI Symbol;Acc:MGI:3646648]	2.32	0.0166
Mir692-1	microRNA 692-1	4.97	0.0166
Gm25587	predicted gene, 25587 [Source:MGI Symbol;Acc:MGI:5455364]	-1.77	0.0167
TC1900001091.mm.1		-1.5	0.0167
Mir1947	microRNA 1947	-1.69	0.0169

TC0300000915.mm.1		-1.56	0.0169
Gm25140	predicted gene, 25140 [Source:MGI Symbol;Acc:MGI:5454917]	-1.74	0.017
Mir6964	microRNA 6964	-1.86	0.0172
Gm9242	predicted pseudogene 9242 [Source:MGI Symbol;Acc:MGI:3645172]	-1.63	0.0172
Fam205a1	family with sequence similarity 205, member A1	1.58	0.0173
TC1700002099.mm.1		1.64	0.0173
Gm20349	PREDICTED: predicted gene, 20349 (Gm20349), miscRNA.	3.06	0.0173
TC0200004093.mm.1		8.22	0.0174
Arl2	ADP-ribosylation factor-like 2	1.51	0.0175
Gm19886	PREDICTED: predicted gene, 19886 (Gm19886), miscRNA.	1.94	0.0177
TC1300001785.mm.1		2.97	0.0177
Gm11417; RP23-397J5.2	predicted gene 11417 [Source:MGI Symbol;Acc:MGI:3651779]; novel transcript	-1.54	0.0179
Atp6v1c1	ATPase, H+ transporting, lysosomal V1 subunit C1	1.58	0.0179
TC0500000460.mm.1		1.64	0.018
Rpl18-ps2	ribosomal protein L18, pseudogene 2; 60S ribosomal protein L18 (Rpl18) pseudogene	4.89	0.0181
Olfr1241	olfactory receptor 1241	-1.59	0.0182
Gm13249	predicted gene 13249	2.56	0.0182
Gm9294	predicted pseudogene 9294	2.8	0.0182
Gm23737	predicted gene, 23737 [Source:MGI Symbol;Acc:MGI:5453514]	-2.15	0.0183
TC0600003386.mm.1		-2.35	0.0185
Uqcrc1	ubiquinol-cytochrome c reductase core protein 1	1.89	0.0185
TC0600003007.mm.1		1.92	0.0186
Rpsa-ps4	ribosomal protein S4, pseudogene 4	4.64	0.0188
Gm14450	predicted gene 14450 [Source:MGI Symbol;Acc:MGI:3651431]	1.55	0.0189
Gm12726	predicted gene 12726 [Source:MGI Symbol;Acc:MGI:3649291]	2.35	0.0189
TC1100002935.mm.1		-1.54	0.0191
TC0100002570.mm.1		-1.58	0.0193
TC1100002078.mm.1		-2.23	0.0194
TC0200001416.mm.1		-2.67	0.0195
Gm6117; RP23-192D5.2	predicted gene 6117 [Source:MGI Symbol;Acc:MGI:3647032]; novel transcript	-1.66	0.0195
Sgpp2	sphingosine-1-phosphate phosphotase 2	2.4	0.0195
TC0200002802.mm.1		3.42	0.0195
TC0700000034.mm.1		-3.62	0.0197
TC1000001978.mm.1		-1.84	0.0197
TC1500000708.mm.1		2.22	0.0197

Gm11273	predicted gene 11273	3.4	0.0197
TC1900001403.mm.1		-1.63	0.0198
TC0X00001259.mm.1		-1.56	0.0198
Nhp2l1	NHP2 non-histone chromosome protein 2-like 1 (S. cerevisiae)	1.64	0.0199
Olfr912	olfactory receptor 912	-1.95	0.02
TC0500003353.mm.1		2.08	0.0201
Gm6055	predicted gene 6055	2.44	0.0201
TC0400003889.mm.1		-1.69	0.0202
TC0900000670.mm.1		-2.21	0.0203
TC0300002956.mm.1		1.72	0.0203
Uck2	uridine-cytidine kinase 2	3.06	0.0203
B230325K18Rik	RIKEN cDNA B230325K18 gene	-1.71	0.0204
TC0900001651.mm.1		-1.59	0.0204
Gm25595; Gm16106	predicted gene, 25595 [Source:MGI Symbol;Acc:MGI:5455372]; predicted gene 16106 [Source:MGI Symbol;Acc:MGI:3801781]	1.55	0.0204
TC1000000985.mm.1		-4.39	0.0205
Cuta	cutA divalent cation tolerance homolog (E. coli)	1.6	0.0205
Mir6928	microRNA 6928	-2.09	0.0206
Gm7204	predicted pseudogene 7204 [Source:MGI Symbol;Acc:MGI:3645884]	1.94	0.0206
TC0600000062.mm.1		-2.46	0.021
TC1700000574.mm.1		-1.53	0.0211
Sepw1	selenoprotein W, muscle 1	3	0.0211
TC1100004197.mm.1		-1.74	0.0214
Cox5b	cytochrome c oxidase subunit Vb	2.73	0.0214
TC0600000522.mm.1		-1.61	0.0215
TC0900001505.mm.1		1.91	0.0215
TC0500000114.mm.1		1.92	0.0215
Gm6245	predicted gene 6245 [Source:MGI Symbol;Acc:MGI:3646756]	2.21	0.0215
TC1100001865.mm.1		-1.53	0.0216
Gm20675; RP23-177N16.1	predicted gene 20675 [Source:MGI Symbol;Acc:MGI:5313122]; novel transcript	-1.83	0.0217
TC1100003355.mm.1		-1.55	0.0217
LOC100862094	PREDICTED: uncharacterized LOC100862094 (LOC100862094), miscRNA.	2.36	0.0217
TC0900000960.mm.1		-3.47	0.0218
Gm23662	predicted gene, 23662 [Source:MGI Symbol;Acc:MGI:5453439]	-1.67	0.0218
Gm25833	predicted gene, 25833 [Source:MGI Symbol;Acc:MGI:5455610]	-2.08	0.0219

TC1800000960.mm.1		-1.8	0.0219
Gm22202	predicted gene, 22202	-1.59	0.0219
Gm5	predicted gene 5; predicted gene 5 (Gm5), non-coding RNA.	-1.51	0.0219
Atp2b4; Mir6903	ATPase, Ca++ transporting, plasma membrane 4; microRNA 6903	2.09	0.022
Gm4953	predicted pseudogene 4953 [Source:MGI Symbol;Acc:MGI:3647627]	1.55	0.0221
Gm11386	predicted gene 11386 [Source:MGI Symbol;Acc:MGI:3649585]	1.62	0.0221
Mpzl1	myelin protein zero-like 1	2.14	0.0221
Gm17047	predicted gene 17047 [Source:MGI Symbol;Acc:MGI:4937874]	-2.24	0.0222
TC0900002885.mm.1		-2.05	0.0222
TC1700001495.mm.1		-2.05	0.0222
TC1800000057.mm.1		-2.05	0.0222
Epm2aip1	EPM2A (laforin) interacting protein 1	1.71	0.0222
TC1500000169.mm.1		-3.34	0.0224
Gm25109	predicted gene, 25109 [Source:MGI Symbol;Acc:MGI:5454886]	-2.97	0.0224
TC0500001708.mm.1		-1.58	0.0224
Gm17257	predicted gene, 17257 [Source:MGI Symbol;Acc:MGI:4936891]	2.22	0.0224
Gm2933; Gmcl1l	predicted gene 2933 (Gm2933), mRNA	-1.65	0.0225
Opalin	oligodendrocytic myelin paranodal and inner loop protein	-1.59	0.0225
Rgs21	regulator of G-protein signalling 21	-1.59	0.0225
Cd248	CD248 antigen, endosialin	2	0.0225
Gm12251	predicted gene 12251	3.85	0.0225
Pfkm	phosphofructokinase, muscle	1.51	0.0227
TC0600002621.mm.1		2.07	0.0227
Gm16339; RP24-369B4.1	predicted gene 16339 [Source:MGI Symbol;Acc:MGI:3840123]; novel transcript	-1.81	0.0228
Pts	6-pyruvoyl-tetrahydropterin synthase	3.5	0.0228
Gm11367	predicted gene 11367 [Source:MGI Symbol;Acc:MGI:3652074]	3.57	0.0228
TC1100003307.mm.1		-2.29	0.0229
TC1700001763.mm.1		-1.79	0.0229
Gm26278	predicted gene, 26278 [Source:MGI Symbol;Acc:MGI:5456055]	-1.63	0.023
Pabpc1	poly(A) binding protein, cytoplasmic 1	1.68	0.023
Gmps	guanine monophosphate synthetase; guanine monphosphate synthetase	2.03	0.023
Hist1h2al	histone cluster 1, H2al	2.59	0.023
Resp18	regulated endocrine-specific protein 18	7.73	0.023

TC1800000420.mm.1		-1.6	0.0233
Gm7399	predicted gene 7399 [Source:MGI Symbol;Acc:MGI:3647495]	-1.57	0.0233
Zfp36l1	zinc finger protein 36, C3H type-like 1	1.75	0.0233
TC1300000913.mm.1		1.51	0.0236
TC0500001812.mm.1		1.86	0.0236
Gm16354	predicted gene 16354 [Source:MGI Symbol;Acc:MGI:3840116]	2.42	0.0236
TC0400003340.mm.1		-2.11	0.0237
TC1000002949.mm.1		-2.11	0.0238
TC0800001985.mm.1		-1.72	0.0239
TC0100001808.mm.1		6.63	0.0239
Gm14209	predicted gene 14209 [Source:MGI Symbol;Acc:MGI:3649779]; putative novel transcript	-2.42	0.024
Cdc40	cell division cycle 40	1.54	0.024
TC1600001870.mm.1		1.93	0.024
Gm7887	predicted gene 7887 [Source:MGI Symbol;Acc:MGI:3648251]	2.13	0.024
Gm12618	predicted gene 12618	3	0.024
LOC100862145	uncharacterized LOC100862145, transcript variant 1	1.52	0.0241
TC1100001845.mm.1		1.83	0.0241
Gm22730	predicted gene, 22730 [Source:MGI Symbol;Acc:MGI:5452507]	-2.72	0.0242
Rpsa-ps12	ribosomal protein SA, pseudogene 12	2.97	0.0242
TC0X00000528.mm.1		-1.67	0.0243
Gm19496	PREDICTED: predicted gene, 19496 (Gm19496), miscRNA.	6.97	0.0243
TC1200001247.mm.1		-1.86	0.0245
TC0700001911.mm.1		2.15	0.0245
TC0500003354.mm.1		-1.79	0.0246
Vkorc1	vitamin K epoxide reductase complex, subunit 1	8.64	0.0246
Gm14604; RP23-260P9.1	predicted gene 14604; novel pseudogene	-2.74	0.0247
Mir1941	microRNA 1941	-2.01	0.0247
Fndc4	fibronectin type III domain containing 4; fibronectin type III domain containing 4 (Fndc4), mRNA.	1.52	0.0247
TC1200001872.mm.1		-2.59	0.0249
Lrp1	low density lipoprotein receptor-related protein 1	1.73	0.025
TC0200001568.mm.1		-2.3	0.0251
Tmem176b	transmembrane protein 176B	1.7	0.0251
Pik3c2a	phosphatidylinositol 3-kinase, C2 domain containing, alpha polypeptide	1.56	0.0254
Ctsb	cathepsin B	2.75	0.0254
TC0300001003.mm.1		-3	0.0255

Gm3208	predicted gene 3208 [Source:MGI Symbol;Acc:MGI:3781387]	-1.57	0.0255
Gm11511	predicted gene 11511 [Source:MGI Symbol;Acc:MGI:3649770]	3.28	0.0255
Dtymk	deoxythymidylate kinase	1.64	0.0256
Gm16238	predicted gene 16238 [Source:MGI Symbol;Acc:MGI:3802104]	2.02	0.0256
Gm17756	PREDICTED: predicted gene, 17756 (Gm17756), mRNA.	1.97	0.0257
Gm8652	predicted gene 8652 [Source:MGI Symbol;Acc:MGI:3643616]	-2.05	0.0258
D5Ertd683e	DNA segment, Chr 5, ERATO Doi 683, expressed (D5Ertd683e), miscRNA.	-1.87	0.0258
TC1700001106.mm.1		-1.67	0.0258
TC0800002885.mm.1		-1.61	0.026
TC1400002541.mm.1		2.19	0.026
Clasp2	CLIP associating protein 2; CLIP associating protein 2 (Clasp2), transcript variant 1, mRNA.	1.51	0.0261
Gstp2	glutathione S-transferase, pi 2	2.07	0.0261
Cox5a	cytochrome c oxidase subunit Va	7.18	0.0261
Mylip	myosin regulatory light chain interacting protein	-1.63	0.0262
TC1300001200.mm.1		10.19	0.0264
TC0700004255.mm.1		-1.8	0.0265
Prelid1	PRELI domain containing 1	1.55	0.0265
TC0700004127.mm.1		2.79	0.0265
TC1700000976.mm.1		-1.53	0.0266
TC1600000613.mm.1		-2.02	0.0267
Mirlet7f-1	microRNA let7f-1; microRNA let7f-1 (Mirlet7f-1), microRNA.	-1.99	0.0267
Rassf3	Ras association (RalGDS/AF-6) domain family member 3	1.63	0.0267
Dap3	death associated protein 3	1.65	0.0267
Ndufc1	NADH dehydrogenase (ubiquinone) 1, subcomplex unknown, 1	1.52	0.0268
Smim13	small integral membrane protein 13	1.53	0.0268
Gm13573	predicted gene 13573 [Source:MGI Symbol;Acc:MGI:3652221]	2.82	0.0269
Ighv10-1	immunoglobulin heavy variable 10-1	-1.62	0.027
TC0100003596.mm.1		1.65	0.027
TC1300001214.mm.1		-1.81	0.0271
TC0200001495.mm.1		-1.69	0.0271
Gm25260	predicted gene, 25260 [Source:MGI Symbol;Acc:MGI:5455037]	-1.65	0.0271
TC0800002640.mm.1		-1.76	0.0272
TC1000000631.mm.1		-1.59	0.0277

TC1000001880.mm.1		-1.55	0.0277
Gm25712	predicted gene, 25712 [Source:MGI Symbol;Acc:MGI:5455489]	-1.53	0.0277
Gm16050; CH36-125B10.7	predicted gene 16050 [Source:MGI Symbol;Acc:MGI:3801837]; Novel transcript	-1.57	0.0278
Pla2g10os	phospholipase A2, group X, opposite strand	-1.54	0.0278
TC0100002048.mm.1		-1.95	0.028
TC0900002700.mm.1		-1.52	0.028
Cltb	clathrin, light polypeptide (Lcb)	1.56	0.028
TC1600001059.mm.1		2.81	0.0281
Rpl10; Snora70	ribosomal protein L10; small nucleolar RNA, H/ACA box 70	1.51	0.0282
Gm7502	predicted gene 7502 [Source:MGI Symbol;Acc:MGI:3643245]	1.75	0.0283
Stk39	serine/threonine kinase 39	2.05	0.0284
Gm14670	predicted gene 14670 [Source:MGI Symbol;Acc:MGI:3705370]	-2.47	0.0285
Gm22769	predicted gene, 22769 [Source:MGI Symbol;Acc:MGI:5452546]	-2	0.0285
Gm6444	predicted gene 6444 [Source:MGI Symbol;Acc:MGI:3648226]	3.57	0.0286
Gm13680	predicted gene 13680 [Source:MGI Symbol;Acc:MGI:3652285]	10.61	0.0287
Gm9625	predicted gene 9625 [Source:MGI Symbol;Acc:MGI:3780033]	2.08	0.0289
TC1900000146.mm.1		1.86	0.029
Gm24357	predicted gene, 24357 [Source:MGI Symbol;Acc:MGI:5454134]	4.67	0.029
Atf2	activating transcription factor 2	2.1	0.0291
Maf	avian musculoaponeurotic fibrosarcoma (v-maf) AS42 oncogene homolog	2.13	0.0291
Gm11749; RP23-386D6.3	predicted gene 11749 [Source:MGI Symbol;Acc:MGI:3652041]; novel transcript	-1.66	0.0292
TC1600002029.mm.1		-1.73	0.0293
Lin7a	lin-7 homolog A (C. elegans)	2.32	0.0294
Grina	glutamate receptor, ionotropic, N-methyl D-aspartate-associated protein 1 (glutamate binding)	4.3	0.0294
Scarb2	scavenger receptor class B, member 2	1.76	0.0295
LOC100861642	PREDICTED: uncharacterized LOC100861642 (LOC100861642), miscRNA.	2.61	0.0295
TC1600000882.mm.1		6.02	0.0296
Slc9a9	solute carrier family 9 (sodium/hydrogen exchanger), member 9	1.81	0.0298
Uqcr11	ubiquinol-cytochrome c reductase, complex III subunit XI	2.16	0.0298

Gm7286	predicted gene 7286 [Source:MGI Symbol;Acc:MGI:3645875]	2.89	0.0298
Odc1	ornithine decarboxylase, structural 1	3.05	0.0298
Gm3091	predicted gene 3091 [Source:MGI Symbol;Acc:MGI:3781267]	-1.51	0.0299
Gm23330	predicted gene, 23330 [Source:MGI Symbol;Acc:MGI:5453107]	-2.62	0.03
TC0900003192.mm.1		-1.78	0.03
D8Ertd738e	DNA segment, Chr 8, ERATO Doi 738, expressed	2.4	0.03
Taldo1	transaldolase 1	1.76	0.0301
TC1000000237.mm.1		-3.86	0.0302
Lynx1	Ly6/neurotoxin 1	1.54	0.0302
Capns1	calpain, small subunit 1	1.62	0.0302
Gm5139	predicted gene 5139 [Source:MGI Symbol;Acc:MGI:3779465]	1.89	0.0302
TC1600001476.mm.1		3.51	0.0302
TC1900000412.mm.1		-2.11	0.0304
Vamp1	vesicle-associated membrane protein 1	2.14	0.0304
TC1000000501.mm.1		-2.07	0.0305
TC1400001905.mm.1		-1.55	0.0307
Ighv1-54	immunoglobulin heavy variable V1-54	-1.74	0.0308
Gm15028	predicted gene 15028 [Source:MGI Symbol;Acc:MGI:3705685]	-1.59	0.0308
TC1100003668.mm.1		-1.57	0.0308
Gm14388	predicted gene 14388 [Source:MGI Symbol;Acc:MGI:3650926]	2.41	0.0308
Gm11628	predicted gene 11628 [Source:MGI Symbol;Acc:MGI:3651276]	2.54	0.0308
TC1000002228.mm.1		-1.69	0.0309
TC0100001704.mm.1		1.84	0.0309
TC0100003511.mm.1		-1.57	0.031
Gm14734; RP23-272G10.3	predicted gene 14734; novel pseudogene	-2.16	0.0311
TC0X00003293.mm.1		1.53	0.0311
Tmem59l	transmembrane protein 59-like	1.71	0.0311
Hist2h4	histone cluster 2, H4	2.04	0.0311
Gm17541	predicted gene, 17541 [Source:MGI Symbol;Acc:MGI:4937175]	4.23	0.0312
TC0900001688.mm.1		-1.94	0.0313
Maged1	melanoma antigen, family D, 1	1.63	0.0313
Gm11688	predicted gene 11688 [Source:MGI Symbol;Acc:MGI:3650868]	2.18	0.0313
TC1300001308.mm.1		2.24	0.0313

Rbm4	RNA binding motif protein 4	1.85	0.0314
TC1600000864.mm.1		-1.79	0.0316
Hist1h1c	histone cluster 1, H1c	2.73	0.0317
Psmb5; Mir686	proteasome (prosome, macropain) subunit, beta type 5; microRNA 686	2.51	0.0318
Cisd1	CDGSH iron sulfur domain 1	1.99	0.0319
Gm12816	predicted gene 12816	2.21	0.0321
TC0600003233.mm.1		-1.8	0.0322
TC0500000668.mm.1		2.24	0.0322
LOC100862384	PREDICTED: uncharacterized LOC100862384 (LOC100862384), miscRNA.	1.72	0.0323
Gm2710; Gm10075	predicted gene 2710 [Source:MGI Symbol;Acc:MGI:3780879]; predicted gene 10075 [Source:MGI Symbol;Acc:MGI:3710521]	-1.68	0.0324
Pnmal1	PNMA-like 1	1.52	0.0325
Rabac1	Rab acceptor 1 (prenylated)	3.74	0.0325
Slc38a3	solute carrier family 38, member 3	1.65	0.0326
TC1000001757.mm.1		1.77	0.0326
TC0600002803.mm.1		1.93	0.0326
Eef2	eukaryotic translation elongation factor 2	2.34	0.0326
Dennd6a	DENN/MADD domain containing 6A	1.56	0.0329
Rpl19-ps12	ribosomal protein L19, pseudogene 12	3.04	0.0329
LOC100861649	uncharacterized LOC100861649, transcript variant 1	1.76	0.033
TC0400001061.mm.1		1.98	0.033
TC0200004276.mm.1		2.11	0.033
TC1700001338.mm.1		3.01	0.033
TC0X00002812.mm.1		-2.08	0.0332
TC0200003537.mm.1		-1.51	0.0332
Gm5417	predicted gene 5417 [Source:MGI Symbol;Acc:MGI:3648626]	2.31	0.0332
Gm6829	predicted pseudogene 6829 [Source:MGI Symbol;Acc:MGI:3648590]	2.6	0.0332
TC1400002602.mm.1		-1.95	0.0333
Map3k7cl	Map3k7 C-terminal like	-1.51	0.0333
TC0600002130.mm.1		1.5	0.0333
n-R5s127	nuclear encoded rRNA 5S 127 [Source:MGI Symbol;Acc:MGI:4421983]	-1.51	0.0334
Ano6	anoctamin 6	1.6	0.0334
Oxct1	3-oxoacid CoA transferase 1	1.68	0.0334
TC1000001969.mm.1		1.61	0.0335
Gm26983	predicted gene, 26983 [Source:MGI Symbol;Acc:MGI:5504098]	2.29	0.0335
Ppa1	pyrophosphatase (inorganic) 1	1.75	0.0336

Gm23119	predicted gene, 23119 [Source:MGI Symbol;Acc:MGI:5452896]	2.44	0.0336
TC0800001971.mm.1		-2.05	0.0338
Ufd1l	ubiquitin fusion degradation 1 like	1.52	0.034
Ifi27	interferon, alpha-inducible protein 27	2.14	0.034
TC0100003086.mm.1		2.24	0.034
TC1100000103.mm.1		-1.92	0.0341
Gm11675	predicted gene 11675 [Source:MGI Symbol;Acc:MGI:3650970]	2.55	0.0341
Prpf8	pre-mRNA processing factor 8	2.93	0.0341
TC1400001018.mm.1		-1.6	0.0343
Rprl1	ribonuclease P RNA-like 1	-1.63	0.0344
Mir6347	microRNA 6347 [Source:MGI Symbol;Acc:MGI:5531245]	-1.5	0.0344
Mir6347	microRNA 6347	-1.5	0.0344
TC0400000418.mm.1		-2.07	0.0345
TC1200000360.mm.1		-1.87	0.0345
Gm13171	predicted gene 13171 [Source:MGI Symbol;Acc:MGI:3651089]	1.72	0.0346
Mir7053	microRNA 7053	-2.18	0.0347
TC1900000956.mm.1		-1.65	0.0347
Gm25630	predicted gene, 25630 [Source:MGI Symbol;Acc:MGI:5455407]	-1.57	0.0349
TC1600000578.mm.1		-2.62	0.035
Gm17069	predicted gene 17069 [Source:MGI Symbol;Acc:MGI:4937896]	-1.61	0.035
Tsn	Translin	1.85	0.035
TC1800000772.mm.1		-1.88	0.0351
Gm26826	predicted gene, 26826 [Source:MGI Symbol;Acc:MGI:5477320]	-1.56	0.0351
TC0100002672.mm.1		1.72	0.0351
Snord82	small nucleolar RNA, C/D box 82	-3.27	0.0352
Gm13018; RP23-416J8.1	predicted gene 13018; novel pseudogene	-1.61	0.0352
Ephx1	epoxide hydrolase 1, microsomal	2.55	0.0352
TC1000000989.mm.1		-1.9	0.0353
TC0400001114.mm.1		-1.58	0.0353
Babam1	BRISC and BRCA1 A complex member 1	2.17	0.0354
Clk3	CDC-like kinase 3	2.12	0.0355
Gm11956	predicted gene 11956 [Source:MGI Symbol;Acc:MGI:3649806]	1.58	0.0356
TC1000002010.mm.1		-1.77	0.0358
Ndufa13	NADH dehydrogenase (ubiquinone) 1 alpha subcomplex, 13	3.82	0.0359
TC1900001387.mm.1		-1.65	0.036

TC1000001217.mm.1		-1.65	0.036
Rpl18	ribosomal protein L18	1.59	0.036
TC1100002615.mm.1		-2.22	0.0365
TC1500001507.mm.1		-1.52	0.0365
Gm4017; Gm10157	predicted gene 4017 [Source:MGI Symbol;Acc:MGI:3782191]; predicted gene 10157 [Source:MGI Symbol;Acc:MGI:3642264]	7.95	0.0366
Gm26361	predicted gene, 26361 [Source:MGI Symbol;Acc:MGI:5456138]	-1.81	0.0367
TC0200001159.mm.1		-1.68	0.0367
Sf3a3	splicing factor 3a, subunit 3	1.68	0.0368
TC1100004086.mm.1		2.08	0.0368
Gm13436	predicted gene 13436	2.35	0.0368
TC1100004012.mm.1		2.73	0.0368
TC0200000837.mm.1		1.55	0.0369
TC0400001849.mm.1		-1.88	0.037
Gm13413	predicted gene 13413 [Source:MGI Symbol;Acc:MGI:3650613]	3.58	0.037
TC1500000243.mm.1		-1.69	0.0371
Mir1961	microRNA 1961	-1.59	0.0371
TC1500001650.mm.1		1.61	0.0371
Cdc27	cell division cycle 27; Synthetic construct	1.56	0.0372
Gm14150	predicted gene 14150	3.82	0.0372
Mzt1	mitotic spindle organizing protein 1	5.77	0.0372
TC1200001181.mm.1		1.62	0.0373
Tmem106b	transmembrane protein 106B	1.81	0.0373
Gkn3	gastrokine 3	2.11	0.0373
TC1300002065.mm.1		-2.32	0.0376
TC0300001917.mm.1		-1.53	0.0376
Napb	N-ethylmaleimide sensitive fusion protein attachment protein beta	2.39	0.0376
Gm13044; RP23-58H5.7	predicted gene 13044; novel pseudogene	-1.93	0.0377
TC0600000159.mm.1		-1.74	0.0378
TC1300000419.mm.1		1.51	0.0378
Ccng1	cyclin G1	2.21	0.0378
Atl2	atlastin GTPase 2; atlastin GTPase 2 (Atl2), transcript variant 2, mRNA.	2.01	0.038
Gm26951; RP23-114E15.10	predicted gene, 26951; novel transcript	1.65	0.0381
Gm24258	predicted gene, 24258 [Source:MGI Symbol;Acc:MGI:5454035]	-1.79	0.0383

Gm26455	predicted gene, 26455 [Source:MGI Symbol;Acc:MGI:5456232]	-1.55	0.0383
Gm25722	predicted gene, 25722	-1.54	0.0383
Fnta	farnesyltransferase, CAAX box, alpha	1.53	0.0383
TC0700000955.mm.1		2.15	0.0383
TC1300000008.mm.1		-1.74	0.0384
Gm16418	predicted pseudogene 16418 [Source:MGI Symbol;Acc:MGI:3644548]	2.48	0.0384
TC0900001581.mm.1		2.73	0.0385
TC1100002930.mm.1		-1.61	0.0386
Gm24588	predicted gene, 24588 [Source:MGI Symbol;Acc:MGI:5454365]	2.2	0.0387
Pcna-ps2	proliferating cell nuclear antigen pseudogene 2 [Source:MGI Symbol;Acc:MGI:97505]	2.28	0.0387
TC0400001459.mm.1		1.69	0.0388
Crbn	cereblon; cereblon (Crbn), transcript variant 2, mRNA.	1.8	0.0388
Tmem30a	transmembrane protein 30A	3.09	0.0388
Uchl1	ubiquitin carboxy-terminal hydrolase L1	2.3	0.0389
Gm26121	predicted gene, 26121 [Source:MGI Symbol;Acc:MGI:5455898]	-2.42	0.0391
Pgam1-ps2	phosphoglycerate mutase 1, pseudogene 2	1.55	0.0391
TC0600003398.mm.1		1.77	0.0391
Ncam1	neural cell adhesion molecule 1	2.06	0.0392
TC1100001364.mm.1		2.2	0.0392
Mir5125	microRNA 5125	-6.45	0.0393
TC0500001040.mm.1		3.17	0.0393
Gm5265	predicted pseudogene 5265 [Source:MGI Symbol;Acc:MGI:3643416]	4.28	0.0393
Gm23448	predicted gene, 23448 [Source:MGI Symbol;Acc:MGI:5453225]	-1.53	0.0394
Cyfip2	cytoplasmic FMR1 interacting protein 2	1.58	0.0394
Mtch1	mitochondrial carrier homolog 1 (C. elegans)	1.63	0.0394
TC0700002668.mm.1		1.7	0.0394
Mir7212	microRNA 7212	-2.41	0.0395
TC0600001298.mm.1		-1.69	0.0395
Gm22687	predicted gene, 22687 [Source:MGI Symbol;Acc:MGI:5452464]	-1.6	0.0395
Tspan6	tetraspanin 6	-1.5	0.0395
TC1800001098.mm.1		-2.08	0.0396
TC1600000884.mm.1		-1.76	0.0396
Rbx1	ring-box 1	3.07	0.0396
TC1200001368.mm.1		3.14	0.0396
Tspyl4	TSPY-like 4	2.18	0.0397

Fam168a	family with sequence similarity 168, member A	1.76	0.0398
Gm14292	predicted gene 14292 [Source:MGI Symbol;Acc:MGI:3649964]	7.55	0.0398
Gm2531	predicted gene 2531 [Source:MGI Symbol;Acc:MGI:3780699]	-2.5	0.0399
TC0400001488.mm.1		-1.63	0.0399
LOC100861762	PREDICTED: uncharacterized LOC100861762 (LOC100861762), miscRNA.	1.55	0.0399
Trappc11	trafficking protein particle complex 11	1.62	0.0399
Polr2j	polymerase (RNA) II (DNA directed) polypeptide J	1.64	0.0399
Mir5128	microRNA 5128	-1.94	0.04
Cul3	cullin 3	1.58	0.04
Gm13123	predicted gene 13123	1.66	0.04
TC0400002879.mm.1		2.65	0.04
TC0300000540.mm.1		-1.83	0.0403
TC1200000112.mm.1		-1.72	0.0403
Mir6926	microRNA 6926	-1.71	0.0403
Eif5al3-ps	eukaryotic translation initiation factor 5A-like 3, pseudogene	2.82	0.0403
TC1700001629.mm.1		-1.7	0.0404
Gm24255	predicted gene, 24255 [Source:MGI Symbol;Acc:MGI:5454032]	-1.55	0.0404
Polr2b	polymerase (RNA) II (DNA directed) polypeptide B	1.66	0.0404
Ube2nl	ubiquitin-conjugating enzyme E2N-like [Source:MGI Symbol;Acc:MGI:3643295]	2.56	0.0404
Gm10167	predicted pseudogene 10167 [Source:MGI Symbol;Acc:MGI:3704263]	-1.74	0.0405
Gm25407	predicted gene, 25407 [Source:MGI Symbol;Acc:MGI:5455184]	-1.68	0.0405
Rpl14-ps1	ribosomal protein L14, pseudogene 1 [Source:MGI Symbol;Acc:MGI:3710579]	2.45	0.0405
Gm5239	predicted pseudogene 5239 [Source:MGI Symbol;Acc:MGI:3648788]	2.64	0.0405
Mrpl27	mitochondrial ribosomal protein L27	1.5	0.0406
Hivep1	human immunodeficiency virus type I enhancer binding protein 1;	1.64	0.0406
Gm12892	predicted gene 12892 [Source:MGI Symbol;Acc:MGI:3649649]	1.54	0.0408
TC1100001421.mm.1		2.93	0.0408
TC0Y00000112.mm.1		-1.76	0.0409
Tubb2a	tubulin, beta 2A class IIA	3.37	0.0409
TC1900001212.mm.1		-1.66	0.041
Tmem14c	transmembrane protein 14C	1.67	0.041
Carm1	coactivator-associated arginine methyltransferase 1	1.66	0.0411

Rpsa	ribosomal protein SA	2.61	0.0411
TC1000000990.mm.1		-3.18	0.0413
Gm24348	predicted gene, 24348 [Source:MGI Symbol;Acc:MGI:5454125]	-1.59	0.0413
Slc2a13	solute carrier family 2 (facilitated glucose transporter), member 13	1.57	0.0413
Gm22663	predicted gene, 22663 [Source:MGI Symbol;Acc:MGI:5452440]	-2.01	0.0414
TC0400003474.mm.1		-1.57	0.0414
TC0800001990.mm.1		-1.51	0.0414
Tbc1d9	TBC1 domain family, member 9	1.55	0.0414
Gm19475	PREDICTED: predicted gene, 19475 (Gm19475), mRNA.	2.62	0.0414
Gm13797	predicted gene 13797 [Source:MGI Symbol;Acc:MGI:3652035]	13.45	0.0414
Fads3	fatty acid desaturase 3	1.64	0.0415
Gm22646	predicted gene, 22646 [Source:MGI Symbol;Acc:MGI:5452423]	1.52	0.0416
Gm6969	predicted pseudogene 6969 [Source:MGI Symbol;Acc:MGI:3645320]	2.53	0.0416
Rpsa-ps10	ribosomal protein SA, pseudogene 10 [Source:MGI Symbol;Acc:MGI:3704228]	4.17	0.0416
Pcdh9	protocadherin 9	2.39	0.0417
Mlc1	megalencephalic leukoencephalopathy with subcortical cysts 1 homolog (human)	1.78	0.0418
TC1400002546.mm.1		1.9	0.0421
Rplp1-ps1	ribosomal protein, large, P1, pseudogene 1	2.05	0.0421
Gm23240	predicted gene, 23240 [Source:MGI Symbol;Acc:MGI:5453017]	-1.52	0.0422
Papd4	PAP associated domain containing 4	2.36	0.0423
Gm12141; Hspd1-ps3	predicted gene 12141; heat shock protein 1 (chaperonin), pseudogene 3 [Source:MGI Symbol;Acc:MGI:3651246]	1.78	0.0424
TC0400003906.mm.1		-1.9	0.0426
TC0300002640.mm.1		-1.76	0.0426
Ano1	anoctamin 1, calcium activated chloride channel	1.53	0.0426
Sccpdh	saccharopine dehydrogenase (putative)	1.55	0.0427
Psma6	proteasome (prosome, macropain) subunit, alpha type 6	1.69	0.0428
TC0900002861.mm.1		-3.08	0.0429
Gm17126	predicted gene 17126 [Source:MGI Symbol;Acc:MGI:4937953]	-1.52	0.0429
Bola2	bolA-like 2 (E. coli)	1.55	0.0431
TC1400001678.mm.1		3.44	0.0431
Gm12238	predicted gene 12238	-6.23	0.0432
TC1500001590.mm.1		-2.03	0.0432

Nfe2l1	nuclear factor, erythroid derived 2,-like 1	1.65	0.0433
Gm11539	predicted gene 11539 [Source:MGI Symbol;Acc:MGI:3651911]	2.38	0.0433
TC0100000036.mm.1		-1.55	0.0434
Vmn1r66	vomeronasal 1 receptor 66	-1.54	0.0434
TC0100002100.mm.1		-2.67	0.0435
Sdhb	succinate dehydrogenase complex, subunit B, iron sulfur (Ip)	1.88	0.0435
Rps2-ps13	ribosomal protein S2, pseudogene 13	3.92	0.0435
TC0X00001537.mm.1		-3.06	0.0437
Gm14645	predicted gene 14645 [Source:MGI Symbol;Acc:MGI:3705691]	-1.59	0.0437
TC1000000972.mm.1		-3.17	0.0438
TC0100001854.mm.1		-1.7	0.0438
TC1300001873.mm.1		-1.63	0.0438
4930473O22Rik	RIKEN cDNA 4930473O22 gene; RIKEN cDNA 4930473O22 gene (4930473O22Rik), non-coding RNA.	-1.55	0.0438
Gm14176	predicted gene 14176	1.53	0.0439
Gm26199	predicted gene, 26199	2	0.0439
TC1400000956.mm.1		-1.51	0.044
TC1100001403.mm.1		1.66	0.0441
Ighv5-12-4	Ighv5-12-4 immunoglobulin heavy variable 5-12-4	-1.92	0.0442
Fam73a	family with sequence similarity 73, member A	1.71	0.0442
Msl2	male-specific lethal 2 homolog (Drosophila); male-specific lethal 2 homolog (Drosophila) (Msl2), mRNA.	2.18	0.0442
Prdx4	peroxiredoxin 4	2.24	0.0442
Rpl19-ps4; Gm27506	ribosomal protein L19, pseudogene 4	3	0.0442
Rpl10-ps1	ribosomal protein L10, pseudogene 1	5.08	0.0442
TC0500001897.mm.1		-1.59	0.0443
Mcts1	malignant T cell amplified sequence 1	1.6	0.0443
Tuba1b	tubulin, alpha 1B	2.34	0.0443
Gm25436	predicted gene, 25436 [Source:MGI Symbol;Acc:MGI:5455213]	-1.56	0.0444
TC1000001386.mm.1		-1.82	0.0445
TC1000002972.mm.1		-1.82	0.0445
LOC100862121	PREDICTED: uncharacterized LOC100862121 (LOC100862121), miscRNA.	1.54	0.0446
Ddn	dendrin	1.87	0.0448
Rpl10-ps6	ribosomal protein L10, pseudogene 6	4.81	0.0448
TC0100002114.mm.1		-1.64	0.0449
TC1600001530.mm.1		-1.61	0.0449
Ube2n	ubiquitin-conjugating enzyme E2N	1.78	0.0449
TC0900001929.mm.1		2.61	0.0449
Id1	inhibitor of DNA binding 1	1.65	0.045

Gm13298; Fam205a4; Gm20938	predicted gene 13298 (Gm13298), mRNA.; family with sequence similarity 205, member A4 [Source:MGI Symbol;Acc:MGI:5434294]; predicted gene, 20938	2.02	0.045
Gm26701	predicted gene, 26701 [Source:MGI Symbol;Acc:MGI:5477195]	2.26	0.045
Mirlet7a-1	microRNA let7a-1	-1.94	0.0451
Gm17221	predicted gene 17221 [Source:MGI Symbol;Acc:MGI:4938048]	-1.56	0.0451
TC0800001046.mm.1		1.62	0.0451
Zfp931	zinc finger protein 931	1.89	0.0451
TC1600000839.mm.1		1.96	0.0451
TC0600002179.mm.1		-2.98	0.0452
Cct5	chaperonin containing Tcp1, subunit 5 (epsilon)	1.73	0.0452
TC1400000128.mm.1		12.12	0.0452
Gm12424	predicted gene 12424 [Source:MGI Symbol;Acc:MGI:3650991]	-1.94	0.0453
Grip1os2	glutamate receptor interacting protein 1, opposite strand 2	-1.56	0.0453
Gm19494	predicted gene, 19494, transcript variant 1 (Gm19494), miscRNA.	6.06	0.0453
Olfr456	olfactory receptor 456	-1.6	0.0455
TC0400000208.mm.1		1.54	0.0455
Gm4735	predicted gene 4735	6.47	0.0455
Gm17428	predicted gene, 17428 [Source:MGI Symbol;Acc:MGI:4937062]	-3.76	0.0456
Tdrd3	tudor domain containing 3	1.64	0.0456
Dusp6	dual specificity phosphatase 6	1.6	0.0457
Rtn4	reticulon 4	1.65	0.0458
Lancl1	LanC (bacterial lantibiotic synthetase component C)-like 1	1.69	0.0458
LOC100862246	PREDICTED: uncharacterized LOC100862246 (LOC100862246), miscRNA.	2.62	0.0458
Gm27626	predicted gene, 27626 [Source:MGI Symbol;Acc:MGI:5531008]	10.84	0.0459
TC0400000440.mm.1		-1.54	0.046
Smdt1	single-pass membrane protein with aspartate rich tail 1	5.81	0.0461
TC0500003042.mm.1		-1.54	0.0462
Gabbr2	gamma-aminobutyric acid (GABA) B receptor, 2	1.75	0.0462
Atp5l2-ps	ATP synthase, H+ transporting, mitochondrial FO complex, subunit G2, pseudogene	4.65	0.0462
Drg1	developmentally regulated GTP binding protein 1	5.28	0.0464
Gm25581	predicted gene, 25581 [Source:MGI Symbol;Acc:MGI:5455358]	-2.43	0.0465
Gm26308	predicted gene, 26308 [Source:MGI Symbol;Acc:MGI:5456085]	-1.62	0.0465

Rplp0	ribosomal protein, large, P0	1.51	0.0465
TC1200002202.mm.1		1.54	0.0465
Nol4	nucleolar protein 4	1.57	0.0465
Rheb	Ras homolog enriched in brain	1.64	0.0465
LOC100861833	uncharacterized LOC100861833 (LOC100861833), miscRNA.	2.52	0.0465
TC0600000497.mm.1		-1.63	0.0466
Gm16580	predicted gene 16580 [Source:MGI Symbol;Acc:MGI:4415000]	3.6	0.0467
TC1200000510.mm.1		-1.77	0.0469
8430408G22Rik	RIKEN cDNA 8430408G22 gene	1.61	0.0469
TC0500000141.mm.1		1.55	0.047
Mtmr6	myotubularin related protein 6	1.85	0.047
Vps28	vacuolar protein sorting 28 (yeast)	4.33	0.0471
Gm26264	predicted gene, 26264 [Source:MGI Symbol;Acc:MGI:5456041]	-1.81	0.0472
Gm24519	predicted gene, 24519 [Source:MGI Symbol;Acc:MGI:5454296]	-1.63	0.0472
Spcs2	signal peptidase complex subunit 2 homolog (S. cerevisiae)	3.41	0.0472
Raph1	Ras association (RalGDS/AF-6) and pleckstrin homology domains 1	1.96	0.0473
TC0400000306.mm.1		1.86	0.0474
TC1700001387.mm.1		-8.09	0.0475
TC0800000683.mm.1		2.12	0.0475
TC1600001480.mm.1		2.74	0.0476
Actg-ps1; Gm23812	actin, gamma, pseudogene 1 [Source:MGI Symbol;Acc:MGI:87907]; predicted gene, 23812 [Source:MGI Symbol;Acc:MGI:5453589]	2.45	0.0477
Gm9840	predicted gene 9840	3.91	0.0477
Gm22087	predicted gene, 22087 [Source:MGI Symbol;Acc:MGI:5451864]	-2.13	0.0478
TC0100000104.mm.1		2.31	0.0478
TC1200001267.mm.1		-2.13	0.048
Cpt1a	carnitine palmitoyltransferase 1a, liver	1.93	0.048
Gm11478	predicted gene 11478 [Source:MGI Symbol;Acc:MGI:3650888]	5.38	0.048
Rpsa-ps9	ribosomal protein SA, pseudogene 9	3.58	0.0481
Gm3934	PREDICTED: predicted gene 3934 (Gm3934), mRNA.	1.73	0.0482
Dram2	DNA-damage regulated autophagy modulator 2; VDNA-damage regulated autophagy modulator 2	1.89	0.0482
Oat	ornithine aminotransferase	1.55	0.0483
Etnk1	ethanolamine kinase 1	5.47	0.0483

Gm8152	predicted gene 8152 [Source:MGI Symbol;Acc:MGI:3644504]	-1.69	0.0484
Gm23753	predicted gene, 23753 [Source:MGI Symbol;Acc:MGI:5453530]	-1.81	0.0486
Rnf113a2	ring finger protein 113A2	1.53	0.0486
Gm5384	predicted gene 5384 [Source:MGI Symbol;Acc:MGI:3646660]	1.92	0.0486
Slc12a2	solute carrier family 12, member 2	1.89	0.0487
Ttc9	tetratricopeptide repeat domain 9	1.63	0.049
Gm9727	predicted gene 9727 [Source:MGI Symbol;Acc:MGI:3779431]	1.7	0.049
Gm15487; Atp6v0c	predicted gene 15487; Source:MGI Symbol;Acc:MGI:3709610]; ATPase, H+ transporting, lysosomal V0 subunit C	4.2	0.0491
Scarna3a; Mir1843b	small Cajal body-specific RNA 3A; microRNA 1843b	-3.11	0.0492
Gm23245	predicted gene, 23245 [Source:MGI Symbol;Acc:MGI:5453022]	1.92	0.0493
Ostc	oligosaccharyltransferase complex subunit	4.19	0.0493
Gm27684	predicted gene, 27684 [Source:MGI Symbol;Acc:MGI:5531066]	6.07	0.0493
Gm21954	predicted gene, 21954 [Source:MGI Symbol;Acc:MGI:5439423]	1.82	0.0494
Gm26557	predicted gene, 26557 [Source:MGI Symbol;Acc:MGI:5477051]	-1.53	0.0495
Irak1bp1	interleukin-1 receptor-associated kinase 1 binding protein 1	1.61	0.0495
TC0200005131.mm.1		-1.81	0.0496
Calb2	calbindin 2	1.54	0.0496
Gm13422	predicted gene 13422 [Source:MGI Symbol;Acc:MGI:3650167]	1.65	0.0496
TC1900001466.mm.1		-2.33	0.0497
Gm3531	predicted pseudogene 3531 [Source:MGI Symbol;Acc:MGI:3781708]	2.6	0.0497
Mir7081	microRNA 7081	-1.55	0.0498
Traj59	T cell receptor alpha joining 59	-2.65	0.0499
Arl5a	ADP-ribosylation factor-like 5A	1.92	0.05

Supplementary Table 2: HGD/LGD Coding. Protein coding genes (mRNA) differentially expressed by HGD relative to LGD

Gene Symbol	Name	Fold Change	P-val
Dnajc4	DnaJ (Hsp40) homolog, subfamily C, member 4	1.97	0.0000316
Tubg1	tubulin, gamma 1	1.74	0.0003
Slc25a18	solute carrier family 25 (mitochondrial carrier), member 18	1.78	0.0005

Jag1	jagged 1	1.95	0.0006
Ppp1r14b	protein phosphatase 1, regulatory (inhibitor) subunit 14B	1.84	0.001
Gast	Gastrin	-1.54	0.0015
Brms1l	breast cancer metastasis-suppressor 1-like	2.76	0.002
Pcyox1	prenylcysteine oxidase 1	1.54	0.0022
Triqk	triple QxxK/R motif containing	1.74	0.0023
Mrpl49	mitochondrial ribosomal protein L49	1.62	0.0025
Cox6a1	cytochrome c oxidase subunit VIa polypeptide 1	3.07	0.0026
Abhd3	abhydrolase domain containing 3	2.01	0.0027
Tgfb3	transforming growth factor, beta 3	1.98	0.0028
Gm17651	predicted gene, 17651	-4.38	0.0031
Gabra3	gamma-aminobutyric acid (GABA) A receptor, subunit alpha 3	2.11	0.004
Arl2	Rho GDP dissociation inhibitor (GDI) alpha	1.58	0.0045
Ttc9b	tetratricopeptide repeat domain 9B	1.67	0.0046
Camk2n1	calcium/calmodulin-dependent protein kinase II inhibitor 1	1.51	0.0048
Ndufb6	NADH dehydrogenase (ubiquinone) 1 beta subcomplex, 6	2.58	0.0054
Ppm1d	protein phosphatase 1D magnesium-dependent, delta isoform	-1.61	0.0058
Tnfaip1	tumor necrosis factor, alpha-induced protein 1 (endothelial)	1.56	0.0059
Ost4	oligosaccharyltransferase 4 homolog (S. cerevisiae)	1.77	0.0061
Arhgdia	apolipoprotein A-I binding protein	2.18	0.0067
Lonp2	lon peptidase 2, peroxisomal	2.32	0.0069
Higd2a	HIG1 domain family, member 2A	11.4	0.007
Gstp1	glutathione S-transferase, pi 1	2.51	0.0071
Uck2	uridine-cytidine kinase 2	2.28	0.0076
Gatad1	GATA zinc finger domain containing 1	1.92	0.0081
Ubc	ubiquitin C	5.41	0.0098
Myl9	myosin, light polypeptide 9, regulatory	1.69	0.0099
Atp2b4	ATPase, Na+/K+ transporting, beta 2 polypeptide	2.15	0.01
Dctn2	dynactin 2	1.85	0.0105
Pcna	proliferating cell nuclear antigen	2.29	0.0109
Plxnc1	plexin C1	1.68	0.0112
Rab24	RAB24, member RAS oncogene family	1.9	0.0117
Wdr74	WD repeat domain 74	1.82	0.0118
Eif2b5	eukaryotic translation initiation factor 2B, subunit 5 epsilon	1.51	0.0124
Cox7a2l	cytochrome c oxidase subunit VIIa polypeptide 2-like	1.64	0.0127
Gm11037	predicted gene 11037	-2.91	0.0144
Copb2	coatomer protein complex, subunit beta 2 (beta prime)	1.56	0.0148
Plpp3	phospholipid phosphatase 3	1.91	0.015
Ndufb7	NADH dehydrogenase (ubiquinone) 1 beta subcomplex, 7	2.12	0.0152
Rps4x	ribosomal protein S4, X-linked	5.12	0.0152
Ctsb	complement receptor related protein, pseudogene	2.66	0.0154
Lage3	L antigen family, member 3	-1.67	0.0157

Nlk	nemo like kinase	1.97	0.016
Olfr692	olfactory receptor 692	-1.5	0.0162
Hey2	hairy/enhancer-of-split related with YRPW motif 2	1.51	0.0163
Ik	IK cytokine	1.84	0.0163
Fam205a1	family with sequence similarity 205, member A1	1.58	0.0173
Arl5a	ADP-ribosylation factor-like 2	1.51	0.0175
Atp6v1c1	ATPase, H+ transporting, lysosomal V1 subunit C1	1.58	0.0179
Olfr1241	olfactory receptor 1241	-1.59	0.0182
Uqcrc1	ubiquinol-cytochrome c reductase core protein 1	1.89	0.0185
Sgpp2	sphingosine-1-phosphate phosphotase 2	2.4	0.0195
Olfr912	olfactory receptor 912	-1.95	0.02
Cyfip2	cutA divalent cation tolerance homolog (E. coli)	1.6	0.0205
Cox5b	cytochrome c oxidase subunit Vb	2.73	0.0214
Mpzl1	myelin protein zero-like 1	2.14	0.0221
Epm2aip1	EPM2A (laforin) interacting protein 1	1.71	0.0222
Cd248	CD248 antigen, endosialin	2	0.0225
Opalin	oligodendrocytic myelin paranodal and inner loop protein	-1.59	0.0225
Rgs21	regulator of G-protein signalling 21	-1.59	0.0225
Pfkm	phosphofructokinase, muscle	1.51	0.0227
Pts	6-pyruvoyl-tetrahydropterin synthase	3.5	0.0228
Gmps	guanine monophosphate synthetase	2.03	0.023
Pabpc1	poly(A) binding protein, cytoplasmic 1	1.68	0.023
Resp18	regulated endocrine-specific protein 18	7.73	0.023
Zfp36l1	zinc finger protein 36, C3H type-like 1	1.75	0.0233
Cdc40	cell division cycle 40	1.54	0.024
Vkorc1	vitamin K epoxide reductase complex, subunit 1	8.64	0.0246
Fndc4	fibronectin type III domain containing 4	1.52	0.0247
Lrp1	low density lipoprotein receptor-related protein 1	1.73	0.025
Tmem176b	transmembrane protein 176B	1.7	0.0251
Cul3	cathepsin B	2.75	0.0254
Pik3c2a	phosphatidylinositol 3-kinase, C2 domain containing, alpha polypeptide	1.56	0.0254
Dtymk	deoxythymidylate kinase	1.64	0.0256
Clasp2	CLIP associating protein 2	1.51	0.0261
Cox5a	cytochrome c oxidase subunit Va	7.18	0.0261
Gstp2	glutathione S-transferase, pi 2	2.07	0.0261
Mylip	myosin regulatory light chain interacting protein	-1.63	0.0262
Prelid1	PRELI domain containing 1	1.55	0.0265
Dap3	death associated protein 3	1.65	0.0267
Rassf3	Ras association (RalGDS/AF-6) domain family member 3	1.63	0.0267
Ndufc1	NADH dehydrogenase (ubiquinone) 1, subcomplex unknown, 1	1.52	0.0268
Smim13	small integral membrane protein 13	1.53	0.0268

Cltb	clathrin, light polypeptide (Lcb)	1.56	0.028
Rpl10	ribosomal protein L10	1.51	0.0282
Stk39	serine/threonine kinase 39	2.05	0.0284
Atf2	activating transcription factor 2	2.1	0.0291
Maf	avian musculoaponeurotic fibrosarcoma (v-maf) AS42 oncogene homolog	2.13	0.0291
Grina	glutamate receptor, ionotropic, N-methyl D-aspartate-associated protein 1 (glutamate binding)	4.3	0.0294
Lin7a	lin-7 homolog A (C. elegans)	2.32	0.0294
Scarb2	scavenger receptor class B, member 2	1.76	0.0295
Odc1	ornithine decarboxylase, structural 1	3.05	0.0298
Slc9a9	solute carrier family 9 (sodium/hydrogen exchanger), member 9	1.81	0.0298
Uqcr11	ubiquinol-cytochrome c reductase, complex III subunit XI	2.16	0.0298
Taldo1	transaldolase 1	1.76	0.0301
Capns1	calpain, small subunit 1	1.62	0.0302
Lynx1	Ly6/neurotoxin 1	1.54	0.0302
Vamp1	vesicle-associated membrane protein 1	2.14	0.0304
Tmem59l	transmembrane protein 59-like	1.71	0.0311
Maged1	melanoma antigen, family D, 1	1.63	0.0313
Rbm4	RNA binding motif protein 4	1.85	0.0314
Psmb5	proteasome (prosome, macropain) subunit, beta type 5	2.51	0.0318
Cisd1	CDGSH iron sulfur domain 1	1.99	0.0319
Pnmal1	PNMA-like 1	1.52	0.0325
Rabac1	Rab acceptor 1 (prenylated)	3.74	0.0325
Eef2	eukaryotic translation elongation factor 2	2.34	0.0326
Slc38a3	solute carrier family 38, member 3	1.65	0.0326
Dennd6a	DENN/MADD domain containing 6A	1.56	0.0329
Map3k7cl	Map3k7 C-terminal like	-1.51	0.0333
Ano6	anoctamin 6	1.6	0.0334
Oxct1	3-oxoacid CoA transferase 1	1.68	0.0334
Ppa1	pyrophosphatase (inorganic) 1	1.75	0.0336
Ifi27	interferon, alpha-inducible protein 27	2.14	0.034
Prpf8	pre-mRNA processing factor 8	2.93	0.0341
Tsn	Translin	1.85	0.035
Ephx1	epoxide hydrolase 1, microsomal	2.55	0.0352
Babam1	BRISC and BRCA1 A complex member 1	2.17	0.0354
Clk3	CDC-like kinase 3	2.12	0.0355
Ndufa13	NADH dehydrogenase (ubiquinone) 1 alpha subcomplex, 13	3.82	0.0359
Rpl18	ribosomal protein L18	1.59	0.036
Sf3a3	splicing factor 3a, subunit 3	1.68	0.0368
Cdc27	cell division cycle 27	1.56	0.0372
Mzt1	mitotic spindle organizing protein 1	5.77	0.0372

Gkn3	gastrokine 3	2.11	0.0373
Tmem106b	transmembrane protein 106B	1.81	0.0373
Napb	N-ethylmaleimide sensitive fusion protein attachment protein beta	2.39	0.0376
Ccng1	cyclin G1	2.21	0.0378
Atp1b2	atlastin GTPase 2	2.01	0.038
Fnta	farnesyltransferase, CAAX box, alpha	1.53	0.0383
Crbn	Cereblon	1.8	0.0388
Tmem30a	transmembrane protein 30A	3.09	0.0388
Uchl1	ubiquitin carboxy-terminal hydrolase L1	2.3	0.0389
Ncam1	neural cell adhesion molecule 1	2.06	0.0392
D8Ertd738e	cytoplasmic FMR1 interacting protein 2	1.58	0.0394
Mtch1	mitochondrial carrier homolog 1 (C. elegans)	1.63	0.0394
Tspan6	tetraspanin 6	-1.5	0.0395
Rbx1	ring-box 1	3.07	0.0396
Tspyl4	TSPY-like 4	2.18	0.0397
Fam168a	family with sequence similarity 168, member A	1.76	0.0398
Polr2j	polymerase (RNA) II (DNA directed) polypeptide J	1.64	0.0399
Trappc11	trafficking protein particle complex 11	1.62	0.0399
Cuta	cullin 3	1.58	0.04
Polr2b	polymerase (RNA) II (DNA directed) polypeptide B	1.66	0.0404
Hivep1	human immunodeficiency virus type I enhancer binding protein 1	1.64	0.0406
Mrpl27	mitochondrial ribosomal protein L27	1.5	0.0406
Tubb2a	tubulin, beta 2A class IIA	3.37	0.0409
Tmem14c	transmembrane protein 14C	1.67	0.041
Carm1	coactivator-associated arginine methyltransferase 1	1.66	0.0411
Rpsa	ribosomal protein SA	2.61	0.0411
Slc2a13	solute carrier family 2 (facilitated glucose transporter), member 13	1.57	0.0413
Tbc1d9	TBC1 domain family, member 9	1.55	0.0414
Fads3	fatty acid desaturase 3	1.64	0.0415
Pcdh9	protocadherin 9	2.39	0.0417
Mlc1	megalencephalic leukoencephalopathy with subcortical cysts 1 homolog (human)	1.78	0.0418
Ano1	anoctamin 1, calcium activated chloride channel	1.53	0.0426
Sccpdh	saccharopine dehydrogenase (putative)	1.55	0.0427
Psma6	proteasome (prosome, macropain) subunit, alpha type 6	1.69	0.0428
Bola2	bolA-like 2 (E. coli)	1.55	0.0431
Nfe2l1	nuclear factor, erythroid derived 2,-like 1	1.65	0.0433
Vmn1r66	vomeronasal 1 receptor 66	-1.54	0.0434
Sdhb	succinate dehydrogenase complex, subunit B, iron sulfur (Ip)	1.88	0.0435
Msl2	male-specific lethal 2 homolog (Drosophila)	2.18	0.0442
Prdx4	peroxiredoxin 4	2.24	0.0442

Mcts1	malignant T cell amplified sequence 1	1.6	0.0443
Tuba1b	tubulin, alpha 1B	2.34	0.0443
Ddn	dendrin	1.87	0.0448
Ube2n	ubiquitin-conjugating enzyme E2N	1.78	0.0449
Id1	inhibitor of DNA binding 1	1.65	0.045
Zfp931	zinc finger protein 931	1.89	0.0451
Cct5	chaperonin containing Tcp1, subunit 5 (epsilon)	1.73	0.0452
Olfr456	olfactory receptor 456	-1.6	0.0455
Gm17428	predicted gene, 17428	-3.76	0.0456
Tdrd3	tudor domain containing 3	1.64	0.0456
Dusp6	dual specificity phosphatase 6	1.6	0.0457
Lancl1	LanC (bacterial lantibiotic synthetase component C)-like 1	1.69	0.0458
Rtn4	reticulon 4	1.65	0.0458
Smdt1	single-pass membrane protein with aspartate rich tail 1	5.81	0.0461
Gabbr2	gamma-aminobutyric acid (GABA) B receptor, 2	1.75	0.0462
Drg1	developmentally regulated GTP binding protein 1	5.28	0.0464
Nol4	nucleolar protein 4	1.57	0.0465
Rheb	Ras homolog enriched in brain	1.64	0.0465
Rplp0	ribosomal protein, large, P0	1.51	0.0465
Mtmr6	myotubularin related protein 6	1.85	0.047
Vps28	vacuolar protein sorting 28 (yeast)	4.33	0.0471
Spcs2	signal peptidase complex subunit 2 homolog (S. cerevisiae)	3.41	0.0472
Raph1	Ras association (RalGDS/AF-6) and pleckstrin homology domains 1	1.96	0.0473
Cpt1a	carnitine palmitoyltransferase 1a, liver	1.93	0.048
Dram2	DNA-damage regulated autophagy modulator 2	1.89	0.0482
Etnk1	ethanolamine kinase 1	5.47	0.0483
Oat	ornithine aminotransferase	1.55	0.0483
Rnf113a2	ring finger protein 113A2	1.53	0.0486
Slc12a2	solute carrier family 12, member 2	1.89	0.0487
Ttc9	tetratricopeptide repeat domain 9	1.63	0.049
Ostc	oligosaccharyltransferase complex subunit	4.19	0.0493
Gm21954	predicted gene, 21954	1.82	0.0494
Irak1bp1	interleukin-1 receptor-associated kinase 1 binding protein 1	1.61	0.0495
Calb2	calbindin 2	1.54	0.0496
Atf2	ADP-ribosylation factor-like 5A	1.92	0.05

Supplementary Table 3: HGD/LGD miRNAs. Non-coding microRNAs differentially expressed by HGD relative to LGD

Gene Symbol	Name	5p/3p strand	Fold Change	P-val
Mir5125	microRNA 5125	mmu-miR-5125	-3.93	0.0012

Mir7048	microRNA 7048	mmu-miR-7048-5p	-1.75	0.0067
Mir8095	microRNA 8095	mmu-miR-8095	-2.28	0.0074
Mir5099	microRNA 5099	mmu-miR-5099	-1.99	0.0094
Mir7076	microRNA 7076	mmu-miR-7076-5p	-1.6	0.0132
Mir692-1	microRNA 692-1	mmu-miR-692	4.97	0.0166
Mir1947	microRNA 1947	mmu-miR-1947-5p	-1.69	0.0169
Mir6964	microRNA 6964	mmu-miR-6964-5p	-1.86	0.0172
Mir6928	microRNA 6928	mmu-miR-6928-3p	-2.09	0.0206
Mir1941	microRNA 1941	mmu-miR-1941-5p	-2.01	0.0247
Mirlet7f-1	microRNA let7f-1	mmu-let-7f-5p	-1.99	0.0267
Mir6347	microRNA 6347	mmu-miR-6347	-1.5	0.0344
Mir7053	microRNA 7053	mmu-miR-7053-5p	-2.18	0.0347
Mir1961	microRNA 1961	mmu-miR-1961	-1.59	0.0371
Mir7212	microRNA 7212	mmu-miR-7212-5p	-2.41	0.0395
Mir5128	microRNA 5128	mmu-miR-5128	-1.94	0.04
Mir6926	microRNA 6926	mmu-miR-6926-5p	-1.71	0.0403
Mirlet7a-1	microRNA let7a-1	mmu-let-7a-5p	-1.94	0.0451
Mir7081	microRNA 7081	mmu-miR-7081-3p	-1.55	0.0498

Supplementary Table 4: HGD/LGD lncRNAs. Long non-coding RNAs (lncRNAs) differentially expressed by HGD relative to LGD

Gene Symbol	Name	Fold Change	P-val
Gm15680	predicted gene 15665 [Source:MGI Symbol;Acc:MGI:3783107]	-4.09	0.0006
Gm14951	predicted gene 14951 [Source:MGI Symbol;Acc:MGI:3705251]; putative novel transcript	-1.57	0.0094
Gm15749	predicted gene 15749 [Source:MGI Symbol;Acc:MGI:3783191]; Novel transcript	-1.58	0.0137
Gm11417	predicted gene 11417 [Source:MGI Symbol;Acc:MGI:3651779]; novel transcript	-1.54	0.0179
Gm6117	predicted gene 6117 [Source:MGI Symbol;Acc:MGI:3647032]; novel transcript	-1.66	0.0195
Gm20675	predicted gene 20675 [Source:MGI Symbol;Acc:MGI:5313122]; novel transcript	-1.83	0.0217
Gm5	predicted gene 5; predicted gene 5 (Gm5), non-coding RNA.	-1.51	0.0219
Gm16339	predicted gene 16339 [Source:MGI Symbol;Acc:MGI:3840123]; novel transcript	-1.81	0.0228

Gm14209	predicted gene 14209 [Source:MGI Symbol;Acc:MGI:3649779]; putative novel transcript	-2.42	0.024
Gm16050	predicted gene 16050 [Source:MGI Symbol;Acc:MGI:3801837]; Novel transcript	-1.57	0.0278
Pla2g10os	phospholipase A2, group X, opposite strand	-1.54	0.0278
Gm11749	predicted gene 11749 [Source:MGI Symbol;Acc:MGI:3652041]; novel transcript	-1.66	0.0292
Gm26826	predicted gene, 26826 [Source:MGI Symbol;Acc:MGI:5477320]	-1.56	0.0351
Gm12238	predicted gene 12238	-6.23	0.0432
4930473O22Rik	RIKEN cDNA 4930473O22 gene; RIKEN cDNA 4930473O22 gene (4930473O22Rik), non-coding RNA.	-1.55	0.0438
Grip1os2	glutamate receptor interacting protein 1, opposite strand 2	-1.56	0.0453

Supplementary Table 5: HGD/LGD snoRNAs. Non-coding small nucleus/nucleolar RNAs differentially expressed by HGD relative to LGD

Gene Symbol	Name	Fold Change	P-val
Gm24621	predicted gene, 24621 [Source:MGI Symbol;Acc:MGI:5454398]	-1.62	0.0015
Gm24400	predicted gene, 24400 [Source:MGI Symbol;Acc:MGI:5454177]	19.32	0.0019
Gm25418	predicted gene, 25418 [Source:MGI Symbol;Acc:MGI:5455195]	-1.56	0.0025
Gm25938	predicted gene, 25938 [Source:MGI Symbol;Acc:MGI:5455715]	-1.74	0.0036
Gm26347	predicted gene, 26347 [Source:MGI Symbol;Acc:MGI:5456124]	-1.51	0.0065
Gm22965	predicted gene, 22965 [Source:MGI Symbol;Acc:MGI:5452742]	-2.19	0.0085
Gm25587	predicted gene, 25587 [Source:MGI Symbol;Acc:MGI:5455364]	-1.77	0.0167
Gm25140	predicted gene, 25140 [Source:MGI Symbol;Acc:MGI:5454917]	-1.74	0.017
Gm23737	predicted gene, 23737 [Source:MGI Symbol;Acc:MGI:5453514]	-2.15	0.0183
Gm25833	predicted gene, 25833 [Source:MGI Symbol;Acc:MGI:5455610]	-2.08	0.0219
Gm26278	predicted gene, 26278 [Source:MGI Symbol;Acc:MGI:5456055]	-1.63	0.023
Gm25260	predicted gene, 25260 [Source:MGI Symbol;Acc:MGI:5455037]	-1.65	0.0271
Gm22769	predicted gene, 22769 [Source:MGI Symbol;Acc:MGI:5452546]	-2	0.0285
Gm24357	predicted gene, 24357 [Source:MGI Symbol;Acc:MGI:5454134]	4.67	0.029
Gm23119	predicted gene, 23119 [Source:MGI Symbol;Acc:MGI:5452896]	2.44	0.0336
Gm25630	predicted gene, 25630 [Source:MGI Symbol;Acc:MGI:5455407]	-1.57	0.0349
Snord82	small nucleolar RNA, C/D box 82	-3.27	0.0352
Gm26361	predicted gene, 26361 [Source:MGI Symbol;Acc:MGI:5456138]	-1.81	0.0367
Gm24258	predicted gene, 24258 [Source:MGI Symbol;Acc:MGI:5454035]	-1.79	0.0383
Gm26121	predicted gene, 26121 [Source:MGI Symbol;Acc:MGI:5455898]	-2.42	0.0391
Gm24255	predicted gene, 24255 [Source:MGI Symbol;Acc:MGI:5454032]	-1.55	0.0404

Gm25407	predicted gene, 25407 [Source:MGI Symbol;Acc:MGI:5455184]	-1.68	0.0405
Gm24348	predicted gene, 24348 [Source:MGI Symbol;Acc:MGI:5454125]	-1.59	0.0413
Gm22663	predicted gene, 22663 [Source:MGI Symbol;Acc:MGI:5452440]	-2.01	0.0414
Gm23240	predicted gene, 23240 [Source:MGI Symbol;Acc:MGI:5453017]	-1.52	0.0422
Gm25436	predicted gene, 25436 [Source:MGI Symbol;Acc:MGI:5455213]	-1.56	0.0444
Gm25581	predicted gene, 25581 [Source:MGI Symbol;Acc:MGI:5455358]	-2.43	0.0465
Gm26264	predicted gene, 26264 [Source:MGI Symbol;Acc:MGI:5456041]	-1.81	0.0472
Gm22087	predicted gene, 22087 [Source:MGI Symbol;Acc:MGI:5451864]	-2.13	0.0478
Gm23245	predicted gene, 23245 [Source:MGI Symbol;Acc:MGI:5453022]	1.92	0.0493

Supplementary Table 6: All HGD+Curc/HGD DEGs. All differentially expressed genes (DEGs) of high-glycemic diet + curcumin 0.2% in diet (HGD+Curc) compared to high-glycemic diet (HGD)

Gene Symbol	Description	Fold Change	P-val
Gm24187	predicted gene, 24187	-300.14	1.77E-08
Gm24245	predicted gene, 24245	-265.79	2.08E-08
Gm24270	predicted gene, 24270	-265.79	2.08E-08
Gm23935	predicted gene, 23935	-14.54	8.61E-08
Gm23388	predicted gene, 23388	-84.5	1.10E-06
Gm25732	predicted gene, 25732	-6.2	1.27E-06
Gm25911	predicted gene, 25911	-366.62	1.78E-06
Dnajc4	DnaJ (Hsp40) homolog, subfamily C, member 4	-2.38	8.13E-06
Sult2a-ps3	sulfotransferase family 2A, dehydroepiandrosterone (DHEA)-preferring, pseudogene 3	1.78	1.57E-05
TC1500002241.mm.1		1.9	3.58E-05
TC1600001123.mm.1		-5.06	5.43E-05
Park7	Parkinson disease (autosomal recessive, early onset) 7	-1.95	5.46E-05
TC0200002707.mm.1		1.54	7.24E-05
Gm25992	predicted gene, 25992	-2.58	7.70E-05
Lgals1	lectin, galactose binding, soluble 1	-2.21	9.21E-05
4930413M19Rik	RIKEN cDNA 4930413M19 gene	1.59	0.0001

Arhgef7	Rho guanine nucleotide exchange factor (GEF7)	-1.68	0.0001
AY036118	cDNA sequence AY036118	-3.66	0.0001
Camk2n1	calcium/calmodulin-dependent protein kinase II inhibitor 1	-2.19	0.0001
Cox7a2l	cytochrome c oxidase subunit VIIa polypeptide 2-like	-2.77	0.0001
Gm19774	predicted gene, 19774 (Gm19774), mRNA.	-30.91	0.0001
LOC100861642	uncharacterized LOC100861642	-13.46	0.0001
Myl9	myosin, light polypeptide 9, regulatory	-3.22	0.0001
TC1700002099.mm.1		-2.98	0.0001
A130050O07Rik	RIKEN cDNA A130050O07 gene	1.53	0.0002
Aldoa	aldolase A, fructose-bisphosphate	-1.81	0.0002
Gm19886	PREDICTED: predicted gene, 19886 (Gm19886), miscRNA.	-4.59	0.0002
Nbl1	neuroblastoma, suppression of tumorigenicity 1	-1.56	0.0002
Ppp1r14b	protein phosphatase 1, regulatory (inhibitor) subunit 14B	-2.39	0.0002
TC0600002682.mm.1		-2.61	0.0002
TC1200002109.mm.1		2.38	0.0002
TC1700001421.mm.1		1.59	0.0002
Tubg1	tubulin, gamma 1	-1.88	0.0002
4930571O06Rik	RIKEN cDNA 4930571O06 gene	1.67	0.0003
Gm24913	predicted gene, 24913	1.53	0.0003
Gm8202	predicted gene 8202	1.59	0.0003
Scp2-ps2	sterol carrier protein 2, pseudogene 2	-4.17	0.0003
Mir7008	microRNA 7008	1.59	0.0004
Ost4	oligosaccharyltransferase 4 homolog (S. cerevisiae)	-2.7	0.0004
TC0100002914.mm.1		1.69	0.0004
TC0500003670.mm.1		3.07	0.0004
TC1100000536.mm.1		1.52	0.0004
TC1900000003.mm.1		-3.68	0.0004
Ttc9b	tetratricopeptide repeat domain 9B	-2.24	0.0004
Apoa1bp	apolipoprotein A-I binding protein	-3.64	0.0005
Cxcr6	chemokine (C-X-C motif) receptor 6	1.71	0.0005
Gm12225	predicted gene 12225	1.64	0.0005
Gm5555	predicted pseudogene 5555	-3.35	0.0005
TC0200002881.mm.1		1.57	0.0005
TC0300000427.mm.1		-1.52	0.0005
TC0500002864.mm.1		1.59	0.0005

TC0800000644.mm.1		1.67	0.0006
TC0100000555.mm.1		-16.33	0.0006
Ik	IK cytokine	-3.38	0.0006
Jag1	jagged 1	-2.15	0.0006
TC0X00000604.mm.1		1.74	0.0006
TC0200003021.mm.1		-2.43	0.0007
TC0500002144.mm.1		3.74	0.0007
TC1400000180.mm.1		1.63	0.0007
Gm26022	predicted gene, 26022	2.27	0.0007
Gm26210	predicted gene, 26210	1.98	0.0007
LOC100862392	PREDICTED: uncharacterized LOC100862392 (LOC100862392), miscRNA.	-2.22	0.0007
Myh11	myosin, heavy polypeptide 11, smooth muscle	-2.14	0.0007
Pcyox1	prenylcysteine oxidase 1	-1.8	0.0007
TC1100003981.mm.1		-2.96	0.0008
Gm11681	predicted gene 11681	1.57	0.0008
Gm13189; RP23-416H10.2	predicted gene 13189	3.62	0.0008
Gm13495	predicted gene 13495	1.7	0.0008
Gm9825	predicted gene 9825	-1.51	0.0008
TC1300001711.mm.1		1.8	0.0009
TC1900000579.mm.1		1.5	0.0009
TC1700002371.mm.1		1.65	0.0009
Abhd3	abhydrolase domain containing 3	-2.27	0.0009
Gm16121; AC145748.2	predicted gene 16121	1.6	0.0009
Ndst1	N-deacetylase/N-sulfotransferase (heparan glucosaminyl) 1	-1.55	0.0009
Triqk	triple QxxK/R motif containing	-2.01	0.0009
TC1000002912.mm.1		2.31	0.001
TC1200001378.mm.1		2.15	0.001
1700040F17Rik	PREDICTED: RIKEN cDNA 1700040F17 gene (1700040F17Rik), miscRNA.	1.85	0.001
Gm16209	predicted gene 16209	-2.26	0.001
Gm22203	predicted gene, 22203	1.56	0.001
TC0600002836.mm.1		-2.11	0.0011
TC0800002019.mm.1		-6.21	0.0011
TC0500003017.mm.1		-2.43	0.0011
TC0500002941.mm.1		1.87	0.0011
Copb2	coatomer protein complex, subunit beta 2 (beta prime)	-2.37	0.0011

Cox6a1	cytochrome c oxidase subunit VIa polypeptide 1	-3.48	0.0011
TC0800000894.mm.1		1.65	0.0012
TC1500001804.mm.1		1.67	0.0012
TC0900002903.mm.1		1.99	0.0012
TC0X00001804.mm.1		2.16	0.0012
TC0X00002864.mm.1		2.16	0.0012
TC1100001801.mm.1		2.16	0.0012
Gm12251	predicted gene 12251	-8.48	0.0012
Gm13590	predicted gene 13590	1.52	0.0012
Gm26020	predicted gene, 26020	1.53	0.0012
LOC100862094	PREDICTED: uncharacterized LOC100862094 (LOC100862094), miscRNA.	-4.14	0.0012
Olfr113	olfactory receptor 113	2.08	0.0012
TC1100003903.mm.1		1.62	0.0013
Ighv1-43	immunoglobulin heavy variable V1-43	1.55	0.0013
Snord16a	small nucleolar RNA, C/D box 16A	-4.07	0.0013
TC0300001068.mm.1		1.7	0.0014
Gm25418	predicted gene, 25418	1.74	0.0014
Gm15590; Gm22774	predicted gene 15590	-3.61	0.0015
Gm6023	predicted gene 6023	-2.34	0.0015
Tgfb3	transforming growth factor, beta 3	-2.28	0.0015
TC1300000270.mm.1		1.55	0.0016
TC1900000316.mm.1		1.96	0.0016
TC0400003261.mm.1		-3.21	0.0016
Gm11848	predicted gene 11848	1.54	0.0016
Gm4953	predicted pseudogene 4953	-2.46	0.0016
TC0200003718.mm.1		2.28	0.0017
TC1200000705.mm.1		1.73	0.0017
Baalc	brain and acute leukemia, cytoplasmic	-1.77	0.0017
TC0500002367.mm.1		-3.55	0.0018
Gm19595	PREDICTED: predicted gene, 19595 (Gm19595), miscRNA.	-13.47	0.0018
Gtpbp4-ps3	GTP binding protein 4, pseudogene 3	2.71	0.0018
Olfr1282	olfactory receptor 1282	1.66	0.0018
Rplp0	ribosomal protein, large, P0	-2.33	0.0018
Tas2r130	taste receptor, type 2, member 130	1.55	0.0018
Mrps16	mitochondrial ribosomal protein S16	-1.57	0.0019
Trav12-2	T cell receptor alpha variable 12-2	1.6	0.0019
Vmn2r-ps126	vomeronasal 2, receptor, pseudogene 126	1.6	0.0019
TC0400003277.mm.1		1.51	0.002
Gm11478	predicted gene 11478	-16.11	0.002

Gm20014	PREDICTED: predicted gene, 20014, transcript variant 1 (Gm20014), miscRNA.	-1.51	0.002
LOC100861862	PREDICTED: uncharacterized LOC100861862 (LOC100861862), miscRNA.	-3.29	0.002
Tm7sf3	transmembrane 7 superfamily member 3	-1.61	0.002
TC1000001008.mm.1		1.59	0.0021
TC1300000252.mm.1		1.54	0.0021
TC1100001822.mm.1		-14.51	0.0021
TC1600000380.mm.1		-3.74	0.0021
Gm8420	predicted gene 8420	-3	0.0021
LOC100861675	PREDICTED: uncharacterized LOC100861675 (LOC100861675), miscRNA.	-2.97	0.0021
TC1300001785.mm.1		-4.63	0.0022
TC0100003086.mm.1		-5.02	0.0022
Gm24400	predicted gene, 24400	-16.58	0.0022
TC0100000122.mm.1		1.7	0.0023
TC1000000985.mm.1		8.53	0.0023
TC1300001031.mm.1		1.55	0.0023
Gm12226	predicted pseudogene 12226	-2.31	0.0023
Gm14165	predicted gene 14165	-2.8	0.0023
Gm9625	predicted gene 9625	-3.85	0.0023
Obox4-ps17	oocyte specific homeobox 4, pseudogene 17	1.6	0.0023
Rarres2	retinoic acid receptor responder (tazarotene induced) 2	-1.79	0.0023
Rpl18-ps2	ribosomal protein L18, pseudogene 2	-7.93	0.0023
TC1700000892.mm.1		1.59	0.0024
TC1700000547.mm.1		2.38	0.0024
Cdh5	cadherin 5	-1.65	0.0024
Gm14450	predicted gene 14450	-2.39	0.0024
Ppp1r15b	protein phosphatase 1, regulatory (inhibitor) subunit 15b	-1.59	0.0024
TC0300001642.mm.1		1.52	0.0025
TC1600000626.mm.1		1.73	0.0025
Arhgdia	Rho GDP dissociation inhibitor (GDI) alpha	-1.67	0.0025
Gm15175	predicted gene 15175	1.82	0.0025
TC1600001435.mm.1		-2.1	0.0026
Atp6v1c1	ATPase, H+ transporting, lysosomal V1 subunit C1	-1.8	0.0026

Gm10808	PREDICTED: predicted gene 10808 (Gm10808), miscRNA.	1.97	0.0026
Gm11336	predicted gene 11336	-2.02	0.0026
Gm19974	PREDICTED: predicted gene, 19974 (Gm19974), miscRNA.	-2.41	0.0026
LOC100862145	PREDICTED: uncharacterized LOC100862145, transcript variant 1 (LOC100862145), miscRNA.	-2.26	0.0026
Pkm	pyruvate kinase, muscle	-1.54	0.0026
Atp2b4; Mir6903	ATPase, Ca++ transporting, plasma membrane 4	-2.85	0.0027
LOC432823	similar to hypothetical protein MGC37588,	-4.47	0.0027
TC1200000543.mm.1		-2.45	0.0028
Gm19933	PREDICTED: predicted gene, 19933, transcript variant 2 (Gm19933), miscRNA.	-4.96	0.0028
March6	membrane-associated ring finger (C3HC4) 6	-1.54	0.0028
Olfr855	olfactory receptor 855	1.51	0.0028
Slc25a18	solute carrier family 25 (mitochondrial carrier), member 18	-1.52	0.0028
TC0700000710.mm.1		1.59	0.0029
TC0X00000185.mm.1		1.6	0.0029
TC1700001287.mm.1		1.86	0.0029
TC0600000833.mm.1		1.81	0.0029
Atp6v0b	ATPase, H+ transporting, lysosomal V0 subunit B	-3.31	0.0029
Gm15224	predicted gene 15224	2.47	0.0029
Gm24628	predicted gene, 24628	1.56	0.0029
Rab24	RAB24, member RAS oncogene family	-3.34	0.0029
TC1400002772.mm.1		1.8	0.0031
TC0700004314.mm.1		1.93	0.0031
Gm26957	predicted gene, 26957	1.61	0.0031
Gm9457	predicted gene 9457	1.63	0.0031
TC0800001957.mm.1		1.81	0.0032
TC0800002159.mm.1		-6.01	0.0032
Gm25956	predicted gene, 25956	1.91	0.0032
Rplp0-ps1	ribosomal protein, large, P0, pseudogene 1	-2.37	0.0032
Ubc; Uba52	ubiquitin C	-4.86	0.0032
Pcna	proliferating cell nuclear antigen	-2.22	0.0033
Pphln1	periphilin 1	-1.69	0.0033
Gm22868	predicted gene, 22868	1.89	0.0034

Gm8730	predicted pseudogene 8730	-5.18	0.0034
n-R5s158	nuclear encoded rRNA 5S 158	1.7	0.0034
TC0700004517.mm.1		1.81	0.0035
Mir384	microRNA 384	1.77	0.0035
2310036O22Rik	RIKEN cDNA 2310036O22 gene	-1.55	0.0036
Uqcrc1	ubiquinol-cytochrome c reductase core protein 1	-2.15	0.0036
TC1200001200.mm.1		1.52	0.0037
TC1200001491.mm.1		1.75	0.0037
TC1900000765.mm.1		-1.52	0.0037
TC1800000248.mm.1		1.58	0.0037
TC1600001652.mm.1		1.9	0.0037
TC1400002592.mm.1		1.51	0.0037
Amd2; Amd1	S-adenosylmethionine decarboxylase 2	-3.8	0.0037
TC0500000143.mm.1		-1.64	0.0038
TC1300002163.mm.1		1.55	0.0038
4930466F19RIK	Protein Gm10592	-2.11	0.0039
Camk4	calcium/calmodulin-dependent protein kinase IV	-2	0.0039
Gm10592	predicted gene 10592	-2.11	0.0039
Gm17361	predicted gene, 17361	1.53	0.0039
Gm17522	predicted gene, 17522	1.53	0.0039
Gm21968	predicted gene, 21968	-2.11	0.0039
Pip5k1c	phosphatidylinositol-4-phosphate 5-kinase, type 1 gamma	-1.62	0.0039
TC0X00000842.mm.1		1.6	0.004
TC1200000643.mm.1		1.54	0.004
Clstn1	calsyntenin 1	-2.02	0.004
Gm15696	predicted gene 15696	-1.69	0.004
Gm24079	predicted gene, 24079	1.69	0.004
Mir692-1	microRNA 692-1	-7.25	0.004
Slc30a10	solute carrier family 30, member 10	-1.69	0.004
TC1200000587.mm.1		1.76	0.0041
TC0300002575.mm.1		1.76	0.0041
Arf1	ADP-ribosylation factor 1	-1.72	0.0041
Gm14656	predicted gene 14656	1.64	0.0041
Gm22220	predicted gene, 22220	-1.58	0.0041
Gm27626	predicted gene, 27626	-29.72	0.0041
Gm7332	predicted gene 7332	-2.09	0.0041
Cdk19	cyclin-dependent kinase 19	-5.91	0.0042
Gm24336	predicted gene, 24336	-2.36	0.0042
Gm26633	predicted gene, 26633	1.56	0.0042

Ndufb7	NADH dehydrogenase (ubiquinone) 1 beta subcomplex, 7	-2.76	0.0042
Agrn	agrin	-1.78	0.0043
TC0200005310.mm.1		1.53	0.0044
Flna	filamin, alpha	-2.48	0.0044
Gm19496	PREDICTED: predicted gene, 19496 (Gm19496), miscRNA.	-15.39	0.0044
Gm20721	predicted gene, 20721	-1.74	0.0044
Ncl	nucleolin	-2.03	0.0044
Uck1	uridine-cytidine kinase 2	-3.86	0.0044
TC0800003205.mm.1		1.75	0.0045
Gm19899	PREDICTED: predicted gene, 19899 (Gm19899), miscRNA.	1.56	0.0045
LOC100862246	PREDICTED: uncharacterized LOC100862246 (LOC100862246), miscRNA.	-4.67	0.0045
Vmn1r27	vomeronasal 1 receptor 27	1.53	0.0045
TC0100000881.mm.1		1.58	0.0046
TC1700001452.mm.1		2.17	0.0046
Cd248	CD248 antigen, endosialin	-2.55	0.0046
Gm23119	predicted gene, 23119	-4.67	0.0046
TC0700004096.mm.1		-3.84	0.0047
Dctn2	dynactin 2	-1.95	0.0047
TC0700000477.mm.1		1.56	0.0048
TC1400001758.mm.1		-1.63	0.0048
Gm7866	predicted gene 7866	-1.62	0.0048
TC1700001387.mm.1		-5.88	0.0049
TC1400001082.mm.1		1.99	0.0049
Eef2	eukaryotic translation elongation factor 2	-2.42	0.0049
Wdr74	WD repeat domain 74	-2.17	0.0049
TC1700001404.mm.1		1.78	0.005
TC0800000858.mm.1		1.84	0.005
TC0500003228.mm.1		1.65	0.005
TC1900000391.mm.1		1.6	0.005
Gm25798	predicted gene, 25798	3.06	0.005
Pdxp	pyridoxal (pyridoxine, vitamin B6) phosphatase	-1.65	0.005
Vmn1r42	vomeronasal 1 receptor 42	1.64	0.005
TC0200001598.mm.1		1.6	0.0051
Fam205a1	family with sequence similarity 205, member A1	-1.57	0.0051
Gm25224	predicted gene, 25224	2.62	0.0051

Gm26723; LOC100861669	predicted gene, 26723	-6	0.0051
Krtap27-1	keratin associated protein 27-1	1.53	0.0051
Rnf187	ring finger protein 187	-2.06	0.0051
TC0X00002722.mm.1		1.57	0.0052
Gm22947	predicted gene, 22947	1.54	0.0052
Hist1h2af	histone cluster 1, H2af	-4.28	0.0052
Hist1h2aj	histone cluster 1, H2aj	-7.28	0.0052
TC1300001307.mm.1		1.59	0.0053
Commd3	COMM domain containing 3	-2.01	0.0053
TC1900000632.mm.1		1.67	0.0054
Gm22289	predicted gene, 22289	-18.92	0.0054
Ndufs3	NADH dehydrogenase (ubiquinone) Fe-S protein 3	-2.77	0.0054
Plpp3	phospholipid phosphatase 3	-2.45	0.0054
Gm12179	predicted gene 12179	1.55	0.0055
Mir5115	microRNA 5115 (Mir5115), microRNA.	-5.84	0.0055
Mir7058	microRNA 7058	1.69	0.0055
Psmb5; Mir686	proteasome (prosome, macropain) subunit, beta type 5	-4.28	0.0055
TC1900001356.mm.1		1.78	0.0056
TC1700002544.mm.1		1.5	0.0056
Cox5b	cytochrome c oxidase subunit Vb	-3.69	0.0057
Ptprb	protein tyrosine phosphatase, receptor type, B	-3.12	0.0057
Atp1a1	ATPase, Na+/K+ transporting, alpha 1 polypeptide	-3.11	0.0058
Gm12726	predicted gene 12726	-2.59	0.0058
Gm6969	predicted pseudogene 6969	-3.64	0.0058
TC1700000561.mm.1		3	0.0059
TC0800001008.mm.1		2.04	0.0059
TC0600003187.mm.1		-2.06	0.0059
Dnajc19	DnaJ (Hsp40) homolog, subfamily C, member 19	-2.4	0.0059
Gm23607	predicted gene, 23607	1.53	0.0059
Mir6236	microRNA 6236	-1.94	0.0059
TC0500002530.mm.1		-1.78	0.006
TC1300000114.mm.1		-5.73	0.006
4921524J17Rik	RIKEN cDNA 4921524J17 gene	-1.87	0.006
Ndufv3	NADH dehydrogenase (ubiquinone) flavoprotein 3	-1.82	0.006
TC0600002933.mm.1		-1.51	0.0061
Cyr61	cysteine rich protein 61	-1.86	0.0061

Gm19660	PREDICTED: predicted gene, 19660, transcript variant 1 (Gm19660), miscRNA.	-3.26	0.0061
Psma6	proteasome (prosome, macropain) subunit, alpha type 6	-2.2	0.0061
TC0600000156.mm.1		1.67	0.0062
Gm11633	predicted gene 11633	-1.64	0.0062
Gm14876	predicted gene 14876	1.95	0.0062
TC1500000270.mm.1		2.61	0.0063
TC1500000911.mm.1		1.85	0.0063
TC1800000844.mm.1		-5	0.0063
4930555M17Rik; RP24-309A12.1	RIKEN cDNA 4930555M17 gene	1.67	0.0063
Mir199a-1	microRNA 199a-1	1.59	0.0063
Ppa1	pyrophosphatase (inorganic) 1	-2.61	0.0063
TC0600001127.mm.1		1.79	0.0064
TC0300002644.mm.1		2.05	0.0064
TC0900002141.mm.1		1.59	0.0064
TC1900000803.mm.1		-1.95	0.0064
Gm12955	predicted gene 12955	1.62	0.0064
Gm23154	predicted gene, 23154	-9.99	0.0064
Gm9637	predicted gene 9637	1.53	0.0064
TC1100002627.mm.1		1.77	0.0065
TC0900001286.mm.1		2.97	0.0065
Astx3	amplified spermatogenic transcripts X encoded 3	1.53	0.0065
Krtap28-13	PREDICTED: keratin associated protein 28-13 (Krtap28-13), mRNA.	1.51	0.0065
Mertk	c-mer proto-oncogene tyrosine kinase	-1.53	0.0065
TC1100003309.mm.1		1.65	0.0066
Gm10406	predicted gene 10406	1.67	0.0066
Gm19270	PREDICTED: predicted gene, 19270 (Gm19270), miscRNA.	1.93	0.0066
TC0300001773.mm.1		1.9	0.0067
TC1100001857.mm.1		1.52	0.0067
TC0300000793.mm.1		1.61	0.0067
TC0500001812.mm.1		-2.21	0.0067
Atp6ap2	ATPase, H+ transporting, lysosomal accessory protein 2	-2.66	0.0067
Gm20349	PREDICTED: predicted gene, 20349 (Gm20349), miscRNA.	-3.44	0.0067
Gm23069	predicted gene, 23069	1.74	0.0067
Vamp2	vesicle-associated membrane protein 2	-1.83	0.0067

TC0100002363.mm.1		1.74	0.0068
Polr2f	polymerase (RNA) II (DNA directed) polypeptide F	-3.25	0.0068
TC1900000145.mm.1		-4.08	0.0069
Gstp1	glutathione S-transferase, pi 1	-2.14	0.0069
Hist1h2an	histone cluster 1, H2an	-5.89	0.0069
TC1600000757.mm.1		-8.01	0.007
TC0X00003111.mm.1		-2.96	0.007
Gm19935	PREDICTED: predicted gene, 19935 (Gm19935), miscRNA.	2.28	0.007
TC0400002399.mm.1		1.54	0.0071
TC0200004398.mm.1		1.5	0.0071
Ccdc169	coiled-coil domain containing 169	1.58	0.0071
Cdc42ep1	CDC42 effector protein (Rho GTPase binding) 1	-1.83	0.0071
TC0700004211.mm.1		1.53	0.0072
Gm14537	predicted gene 14537	3.63	0.0072
Ctsb	cathepsin B	-2.91	0.0073
Gm22730	predicted gene, 22730	3.43	0.0074
Kif5a	kinesin family member 5A	-5.41	0.0074
Uck2	uridine-cytidine kinase 2	-2.81	0.0074
TC0200004977.mm.1		1.52	0.0075
TC1900000853.mm.1		1.92	0.0075
Cnot6	CCR4-NOT transcription complex, subunit 6	-1.65	0.0075
Gm16084; RP23-80I4.3	predicted gene 16084	1.53	0.0075
Gm1866	predicted gene 1866	-3.3	0.0075
Gm7497	predicted gene 7497	-2.99	0.0075
TC1900001403.mm.1		1.85	0.0076
TC0200003609.mm.1		1.58	0.0077
TC1900000146.mm.1		-2.2	0.0077
Eogt	EGF domain-specific O-linked N-acetylglucosamine (GlcNAc) transferase	-1.65	0.0077
Gm11270	predicted gene 11270	-1.59	0.0077
Gm12852	predicted gene 12852	-1.87	0.0077
Gm25226	predicted gene, 25226	1.66	0.0077
Gm26455	predicted gene, 26455	1.69	0.0077
Gm2974	predicted gene 2974	1.63	0.0077
Olfr159	olfactory receptor 159	1.54	0.0077
Stk39	serine/threonine kinase 39	-2.72	0.0077
TC0800001046.mm.1		-2.22	0.0079
TC0400001061.mm.1		-2.61	0.0079
TC0300001613.mm.1		2.22	0.0079

TC0100000025.mm.1		1.54	0.0079
Gm19474	PREDICTED: predicted gene, 19474, transcript variant 2 (Gm19474), miscRNA.	-4.78	0.0079
Gm20417; RP24-458F14.3	predicted gene 20417	-3.63	0.0079
Gm9103	predicted gene 9103	-1.73	0.0079
Ighv10-1	immunoglobulin heavy variable 10-1	2.16	0.0079
Sh3bgrl3	SH3 domain binding glutamic acid-rich protein-like 3	-1.58	0.0079
Sipa1l1	signal-induced proliferation-associated 1 like 1	-2.49	0.0079
Slc38a3	solute carrier family 38, member 3	-1.85	0.0079
TC0200002252.mm.1		1.54	0.008
TC0200004859.mm.1		1.54	0.008
TC0200004870.mm.1		1.54	0.008
TC0100000104.mm.1		-2.95	0.008
Gm5239	predicted pseudogene 5239	-4.63	0.008
TC0500000674.mm.1		1.68	0.0081
Gm12034	predicted gene 12034	-3.34	0.0081
TC1100001865.mm.1		1.8	0.0082
TC1200000195.mm.1		-13.08	0.0082
Gm13981	predicted gene 13981	-1.66	0.0082
Gm25539	predicted gene, 25539	1.5	0.0082
Gm22201	predicted gene, 22201	1.61	0.0083
Gm26308	predicted gene, 26308	2.22	0.0083
Sdha	succinate dehydrogenase complex, subunit A, flavoprotein (Fp)	-2.45	0.0083
Sepw1	selenoprotein W, muscle 1	-3.08	0.0083
Wwp1	WW domain containing E3 ubiquitin protein ligase 1	-1.85	0.0083
TC1000002186.mm.1		1.55	0.0084
Gm22553	predicted gene, 22553	1.57	0.0084
Nfe2l1	nuclear factor, erythroid derived 2,-like 1	-1.64	0.0084
Polr2j	polymerase (RNA) II (DNA directed) polypeptide J	-1.83	0.0084
Rs5-8s1	5.8S ribosomal RNA	-4.05	0.0084
TC0200003580.mm.1		1.54	0.0085
Gm23081	predicted gene, 23081	1.58	0.0085
Gm23219	predicted gene, 23219	1.82	0.0085
TC1500001950.mm.1		-2.49	0.0086
Brms1l	breast cancer metastasis-suppressor 1-like	-2.03	0.0086
Rab21	RAB21, member RAS oncogene family	-1.93	0.0086

Vmn1r209	vomeronasal 1 receptor 209	1.8	0.0086
TC0700004424.mm.1		1.74	0.0087
6230426M11Rik	PREDICTED: RIKEN cDNA 6230426M11 gene (6230426M11Rik), miscRNA.	1.59	0.0087
D5Ertd683e	PREDICTED: DNA segment, Chr 5, ERATO Doi 683, expressed (D5Ertd683e), miscRNA.	2.06	0.0087
Gm11836	predicted gene 11836	-4.49	0.0088
Gm12341	predicted gene 12341	-3.45	0.0088
Rpl17-ps1	ribosomal protein L17, pseudogene 1	-3.46	0.0088
2410015M20Rik	RIKEN cDNA 2410015M20 gene	-2.12	0.0089
Gm14706	predicted gene 14706	-1.66	0.0089
Gm20675; RP23-177N16.1	predicted gene 20675	1.83	0.0089
Gm21809	predicted gene, 21809	1.51	0.0089
Ndufs6	NADH dehydrogenase (ubiquinone) Fe-S protein 6	-3	0.0089
TC0800001072.mm.1		4.91	0.009
Gm22211	predicted gene, 22211	2	0.009
Gm7381	predicted gene 7381	-5.73	0.009
Mcam	melanoma cell adhesion molecule	-1.92	0.009
Mir467c	microRNA 467c	-4.95	0.009
Sv2b	synaptic vesicle glycoprotein 2 b	-3.01	0.009
TC0900000547.mm.1		-1.84	0.0091
Atl1; Gm3086	atlastin GTPase 1	-1.61	0.0091
Gm19831	predicted gene, 19831, transcript variant 1 (Gm19831), miscRNA.	-7.19	0.0092
Gm23066	predicted gene, 23066	1.72	0.0092
Mir6917	microRNA 6917	3.01	0.0092
Mirlet7a-1	microRNA let7a-1	2.46	0.0092
Ndufa1	NADH dehydrogenase (ubiquinone) 1 alpha subcomplex, 1	-13.19	0.0092
Rpl9-ps1	ribosomal protein L9, pseudogene 1	-2.58	0.0092
TC0X00002175.mm.1		1.55	0.0093
TC0500003353.mm.1		-2.49	0.0093
TC1800000713.mm.1		1.53	0.0093
Krtap19-3	keratin associated protein 19-3	1.63	0.0093
TC0100000695.mm.1		1.58	0.0094
TC1200001052.mm.1		1.54	0.0094
Clybl	citrate lyase beta like	-1.52	0.0094
LOC100503279	uncharacterized LOC100503279 (LOC100503279), miscRNA.	-4.71	0.0094

LOC100862193	uncharacterized LOC100862193 (LOC100862193), miscRNA.	-2.16	0.0094
Ndufa9	NADH dehydrogenase (ubiquinone) 1 alpha subcomplex, 9	-3.13	0.0094
TC1700002252.mm.1		1.71	0.0095
Gm12753; RP23-469G19.2	predicted gene 12753	1.52	0.0095
TC0100002643.mm.1		1.51	0.0096
TC1000002133.mm.1		1.66	0.0096
6030471H07Rik; RP23-471O23.1	RIKEN cDNA 6030471H07 gene	1.55	0.0096
Rpl14-ps1	ribosomal protein L14, pseudogene 1	-3.9	0.0096
Bcan	brevican	-1.63	0.0097
Gm21614	predicted gene, 21614	1.58	0.0097
Gm22748	predicted gene, 22748	-16.42	0.0097
TC0200000414.mm.1		1.63	0.0098
TC0800002784.mm.1		-1.58	0.0098
TC0200000890.mm.1		-1.63	0.0098
Gm17267	predicted gene, 17267	1.57	0.0098
Gm26288	predicted gene, 26288	1.65	0.0098
Gm5941	predicted gene 5941	1.63	0.0098
LOC100862257	PREDICTED: uncharacterized LOC100862257 (LOC100862257), miscRNA.	-3.51	0.0098
Pitpnb	phosphatidylinositol transfer protein, beta	-2.11	0.0098
TC0100002733.mm.1		1.69	0.0099
Anp32b-ps1	Bacidic (leucine-rich) nuclear phosphoprotein 32 family, member B, pseudogene 1	-1.83	0.0099
Dnttip1	deoxynucleotidyltransferase, terminal, interacting protein 1	-1.84	0.0099
Lonp2	lon peptidase 2, peroxisomal	-2.26	0.0099
Tecr	trans-2,3-enoyl-CoA reductase	-2.93	0.0099
TC1700001149.mm.1		1.64	0.01
TC0900000843.mm.1		2	0.01
TC0200001493.mm.1		-2.5	0.01
TC1500001311.mm.1		1.54	0.01
Gm11510	predicted gene 11510	-2.36	0.01
Gm13298; Fam205a4; Gm20938	predicted gene 13298 (Gm13298), mRNA.	-2.21	0.01
Hist1h2al	histone cluster 1, H2al	-3.45	0.01
Ilf2	interleukin enhancer binding factor 2	-1.61	0.01
Rnf4	ring finger protein 4	-1.87	0.01
TC1900000553.mm.1		1.57	0.0101

TC0900002148.mm.1		-4.43	0.0101
Gm25051	predicted gene, 25051	1.51	0.0101
LOC100862154	uncharacterized LOC100862154 (LOC100862154), miscRNA.	-1.51	0.0101
TC0800002360.mm.1		-1.7	0.0102
Gm13573	predicted gene 13573	-2.33	0.0102
TC0500000743.mm.1		1.66	0.0103
TC0300002213.mm.1		2.08	0.0103
Gm16580	predicted gene 16580	-9.24	0.0103
Gm23554	predicted gene, 23554	1.66	0.0103
Rps15	ribosomal protein S15	-1.71	0.0103
Snord59a	small nucleolar RNA, C/D box 59A	1.59	0.0103
TC1000001603.mm.1		-2.55	0.0104
TC0500003091.mm.1		1.79	0.0104
TC0300003044.mm.1		1.64	0.0104
Cdr1	cerebellar degeneration related antigen 1	-8.53	0.0104
Gm6630	predicted pseudogene 6630	-1.57	0.0104
Ypel3	yippee-like 3 (Drosophila)	-1.51	0.0104
TC1500001303.mm.1		1.99	0.0105
Kcna1	potassium voltage-gated channel, shaker-related subfamily, member 1	-3.66	0.0105
Lrp1	low density lipoprotein receptor-related protein 1	-1.61	0.0105
TC1900000385.mm.1		3.03	0.0106
Gm15487; Atp6v0c	predicted gene 15487	-4.61	0.0106
Gm26951; RP23-114E15.10	predicted gene, 26951	-1.93	0.0106
Gm5321	predicted gene 5321	-2.28	0.0106
TC1600002071.mm.1		1.59	0.0107
TC0400002552.mm.1		1.57	0.0107
Arpp19	cAMP-regulated phosphoprotein 19	-1.82	0.0107
Gm14407	predicted gene 14407	-1.76	0.0107
TC1700001041.mm.1		1.66	0.0108
TC0600000891.mm.1		1.56	0.0108
Prpf18	PRP18 pre-mRNA processing factor 18 homolog (yeast)	-2.01	0.0108
TC0200004093.mm.1		-8.8	0.0109
Gm24814	predicted gene, 24814	-1.5	0.0109
Gm27740	predicted gene, 27740	1.83	0.0109
Gm27775	predicted gene, 27775	1.83	0.0109
Orc4	origin recognition complex, subunit 4	-1.5	0.0109
TC1500000708.mm.1		-2.43	0.011
TC1800001291.mm.1		1.61	0.011

Gm12091	predicted gene 12091	-2.09	0.011
Gm2897	predicted gene 2897 (Gm2897), transcript variant 1, mRNA.	1.71	0.011
Smim13	small integral membrane protein 13	-1.64	0.011
TC1300000039.mm.1		1.81	0.0111
Atp5b	ATP synthase, H+ transporting mitochondrial F1 complex, beta subunit	-2.94	0.0111
Smarcd1	SWI/SNF related, matrix associated, actin dependent regulator of chromatin, subfamily d, member 1	-1.5	0.0111
TC1700002236.mm.1		1.51	0.0112
Gm10171	predicted gene 10171	-1.83	0.0112
Higd2a	HIG1 domain family, member 2A	-8.94	0.0112
LOC100862384	uncharacterized LOC100862384 (LOC100862384), miscRNA.	-1.95	0.0112
TC0500001040.mm.1		-4.47	0.0113
Gm8062	predicted pseudogene 8062	-3.07	0.0113
Ndufs4	NADH dehydrogenase (ubiquinone) Fe-S protein 4	-4.49	0.0113
TC1600001480.mm.1		-3.91	0.0114
TC1400000449.mm.1		1.79	0.0114
TC0700001089.mm.1		2.02	0.0114
TC1500001500.mm.1		2.42	0.0114
TC1400002775.mm.1		-3.42	0.0114
TC0X00001258.mm.1		2.05	0.0115
TC1300001311.mm.1		-1.75	0.0115
TC0700004375.mm.1		-2.85	0.0115
Ighv1-63	immunoglobulin heavy variable V1-63	6.19	0.0115
Rps4x	ribosomal protein S4, X-linked	-4.37	0.0115
TC0500003033.mm.1		1.9	0.0116
TC1000002026.mm.1		1.65	0.0116
TC1500000003.mm.1		-1.54	0.0116
Gm13295	predicted gene 13295	2.17	0.0116
Gm23880	predicted gene, 23880	2.17	0.0116
Smdt1	single-pass membrane protein with aspartate rich tail 1	-8.99	0.0116
TC0300002641.mm.1		1.57	0.0117
Gm19976	PREDICTED: predicted gene, 19976, transcript variant 6 (Gm19976), miscRNA.	-4.32	0.0117
Gm7808	predicted pseudogene 7808	-2.84	0.0117
TC0200002061.mm.1		-2.49	0.0118
Gm17541	predicted gene, 17541	-3.86	0.0118

Gm26826	predicted gene, 26826	2.16	0.0118
Gm4596	predicted gene 4596	-2.47	0.0118
TC0X00001298.mm.1		1.52	0.0119
Gm24255	predicted gene, 24255	1.79	0.0119
Hba-a2; Hba-a1	hemoglobin alpha, adult chain 2	-4.08	0.0119
TC0200002802.mm.1		-4.45	0.012
Crbn	cereblon	-2.09	0.012
Gm14944; RP23-302B23.2	predicted gene 14944	-2.18	0.012
TC0X00002607.mm.1		1.53	0.0121
Akr1a1	aldo-keto reductase family 1, member A1 (aldehyde reductase)	-2.92	0.0121
Actb	actin, beta	-3.52	0.0122
Gm23300	predicted gene, 23300	-1.62	0.0122
Gm24238	predicted gene, 24238	1.73	0.0122
Gm25630	predicted gene, 25630	1.71	0.0122
Kctd13	potassium channel tetramerisation domain containing 13	-1.74	0.0122
TC0600003377.mm.1		1.57	0.0123
TC0100002334.mm.1		1.6	0.0123
TC0Y00000376.mm.1		1.51	0.0123
TC1100001387.mm.1		1.56	0.0123
Dap3	death associated protein 3	-1.77	0.0123
TC1800001108.mm.1		1.52	0.0124
TC0200001416.mm.1		3.09	0.0124
Tgoln1; Tgoln2	trans-golgi network protein	-2.98	0.0124
Vamp5	vesicle-associated membrane protein 5	-1.65	0.0124
Gm22527	predicted gene, 22527	2.71	0.0125
Gm22809	predicted gene, 22809	1.57	0.0125
LOC100861833	PREDICTED: uncharacterized LOC100861833 (LOC100861833), miscRNA.	-3.59	0.0125
Mir875	microRNA 875	1.55	0.0125
Rbx1	ring-box 1	-4.2	0.0125
Slc38a11	solute carrier family 38, member 11	-2.11	0.0125
Snora34; Mir1291	small nucleolar RNA, H/ACA box 34	-4.5	0.0125
TC0X00002951.mm.1		1.65	0.0126
Gm13171	predicted gene 13171	-1.64	0.0126
Gm19738	PREDICTED: predicted gene, 19738, transcript variant 1 (Gm19738), miscRNA.	-3.09	0.0126

LOC100862313	PREDICTED: uncharacterized LOC100862313, transcript variant 1 (LOC100862313), miscRNA.	-5.63	0.0126
Mir7k	microRNA 7k	1.65	0.0126
TC0400002938.mm.1		1.65	0.0127
Cask	calcium/calmodulin-dependent serine protein kinase (MAGUK family)	-1.59	0.0127
Gm21803	predicted gene, 21803	1.53	0.0127
Gm6104	predicted gene 6104	-2.86	0.0127
Olfr383-ps1	olfactory receptor 383, pseudogene 1	1.54	0.0127
Olfr509	olfactory receptor 509	1.94	0.0127
Olfr948	olfactory receptor 948	1.74	0.0127
TC1600001933.mm.1		1.65	0.0128
TC0300000474.mm.1		1.51	0.0128
Chmp1b	charged multivesicular body protein 1B	-1.54	0.0128
Gm15414	predicted gene 15414	1.51	0.0128
Gm22751; Gm12164	predicted gene, 22751	-2.25	0.0128
Gm26557	predicted gene, 26557	1.82	0.0128
Maf	avian musculoaponeurotic fibrosarcoma (v-maf) AS42 oncogene homolog	-2.11	0.0128
Rpl18	ribosomal protein L18	-1.62	0.0128
TC1000002856.mm.1		1.69	0.0129
Eif5a	eukaryotic translation initiation factor 5A	-1.57	0.0129
TC1300000962.mm.1		-82.14	0.013
Cct2	chaperonin containing Tcp1, subunit 2 (beta)	-1.7	0.013
D8Ertd738e	DNA segment, Chr 8, ERATO Doi 738, expressed	-5.55	0.013
Gm13680	predicted gene 13680	-15.02	0.013
LOC100861832	PREDICTED: uncharacterized LOC100861832 (LOC100861832), miscRNA.	-2.42	0.013
Necap1	NECAP endocytosis associated 1	-1.55	0.013
TC0400002061.mm.1		1.61	0.0131
TC0X00003024.mm.1		-9.78	0.0131
Clta	clathrin, light polypeptide (Lca)	-1.53	0.0131
Gm20608; RP24-370G23.7	predicted gene 20608	1.57	0.0131
Igfbp7	insulin-like growth factor binding protein 7	-2.37	0.0132
Psmc1	protease (prosome, macropain) 26S subunit, ATPase 1	-2.51	0.0132
TC1300001518.mm.1		1.53	0.0133
TC0300002894.mm.1		2.03	0.0133

E030024N20Rik	RIKEN cDNA E030024N20 gene	-3.03	0.0133
Gm17257	predicted gene, 17257	-2.41	0.0133
Scn1b	sodium channel, voltage-gated, type I, beta	-1.65	0.0134
TC1800000918.mm.1		1.52	0.0135
TC1400002610.mm.1		-2.16	0.0135
TC1200002047.mm.1		1.62	0.0135
Cnn3	calponin 3, acidic	-2.01	0.0135
Gm13298; Fam205a2	predicted gene 13298 (Gm13298), mRNA.	-1.65	0.0135
Gm13298; Fam205a3; Gm21598	predicted gene 13298 (Gm13298), mRNA.	-1.65	0.0135
Gm7134	kinesin family member 18B pseudogene	1.73	0.0135
Ighv5-12-4	Ighv5-12-4 immunoglobulin heavy variable 5-12-4	2.07	0.0135
TC1900000070.mm.1		1.51	0.0136
TC1500001632.mm.1		1.61	0.0136
TC1700000165.mm.1		1.68	0.0136
TC0700003554.mm.1		1.81	0.0136
Rpl36	ribosomal protein L36	-2.09	0.0136
TC1400001526.mm.1		1.58	0.0137
Epm2aip1	EPM2A (laforin) interacting protein 1	-1.69	0.0137
Manbal	mannosidase, beta A, lysosomal-like	-2.19	0.0137
Nell2	NEL-like 2	-2.84	0.0137
TC1700000441.mm.1		2.14	0.0138
TC0500001271.mm.1		1.69	0.0138
Grm3	glutamate receptor, metabotropic 3	-2.41	0.0138
Mir133a-2	microRNA 133a-2	1.63	0.0138
Msl2	male-specific lethal 2 homolog (Drosophila)	-2.8	0.0138
Psmc5	protease (prosome, macropain) 26S subunit, ATPase 5	-3.37	0.0138
Ufd1l	ubiquitin fusion degradation 1 like	-1.62	0.0138
Gm11628	predicted gene 11628	-2.92	0.0139
Gm12117	predicted gene 12117	-1.82	0.0139
Gm12943	predicted gene 12943	-5.51	0.0139
Gm19453	PREDICTED: predicted gene, 19453 (Gm19453), miscRNA.	-9.34	0.0139
Gm25025	predicted gene, 25025	-1.8	0.0139
Gm4735	predicted gene 4735	-6.03	0.0139
Gm12816	predicted gene 12816	-2.76	0.014
Olfr692	olfactory receptor 692	1.7	0.014
TC0700000549.mm.1		-2.8	0.0141

TC0100000132.mm.1		1.93	0.0141
TC0200005212.mm.1		1.78	0.0141
TC1000002913.mm.1		1.94	0.0141
TC0800002202.mm.1		1.78	0.0141
Ddn	dendrin	-2.22	0.0141
Gkn3	gastrokine 3	-2.44	0.0141
Gm14269	predicted gene 14269	-2.68	0.0141
LOC100861805	PREDICTED: uncharacterized LOC100861805 (LOC100861805), miscRNA.	-2.05	0.0141
TC1200000028.mm.1		1.53	0.0142
TC0900000784.mm.1		1.65	0.0142
Drg1	developmentally regulated GTP binding protein 1	-6.08	0.0142
Hba-a2; Hba-a1	hemoglobin alpha, adult chain 2 (Hba-a2), mRNA.	-4.71	0.0142
Mir6916	microRNA 6916	3.21	0.0142
TC0400002068.mm.1		1.54	0.0143
Gm12254	predicted gene 12254	-2.77	0.0143
Gm13352	predicted gene 13352	-4.48	0.0144
Rtf1	Rtf1, Paf1/RNA polymerase II complex component, homolog (S. cerevisiae)	-1.59	0.0144
TC0700003102.mm.1		-2.12	0.0145
Aplp1	amyloid beta (A4) precursor-like protein 1	-1.77	0.0145
Cox5a	cytochrome c oxidase subunit Va	-6.6	0.0145
Actg-ps1; Gm23812	actin, gamma, pseudogene 1	-2.72	0.0146
Gm12892	predicted gene 12892	-1.67	0.0146
Rpl18-ps1	ribosomal protein L18, pseudogene 1	-16.03	0.0146
TC0Y00000526.mm.1		1.53	0.0147
LOC100861882	uncharacterized LOC100861882, transcript variant 1	-9.17	0.0147
Rtfdc1	replication termination factor 2 domain containing 1	-1.56	0.0147
Gm10250	predicted pseudogene 10250	-4.34	0.0148
Mir3092	microRNA 3092	3.75	0.0148
TC1800000439.mm.1		-1.54	0.0149
TC1200000372.mm.1		1.85	0.0149
Fkbp2	FK506 binding protein 2	-1.95	0.0149
Gm11273	predicted gene 11273	-4.04	0.0149
Gm14150	predicted gene 14150	-4.69	0.0149
Gm24348	predicted gene, 24348	1.54	0.015
Mrpl27	mitochondrial ribosomal protein L27	-1.64	0.015

TC0700000093.mm.1		2.32	0.0151
TC0900002946.mm.1		1.97	0.0151
Nlk	nemo like kinase	-1.62	0.0151
Olfr460	olfactory receptor 460	1.53	0.0151
TC0800001782.mm.1		1.56	0.0152
TC0600001651.mm.1		1.52	0.0152
TC0600000208.mm.1		1.66	0.0152
Gm13092	predicted gene 13092	-1.58	0.0152
Gm6055	predicted gene 6055	-1.78	0.0152
Lin7a	lin-7 homolog A (C. elegans)	-2.77	0.0152
Mir7223	microRNA 7223	1.56	0.0152
TC0900002051.mm.1		1.8	0.0153
Bap1	Brca1 associated protein 1	-1.61	0.0153
Gm14411	predicted gene 14411	-3.09	0.0153
TC1500001287.mm.1		1.55	0.0154
TC0600000756.mm.1		1.68	0.0154
Atp1b1	ATPase, Na+/K+ transporting, beta 1 polypeptide	-5.3	0.0154
Clk3	CDC-like kinase 3	-2.53	0.0154
Gm24665	predicted gene, 24665	1.64	0.0154
Olfr813	olfactory receptor 813	1.74	0.0155
Rpl36-ps3	ribosomal protein L36, pseudogene 3	-1.76	0.0155
Gm26244	predicted gene, 26244	-1.54	0.0156
Gm7324	predicted gene 7324	-2.1	0.0156
Large	like-glycosyltransferase	-1.78	0.0156
Obox4-ps18	oocyte specific homeobox 4, pseudogene 18	1.56	0.0156
Obox4-ps19	oocyte specific homeobox 4, pseudogene 19	1.56	0.0156
Obox4-ps20	oocyte specific homeobox 4, pseudogene 20	1.56	0.0156
Obox4-ps21	oocyte specific homeobox 4, pseudogene 21	1.56	0.0156
Obox4-ps22	oocyte specific homeobox 4, pseudogene 22	1.56	0.0156
Prpf8	pre-mRNA processing factor 8	-3.01	0.0156
TC0800001985.mm.1		2.05	0.0157
Gpr37l1	G protein-coupled receptor 37-like 1	-2.03	0.0157
Oxct1	3-oxoacid CoA transferase 1	-1.81	0.0157
TC1600001813.mm.1		1.56	0.0158
Gm5915	predicted pseudogene 5915	-5.9	0.0158
TC0100000405.mm.1		-2.12	0.0159

Atp5g3	ATP synthase, H+ transporting, mitochondrial F0 complex, subunit C3 (subunit 9)	-1.9	0.0159
Cox7a2	cytochrome c oxidase subunit VIIa 2	-32.64	0.0159
Gm12074	predicted gene 12074	-1.96	0.0159
Gm20545	predicted gene 20545	-5.06	0.0159
Olfr676	olfactory receptor 676	1.58	0.0159
Vamp1	vesicle-associated membrane protein 1	-2.73	0.0159
Gm11956	predicted gene 11956	-1.85	0.016
Gm12922	predicted gene 12922	-2.49	0.016
Id1	inhibitor of DNA binding 1	-1.88	0.016
TC0X00001537.mm.1		3.29	0.0161
TC1400000227.mm.1		1.56	0.0161
Rplp1	ribosomal protein, large, P1	-12.14	0.0161
Scap	SREBF chaperone	-1.6	0.0161
TC1300002749.mm.1		1.69	0.0162
Lamb2	laminin, beta 2	-1.51	0.0162
LOC100861930	uncharacterized LOC100861930, transcript variant 1	1.5	0.0162
Tsn	translin	-2	0.0162
Uqcr11	ubiquinol-cytochrome c reductase, complex III subunit XI	-2.64	0.0162
TC0500003363.mm.1		1.77	0.0163
TC1700001735.mm.1		1.71	0.0163
Gm5566	predicted pseudogene 5566	-2.03	0.0163
Gm5921	predicted gene 5921	-3.14	0.0163
Olfr854	olfactory receptor 854	1.62	0.0163
Ppp1r14a	protein phosphatase 1, regulatory (inhibitor) subunit 14A	-2.63	0.0163
TC1500001489.mm.1		-1.61	0.0164
TC0200001814.mm.1		-3.91	0.0164
TC0500001484.mm.1		1.51	0.0165
Gm13822; RP24-297N9.6	predicted gene 13822	1.63	0.0165
Vmn1r3	vomeronasal 1 receptor 3	1.78	0.0165
TC0300000472.mm.1		1.68	0.0166
TC1300000720.mm.1		1.56	0.0166
TC0300000064.mm.1		2.46	0.0166
Atp6v0c; Atp6v0c-ps2	ATPase, H+ transporting, lysosomal V0 subunit C	-1.79	0.0166
Ddrgk1	DDRGK domain containing 1	-1.66	0.0166
Gm25253	predicted gene, 25253	1.87	0.0166
Mrpl42	mitochondrial ribosomal protein L42	-2.23	0.0166

TC1700000023.mm.1		1.61	0.0167
Cct7	chaperonin containing Tcp1, subunit 7 (eta)	-1.63	0.0167
Gm25138	predicted gene, 25138	-3.06	0.0167
Snapc1	small nuclear RNA activating complex, polypeptide 1	-1.5	0.0167
TC0900000250.mm.1		1.69	0.0168
TC1100002151.mm.1		1.59	0.0168
TC1900000187.mm.1		1.6	0.0168
Atp1b2	ATPase, Na+/K+ transporting, beta 2 polypeptide	-1.59	0.0168
Gm25560	predicted gene, 25560	1.51	0.0168
TC1000000965.mm.1		2.33	0.0169
TC0300000493.mm.1		2.09	0.0169
Arl6ip5	ADP-ribosylation factor-like 6 interacting protein 5	-1.91	0.0169
Lancl1	LanC (bacterial lantibiotic synthetase component C)-like 1	-2.18	0.0169
Mir7063	microRNA 7063	2.35	0.0169
Ppib	peptidylprolyl isomerase B	-1.52	0.0169
TC0700001537.mm.1		1.77	0.017
Etnk1	ethanolamine kinase 1	-6.39	0.017
Mir692-3; Ftl1; Ftl2-ps	microRNA 692-3	-3.4	0.017
Pdcd4	programmed cell death 4	-2.81	0.017
TC1300001299.mm.1		-1.58	0.0171
TC0400002736.mm.1		1.6	0.0171
TC1900001043.mm.1		-6.28	0.0171
Ttc9	tetratricopeptide repeat domain 9	-1.79	0.0171
Zcchc9-ps	zinc finger, CCHC domain containing 9, pseudogene	1.69	0.0171
TC1100000455.mm.1		1.53	0.0172
TC1000000777.mm.1		1.69	0.0172
Cyfip2	cytoplasmic FMR1 interacting protein 2	-2.11	0.0172
Dennd6a	DENN/MADD domain containing 6A	-1.69	0.0172
Gm14048	predicted gene 14048	-2.45	0.0172
Gm16073	predicted gene 16073	-1.94	0.0172
Rps2-ps13	ribosomal protein S2, pseudogene 13	-5.21	0.0172
Ap3m1-ps	adaptor-related protein complex 3, mu 1 subunit, pseudogene	-2.66	0.0173
Hnrnpab	heterogeneous nuclear ribonucleoprotein A/B	-1.85	0.0173
Idh3g	isocitrate dehydrogenase 3 (NAD+), gamma	-1.58	0.0173
Pja2	praja 2, RING-H2 motif containing	-3.29	0.0173

Sept3	septin 3	-1.56	0.0173
TC1700002574.mm.1		-2.04	0.0174
Eef1b2	eukaryotic translation elongation factor 1 beta 2	-2.04	0.0175
Gm16418	predicted pseudogene 16418	-3.31	0.0175
Gm3531	predicted pseudogene 3531	-3.1	0.0175
Hspa4	heat shock protein 4	-2.05	0.0175
Srsf7	serine/arginine-rich splicing factor 7	-1.93	0.0175
Trappc11	trafficking protein particle complex 11	-1.76	0.0175
Gm16399	predicted pseudogene 16399	-4.48	0.0176
Kif1b	kinesin family member 1B	-1.8	0.0176
Gatc	glutamyl-tRNA(Gln) amidotransferase, subunit C	-3.53	0.0177
Ndufa13	NADH dehydrogenase (ubiquinone) 1 alpha subcomplex, 13	-5.91	0.0177
Wdr47	WD repeat domain 47	-1.66	0.0177
TC1300001873.mm.1		1.52	0.0178
Sdf2	stromal cell derived factor 2	-1.86	0.0178
Vmn2r-ps74	vomeronasal 2, receptor, pseudogene 74	1.57	0.0178
TC1200000713.mm.1		-2.09	0.0179
TC0600002818.mm.1		-1.64	0.0179
Gm3221	predicted gene 3221	1.53	0.0179
Pcna-ps2	proliferating cell nuclear antigen pseudogene 2	-2.23	0.0179
TC1100001421.mm.1		-3.9	0.018
TC1800000795.mm.1		1.57	0.018
Gm5070	predicted gene 5070	-2.46	0.018
Lpgat1	lysophosphatidylglycerol acyltransferase 1	-1.5	0.018
Mir1941	microRNA 1941	2.11	0.018
TC0200004677.mm.1		-5.65	0.0181
Gm5848	PREDICTED: predicted pseudogene 5848 (Gm5848), mRNA.	-4.26	0.0181
Oaz1; Oaz1-ps; Mir1982	ornithine decarboxylase antizyme 1	-1.7	0.0181
Sf3a3	splicing factor 3a, subunit 3	-1.86	0.0181
TC1800000032.mm.1		1.53	0.0182
4930583P06Rik	RIKEN cDNA 4930583P06 gene	1.71	0.0182
Gm16307	predicted gene 16307	1.68	0.0182
Inmt	indolethylamine N-methyltransferase	-1.66	0.0182
Zfp11	zinc finger protein 11	1.61	0.0182
TC0800000728.mm.1		1.71	0.0183
TC0X00002823.mm.1		1.69	0.0183
Gm12231	predicted gene 12231	-7.18	0.0183

Gm20568	PREDICTED: predicted gene, 20568 (Gm20568), miscRNA.	-3.19	0.0183
Rps15-ps2	ribosomal protein S15, pseudogene 2	-2.08	0.0183
Atl2	atlastin GTPase 2	-2.63	0.0184
C530030P08Rik	RIKEN cDNA C530030P08 gene	-8.37	0.0184
Cd81	CD81 antigen	-4.65	0.0184
Gm26238	predicted gene, 26238	-1.8	0.0184
TC0600002593.mm.1		1.55	0.0185
Gm12204; RP23-263C23.4	predicted gene 12204	1.6	0.0185
Lynx1	Ly6/neurotoxin 1	-1.61	0.0185
TC1600000498.mm.1		1.78	0.0186
Vps28	vacuolar protein sorting 28 (yeast)	-3.19	0.0186
Id2	inhibitor of DNA binding 2	-3.15	0.0187
Rabac1	Rab acceptor 1 (prenylated)	-4.46	0.0187
TC1400002566.mm.1		-2.67	0.0188
Actg1; Mir6935	actin, gamma, cytoplasmic 1	-2.51	0.0188
Gm9174	predicted pseudogene 9174	-3.35	0.0188
Mir6394	microRNA 6394	1.75	0.0188
Polb	polymerase (DNA directed), beta	-2.2	0.0188
Rpl14; Rpl14-ps1	ribosomal protein L14	-3.06	0.0188
Scarb2	scavenger receptor class B, member 2	-1.87	0.0188
TC0100003767.mm.1		-2.56	0.0189
TC0300001724.mm.1		-2.56	0.0189
TC1500001156.mm.1		-2.56	0.0189
TC1500001550.mm.1		-2.56	0.0189
TC0600000399.mm.1		1.93	0.0189
Gm15778	predicted gene 15778	1.77	0.0189
Mrpl48-ps	mitochondrial ribosomal protein L48 pseudogene	-4.36	0.0189
Olfr663	olfactory receptor 663	1.52	0.0189
Tmem30a	transmembrane protein 30A	-3.11	0.0189
Vps4a	vacuolar protein sorting 4a (yeast)	-1.57	0.0189
TC0600002817.mm.1		1.57	0.019
Asah1	N-acylsphingosine amidohydrolase 1	-1.53	0.019
Cdc37l1	cell division cycle 37-like 1	-1.56	0.019
Puf60	poly-U binding splicing factor 60	-1.51	0.019
TC0600003429.mm.1		1.68	0.0191
Kpna3	karyopherin (importin) alpha 3	-1.71	0.0191
Rpl18a-ps1	ribosomal protein L18A, pseudogene 1	-6.14	0.0191
Rpl4	ribosomal protein L4	-13.51	0.0191
TC1800000027.mm.1		1.52	0.0192

Ap3d1	adaptor-related protein complex 3, delta 1 subunit	-1.63	0.0192
Gm27509	predicted gene, 27509	-1.59	0.0192
TC0700001055.mm.1		1.81	0.0193
TC0800001940.mm.1		1.89	0.0193
TC1000002171.mm.1		2.12	0.0193
TC1700001338.mm.1		-3.37	0.0193
Gm10360	predicted gene 10360	1.6	0.0193
Vmn1r205; Vmn1r200	vomeronasal 1 receptor 205	1.56	0.0193
TC0200004781.mm.1		-1.58	0.0194
Gm20463; RP23-24A13.20	predicted gene 20463	1.67	0.0194
Gm25407	predicted gene, 25407	1.8	0.0194
Mgrn1	mahogunin, ring finger 1	-1.84	0.0194
Mir98	microRNA 98	2.44	0.0194
H2afv	H2A histone family, member V	-2	0.0195
Vps29	vacuolar protein sorting 29 (S. pombe)	-1.78	0.0195
TC1300001718.mm.1		-1.57	0.0196
TC0900000757.mm.1		-7.08	0.0196
TC1800000780.mm.1		1.56	0.0196
Hmgn2l6	high-mobility group nucleosomal binding domain 2-like 6	1.58	0.0196
Txn1	thioredoxin 1	-2.67	0.0196
TC0800000334.mm.1		2.97	0.0197
TC0600003359.mm.1		1.53	0.0198
TC0700001449.mm.1		1.52	0.0198
Apbb1	amyloid beta (A4) precursor protein-binding, family B, member 1	-1.59	0.0198
Gm16372	predicted pseudogene 16372	-1.75	0.0198
TC0X00001417.mm.1		1.82	0.0199
TC1900000648.mm.1		-2.8	0.0199
Bloc1s2-ps	biogenesis of lysosome-related organelles complex-1, subunit 2, pseudogene	-1.7	0.0199
Gm14227	predicted gene 14227	-1.66	0.0199
Gm9762	predicted pseudogene 9762	-2.77	0.0199
Ncdn	neurochondrin	-1.57	0.0199
TC0400003735.mm.1		1.84	0.02
C030005K15Rik	RIKEN cDNA C030005K15 gene	1.85	0.02
Gm10131	predicted pseudogene 10131	-9.7	0.02
Olfr700	olfactory receptor 700	1.77	0.02
TC0500003321.mm.1		1.56	0.0201
Gcnt2	glucosaminyl (N-acetyl) transferase 2, I-branching enzyme	-1.82	0.0201

Gm27248	predicted gene 27248	-2.12	0.0201
Ssr2	signal sequence receptor, beta	-3.45	0.0201
Taldo1	transaldolase 1	-1.8	0.0201
Tuba1b	tubulin, alpha 1B	-2.37	0.0201
Gm11539	predicted gene 11539	-3.14	0.0202
TC1100001897.mm.1		5.23	0.0203
Krtap19-9b	keratin associated protein 19-9B	1.56	0.0203
Mapre1	microtubule-associated protein, RP/EB family, member 1	-1.63	0.0203
Rpl15-ps1	ribosomal protein L15, pseudogene 1	-1.79	0.0203
Uchl1	ubiquitin carboxy-terminal hydrolase L1	-4.06	0.0203
1700120E14Rik	RIKEN cDNA 1700120E14 gene	1.63	0.0205
Nr1d1	nuclear receptor subfamily 1, group D, member 1	-1.61	0.0205
TC1500000243.mm.1		1.7	0.0206
Gm9840	predicted gene 9840	-5.41	0.0206
Isca1	iron-sulfur cluster assembly 1 homolog (S. cerevisiae)	-1.51	0.0206
Mir7094-2	microRNA 7094-2	2	0.0206
Ybx1	Y box protein 1	-1.85	0.0206
TC1400002475.mm.1		-1.97	0.0207
Gm10053	predicted gene 10053	-12.56	0.0207
Gm10073	predicted pseudogene 10073	-4.11	0.0207
Mir665	microRNA 665	-1.65	0.0207
Gm5786	predicted pseudogene 5786	-5.57	0.0208
Nctc1	non-coding transcript 1	1.51	0.0208
Rpl9-ps7	ribosomal protein L9, pseudogene 7	-11.48	0.0208
TC0400000399.mm.1		-3.11	0.0209
Gm22154	predicted gene, 22154	-2.22	0.0209
Psmd11	proteasome (prosome, macropain) 26S subunit, non-ATPase, 11	-1.73	0.0209
TC0900002689.mm.1		1.52	0.021
Gm23513	predicted gene, 23513	1.55	0.021
Vmn1r79	vomeronasal 1 receptor 79	1.56	0.021
TC1800001363.mm.1		1.99	0.0211
TC1400000907.mm.1		-2	0.0211
TC0X00000392.mm.1		1.84	0.0211
Dram2	DNA-damage regulated autophagy modulator 2	-2.07	0.0211
Mir7028	microRNA 7028	1.74	0.0211
TC1600001678.mm.1		1.51	0.0212
Gm16100	predicted gene 16100	-1.66	0.0212
Dad1	defender against cell death 1	-1.77	0.0213

Rfxap	regulatory factor X-associated protein	-1.53	0.0213
Eif2b5	eukaryotic translation initiation factor 2B, subunit 5 epsilon	-1.51	0.0214
Gm15427	predicted pseudogene 15427	-12.15	0.0214
Raph1	Ras association (RalGDS/AF-6) and pleckstrin homology domains 1	-1.96	0.0214
Scyl1	SCY1-like 1 (S. cerevisiae)	-1.56	0.0214
TC1300000772.mm.1		1.96	0.0215
0610009B22Rik	RIKEN cDNA 0610009B22 gene	-1.62	0.0215
Npm3-ps1	nucleoplasmin 3, pseudogene 1	-1.98	0.0215
Tceb1	transcription elongation factor B (SIII), polypeptide 1	-26.91	0.0215
TC1000000237.mm.1		3.77	0.0216
TC1700001727.mm.1		1.95	0.0216
TC0500000139.mm.1		1.72	0.0216
Cops3	COP9 (constitutive photomorphogenic) homolog, subunit 3 (Arabidopsis thaliana)	-1.84	0.0216
Gm19494	PREDICTED: predicted gene, 19494, transcript variant 1 (Gm19494), miscRNA.	-10.64	0.0216
Gm24811	predicted gene, 24811	-4.32	0.0216
TC0300002928.mm.1		1.79	0.0217
TC0300002546.mm.1		1.55	0.0217
Ap1g1	adaptor protein complex AP-1, gamma 1 subunit	-1.51	0.0217
Mir142	microRNA 142	2.1	0.0217
Mir692-2	microRNA 692-2	-12.24	0.0217
TC0500000367.mm.1		1.86	0.0218
Cap2	CAP, adenylate cyclase-associated protein, 2 (yeast)	-2.21	0.0218
Rnf11	ring finger protein 11	-1.69	0.0218
Tspan5	tetraspanin 5	-1.52	0.0218
Zxdb	zinc finger, X-linked, duplicated B	-1.56	0.0218
TC1000002131.mm.1		1.53	0.0219
TC0600000313.mm.1		1.59	0.022
Dnajc19-ps	DnaJ (Hsp40) homolog, subfamily C, member 19, pseudogene	-1.86	0.022
Nt5c	5,3-nucleotidase, cytosolic	-1.51	0.022
Olfr1382	olfactory receptor 1382	1.71	0.022
TC0900001735.mm.1		1.67	0.0221
TC1000001757.mm.1		-1.78	0.0221
Ighv7-1	immunoglobulin heavy variable 7-1	1.56	0.0221
Rab6b	RAB6B, member RAS oncogene family	-1.64	0.0221

TC1500001433.mm.1		1.7	0.0222
TC0700004235.mm.1		1.52	0.0222
TC0100000536.mm.1		-1.68	0.0222
Gm23294	predicted gene, 23294	1.61	0.0222
Ano1	anoctamin 1, calcium activated chloride channel	-1.76	0.0223
TC1500000340.mm.1		1.87	0.0224
Gm27722	predicted gene, 27722	3	0.0224
Gm9385	predicted pseudogene 9385	-1.56	0.0224
Romo1	reactive oxygen species modulator 1	-1.6	0.0224
Vmn2r-ps9	vomeronasal 2, receptor, pseudogene 9	1.6	0.0224
TC1100000879.mm.1		1.52	0.0225
LOC100862001	uncharacterized LOC100862001	-2.09	0.0225
Gm14231	predicted gene 14231	-2.48	0.0226
Gm24590	predicted gene, 24590	-2.26	0.0226
Gm4294	predicted gene 4294	-3.29	0.0226
Hist1h1c	histone cluster 1, H1c	-2.88	0.0226
Ifi27	interferon, alpha-inducible protein 27	-2.14	0.0226
Lrrc58	leucine rich repeat containing 58	-2.24	0.0226
Rps6-ps2	ribosomal protein S6, pseudogene 2	-2.16	0.0226
TC0X00000393.mm.1		-1.69	0.0227
TC1100000878.mm.1		1.51	0.0227
Selk	selenoprotein K	-1.72	0.0227
Cd63	CD63 antigen	-2.59	0.0228
Nedd4	neural precursor cell expressed, developmentally down-regulated 4	-2.44	0.0228
TC0200000560.mm.1		-2.05	0.0229
LOC100861649	uncharacterized LOC100861649, transcript variant 1	-1.82	0.0229
LOC100862081	PREDICTED: uncharacterized LOC100862081 (LOC100862081), miscRNA.	-2.59	0.0229
Mir6993	microRNA 6993	2.73	0.0229
Pik3ca	phosphatidylinositol 3-kinase, catalytic, alpha polypeptide	-1.65	0.0229
Acta2	actin, alpha 2, smooth muscle, aorta	-3.15	0.023
Gnai2	guanine nucleotide binding protein (G protein), alpha inhibiting 2	-2.01	0.023
TC0300002130.mm.1		1.61	0.0231
Gm14017	predicted gene 14017	-5.11	0.0231
Gm22794	predicted gene, 22794	1.59	0.0231
Gm5621	predicted gene 5621	-5.72	0.0232
Ptgds	prostaglandin D2 synthase (brain)	-3.21	0.0232

Cox8a	cytochrome c oxidase subunit VIIIa	-3.57	0.0233
Gm13363	predicted gene 13363	-5.93	0.0233
Gm27731	predicted gene, 27731	-2.24	0.0233
Gm5815	predicted pseudogene 5815	-3.95	0.0233
TC0300002990.mm.1		1.75	0.0234
Eif3d	eukaryotic translation initiation factor 3, subunit D	-3.18	0.0234
Oat	ornithine aminotransferase	-1.6	0.0234
TC0500000698.mm.1		1.54	0.0235
TC0X00000265.mm.1		-2.98	0.0235
Gm12933	predicted gene 12933	-1.79	0.0235
Gm24519	predicted gene, 24519	1.8	0.0235
Sgpp2	sphingosine-1-phosphate phosphotase 2	-2.75	0.0235
TC0600003004.mm.1		-2.37	0.0236
Gm3208	predicted gene 3208	1.64	0.0236
Slc6a6	solute carrier family 6 (neurotransmitter transporter, taurine), member 6	-2.28	0.0236
TC1200001291.mm.1		1.51	0.0237
TC0300000779.mm.1		-2.35	0.0237
TC1900001079.mm.1		-1.54	0.0237
Fam174a	family with sequence similarity 174, member A	-2.02	0.0237
LOC100861939	PREDICTED: uncharacterized LOC100861939, transcript variant 1 (LOC100861939), miscRNA.	-1.69	0.0237
LOC100862217	PREDICTED: uncharacterized LOC100862217 (LOC100862217), miscRNA.	1.51	0.0237
TC0800001673.mm.1		-1.73	0.0238
Gm11942	predicted gene 11942	-8.2	0.0238
Gm16199	predicted gene 16199	-16.51	0.0238
Ssr1	signal sequence receptor, alpha	-1.61	0.0238
TC0500002561.mm.1		1.97	0.024
TC0100000680.mm.1		2.01	0.024
TC1200000078.mm.1		1.8	0.0241
Pgam1-ps2	phosphoglycerate mutase 1, pseudogene 2	-1.53	0.0241
TC1800000888.mm.1		1.56	0.0242
Gm12715	predicted gene 12715	-2.12	0.0242
Gm13192	predicted gene 13192	-6.77	0.0242
TC0300003132.mm.1		1.51	0.0243
TC0X00001920.mm.1		-4.86	0.0243
Aldoc	aldolase C, fructose-bisphosphate	-1.93	0.0243
Cox4i1	cytochrome c oxidase subunit IV isoform 1	-1.56	0.0243

Fads3	fatty acid desaturase 3	-1.76	0.0243
Gm25435	predicted gene, 25435	2.27	0.0243
Klf6	Kruppel-like factor 6	-1.51	0.0243
3110035E14Rik	RIKEN cDNA 3110035E14 gene	-3.05	0.0244
Cript	cysteine-rich PDZ-binding protein	-1.93	0.0244
Rbm4	RNA binding motif protein 4	-1.72	0.0244
TC1700002744.mm.1		-5.25	0.0245
TC0400003899.mm.1		1.82	0.0245
Cuta	cutA divalent cation tolerance homolog (E. coli)	-1.53	0.0245
Gpm6a	glycoprotein m6a	-3.65	0.0245
Hnrnpa2b1	heterogeneous nuclear ribonucleoprotein A2/B1	-1.6	0.0245
Slc25a11	solute carrier family 25 (mitochondrial carrier oxoglutarate carrier), member 11	-1.88	0.0245
TC0X00000188.mm.1		1.6	0.0246
Atf2	activating transcription factor 2	-2.1	0.0246
TC0600001570.mm.1		1.57	0.0247
TC0500000827.mm.1		1.53	0.0247
TC0900001581.mm.1		-3.09	0.0247
TC1100001558.mm.1		1.54	0.0247
TC0200003719.mm.1		1.57	0.0247
TC0300001503.mm.1		1.53	0.0247
Rpl10-ps1	ribosomal protein L10, pseudogene 1	-8.9	0.0247
TC0800001283.mm.1		1.79	0.0248
TC1100004086.mm.1		-2.92	0.0249
Ano6	anoctamin 6	-1.61	0.0249
Gm15453	predicted gene 15453	-1.75	0.0249
Gm6444	predicted gene 6444	-3.67	0.0249
LOC100862121	PREDICTED: uncharacterized LOC100862121 (LOC100862121), miscRNA.	-1.55	0.0249
Mir6396	microRNA 6396	1.57	0.0249
Ccnd2	cyclin D2	-2.12	0.025
Gm12497	predicted pseudogene 12497	-1.84	0.025
Gm12821	predicted gene 12821	-1.57	0.025
Gm15028	predicted gene 15028	1.73	0.025
Gm5637	predicted pseudogene 5637	-1.68	0.025
TC1000002221.mm.1		1.62	0.0251
Crip1	cysteine-rich protein 1 (intestinal)	-2.71	0.0251
Ndufv2	NADH dehydrogenase (ubiquinone) flavoprotein 2	-3.36	0.0252

Fis1	fission 1 (mitochondrial outer membrane) homolog (yeast)	-2.35	0.0253
Gm3934	PREDICTED: predicted gene 3934 (Gm3934), mRNA.	-2.38	0.0253
TC0400001114.mm.1		1.58	0.0254
TC0400001488.mm.1		1.5	0.0254
Ap4s1	adaptor-related protein complex AP-4, sigma 1	-1.92	0.0254
Gm10051	predicted pseudogene 10051	-2.57	0.0254
Gm15713	predicted gene 15713	1.74	0.0254
Gm25845	predicted gene, 25845	2.24	0.0254
Kpnb1	karyopherin (importin) beta 1	-1.6	0.0254
Gm11688	predicted gene 11688	-2.3	0.0255
Gm17014	predicted gene 17014	-1.68	0.0255
TC0200003558.mm.1		2.03	0.0256
Gm12183	predicted gene 12183	-1.78	0.0256
Gm20719; RP24-91B1.5	predicted gene 20719	1.53	0.0256
Gm23153	predicted gene, 23153	-2.24	0.0256
Gm26358	predicted gene, 26358	2.09	0.0256
Gstp2	glutathione S-transferase, pi 2	-1.84	0.0256
Laptm4a	lysosomal-associated protein transmembrane 4A	-3.44	0.0256
Rps18-ps1	ribosomal protein S18, pseudogene 1	-3.24	0.0256
Slc25a3	solute carrier family 25 (mitochondrial carrier, phosphate carrier), member 3	-1.89	0.0256
TC0600002436.mm.1		2.16	0.0257
TC1100002289.mm.1		-1.52	0.0257
Gm11810	predicted gene 11810	-4.95	0.0257
Gm22965	predicted gene, 22965	1.82	0.0257
Gm6117; RP23-192D5.2	predicted gene 6117	1.79	0.0257
TC0900003135.mm.1		1.57	0.0258
TC0600002867.mm.1		1.62	0.0258
TC0500000577.mm.1		1.7	0.0258
TC1500001317.mm.1		1.62	0.0258
Atp2b1	ATPase, Ca++ transporting, plasma membrane 1	-1.74	0.0258
Gm14283	predicted gene 14283	1.6	0.0258
Gm24626	predicted gene, 24626	1.55	0.0258
TC0900000102.mm.1		1.51	0.0259
TC1800000905.mm.1		-10.11	0.0259
TC1300000419.mm.1		-1.68	0.0259
Gm20391	predicted gene 20391	1.71	0.0259

Gm22607	predicted gene, 22607	2.08	0.0259
TC0300003060.mm.1		1.93	0.026
TC1800000499.mm.1		1.86	0.026
Gm11450	predicted gene 11450	-8.41	0.026
Gm5943	predicted pseudogene 5943	-1.79	0.026
Gm5970	predicted gene 5970	1.51	0.026
Gpi1	glucose phosphate isomerase 1	-1.74	0.026
LOC100861650	PREDICTED: uncharacterized LOC100861650, transcript variant 1 (LOC100861650), miscRNA.	-2.83	0.026
Peg3os	Peg3 opposite strand	-2.26	0.026
TC0500001339.mm.1		1.63	0.0261
Gm13175; RP23-198D21.1	predicted gene 13175	1.63	0.0261
Gm5514	predicted gene 5514	-7.4	0.0261
TC0400000440.mm.1		2.06	0.0262
Atp1a2	ATPase, Na+/K+ transporting, alpha 2 polypeptide	-2.45	0.0262
Gm16259	predicted gene 16259	-2.08	0.0262
Pcdh9	protocadherin 9	-2.55	0.0262
Sf3b1	splicing factor 3b, subunit 1	-1.91	0.0262
4930548K13Rik	RIKEN cDNA 4930548K13 gene	1.63	0.0263
Bmpr2	bone morphogenetic protein receptor, type II (serine/threonine kinase)	-6.25	0.0263
Cul3	cullin 3	-1.58	0.0263
Gm5777	predicted gene 5777	-2.62	0.0263
Rpl18a	ribosomal protein L18A	-3.75	0.0263
Trf	transferrin	-2.16	0.0263
TC0400002042.mm.1		1.78	0.0264
TC1700000823.mm.1		1.54	0.0264
Gm5451	predicted gene 5451	-10.29	0.0264
Mup-ps26	major urinary protein, pseudogene 26	1.61	0.0264
Pfdn5	prefoldin 5	-6.99	0.0264
Atp5a1	ATP synthase, H+ transporting, mitochondrial F1 complex, alpha subunit 1	-1.9	0.0266
Rpl9-ps4	ribosomal protein L9, pseudogene 4	-10.91	0.0266
Spcs1	signal peptidase complex subunit 1 homolog (S. cerevisiae)	-1.59	0.0266
TC0300002637.mm.1		1.98	0.0267
2900011O08Rik	RIKEN cDNA 2900011O08 gene	-2.35	0.0267
Gm16335	predicted gene 16335	-5.87	0.0267
TC0500002378.mm.1		-1.75	0.0268

Ostc	oligosaccharyltransferase complex subunit	-7.03	0.0268
Rtn4	reticulon 4	-1.58	0.0268
Sort1	sortilin 1	-1.93	0.0268
Gm6065	predicted gene 6065	-2.96	0.0269
TC1100000868.mm.1		2.18	0.027
Atp6v1d	ATPase, H+ transporting, lysosomal V1 subunit D	-4.21	0.027
Myl6	myosin, light polypeptide 6, alkali, smooth muscle and non-muscle	-2.57	0.027
Slc2a1	solute carrier family 2 (facilitated glucose transporter), member 1	-2.41	0.027
TC1100001772.mm.1		1.63	0.0271
TC1700001872.mm.1		1.51	0.0271
Gm10462	predicted gene 10462	-8.46	0.0271
Gm14391; Gm6710	predicted gene 14391 (Gm14391), transcript variant 1, mRNA.	-1.76	0.0271
Gm15557	predicted gene 15557	1.55	0.0271
Gm9794	PREDICTED: predicted pseudogene 9794 (Gm9794), miscRNA.	-5.73	0.0271
Timm13	translocase of inner mitochondrial membrane 13	-1.78	0.0271
Vmn2r-ps6	vomeronasal 2, receptor, pseudogene 6	1.53	0.0271
TC1000001279.mm.1		1.5	0.0272
TC0400003138.mm.1		-1.66	0.0272
Cdc42	cell division cycle 42	-2.34	0.0272
Rap2a	RAS related protein 2a	-1.77	0.0272
Rpl10a-ps1	ribosomal protein L10A, pseudogene 1	-3.18	0.0272
Rpl8	ribosomal protein L8	-4.84	0.0272
Serf2	small EDRK-rich factor 2	-1.61	0.0272
TC1700001073.mm.1		1.58	0.0273
G630093K05Rik	RIKEN cDNA G630093K05 gene	1.62	0.0273
Gm25610	predicted gene, 25610	1.54	0.0273
Gm3261	predicted gene 3261	1.53	0.0273
Ssfa2	sperm specific antigen 2	-2.02	0.0273
Gm13144	predicted gene 13144	-2.14	0.0274
Gm8662	predicted gene 8662	-6.5	0.0274
Rps18-ps3	ribosomal protein S18, pseudogene 3	-4.09	0.0274
Sept11	septin 11	-1.54	0.0274
Gm6206	predicted pseudogene 6206	-2.96	0.0275
TC1900000480.mm.1		1.59	0.0276
TC1600000627.mm.1		3.49	0.0276
TC1900001746.mm.1		1.93	0.0276

4833439L19Rik	RIKEN cDNA 4833439L19 gene	-2.61	0.0276
Gm14665	predicted gene 14665	1.53	0.0276
Irf2bp2	interferon regulatory factor 2 binding protein 2	-1.52	0.0276
Snrpd2	small nuclear ribonucleoprotein D2	-2.95	0.0276
Gm6265	predicted pseudogene 6265	-6.03	0.0277
TC1300002570.mm.1		-1.72	0.0278
Gm19328	PREDICTED: predicted gene, 19328 (Gm19328), miscRNA.	-2.13	0.0278
Gm5406	predicted gene 5406	-2.11	0.0278
Olfr1004-ps1	olfactory receptor 1004, pseudogene 1	1.55	0.0278
Olfr324	olfactory receptor 324	1.52	0.0278
TC0600002483.mm.1		1.8	0.0279
TC0700000230.mm.1		1.73	0.0279
TC1000001899.mm.1		1.59	0.0279
Gm15289	predicted gene 15289	-1.67	0.0279
Gm3617	predicted gene 3617	-3.5	0.0279
Ildr2	immunoglobulin-like domain containing receptor 2	-1.54	0.0279
Obox3-ps1	oocyte specific homeobox 3, pseudogene 1	1.72	0.0279
Ppp1r9b	protein phosphatase 1, regulatory subunit 9B	-1.66	0.0279
TC1200002202.mm.1		-1.87	0.028
TC0400001632.mm.1		1.74	0.028
TC1300001742.mm.1		1.53	0.028
Fam213b	family with sequence similarity 213, member B	-1.79	0.028
Sod1	superoxide dismutase 1, soluble	-3.78	0.028
Gm14946	predicted gene 14946	1.84	0.0281
Gm22202	predicted gene, 22202	1.54	0.0281
Tm4sf1	transmembrane 4 superfamily member 1	-1.72	0.0281
TC0400002422.mm.1		-6.67	0.0282
TC0600000497.mm.1		1.69	0.0282
Cltb	clathrin, light polypeptide (Lcb)	-1.5	0.0282
Gm10232	predicted pseudogene 10232	-1.65	0.0282
Gm3344	predicted gene 3344	1.53	0.0282
H2-Ke6	H2-K region expressed gene 6	-1.61	0.0282
S100b	S100 protein, beta polypeptide, neural	-1.7	0.0282
TC0300001460.mm.1		1.8	0.0283
TC1100004005.mm.1		1.95	0.0283
Gm10420	predicted gene 10420	-2.41	0.0283

Grina	glutamate receptor, ionotropic, N-methyl D-aspartate-associated protein 1 (glutamate binding)	-3.28	0.0283
Gm15801	predicted gene 15801	-6.35	0.0284
Gm8783	predicted pseudogene 8783	-6.35	0.0284
Mir7212	microRNA 7212	2.53	0.0284
Pfkm	phosphofructokinase, muscle	-1.6	0.0284
Slc35b1	solute carrier family 35, member B1	-1.6	0.0284
TC1200000542.mm.1		1.53	0.0285
Fxyd6	FXYD domain-containing ion transport regulator 6	-1.77	0.0285
Rprml	reprimo-like	-1.66	0.0285
Dynlrb1	dynein light chain roadblock-type 1	-3.55	0.0286
Pik3c2a	phosphatidylinositol 3-kinase, C2 domain containing, alpha polypeptide	-1.56	0.0286
Rpl17-ps4	ribosomal protein L17, pseudogene 4	-3.12	0.0286
Gm13973	predicted gene 13973	1.53	0.0287
Gm15418; AC115800.2	predicted gene 15418	1.51	0.0287
Gm20563	predicted gene, 20563	-4.28	0.0287
Mlc1	megalencephalic leukoencephalopathy with subcortical cysts 1 homolog (human)	-2.01	0.0287
Slc6a1	solute carrier family 6 (neurotransmitter transporter, GABA), member 1	-1.79	0.0287
TC0X00003293.mm.1		-1.53	0.0288
TC1200000920.mm.1		-1.6	0.0288
Gm26072	predicted gene, 26072	1.69	0.0288
Gnao1	guanine nucleotide binding protein, alpha O	-1.84	0.0288
H2afz	H2A histone family, member Z	-11.42	0.0288
TC0200003881.mm.1		2.91	0.0289
BC030336	cDNA sequence BC030336	-1.51	0.0289
Gm24857	predicted gene, 24857	1.52	0.0289
Rab7	RAB7, member RAS oncogene family	-5.62	0.0289
Rpl15-ps2	ribosomal protein L15, pseudogene 2	-2.72	0.0289
TC1600002040.mm.1		1.53	0.029
TC0800000833.mm.1		-1.67	0.029
Mir181d	microRNA 181d	1.56	0.029
Gm13226	predicted gene 13226	-3	0.0291
Sobp	sine oculis-binding protein homolog (Drosophila)	-1.75	0.0291
TC0400004038.mm.1		-1.64	0.0292
TC0200001490.mm.1		1.58	0.0292
TC0200002002.mm.1		1.55	0.0292

Cend1	cell cycle exit and neuronal differentiation 1	-1.75	0.0292
Gm26905	predicted gene, 26905	-4.85	0.0292
Hey1	hairy/enhancer-of-split related with YRPW motif 1	-4.86	0.0292
Gm2389	predicted pseudogene 2389	-4.56	0.0294
Gm3244	predicted pseudogene 3244	-2.06	0.0294
TC0100002730.mm.1		2.03	0.0295
Gm11263	predicted gene 11263	-4.1	0.0295
Gm3727	predicted gene 3727	1.73	0.0295
Mir28c	microRNA 28c (Mir28c), microRNA.	1.73	0.0295
Mtch1	mitochondrial carrier homolog 1 (C. elegans)	-2.01	0.0295
TC0X00002244.mm.1		1.79	0.0296
Gm14270	predicted gene 14270	-1.56	0.0296
Gm6341	predicted pseudogene 6341	-3.11	0.0296
LOC100862318	PREDICTED: uncharacterized LOC100862318, transcript variant 1 (LOC100862318), miscRNA.	-2.68	0.0296
TC0200003456.mm.1		1.56	0.0297
Gm24357	predicted gene, 24357	-7.08	0.0297
Oaz1-ps	ornithine decarboxylase antizyme 1, pseudogene	-5.42	0.0297
TC0500000530.mm.1		1.53	0.0298
Ogdh	oxoglutarate (alpha-ketoglutarate) dehydrogenase (lipoamide)	-1.73	0.0298
Rpl10-ps6	ribosomal protein L10, pseudogene 6	-5.28	0.0298
Vti1b	vesicle transport through interaction with t-SNAREs 1B	-1.61	0.0298
TC0400000970.mm.1		-1.59	0.0299
Gm24816	predicted gene, 24816	1.89	0.0299
Gm1840	predicted gene 1840	-4.46	0.03
4930414N06Rik	RIKEN cDNA 4930414N06 gene	1.57	0.0301
Gm14308	predicted gene 14308 (Gm14308), mRNA.	-6.25	0.0301
TC0800001971.mm.1		2.03	0.0302
Epas1	endothelial PAS domain protein 1	-4.83	0.0302
Gm22087	predicted gene, 22087	1.8	0.0302
Olfr348	olfactory receptor 348	1.51	0.0302
5830472F04Rik	RIKEN cDNA 5830472F04 gene	2.02	0.0303
TC0X00002499.mm.1		-2.94	0.0304
TC1300002516.mm.1		1.73	0.0304
Gm26701	predicted gene, 26701	-3.02	0.0304
Mrpl13	mitochondrial ribosomal protein L13	-1.54	0.0304

Polr2k-ps; LOC102238523	polymerase (RNA) II (DNA directed) polypeptide K, pseudogene	1.84	0.0304
Prps1	phosphoribosyl pyrophosphate synthetase 1	-1.85	0.0304
Ptp4a2	protein tyrosine phosphatase 4a2	-2.1	0.0304
TC1900000144.mm.1		-2.4	0.0305
Gm13413	predicted gene 13413	-2.91	0.0305
TC0600002151.mm.1		1.84	0.0306
TC1800000603.mm.1		1.88	0.0306
Tbc1d9	TBC1 domain family, member 9	-1.55	0.0306
Gm5805	predicted gene 5805	-2.22	0.0307
Rora	RAR-related orphan receptor alpha	-2.9	0.0307
TC1900001202.mm.1		1.56	0.0308
Gm11099	predicted gene 11099	1.83	0.0308
Gm22555	predicted gene, 22555	2.47	0.0308
Gm22990	predicted gene, 22990	-2.09	0.0308
Gm5908	predicted gene 5908	-3.13	0.0308
TC0200000614.mm.1		1.58	0.0309
Gm22530	predicted gene, 22530	1.6	0.0309
Gm5978	PREDICTED: predicted gene 5978 (Gm5978), mRNA.	-1.75	0.0309
Mtmr6	myotubularin related protein 6	-1.6	0.0309
Rpl28-ps3	ribosomal protein L28, pseudogene 3	-4.27	0.0309
Tspyl4	TSPY-like 4	-2.16	0.0309
Gm24730	predicted gene, 24730	1.56	0.031
Rps12-ps24	ribosomal protein S12, pseudogene 24	-3.35	0.031
TC0100002732.mm.1		-1.99	0.0311
TC1500000195.mm.1		1.8	0.0311
Dtymk	deoxythymidylate kinase	-1.58	0.0311
Gm22930	predicted gene, 22930	-2	0.0311
Gm5449	predicted pseudogene 5449	-2.23	0.0311
Olfr1055	olfactory receptor 1055	-2.03	0.0311
Snord82	small nucleolar RNA, C/D box 82	4.72	0.0311
Vkorc1	vitamin K epoxide reductase complex, subunit 1	-7.56	0.0311
Mir7054	microRNA 7054	1.72	0.0312
Ndufa4	NADH dehydrogenase (ubiquinone) 1 alpha subcomplex, 4	-5.06	0.0312
Unc50	unc-50 homolog (C. elegans)	-1.61	0.0312
Gm11249	predicted gene 11249	-4.34	0.0313
Gm11361	predicted pseudogene 11361	-3.37	0.0313
Tnpo3	transportin 3	-1.53	0.0313
TC0200001796.mm.1		-2.02	0.0314

Acsl5	acyl-CoA synthetase long-chain family member 5	-1.53	0.0314
Gm13433	predicted gene 13433	-8.82	0.0314
Papd4	PAP associated domain containing 4	-2.87	0.0314
Serpini1	serine (or cysteine) peptidase inhibitor, clade I, member 1	-1.52	0.0314
TC0800002366.mm.1		-2.76	0.0315
Coprs	coordinator of PRMT5, differentiation stimulator	-1.62	0.0315
Gm17069	predicted gene 17069	1.6	0.0315
Gm7003	Gm7003 predicted gene 7003	1.62	0.0315
TC1700002737.mm.1		-1.68	0.0316
TC1200001267.mm.1		1.83	0.0316
Gm17107	predicted gene 17107	1.51	0.0316
Zmat2	zinc finger, matrin type 2	-2.25	0.0316
TC1000001230.mm.1		1.87	0.0317
TC1700000094.mm.1		2.06	0.0317
TC0500001904.mm.1		1.59	0.0317
TC4_JH584293_random00000011.mm.1		-2.56	0.0317
TC1500001371.mm.1		-1.75	0.0317
Gm18354	predicted gene, 18354	1.64	0.0317
Gm25121	predicted gene, 25121	-1.55	0.0318
Gm5436	predicted pseudogene 5436	-2.72	0.0318
Gm14308	predicted gene 14308 (Gm14308), mRNA.	-6.94	0.0319
Gm20091	predicted gene, 20091	-5.27	0.0319
Tmod2	tropomodulin 2	-1.78	0.0319
TC1000002153.mm.1		1.62	0.032
Gm9013	predicted gene 9013	-2.31	0.032
Mir1958	microRNA 1958	-4	0.032
Rny1	RNA, Y1 small cytoplasmic, Ro-associated	-1.78	0.032
TC0600003386.mm.1		1.81	0.0321
Gm11334	predicted gene 11334	1.86	0.0321
Gm17221	predicted gene 17221	1.56	0.0321
TC1000001045.mm.1		-2.78	0.0322
Erh	enhancer of rudimentary homolog (Drosophila)	-1.97	0.0322
Gm11531	predicted gene 11531	-4.73	0.0322
Gpatch8	G patch domain containing 8	-2	0.0322
TC0100001808.mm.1		-5.67	0.0323
Mat2a	methionine adenosyltransferase II, alpha	-1.78	0.0323

TC0400003111.mm.1		1.67	0.0324
TC0800001375.mm.1		1.62	0.0324
Gm15724; RP23-246B24.3	predicted gene 15724	1.87	0.0324
Rpl9-ps6	ribosomal protein L9, pseudogene 6	-3.49	0.0324
TC1100002923.mm.1		2.2	0.0325
4930473O22Rik	RIKEN cDNA 4930473O22 gene	1.58	0.0325
Gm11808	predicted gene 11808	-3.15	0.0325
Gm24689	predicted gene, 24689	1.75	0.0325
Gm7536	predicted gene 7536	-2.4	0.0325
Gm6139	predicted gene 6139	-3.87	0.0326
Gm11675	predicted gene 11675	-2.3	0.0327
TC0X00002852.mm.1		1.74	0.0328
Gm23207	predicted gene, 23207	1.68	0.0328
TC0800002613.mm.1		1.74	0.0329
TC1300000485.mm.1		-3.62	0.0329
Eif4g2	eukaryotic translation initiation factor 4, gamma 2	-1.87	0.0329
Gm13335; RP23-204B24.1	predicted gene 13335	-1.97	0.0329
Gm17047	predicted gene 17047	2.07	0.0329
Gm3756	predicted gene 3756	-3.02	0.0329
Gm5762	predicted gene 5762	-1.61	0.0329
TC1600001116.mm.1		-3.96	0.033
Gm11560	predicted gene 11560	-1.91	0.033
Mir7081	microRNA 7081	1.62	0.033
Rpl29; Gm8210	ribosomal protein L29	-1.64	0.033
Pcbd2	pterin 4 alpha carbinolamine dehydratase/dimerization cofactor of hepatocyte nuclear factor 1 alpha (TCF1) 2	-1.69	0.0331
Ppp2r2a	protein phosphatase 2, regulatory subunit B, alpha	-1.7	0.0331
TC0200001155.mm.1		-1.51	0.0332
TC1300001308.mm.1		-2.23	0.0332
Eif5al3-ps	eukaryotic translation initiation factor 5A-like 3, pseudogene	-3.66	0.0332
Gm11425	predicted gene 11425	-2.37	0.0332
Gm16265	predicted gene 16265	-168.2	0.0332
Gm22663	predicted gene, 22663	2.01	0.0332
Glycam1	glycosylation dependent cell adhesion molecule 1	1.72	0.0333
Gm6304	predicted gene 6304	-6.9	0.0333

Lgi1	leucine-rich repeat LGI family, member 1	-2.15	0.0333
B930095G15Rik	RIKEN cDNA B930095G15 gene	-1.93	0.0334
Srp54a	signal recognition particle 54A	-1.83	0.0334
TC1100001364.mm.1		-2.01	0.0335
Cdkn2aipnl	CDKN2A interacting protein N-terminal like	-1.5	0.0335
Gm15846	predicted gene 15846	-1.64	0.0335
Gm3298	predicted gene 3298	2.04	0.0335
Gm25257	predicted gene, 25257	2.54	0.0336
LOC100861951	PREDICTED: uncharacterized LOC100861951 (LOC100861951), miscRNA.	2.49	0.0336
Scd2; Mir5114	stearoyl-Coenzyme A desaturase 2	-2.54	0.0336
TC0700000928.mm.1		1.91	0.0337
Gm10182	predicted pseudogene 10182	-6.64	0.0337
Gm5356	predicted pseudogene 5356	-1.76	0.0337
Mir378c	microRNA 378c	-16.41	0.0337
Arf3	ADP-ribosylation factor 3	-1.54	0.0338
Gm13777	predicted gene 13777	-1.8	0.0338
TC1200001368.mm.1		-3.53	0.0339
Ccdc50	coiled-coil domain containing 50	-2.29	0.0339
Grpel1	GrpE-like 1, mitochondrial	-1.69	0.0339
Rassf3	Ras association (RalGDS/AF-6) domain family member 3	-1.72	0.0339
Gm9719	predicted gene 9719	-1.65	0.034
Mir193b	microRNA 193b	1.75	0.034
Cryab	crystallin, alpha B	-1.7	0.0341
Tmem176b	transmembrane protein 176B	-1.59	0.0341
TC1700002601.mm.1		1.59	0.0342
TC0100002672.mm.1		-1.68	0.0342
TC1800001458.mm.1		1.83	0.0342
TC0500003042.mm.1		1.54	0.0342
Apod	apolipoprotein D	-4.97	0.0342
Gm4017; Gm10157	predicted gene 4017	-5.69	0.0342
Hsp90ab1	heat shock protein 90 alpha (cytosolic), class B member 1	-1.67	0.0342
LOC100862063	PREDICTED: uncharacterized LOC100862063 (LOC100862063), miscRNA.	-2.02	0.0342
Mprip	myosin phosphatase Rho interacting protein	-1.56	0.0342
Gm14308	predicted gene 14308 (Gm14308), mRNA.	-3.14	0.0343
Gm22712	predicted gene, 22712	-12.57	0.0343

Gm23308	predicted gene, 23308	1.52	0.0343
Gm26205	predicted gene, 26205	-2.58	0.0344
TC1100001715.mm.1		1.75	0.0346
Fam107a	family with sequence similarity 107, member A	-4.67	0.0346
Gm2026	predicted gene 2026	-4.52	0.0346
Map7d2	MAP7 domain containing 2	-1.64	0.0346
TC0500001246.mm.1		2.13	0.0347
Dusp19	dual specificity phosphatase 19	-1.53	0.0347
Gm10260	predicted gene 10260	-3.23	0.0347
Napb	N-ethylmaleimide sensitive fusion protein attachment protein beta	-2.15	0.0347
Atp6v0e2	ATPase, H+ transporting, lysosomal V0 subunit E2	-1.96	0.0348
Gm8399; Gm25241	predicted gene 8399	-2.43	0.0348
Rps6-ps1	ribosomal protein S6, pseudogene 1	-4.27	0.0348
TC0500003443.mm.1		-2.94	0.0349
TC1200001628.mm.1		1.55	0.0349
TC0600000239.mm.1		1.59	0.0349
Fam73a	family with sequence similarity 73, member A	-1.71	0.0349
TC1300001995.mm.1		-2.58	0.0351
TC0500000768.mm.1		1.56	0.0351
TC0500000535.mm.1		-1.53	0.0351
Agap2	ArfGAP with GTPase domain, ankyrin repeat and PH domain 2	-2.78	0.0351
Rpl3	ribosomal protein L3	-5.22	0.0351
Slc2a13	solute carrier family 2 (facilitated glucose transporter), member 13	-1.57	0.0351
TC0900000897.mm.1		-1.68	0.0352
TC0900003143.mm.1		-19.49	0.0352
Pfdn2	prefoldin 2	-1.56	0.0352
TC1000001386.mm.1		1.92	0.0353
TC1000002972.mm.1		1.92	0.0353
Gm20834	predicted gene, 20834	1.92	0.0353
Tmem38a	transmembrane protein 38A	-1.64	0.0353
TC1100003335.mm.1		2.57	0.0354
Gm10039	predicted pseudogene 10039	-5.25	0.0354
Gm20198	PREDICTED: predicted gene, 20198 (Gm20198), miscRNA.	-1.51	0.0354
Gm20210	PREDICTED: predicted gene, 20210 (Gm20210), miscRNA.	-1.51	0.0354
Gm22498	predicted gene, 22498	1.57	0.0354

TC1200000402.mm.1		1.66	0.0355
BC005537	cDNA sequence BC005537	-1.82	0.0356
Gm6682	predicted gene 6682 (Gm6682), non-coding RNA.	-4.28	0.0356
2610507B11Rik	RIKEN cDNA 2610507B11 gene	-1.85	0.0357
Mzt1	mitotic spindle organizing protein 1	-5.64	0.0357
Peak1	pseudopodium-enriched atypical kinase 1	-1.92	0.0357
Pten	phosphatase and tensin homolog	-3.89	0.0357
TC1700001629.mm.1		1.65	0.0358
Gba	glucosidase, beta, acid	-1.82	0.0358
Gm5265	predicted pseudogene 5265	-4.3	0.0358
Cfl1	cofilin 1, non-muscle	-1.72	0.0359
Gm13681	predicted gene 13681	1.57	0.0359
Gm14927	predicted gene 14927	-1.56	0.0359
TC1900001632.mm.1		1.68	0.036
TC1200001140.mm.1		-2.16	0.036
TC1000000190.mm.1		-13.22	0.0361
TC1500000117.mm.1		-13.22	0.0361
Mir3098	microRNA 3098	2.07	0.0361
Vkorc1l1	vitamin K epoxide reductase complex, subunit 1-like 1	-1.58	0.0361
Gm15481	predicted gene 15481	-2.23	0.0362
Gm8841	PREDICTED: predicted gene 8841 (Gm8841), mRNA.	-5.24	0.0362
TC0100002049.mm.1		1.58	0.0363
A430010J10Rik	RIKEN cDNA A430010J10 gene	1.62	0.0363
Rpl14-ps1	ribosomal protein L14, pseudogene 1	-2.06	0.0363
Calr	calreticulin	-1.57	0.0364
Igkv12-89	immunoglobulin kappa chain variable 12-89	1.6	0.0364
TC0200003105.mm.1		1.63	0.0365
TC1000002825.mm.1		-1.74	0.0365
TC1100003530.mm.1		-1.54	0.0365
TC1700002700.mm.1		-1.99	0.0365
TC0400001404.mm.1		1.68	0.0365
Gm14438	predicted gene 14438	-1.9	0.0365
TC0600002479.mm.1		-1.68	0.0366
TC1200000147.mm.1		1.56	0.0367
Gm26818	predicted gene, 26818	-2.44	0.0367
Rps8-ps4	ribosomal protein S8, pseudogene 4	-3.47	0.0368
TC1100002978.mm.1		2.1	0.0369
TC1000000094.mm.1		1.5	0.0369
Copz1	coatomer protein complex, subunit zeta 1	-1.72	0.0369

Rps2-ps10	ribosomal protein S2, pseudogene 10	-4.93	0.0369
TC0200004152.mm.1		1.55	0.037
TC0200001122.mm.1		3.93	0.037
Fabp9	fatty acid binding protein 9, testis	1.58	0.037
Gm11653	predicted gene 11653	-1.95	0.037
Gm10231	predicted pseudogene 10231	-1.82	0.0371
Gm17383	predicted gene, 17383	-6.03	0.0371
TC0X00002590.mm.1		1.61	0.0372
TC1700002607.mm.1		3.16	0.0372
TC1700001815.mm.1		1.51	0.0372
Mir6915	microRNA 6915	2.14	0.0372
TC0400003474.mm.1		1.56	0.0374
Gm12723	predicted gene 12723	-1.66	0.0374
Gm19491	PREDICTED: predicted gene, 19491 (Gm19491), miscRNA.	-2.23	0.0374
TC1900000825.mm.1		1.56	0.0375
Gm4968	predicted gene 4968	-2.33	0.0375
Rock1	Rho-associated coiled-coil containing protein kinase 1	-1.71	0.0375
TC1500001245.mm.1		1.68	0.0376
TC0500000146.mm.1		-2.25	0.0376
Gm7618	predicted pseudogene 7618	-1.93	0.0376
Gm8494	predicted gene 8494	1.65	0.0376
Mfsd5	major facilitator superfamily domain containing 5	-1.56	0.0376
Rpl27-ps1	ribosomal protein L27, pseudogene 1	-2.23	0.0376
Gsk3a	glycogen synthase kinase 3 alpha	-1.83	0.0377
TC1000002707.mm.1		1.88	0.0378
TC1200000772.mm.1		-4.15	0.0378
Rps19bp1	ribosomal protein S19 binding protein 1	-1.7	0.0378
Suclg1	succinate-CoA ligase, GDP-forming, alpha subunit	-1.53	0.0378
TC0300002587.mm.1		-1.52	0.0379
Gm12738	predicted gene 12738	1.65	0.0379
TC0400000693.mm.1		1.87	0.038
Mir7224	microRNA 7224	-5.97	0.038
TC1400001678.mm.1		-3.65	0.0381
TC0200004614.mm.1		1.51	0.0381
Rps8-ps2	ribosomal protein S8, pseudogene 2	-3.33	0.0381
St3gal5	ST3 beta-galactoside alpha-2,3-sialyltransferase 5	-2.18	0.0381
Tmem50a	transmembrane protein 50A	-7.57	0.0381
Gm10282	predicted pseudogene 10282	-6.28	0.0382

LOC100862227	PREDICTED: uncharacterized LOC100862227, transcript variant 1 (LOC100862227), miscRNA.	-2.08	0.0382
TC0200003514.mm.1		1.61	0.0383
Atp5f1	ATP synthase, H+ transporting, mitochondrial F0 complex, subunit B1	-1.88	0.0383
Fam168a	family with sequence similarity 168, member A	-1.66	0.0383
Gm13736; Srp9	predicted gene 13736	-1.94	0.0383
TC0300002769.mm.1		1.51	0.0385
Atp5g2	ATP synthase, H+ transporting, mitochondrial F0 complex, subunit C2 (subunit 9)	-1.67	0.0385
Cisd1	CDGSH iron sulfur domain 1	-1.76	0.0385
Eif4h	eukaryotic translation initiation factor 4H	-2.52	0.0385
Gm17428	predicted gene, 17428	3.19	0.0385
Tardbp	TAR DNA binding protein	-2.01	0.0385
TC1500000536.mm.1		1.74	0.0386
Gm12481	predicted gene 12481	-4.2	0.0386
Hprt	hypoxanthine guanine phosphoribosyl transferase	-2.74	0.0386
LOC100862216	PREDICTED: uncharacterized LOC100862216, transcript variant 1 (LOC100862216), miscRNA.	-3.34	0.0386
Rasgrp1	RAS guanyl releasing protein 1	-1.93	0.0386
Slc12a2	solute carrier family 12, member 2	-1.92	0.0386
Tagln3	transgelin 3	-1.82	0.0387
Cpt1a	carnitine palmitoyltransferase 1a, liver	-2.62	0.0389
Pcp4l1	Purkinje cell protein 4-like 1	-1.53	0.0389
Pten	phosphatase and tensin homolog	-1.99	0.0389
Rpl17-ps10; Gm10294	ribosomal protein L17, pseudogene 10	-1.93	0.0389
Trav12d-3	T cell receptor alpha variable 12D-3	1.61	0.0389
Gm15573	predicted gene 15573	-1.55	0.039
LOC100862378	PREDICTED: uncharacterized LOC100862378 (LOC100862378), miscRNA.	-2.18	0.039
Ncam1	neural cell adhesion molecule 1	-1.63	0.039
TC0X00001522.mm.1		1.52	0.0391
TC1300001184.mm.1		1.82	0.0391
2010107E04Rik	RIKEN cDNA 2010107E04 gene	-14.43	0.0391
Gm10737	predicted gene 10737	3.82	0.0391
Gm8927	predicted gene 8927	-1.55	0.0391
Gm9027	predicted gene 9027	-2.87	0.0392
TC0900003292.mm.1		1.83	0.0394

Gm11518	predicted gene 11518	-3.12	0.0394
Gm19645	PREDICTED: predicted gene, 19645 (Gm19645), miscRNA.	-1.68	0.0394
Nfkbia	nuclear factor of kappa light polypeptide gene enhancer in B cells inhibitor, alpha	-1.86	0.0394
TC1200002221.mm.1		1.54	0.0395
Gm10181	predicted gene 10181	9.68	0.0395
TC0700000948.mm.1		-1.95	0.0396
TC1600001583.mm.1		-1.9	0.0396
Dstn	destrin	-4.18	0.0396
Gm12038	predicted gene 12038	-2.06	0.0396
Gm28020	predicted gene, 28020	2.08	0.0396
TC1700000827.mm.1		2.43	0.0397
4930448D08Rik; AC108433.3	RIKEN cDNA 4930448D08 gene	1.99	0.0397
Atp1a3	ATPase, Na+/K+ transporting, alpha 3 polypeptide (Atp1a3), mRNA.	-2.36	0.0397
Gm24675	predicted gene, 24675	1.62	0.0397
Gm7664	predicted gene 7664	-1.72	0.0397
Rps12-ps1	ribosomal protein S12, pseudogene 1	-2.59	0.0398
TC0700004162.mm.1		1.67	0.0399
Arl6ip1	ADP-ribosylation factor-like 6 interacting protein 1	-4.49	0.0399
Gm12222	predicted gene 12222	-1.62	0.0399
Olfr849	olfactory receptor 849	1.54	0.0399
Gm8203	predicted pseudogene 8203	-19.05	0.04
Rhoa	ras homolog gene family, member A	-1.81	0.04
TC0700003344.mm.1		-3.99	0.0401
Adgrf5	adhesion G protein-coupled receptor F5	-2.32	0.0401
Gm14412	predicted gene 14412	-4.19	0.0401
Gm20430	predicted gene 20430	-5.93	0.0401
TC0500001738.mm.1		-1.5	0.0402
Ghitm	growth hormone inducible transmembrane protein	-5.63	0.0402
Gm23492	predicted gene, 23492	-1.61	0.0402
Ppm1a	protein phosphatase 1A, magnesium dependent, alpha isoform	-1.77	0.0402
Gm10778	predicted gene 10778 (Gm10778), mRNA.	1.54	0.0403
Gm14325	predicted gene 14325	-1.7	0.0403
Gm14326	predicted gene 14326	-3.83	0.0403
TC0100002467.mm.1		1.52	0.0404
TC0100001121.mm.1		-5.16	0.0404

Cdk10	cyclin-dependent kinase 10	-3.17	0.0404
Gabbr2	gamma-aminobutyric acid (GABA) B receptor, 2	-1.9	0.0404
Psd3	pleckstrin and Sec7 domain containing 3	-1.59	0.0404
TC1500000255.mm.1		1.56	0.0405
TC1_GL456210_random00000009.mm.1		-2.12	0.0405
TC1_GL456221_random00000005.mm.1		-2.12	0.0405
Gm11331	predicted gene 11331	-1.75	0.0405
Gm20917; Gm21469	predicted gene, 20917	1.59	0.0405
Gm3893; 4933409K07Rik	predicted gene 3893	-1.51	0.0405
TC1600000616.mm.1		3.29	0.0406
Gm13800; RP23-246E11.1	predicted gene 13800	2.23	0.0406
TC0500002372.mm.1		1.53	0.0407
TC0X00001364.mm.1		1.74	0.0407
Gm23662	predicted gene, 23662	1.67	0.0407
Tspan3	tetraspanin 3	-3.36	0.0407
TC0900000991.mm.1		1.75	0.0408
1110004F10Rik	RIKEN cDNA 1110004F10 gene	-1.87	0.0408
Gm12666	predicted gene 12666	-2.16	0.0408
Tmbim4	transmembrane BAX inhibitor motif containing 4	-1.64	0.0408
Uqcrc2	ubiquinol cytochrome c reductase core protein 2	-2.16	0.0408
Gm27025	predicted gene, 27025	1.57	0.0409
Gm5139	predicted gene 5139	-1.67	0.0409
TC1300002636.mm.1		-1.8	0.041
Itm2b	integral membrane protein 2B	-9.47	0.041
Calm3	calmodulin 3	-1.66	0.0411
Gm14287	predicted gene 14287	-5.47	0.0411
Gm25283	predicted gene, 25283	2.37	0.0411
Gm27910	predicted gene, 27910	-4.26	0.0411
Gm5621	predicted gene 5621	-2.25	0.0411
TC1400001159.mm.1		1.97	0.0412
TC0700003407.mm.1		7.54	0.0412
Omg	oligodendrocyte myelin glycoprotein	-1.52	0.0412
TC1000001034.mm.1		1.63	0.0413
TC0800000387.mm.1		-2.43	0.0413
Gm3239	predicted gene 3239	1.89	0.0413
Gm7117; Gm10275	predicted pseudogene 7117	-4.41	0.0413

Lyz2	lysozyme 2	-1.92	0.0413
Dync1h1	dynein cytoplasmic 1 heavy chain 1	-1.6	0.0414
Gm25986	predicted gene, 25986	-1.92	0.0414
Gm3317; Gm3488	predicted gene 3317 (Gm3317), mRNA.	1.78	0.0414
TC0300001272.mm.1		1.89	0.0416
Gm15013	predicted gene 15013	-2.08	0.0416
Gm29376	predicted gene 29376	-1.54	0.0416
Gm4945	predicted gene 4945	-1.55	0.0416
Mir667	microRNA 667	-1.97	0.0416
Mrps36-ps1	mitichondrial ribosomal protein S36, pseudogene 1	-5.65	0.0416
TC1400001034.mm.1		-8.93	0.0417
Gm8566	predicted pseudogene 8566	-26.2	0.0417
TC0900003111.mm.1		-1.83	0.0419
Gm14046	predicted gene 14046	-1.59	0.0419
Prkcdbp	protein kinase C, delta binding protein	-1.91	0.0419
Smek2	SMEK homolog 2, suppressor of mek1 (Dictyostelium)	-1.6	0.0419
Tmem59	transmembrane protein 59	-1.6	0.0419
TC0800002829.mm.1		1.92	0.042
TC0500002682.mm.1		1.51	0.0421
TC0200004246.mm.1		-1.53	0.0421
TC1700002479.mm.1		-2.57	0.0421
TC0300003048.mm.1		-1.58	0.0421
TC1400002410.mm.1		2.23	0.0421
TC0800002473.mm.1		2.37	0.0422
TC0300001931.mm.1		2.27	0.0423
TC0500000141.mm.1		-1.54	0.0424
Ndufb11	NADH dehydrogenase (ubiquinone) 1 beta subcomplex, 11	-4.09	0.0424
Samd8	sterile alpha motif domain containing 8	-1.78	0.0424
TC1000001080.mm.1		-6.12	0.0425
Ndufb6	NADH dehydrogenase (ubiquinone) 1 beta subcomplex, 6	-1.55	0.0425
TC1700001099.mm.1		1.6	0.0426
TC0X00000983.mm.1		-1.72	0.0426
TC1800001543.mm.1		1.74	0.0426
Epb41l1	erythrocyte membrane protein band 4.1 like 1	-1.63	0.0426
Gm24884	predicted gene, 24884	1.56	0.0426
Gm27704	predicted gene, 27704	-5.33	0.0426
Gm27839	predicted gene, 27839	-4	0.0426
Rheb	Ras homolog enriched in brain	-1.89	0.0426

TC0300001099.mm.1		1.61	0.0427
App	amyloid beta (A4) precursor protein	-1.68	0.0427
Gm25069	predicted gene, 25069	-1.55	0.0427
Gm25824	predicted gene, 25824	1.51	0.0427
Sdhb	succinate dehydrogenase complex, subunit B, iron sulfur (Ip)	-1.91	0.0427
TC1000001798.mm.1		-4	0.0428
TC0300002707.mm.1		1.54	0.0429
Gm13331	predicted gene 13331	-1.63	0.0429
TC1700000456.mm.1		2.92	0.043
TC1300001897.mm.1		1.5	0.043
Aplp2	amyloid beta (A4) precursor-like protein 2	-1.61	0.043
Gm13797	predicted gene 13797	-12.61	0.043
Gtf3c6	general transcription factor IIIC, polypeptide 6, alpha	-3.85	0.043
TC0200005216.mm.1		1.52	0.0431
TC1400000215.mm.1		1.51	0.0431
Rpl19-ps12	ribosomal protein L19, pseudogene 12	-2.05	0.0431
C030023E24Rik	RIKEN cDNA C030023E24 gene	-3.24	0.0432
Gm5558	predicted gene 5558	-3.8	0.0432
LOC100862127	PREDICTED: uncharacterized LOC100862127 (LOC100862127), miscRNA.	-1.56	0.0432
Slco1c1	solute carrier organic anion transporter family, member 1c1	-1.75	0.0432
LOC100862086	PREDICTED: uncharacterized LOC100862086 (LOC100862086), miscRNA.	-2.41	0.0433
Nr4a2	nuclear receptor subfamily 4, group A, member 2	-1.61	0.0433
Gm14284	predicted gene 14284	-24.09	0.0435
Gm6134	predicted pseudogene 6134	-2.01	0.0435
Gnb1	guanine nucleotide binding protein (G protein), beta 1	-1.58	0.0435
Hmgn2-ps1	high mobility group nucleosomal binding domain 2, pseudogene 1	1.68	0.0435
TC1800000957.mm.1		1.73	0.0436
TC0800001570.mm.1		1.83	0.0436
TC1800001470.mm.1		1.53	0.0436
TC0Y00000054.mm.1		2.1	0.0437
TC0X00001712.mm.1		1.66	0.0437
Anxa6	annexin A6	-1.51	0.0437
TC1600001095.mm.1		-1.59	0.0438

Gm26400	predicted gene, 26400	1.56	0.0439
Gm3275	predicted gene 3275	1.56	0.0439
Vmn1r-ps47	vomeronasal 1 receptor, pseudogene 47	1.83	0.0439
TC0200002880.mm.1		1.93	0.044
TC0800000272.mm.1		1.58	0.044
Amd-ps4	S-adenosylmethionine decarboxylase, pseudogene 4	-1.53	0.044
Gm15621	predicted gene 15621	-2.68	0.044
TC0700003121.mm.1		-2.22	0.0441
Gm16518	predicted gene, 16518	1.62	0.0441
Pdhb	pyruvate dehydrogenase (lipoamide) beta	-1.77	0.0441
Sumo3	small ubiquitin-like modifier 3	-1.88	0.0441
Gm12517	predicted gene 12517	-2	0.0442
Gm14877	predicted gene 14877	2.06	0.0442
TC1300001920.mm.1		1.59	0.0443
TC1200000907.mm.1		1.54	0.0443
Atp6v0a1	ATPase, H+ transporting, lysosomal V0 subunit A1	-1.57	0.0443
Gm25402	predicted gene, 25402	1.53	0.0443
Gm3873	PREDICTED: predicted gene 3873 (Gm3873), mRNA.	-3.59	0.0443
Dnttip2	deoxynucleotidyltransferase, terminal, interacting protein 2	-2.5	0.0444
TC1000000148.mm.1		-2.4	0.0445
TC0300001063.mm.1		1.51	0.0445
Rps12-ps19	ribosomal protein S12, pseudogene 19	-3.25	0.0445
Ube2nl	ubiquitin-conjugating enzyme E2N-like	-2.65	0.0445
Gm5582	predicted gene 5582	-4.53	0.0446
TC1200001672.mm.1		1.51	0.0447
Pdxk	pyridoxal (pyridoxine, vitamin B6) kinase	-1.8	0.0447
Pik3r3	phosphatidylinositol 3 kinase, regulatory subunit, polypeptide 3 (p55)	-1.83	0.0447
Bmi1	Bmi1 polycomb ring finger oncogene	-2.06	0.0448
TC0600000931.mm.1		-12.42	0.0449
Gm6822	predicted pseudogene 6822	-2.03	0.045
TC0300001954.mm.1		1.77	0.0455
Gja6	gap junction protein, alpha 6	1.71	0.0455
Ypel5	yippee-like 5 (Drosophila)	-1.72	0.0455
TC0X00002271.mm.1		1.52	0.0456
Eif1-ps3	eukaryotic translation initiation factor 1, pseudogene 3	-8.11	0.0456
TC0600003033.mm.1		2.27	0.0457
TC0600002621.mm.1		-1.84	0.0459

Fam171b	family with sequence similarity 171, member B	-2.25	0.046
Mir6976	microRNA 6976	1.75	0.046
Rpl28-ps1	ribosomal protein L28, pseudogene 1	-2.1	0.046
TC0900001929.mm.1		-2.87	0.0461
TC0300001341.mm.1		1.82	0.0461
TC0300000300.mm.1		1.5	0.0461
TC0Y00000214.mm.1		-1.6	0.0461
Lman2	lectin, mannose-binding 2	-1.5	0.0461
TC1000002663.mm.1		-1.99	0.0462
TC0400000120.mm.1		1.53	0.0462
TC1100000194.mm.1		2.05	0.0463
TC1200002255.mm.1		-5.02	0.0463
TC1400002541.mm.1		-2.57	0.0463
TC0900002801.mm.1		1.85	0.0463
Gm23858	predicted gene, 23858	2.22	0.0463
Pabpc1	poly(A) binding protein, cytoplasmic 1	-1.77	0.0463
TC0100003063.mm.1		1.56	0.0464
Rpl27-ps2	ribosomal protein L27, pseudogene 2	-2.68	0.0464
Rtcb	RNA 2,3-cyclic phosphate and 5-OH ligase	-4.35	0.0464
TC0200001465.mm.1		1.5	0.0465
TC1000001240.mm.1		1.65	0.0466
Gm24384	predicted gene, 24384	1.68	0.0466
Sacm1l	SAC1 (suppressor of actin mutations 1, homolog)-like (S. cerevisiae)	-1.54	0.0466
Gm25007	predicted gene, 25007	-1.66	0.0467
Hsp90b1	heat shock protein 90, beta (Grp94), member 1	-1.65	0.0467
Ptprm	protein tyrosine phosphatase, receptor type, M	-1.64	0.0467
TC0100000178.mm.1		-2.1	0.0468
TC1700002364.mm.1		-2.1	0.0468
TC1800000392.mm.1		1.54	0.0468
Gm27892	predicted gene, 27892	-2.49	0.0468
Pcdh7	protocadherin 7	-2.19	0.0468
Tmem14c	transmembrane protein 14C	-1.56	0.0468
TC0700001911.mm.1		-1.58	0.0469
Eif1	eukaryotic translation initiation factor 1	-1.64	0.0469
Mir6380	microRNA 6380	-1.6	0.0469
Rhox7b	reproductive homeobox 7B	1.55	0.047
TC1100004012.mm.1		-2.31	0.0471
TC0700001259.mm.1		1.84	0.0471

Gm12728	predicted gene 12728	-1.7	0.0471
TC0700004466.mm.1		-2.6	0.0472
Fxyd1	FXYD domain-containing ion transport regulator 1	-1.75	0.0472
Gm12096	predicted gene 12096	-2.01	0.0472
Pcsk2	proprotein convertase subtilisin/kexin type 2	-1.54	0.0472
Pomp	proteasome maturation protein	-3.23	0.0472
Trappc4	trafficking protein particle complex 4	-1.63	0.0472
Gm13249	predicted gene 13249	-2.03	0.0473
Gm22088	predicted gene, 22088	1.59	0.0473
Gm22610	predicted gene, 22610	-6.23	0.0473
AA386476	expressed sequence AA386476	1.5	0.0474
Gm11986; RP23-20C9.3	predicted gene 11986	1.61	0.0474
TC0400000607.mm.1		2.03	0.0475
TC1000000627.mm.1		1.56	0.0475
Gm25693	predicted gene, 25693	-2.35	0.0475
TC0500002611.mm.1		1.6	0.0476
Carm1	coactivator-associated arginine methyltransferase 1	-1.51	0.0476
Mir7668	microRNA 7668	1.53	0.0476
TC0500000891.mm.1		-4	0.0477
Wbp5	WW domain binding protein 5	-1.97	0.0477
TC0200003078.mm.1		1.6	0.0478
Gm14292	predicted gene 14292	-13.89	0.0478
Htra1	HtrA serine peptidase 1	-1.61	0.0478
Ephx1	epoxide hydrolase 1, microsomal	-2.13	0.0479
Gm12350	predicted gene 12350	-6.9	0.0479
Gm6768	predicted gene 6768	-5.54	0.0479
TC1300000968.mm.1		-1.5	0.048
TC0200002840.mm.1		3.31	0.048
TC0X00002574.mm.1		-2.27	0.048
Gm24624	predicted gene, 24624	1.53	0.0481
Gm6952	predicted gene 6952	1.64	0.0481
Hist2h2aa1; Hist2h2aa2	histone cluster 2, H2aa1	-1.69	0.0481
Itpr1	inositol 1,4,5-trisphosphate receptor 1	-1.68	0.0481
Prpf4b	PRP4 pre-mRNA processing factor 4 homolog B (yeast)	-1.6	0.0481
TC0700001992.mm.1		1.57	0.0482
TC0400000759.mm.1		1.55	0.0482
Gm10222	predicted gene 10222	6.06	0.0482
Gm14305	predicted gene 14305	-6.82	0.0482

Gm8724	predicted pseudogene 8724	-2.14	0.0482
Pdpk1	3-phosphoinositide dependent protein kinase 1	-1.6	0.0482
Gm14409	predicted gene 14409	-1.72	0.0483
TC0400003962.mm.1		1.53	0.0484
Gm15961; RP23-455J6.4	predicted gene 15961	-2.35	0.0484
TC1000001978.mm.1		1.67	0.0485
Gm27459	predicted gene, 27459	-3.14	0.0485
TC0900001961.mm.1		-1.7	0.0486
Aff4	AF4/FMR2 family, member 4	-2.01	0.0486
Gm12529	predicted gene 12529	-1.83	0.0486
Gm6433	predicted gene 6433	-1.67	0.0486
Gm12878	predicted gene 12878	1.8	0.0487
Gm19868	PREDICTED: predicted gene, 19868 (Gm19868), miscRNA.	-8.68	0.0487
Gm27152	predicted gene 27152	-1.79	0.0487
Rnaset2a; Rnaset2b	ribonuclease T2A	-4.14	0.0487
Gm14308; Gm14430; Gm14434; 0610010B08Rik; Gm4724; Gm11007	predicted gene 14308 (Gm14308), mRNA.	-3.23	0.0488
Gm24256	predicted gene, 24256	1.86	0.0488
Bloc1s1	biogenesis of lysosomal organelles complex-1, subunit 1	-2.23	0.0489
Gm27525	predicted gene, 27525	-3.09	0.0489
Resp18	regulated endocrine-specific protein 18	-5.92	0.0489
Cmtm5	CKLF-like MARVEL transmembrane domain containing 5	-2.81	0.049
TC0800001679.mm.1		-2.49	0.0491
TC0400001476.mm.1		-1.57	0.0491
Slc25a5	solute carrier family 25 (mitochondrial carrier, adenine nucleotide translocator), member 5	-3.28	0.0491
TC0900002006.mm.1		-7.17	0.0492
Gm14586	predicted gene 14586	-2.38	0.0492
Rplp1-ps1	ribosomal protein, large, P1, pseudogene 1	-1.71	0.0493
Gm24229	predicted gene, 24229	2.16	0.0494
Lum	lumican	-1.94	0.0494
TC0300000524.mm.1		1.67	0.0495
Gm2423	predicted gene 2423	-1.53	0.0495
Gm9294	predicted pseudogene 9294	-2.39	0.0495
Gm12469	predicted gene 12469	-2.78	0.0496

Ndufa2	NADH dehydrogenase (ubiquinone) 1 alpha subcomplex, 2	-11.54	0.0496
Nme1	NME/NM23 nucleoside diphosphate kinase 1	-2.18	0.0496
TC1300001092.mm.1		-4.06	0.0497
TC1700001804.mm.1		-3.91	0.0497
Gm8172	predicted pseudogene 8172	-4.39	0.0498
Gm8213	predicted pseudogene 8213	-1.59	0.0498
Rps2-ps6	ribosomal protein S2, pseudogene 6	-4.7	0.0498
Slc4a4	solute carrier family 4 (anion exchanger), member 4	-1.87	0.0498
Slc7a11	solute carrier family 7 (cationic amino acid transporter, y+ system), member 11	-3.94	0.0498
TC0600002180.mm.1		-1.52	0.0499
TC0100003631.mm.1		-1.56	0.0499
Gm14263	predicted gene 14263	-1.7	0.0499
Rpsa-ps4	ribosomal protein S4, pseudogene 4	-2.88	0.0499
Gm27437	predicted gene, 27437	-3.5	0.05

Supplementary Table 7: HGD+Curc/HGD Coding. Protein coding genes (mRNA) differentially expressed by HGD+Curc relative to HGD

Gene Symbol	Name	Fold Change	P-val
Dnajc4	DnaJ (Hsp40) homolog, subfamily C, member 4	-2.38	8.13E-06
Park7	Parkinson disease (autosomal recessive, early onset) 7	-1.95	5.46E-05
Lgals1	lectin, galactose binding, soluble 1	-2.21	9.21E-05
Arhgef7	Rho guanine nucleotide exchange factor (GEF7)	-1.68	0.0001
Camk2n1	calcium/calmodulin-dependent protein kinase II inhibitor 1	-2.19	0.0001
Cox7a2l	cytochrome c oxidase subunit VIIa polypeptide 2-like	-2.77	0.0001
Myl9	myosin, light polypeptide 9, regulatory	-3.22	0.0001
Aldoa	aldolase A, fructose-bisphosphate	-1.81	0.0002
Nbl1	neuroblastoma, suppression of tumorigenicity 1	-1.56	0.0002
Ppp1r14b	protein phosphatase 1, regulatory (inhibitor) subunit 14B	-2.39	0.0002
Tubg1	tubulin, gamma 1	-1.88	0.0002
Ost4	oligosaccharyltransferase 4 homolog (S. cerevisiae)	-2.7	0.0004
Ttc9b	tetratricopeptide repeat domain 9B	-2.24	0.0004
Cxcr6	chemokine (C-X-C motif) receptor 6	1.71	0.0005
Ik	IK cytokine	-3.38	0.0006

Jag1	jagged 1	-2.15	0.0006
Myh11	myosin, heavy polypeptide 11, smooth muscle	-2.14	0.0007
Pcyox1	prenylcysteine oxidase 1	-1.8	0.0007
Abhd3	abhydrolase domain containing 3	-2.27	0.0009
Ndst1	N-deacetylase/N-sulfotransferase (heparan glucosaminyl) 1	-1.55	0.0009
Triqk	triple QxxK/R motif containing	-2.01	0.0009
Copb2	coatomer protein complex, subunit beta 2 (beta prime)	-2.37	0.0011
Cox6a1	cytochrome c oxidase subunit VIa polypeptide 1	-3.48	0.0011
Olfr113	olfactory receptor 113	2.08	0.0012
Tgfb3	transforming growth factor, beta 3	-2.28	0.0015
Baalc	brain and acute leukemia, cytoplasmic	-1.77	0.0017
Olfr1282	olfactory receptor 1282	1.66	0.0018
Rplp0	ribosomal protein, large, P0	-2.33	0.0018
Tas2r130	taste receptor, type 2, member 130	1.55	0.0018
Mrps16	mitochondrial ribosomal protein S16	-1.57	0.0019
Tm7sf3	transmembrane 7 superfamily member 3	-1.61	0.002
Rarres2	retinoic acid receptor responder (tazarotene induced) 2	-1.79	0.0023
Cdh5	cadherin 5	-1.65	0.0024
Ppp1r15b	protein phosphatase 1, regulatory (inhibitor) subunit 15b	-1.59	0.0024
Arhgdia	Rho GDP dissociation inhibitor (GDI) alpha	-1.67	0.0025
Atp6v1c1	ATPase, H+ transporting, lysosomal V1 subunit C1	-1.8	0.0026
Pkm	pyruvate kinase, muscle	-1.54	0.0026
Atp2b4	ATPase, Ca++ transporting, plasma membrane 4	-2.85	0.0027
Olfr855	olfactory receptor 855	1.51	0.0028
Slc25a18	solute carrier family 25 (mitochondrial carrier), member 18	-1.52	0.0028
Atp6v0b	ATPase, H+ transporting, lysosomal V0 subunit B	-3.31	0.0029
Rab24	RAB24, member RAS oncogene family	-3.34	0.0029
Ubc	ubiquitin C	-4.86	0.0032
Pcna	proliferating cell nuclear antigen	-2.22	0.0033
Pphln1	periphilin 1	-1.69	0.0033
Uqcrc1	ubiquinol-cytochrome c reductase core protein 1	-2.15	0.0036
Amd2	S-adenosylmethionine decarboxylase 2	-3.8	0.0037
Camk4	calcium/calmodulin-dependent protein kinase IV	-2	0.0039
Pip5k1c	phosphatidylinositol-4-phosphate 5-kinase, type 1 gamma	-1.62	0.0039
Clstn1	calsyntenin 1	-2.02	0.004
Slc30a10	solute carrier family 30, member 10	-1.69	0.004
Arf1	ADP-ribosylation factor 1	-1.72	0.0041
Cdk19	cyclin-dependent kinase 19	-5.91	0.0042
Ndufb7	NADH dehydrogenase (ubiquinone) 1 beta subcomplex, 7	-2.76	0.0042
Agrn	agrin	-1.78	0.0043
Flna	filamin, alpha	-2.48	0.0044
Gm20721	predicted gene, 20721 [Source:MGI Symbol	-1.74	0.0044

Ncl	nucleolin	-2.03	0.0044
Vmn1r27	vomeronasal 1 receptor 27	1.53	0.0045
Cd248	CD248 antigen, endosialin	-2.55	0.0046
Dctn2	dynactin 2	-1.95	0.0047
Eef2	eukaryotic translation elongation factor 2	-2.42	0.0049
Wdr74	WD repeat domain 74	-2.17	0.0049
Pdxp	pyridoxal (pyridoxine, vitamin B6) phosphatase	-1.65	0.005
Vmn1r42	vomeronasal 1 receptor 42	1.64	0.005
Fam205a1	family with sequence similarity 205, member A1	-1.57	0.0051
Krtap27-1	keratin associated protein 27-1	1.53	0.0051
Rnf187	ring finger protein 187	-2.06	0.0051
Commd3	COMM domain containing 3	-2.01	0.0053
Ndufs3	NADH dehydrogenase (ubiquinone) Fe-S protein 3	-2.77	0.0054
Plpp3	phospholipid phosphatase 3	-2.45	0.0054
Psmb5	proteasome (prosome, macropain) subunit, beta type 5	-4.28	0.0055
Cox5b	cytochrome c oxidase subunit Vb	-3.69	0.0057
Ptprb	protein tyrosine phosphatase, receptor type, B	-3.12	0.0057
Atp1a1	ATPase, Na+/K+ transporting, alpha 1 polypeptide	-3.11	0.0058
Dnajc19	DnaJ (Hsp40) homolog, subfamily C, member 19	-2.4	0.0059
4921524J17Rik	RIKEN cDNA 4921524J17 gene	-1.87	0.006
Ndufv3	NADH dehydrogenase (ubiquinone) flavoprotein 3	-1.82	0.006
Psma6	proteasome (prosome, macropain) subunit, alpha type 6	-2.2	0.0061
Ppa1	pyrophosphatase (inorganic) 1	-2.61	0.0063
Krtap28-13	PREDICTED: keratin associated protein 28-13 (Krtap28-13), mRNA.	1.51	0.0065
Mertk	c-mer proto-oncogene tyrosine kinase	-1.53	0.0065
Gm10406	predicted gene 10406	1.67	0.0066
Atp6ap2	ATPase, H+ transporting, lysosomal accessory protein 2	-2.66	0.0067
Vamp2	vesicle-associated membrane protein 2	-1.83	0.0067
Polr2f	polymerase (RNA) II (DNA directed) polypeptide F	-3.25	0.0068
Gstp1	glutathione S-transferase, pi 1	-2.14	0.0069
Gm19935	PREDICTED: predicted gene, 19935 (Gm19935), miscRNA.	2.28	0.007
Ccdc169	coiled-coil domain containing 169	1.58	0.0071
Cdc42ep1	CDC42 effector protein (Rho GTPase binding) 1	-1.83	0.0071
Ctsb	cathepsin B	-2.91	0.0073
Kif5a	kinesin family member 5A	-5.41	0.0074
Uck2	uridine-cytidine kinase 2	-2.81	0.0074
Cnot6	CCR4-NOT transcription complex, subunit 6	-1.65	0.0075
Eogt	EGF domain-specific O-linked N-acetylglucosamine (GlcNAc) transferase	-1.65	0.0077
Gm2974	predicted gene 2974 [Source:MGI Symbol	1.63	0.0077
Olfr159	olfactory receptor 159	1.54	0.0077

Stk39	serine/threonine kinase 39	-2.72	0.0077
Sh3bgrl3	SH3 domain binding glutamic acid-rich protein-like 3	-1.58	0.0079
Sipa1l1	signal-induced proliferation-associated 1 like 1	-2.49	0.0079
Slc38a3	solute carrier family 38, member 3	-1.85	0.0079
Sdha	succinate dehydrogenase complex, subunit A, flavoprotein (Fp)	-2.45	0.0083
Wwp1	WW domain containing E3 ubiquitin protein ligase 1	-1.85	0.0083
Nfe2l1	nuclear factor, erythroid derived 2,-like 1	-1.64	0.0084
Polr2j	polymerase (RNA) II (DNA directed) polypeptide J	-1.83	0.0084
Brms1l	breast cancer metastasis-suppressor 1-like	-2.03	0.0086
Rab21	RAB21, member RAS oncogene family	-1.93	0.0086
Vmn1r209	vomeronasal 1 receptor 209	1.8	0.0086
Ndufs6	NADH dehydrogenase (ubiquinone) Fe-S protein 6	-3	0.0089
Mcam	melanoma cell adhesion molecule	-1.92	0.009
Sv2b	synaptic vesicle glycoprotein 2 b	-3.01	0.009
Atl1	activating transcription factor 2	-1.61	0.0091
Ndufa1	NADH dehydrogenase (ubiquinone) 1 alpha subcomplex, 1	-13.19	0.0092
Krtap19-3	keratin associated protein 19-3	1.63	0.0093
Clybl	citrate lyase beta like	-1.52	0.0094
Ndufa9	NADH dehydrogenase (ubiquinone) 1 alpha subcomplex, 9	-3.13	0.0094
Bcan	brevican	-1.63	0.0097
Gm5941	predicted gene 5941	1.63	0.0098
Pitpnb	phosphatidylinositol transfer protein, beta	-2.11	0.0098
Dnttip1	deoxynucleotidyltransferase, terminal, interacting protein 1	-1.84	0.0099
Lonp2	lon peptidase 2, peroxisomal	-2.26	0.0099
Tecr	trans-2,3-enoyl-CoA reductase	-2.93	0.0099
Ildr2	immunoglobulin-like domain containing receptor 2	-1.61	0.01
Ilf2	interleukin enhancer binding factor 2	-1.61	0.01
Rnf4	ring finger protein 4	-1.87	0.01
Rps15	ribosomal protein S15	-1.71	0.0103
Ypel3	yippee-like 3 (Drosophila)	-1.51	0.0104
Kcna1	potassium voltage-gated channel, shaker-related subfamily, member 1	-3.66	0.0105
Lrp1	low density lipoprotein receptor-related protein 1	-1.61	0.0105
Arpp19	cAMP-regulated phosphoprotein 19	-1.82	0.0107
Prpf18	PRP18 pre-mRNA processing factor 18 homolog (yeast)	-2.01	0.0108
Orc4	origin recognition complex, subunit 4	-1.5	0.0109
Gm2897	predicted gene 2897 (Gm2897), transcript variant 1, mRNA.	1.71	0.011
Smim13	small integral membrane protein 13	-1.64	0.011
Atp5b	ATP synthase, H+ transporting mitochondrial F1 complex, beta subunit	-2.94	0.0111
Smarcd1	SWI/SNF related, matrix associated, actin dependent regulator of chromatin, subfamily d, member 1	-1.5	0.0111

Higd2a	HIG1 domain family, member 2A	-8.94	0.0112
Ndufs4	NADH dehydrogenase (ubiquinone) Fe-S protein 4	-4.49	0.0113
Rps4x	ribosomal protein S4, X-linked	-4.37	0.0115
Smdt1	single-pass membrane protein with aspartate rich tail 1	-8.99	0.0116
Hba-a2	hemoglobin alpha, adult chain 2	-4.08	0.0119
Crbn	Cereblon	-2.09	0.012
Akr1a1	aldo-keto reductase family 1, member A1 (aldehyde reductase)	-2.92	0.0121
Actb	actin, beta	-3.52	0.0122
Kctd13	potassium channel tetramerisation domain containing 13	-1.74	0.0122
Dap3	death associated protein 3	-1.77	0.0123
Tgoln1	trans-golgi network protein	-2.98	0.0124
Vamp5	vesicle-associated membrane protein 5	-1.65	0.0124
Rbx1	ring-box 1	-4.2	0.0125
Slc38a11	solute carrier family 38, member 11	-2.11	0.0125
Cask	calcium/calmodulin-dependent serine protein kinase (MAGUK family)	-1.59	0.0127
Olfr509	olfactory receptor 509	1.94	0.0127
Olfr948	olfactory receptor 948	1.74	0.0127
Chmp1b	charged multivesicular body protein 1B	-1.54	0.0128
Maf	avian musculoaponeurotic fibrosarcoma (v-maf) AS42 oncogene homolog	-2.11	0.0128
Rpl18	ribosomal protein L18	-1.62	0.0128
Eif5a	eukaryotic translation initiation factor 5A	-1.57	0.0129
Cct2	chaperonin containing Tcp1, subunit 2 (beta)	-1.7	0.013
D8Ertd738e	DNA segment, Chr 8, ERATO Doi 738, expressed	-5.55	0.013
Necap1	NECAP endocytosis associated 1	-1.55	0.013
Clta	clathrin, light polypeptide (Lca)	-1.53	0.0131
Igfbp7	insulin-like growth factor binding protein 7	-2.37	0.0132
Psmc1	protease (prosome, macropain) 26S subunit, ATPase 1	-2.51	0.0132
Scn1b	sodium channel, voltage-gated, type I, beta	-1.65	0.0134
Cnn3	calponin 3, acidic	-2.01	0.0135
Rpl36	ribosomal protein L36	-2.09	0.0136
Epm2aip1	EPM2A (laforin) interacting protein 1	-1.69	0.0137
Manbal	mannosidase, beta A, lysosomal-like	-2.19	0.0137
Nell2	NEL-like 2	-2.84	0.0137
Grm3	glutamate receptor, metabotropic 3	-2.41	0.0138
Msl2	male-specific lethal 2 homolog (Drosophila)	-2.8	0.0138
Psmc5	protease (prosome, macropain) 26S subunit, ATPase 5	-3.37	0.0138
Olfr692	olfactory receptor 692	1.7	0.014
Ddn	dendrin	-2.22	0.0141
Gkn3	gastrokine 3	-2.44	0.0141
Drg1	developmentally regulated GTP binding protein 1	-6.08	0.0142

Rtf1	Rtf1, Paf1/RNA polymerase II complex component, homolog (S. cerevisiae)	-1.59	0.0144
Aplp1	amyloid beta (A4) precursor-like protein 1	-1.77	0.0145
Cox5a	cytochrome c oxidase subunit Va	-6.6	0.0145
Fkbp2	FK506 binding protein 2	-1.95	0.0149
Mrpl27	mitochondrial ribosomal protein L27	-1.64	0.015
Nlk	nemo like kinase	-1.62	0.0151
Olfr460	olfactory receptor 460	1.53	0.0151
Lin7a	lin-7 homolog A (C. elegans)	-2.77	0.0152
Bap1	Brca1 associated protein 1	-1.61	0.0153
Atp1b1	ATPase, Na+/K+ transporting, beta 1 polypeptide	-5.3	0.0154
Clk3	CDC-like kinase 3	-2.53	0.0154
Olfr813	olfactory receptor 813	1.74	0.0155
Gm7324	predicted gene 7324 [Source:MGI Symbol	-2.1	0.0156
Prpf8	pre-mRNA processing factor 8	-3.01	0.0156
Gpr37l1	G protein-coupled receptor 37-like 1	-2.03	0.0157
Oxct1	3-oxoacid CoA transferase 1	-1.81	0.0157
Atp5g3	ATP synthase, H+ transporting, mitochondrial F0 complex, subunit C3 (subunit 9)	-1.9	0.0159
Cox7a2	cytochrome c oxidase subunit VIIa 2	-32.64	0.0159
Olfr676	olfactory receptor 676	1.58	0.0159
Vamp1	vesicle-associated membrane protein 1	-2.73	0.0159
Id1	inhibitor of DNA binding 1	-1.88	0.016
Rplp1	ribosomal protein, large, P1	-12.14	0.0161
Scap	SREBF chaperone	-1.6	0.0161
Lamb2	laminin, beta 2	-1.51	0.0162
Tsn	translin	-2	0.0162
Uqcr11	ubiquinol-cytochrome c reductase, complex III subunit XI	-2.64	0.0162
Olfr854	olfactory receptor 854	1.62	0.0163
Ppp1r14a	protein phosphatase 1, regulatory (inhibitor) subunit 14A	-2.63	0.0163
Vmn1r3	vomeronasal 1 receptor 3	1.78	0.0165
Atp6v0c	ATPase, H+ transporting, lysosomal V0 subunit C	-1.79	0.0166
Ddrgk1	DDRGK domain containing 1	-1.66	0.0166
Mrpl42	mitochondrial ribosomal protein L42	-2.23	0.0166
Cct7	chaperonin containing Tcp1, subunit 7 (eta)	-1.63	0.0167
Snapc1	small nuclear RNA activating complex, polypeptide 1	-1.5	0.0167
Atp1b2	ATPase, Na+/K+ transporting, beta 2 polypeptide	-1.59	0.0168
Arl6ip5	ADP-ribosylation factor-like 6 interacting protein 5	-1.91	0.0169
Lancl1	LanC (bacterial lantibiotic synthetase component C)-like 1	-2.18	0.0169
Ppib	peptidylprolyl isomerase B	-1.52	0.0169
Etnk1	ethanolamine kinase 1	-6.39	0.017
Pdcd4	programmed cell death 4	-2.81	0.017

Ttc9	tetratricopeptide repeat domain 9	-1.79	0.0171
Cyfip2	cytoplasmic FMR1 interacting protein 2	-2.11	0.0172
Dennd6a	DENN/MADD domain containing 6A	-1.69	0.0172
Hnrnpab	heterogeneous nuclear ribonucleoprotein A/B	-1.85	0.0173
Idh3g	isocitrate dehydrogenase 3 (NAD+), gamma	-1.58	0.0173
Pja2	praja 2, RING-H2 motif containing	-3.29	0.0173
Eef1b2	eukaryotic translation elongation factor 1 beta 2	-2.04	0.0175
Hspa4	heat shock protein 4	-2.05	0.0175
Srsf7	serine/arginine-rich splicing factor 7	-1.93	0.0175
Trappc11	trafficking protein particle complex 11	-1.76	0.0175
Kif1b	kinesin family member 1B	-1.8	0.0176
Gatc	glutamyl-tRNA(Gln) amidotransferase, subunit C	-3.53	0.0177
Ndufa13	NADH dehydrogenase (ubiquinone) 1 alpha subcomplex, 13	-5.91	0.0177
Wdr47	WD repeat domain 47	-1.66	0.0177
Sdf2	stromal cell derived factor 2	-1.86	0.0178
Lpgat1	lysophosphatidylglycerol acyltransferase 1	-1.5	0.018
Oaz1	ornithine decarboxylase antizyme 1	-1.7	0.0181
Sf3a3	splicing factor 3a, subunit 3	-1.86	0.0181
Inmt	indolethylamine N-methyltransferase	-1.66	0.0182
Zfp11	zinc finger protein 11	1.61	0.0182
Atl2	atlastin GTPase 2	-2.63	0.0184
Cd81	CD81 antigen	-4.65	0.0184
Lynx1	Ly6/neurotoxin 1	-1.61	0.0185
Vps28	vacuolar protein sorting 28 (yeast)	-3.19	0.0186
Id2	inhibitor of DNA binding 2	-3.15	0.0187
Rabac1	Rab acceptor 1 (prenylated)	-4.46	0.0187
Actg1	actin, gamma, cytoplasmic 1	-2.51	0.0188
Polb	polymerase (DNA directed), beta	-2.2	0.0188
Rpl14	ribosomal protein L14	-3.06	0.0188
Scarb2	scavenger receptor class B, member 2	-1.87	0.0188
Olfr663	olfactory receptor 663	1.52	0.0189
Tmem30a	transmembrane protein 30A	-3.11	0.0189
Vps4a	vacuolar protein sorting 4a (yeast)	-1.57	0.0189
Asah1	N-acylsphingosine amidohydrolase 1	-1.53	0.019
Cdc37l1	cell division cycle 37-like 1	-1.56	0.019
Puf60	poly-U binding splicing factor 60	-1.51	0.019
Kpna3	karyopherin (importin) alpha 3	-1.71	0.0191
Rpl4	ribosomal protein L4	-13.51	0.0191
Ap3d1	adaptor-related protein complex 3, delta 1 subunit	-1.63	0.0192
Vmn1r205	vomeronasal 1 receptor 205	1.56	0.0193
Mgrn1	mahogunin, ring finger 1	-1.84	0.0194
Vps29	vacuolar protein sorting 29 (S. pombe)	-1.78	0.0195

Txn1	thioredoxin 1	-2.67	0.0196
Apbb1	amyloid beta (A4) precursor protein-binding, family B, member 1	-1.59	0.0198
Ncdn	Neurochondrin	-1.57	0.0199
Gm10131	predicted pseudogene 10131 [Source:MGI Symbol	-9.7	0.02
Olfr700	olfactory receptor 700	1.77	0.02
Gcnt2	glucosaminyl (N-acetyl) transferase 2, I-branching enzyme	-1.82	0.0201
Ssr2	signal sequence receptor, beta	-3.45	0.0201
Taldo1	transaldolase 1	-1.8	0.0201
Tuba1b	tubulin, alpha 1B	-2.37	0.0201
Krtap19-9b	keratin associated protein 19-9B	1.56	0.0203
Mapre1	microtubule-associated protein, RP/EB family, member 1	-1.63	0.0203
Uchl1	ubiquitin carboxy-terminal hydrolase L1	-4.06	0.0203
Nr1d1	nuclear receptor subfamily 1, group D, member 1	-1.61	0.0205
Isca1	iron-sulfur cluster assembly 1 homolog (S. cerevisiae)	-1.51	0.0206
Ybx1	Y box protein 1	-1.85	0.0206
Gm10053	predicted gene 10053 [Source:MGI Symbol	-12.56	0.0207
Psmd11	proteasome (prosome, macropain) 26S subunit, non-ATPase, 11	-1.73	0.0209
Vmn1r79	vomeronasal 1 receptor 79	1.56	0.021
Dram2	DNA-damage regulated autophagy modulator 2	-2.07	0.0211
Dad1	defender against cell death 1	-1.77	0.0213
Rfxap	regulatory factor X-associated protein	-1.53	0.0213
Eif2b5	eukaryotic translation initiation factor 2B, subunit 5 epsilon	-1.51	0.0214
Raph1	Ras association (RalGDS/AF-6) and pleckstrin homology domains 1	-1.96	0.0214
Scyl1	SCY1-like 1 (S. cerevisiae)	-1.56	0.0214
0610009B22Rik	RIKEN cDNA 0610009B22 gene	-1.62	0.0215
Cops3	COP9 (constitutive photomorphogenic) homolog, subunit 3 (Arabidopsis thaliana)	-1.84	0.0216
Ap1g1	adaptor protein complex AP-1, gamma 1 subunit	-1.51	0.0217
Cap2	CAP, adenylate cyclase-associated protein, 2 (yeast)	-2.21	0.0218
Rnf11	ring finger protein 11	-1.69	0.0218
Tspan5	tetraspanin 5	-1.52	0.0218
Zxdb	zinc finger, X-linked, duplicated B	-1.56	0.0218
Nt5c	5,3-nucleotidase, cytosolic	-1.51	0.022
Olfr1382	olfactory receptor 1382	1.71	0.022
Rab6b	RAB6B, member RAS oncogene family	-1.64	0.0221
Ano1	anoctamin 1, calcium activated chloride channel	-1.76	0.0223
Romo1	reactive oxygen species modulator 1	-1.6	0.0224
Ifi27	interferon, alpha-inducible protein 27	-2.14	0.0226
Lrrc58	leucine rich repeat containing 58	-2.24	0.0226
Cd63	CD63 antigen	-2.59	0.0228

Nedd4	neural precursor cell expressed, developmentally down-regulated 4	-2.44	0.0228
Pik3ca	phosphatidylinositol 3-kinase, catalytic, alpha polypeptide	-1.65	0.0229
Acta2	actin, alpha 2, smooth muscle, aorta	-3.15	0.023
Gnai2	guanine nucleotide binding protein (G protein), alpha inhibiting 2	-2.01	0.023
Ptgds	prostaglandin D2 synthase (brain)	-3.21	0.0232
Cox8a	cytochrome c oxidase subunit VIIIa	-3.57	0.0233
Eif3d	eukaryotic translation initiation factor 3, subunit D	-3.18	0.0234
Oat	ornithine aminotransferase	-1.6	0.0234
Sgpp2	sphingosine-1-phosphate phosphotase 2	-2.75	0.0235
Slc6a6	solute carrier family 6 (neurotransmitter transporter, taurine), member 6	-2.28	0.0236
Fam174a	family with sequence similarity 174, member A	-2.02	0.0237
Ssr1	signal sequence receptor, alpha	-1.61	0.0238
Aldoc	aldolase C, fructose-bisphosphate	-1.93	0.0243
Cox4i1	cytochrome c oxidase subunit IV isoform 1	-1.56	0.0243
Fads3	fatty acid desaturase 3	-1.76	0.0243
Klf6	Kruppel-like factor 6	-1.51	0.0243
Cript	cysteine-rich PDZ-binding protein	-1.93	0.0244
Rbm4	RNA binding motif protein 4	-1.72	0.0244
Cuta	cutA divalent cation tolerance homolog (E. coli)	-1.53	0.0245
Gpm6a	glycoprotein m6a	-3.65	0.0245
Hnrnpa2b1	heterogeneous nuclear ribonucleoprotein A2/B1	-1.6	0.0245
Slc25a11	solute carrier family 25 (mitochondrial carrier oxoglutarate carrier), member 11	-1.88	0.0245
Atf2	amplified spermatogenic transcripts X encoded 3	-2.1	0.0246
Ano6	anoctamin 6	-1.61	0.0249
Ccnd2	cyclin D2	-2.12	0.025
Crip1	cysteine-rich protein 1 (intestinal)	-2.71	0.0251
Ndufv2	NADH dehydrogenase (ubiquinone) flavoprotein 2	-3.36	0.0252
Fis1	fission 1 (mitochondrial outer membrane) homolog (yeast)	-2.35	0.0253
Ap4s1	adaptor-related protein complex AP-4, sigma 1	-1.92	0.0254
Kpnb1	karyopherin (importin) beta 1	-1.6	0.0254
Gstp2	glutathione S-transferase, pi 2	-1.84	0.0256
Laptm4a	lysosomal-associated protein transmembrane 4A	-3.44	0.0256
Slc25a3	solute carrier family 25 (mitochondrial carrier, phosphate carrier), member 3	-1.89	0.0256
Atp2b1	ATPase, Ca++ transporting, plasma membrane 1	-1.74	0.0258
Gpi1	glucose phosphate isomerase 1	-1.74	0.026
Atp1a2	ATPase, Na+/K+ transporting, alpha 2 polypeptide	-2.45	0.0262
Pcdh9	protocadherin 9	-2.55	0.0262
Sf3b1	splicing factor 3b, subunit 1	-1.91	0.0262

Bmpr2	bone morphogenetic protein receptor, type II (serine/threonine kinase)	-6.25	0.0263
Cul3	cullin 3	-1.58	0.0263
Rpl18a	ribosomal protein L18A	-3.75	0.0263
Trf	transferrin	-2.16	0.0263
Pfdn5	prefoldin 5	-6.99	0.0264
Atp5a1	ATP synthase, H+ transporting, mitochondrial F1 complex, alpha subunit 1	-1.9	0.0266
Spcs1	signal peptidase complex subunit 1 homolog (S. cerevisiae)	-1.59	0.0266
Ostc	oligosaccharyltransferase complex subunit	-7.03	0.0268
Rtn4	reticulon 4	-1.58	0.0268
Sort1	sortilin 1	-1.93	0.0268
Atp6v1d	ATPase, H+ transporting, lysosomal V1 subunit D	-4.21	0.027
Myl6	myosin, light polypeptide 6, alkali, smooth muscle and non-muscle	-2.57	0.027
Slc2a1	solute carrier family 2 (facilitated glucose transporter), member 1	-2.41	0.027
Gm14391	predicted gene 14391 (Gm14391), transcript variant 1, mRNA.	-1.76	0.0271
Gm15557	predicted gene 15557 [Source:MGI Symbol	1.55	0.0271
Timm13	translocase of inner mitochondrial membrane 13	-1.78	0.0271
Cdc42	cell division cycle 42	-2.34	0.0272
Rap2a	RAS related protein 2a	-1.77	0.0272
Rpl8	ribosomal protein L8	-4.84	0.0272
Serf2	small EDRK-rich factor 2	-1.61	0.0272
4833439L19Rik	RIKEN cDNA 4833439L19 gene	-2.61	0.0276
Irf2bp2	interferon regulatory factor 2 binding protein 2	-1.52	0.0276
Snrpd2	small nuclear ribonucleoprotein D2	-2.95	0.0276
Olfr324	olfactory receptor 324	1.52	0.0278
Ppp1r9b	protein phosphatase 1, regulatory subunit 9B	-1.66	0.0279
Sod1	superoxide dismutase 1, soluble	-3.78	0.028
Tm4sf1	transmembrane 4 superfamily member 1	-1.72	0.0281
Cltb	clathrin, light polypeptide (Lcb)	-1.5	0.0282
H2-Ke6	H2-K region expressed gene 6	-1.61	0.0282
S100b	S100 protein, beta polypeptide, neural	-1.7	0.0282
Grina	glutamate receptor, ionotropic, N-methyl D-aspartate-associated protein 1 (glutamate binding)	-3.28	0.0283
Pfkm	phosphofructokinase, muscle	-1.6	0.0284
Slc35b1	solute carrier family 35, member B1	-1.6	0.0284
Fxyd6	FXYD domain-containing ion transport regulator 6	-1.77	0.0285
Rprml	reprimo-like	-1.66	0.0285
Dynlrb1	dynein light chain roadblock-type 1	-3.55	0.0286
Pik3c2a	phosphatidylinositol 3-kinase, C2 domain containing, alpha polypeptide	-1.56	0.0286

Mlc1	megalencephalic leukoencephalopathy with subcortical cysts 1 homolog (human)	-2.01	0.0287
Slc6a1	solute carrier family 6 (neurotransmitter transporter, GABA), member 1	-1.79	0.0287
Gnao1	guanine nucleotide binding protein, alpha O	-1.84	0.0288
Rab7	RAB7, member RAS oncogene family	-5.62	0.0289
Sobp	sine oculis-binding protein homolog (Drosophila)	-1.75	0.0291
Cend1	cell cycle exit and neuronal differentiation 1	-1.75	0.0292
Hey1	hairy/enhancer-of-split related with YRPW motif 1	-4.86	0.0292
Gm3727	predicted gene 3727	1.73	0.0295
Mtch1	mitochondrial carrier homolog 1 (C. elegans)	-2.01	0.0295
Ogdh	oxoglutarate (alpha-ketoglutarate) dehydrogenase (lipoamide)	-1.73	0.0298
Vti1b	vesicle transport through interaction with t-SNAREs 1B	-1.61	0.0298
Gm14308	predicted gene 14308 (Gm14308), mRNA.	-6.25	0.0301
Epas1	endothelial PAS domain protein 1	-4.83	0.0302
Olfr348	olfactory receptor 348	1.51	0.0302
Mrpl13	mitochondrial ribosomal protein L13	-1.54	0.0304
Prps1	phosphoribosyl pyrophosphate synthetase 1	-1.85	0.0304
Ptp4a2	protein tyrosine phosphatase 4a2	-2.1	0.0304
Tbc1d9	TBC1 domain family, member 9	-1.55	0.0306
Rora	RAR-related orphan receptor alpha	-2.9	0.0307
Gm11099	predicted gene 11099 [Source:MGI Symbol	1.83	0.0308
Mtmr6	myotubularin related protein 6	-1.6	0.0309
Tspyl4	TSPY-like 4	-2.16	0.0309
Dtymk	deoxythymidylate kinase	-1.58	0.0311
Olfr1055	olfactory receptor 1055	-2.03	0.0311
Vkorc1	vitamin K epoxide reductase complex, subunit 1	-7.56	0.0311
Ndufa4	NADH dehydrogenase (ubiquinone) 1 alpha subcomplex, 4	-5.06	0.0312
Unc50	unc-50 homolog (C. elegans)	-1.61	0.0312
Tnpo3	transportin 3	-1.53	0.0313
Acsl5	acyl-CoA synthetase long-chain family member 5	-1.53	0.0314
Serpini1	serine (or cysteine) peptidase inhibitor, clade I, member 1	-1.52	0.0314
Coprs	coordinator of PRMT5, differentiation stimulator	-1.62	0.0315
Zmat2	zinc finger, matrin type 2	-2.25	0.0316
Tmod2	tropomodulin 2	-1.78	0.0319
Erh	enhancer of rudimentary homolog (Drosophila)	-1.97	0.0322
Gpatch8	G patch domain containing 8	-2	0.0322
Mat2a	methionine adenosyltransferase II, alpha	-1.78	0.0323
Rpl9-ps6	ribosomal protein L9, pseudogene 6 [Source:MGI Symbol	-3.49	0.0324
Gm11808	predicted gene 11808 [Source:MGI Symbol	-3.15	0.0325
Eif4g2	eukaryotic translation initiation factor 4, gamma 2	-1.87	0.0329
Rpl29	ribosomal protein L29	-1.64	0.033

Pcbd2	pterin 4 alpha carbinolamine dehydratase/dimerization cofactor of hepatocyte nuclear factor 1 alpha (TCF1) 2	-1.69	0.0331
Ppp2r2a	protein phosphatase 2, regulatory subunit B, alpha	-1.7	0.0331
Glycam1	glycosylation dependent cell adhesion molecule 1	1.72	0.0333
Lgi1	leucine-rich repeat LGI family, member 1	-2.15	0.0333
Srp54a	signal recognition particle 54A	-1.83	0.0334
Cdkn2aipnl	CDKN2A interacting protein N-terminal like	-1.5	0.0335
Gm3298	predicted gene 3298 [Source:MGI Symbol	2.04	0.0335
Scd2	stearoyl-Coenzyme A desaturase 2	-2.54	0.0336
Arf3	ADP-ribosylation factor 3	-1.54	0.0338
Ccdc50	coiled-coil domain containing 50	-2.29	0.0339
Grpel1	GrpE-like 1, mitochondrial	-1.69	0.0339
Rassf3	Ras association (RalGDS/AF-6) domain family member 3	-1.72	0.0339
Cryab	crystallin, alpha B	-1.7	0.0341
Tmem176b	transmembrane protein 176B	-1.59	0.0341
Apod	apolipoprotein D	-4.97	0.0342
Hsp90ab1	heat shock protein 90 alpha (cytosolic), class B member 1	-1.67	0.0342
Mprip	myosin phosphatase Rho interacting protein	-1.56	0.0342
Fam107a	family with sequence similarity 107, member A	-4.67	0.0346
Gm2026	predicted gene 2026 [Source:MGI Symbol	-4.52	0.0346
Map7d2	MAP7 domain containing 2	-1.64	0.0346
Dusp19	dual specificity phosphatase 19	-1.53	0.0347
Napb	N-ethylmaleimide sensitive fusion protein attachment protein beta	-2.15	0.0347
Atp6v0e2	ATPase, H+ transporting, lysosomal V0 subunit E2	-1.96	0.0348
Agap2	ArfGAP with GTPase domain, ankyrin repeat and PH domain 2	-2.78	0.0351
Rpl3	ribosomal protein L3	-5.22	0.0351
Slc2a13	solute carrier family 2 (facilitated glucose transporter), member 13	-1.57	0.0351
Pfdn2	prefoldin 2	-1.56	0.0352
Gm20834	predicted gene, 20834	1.92	0.0353
Tmem38a	transmembrane protein 38A	-1.64	0.0353
BC005537	cDNA sequence BC005537	-1.82	0.0356
2610507B11Rik	RIKEN cDNA 2610507B11 gene	-1.85	0.0357
Mzt1	mitotic spindle organizing protein 1	-5.64	0.0357
Peak1	pseudopodium-enriched atypical kinase 1	-1.92	0.0357
Gba	glucosidase, beta, acid	-1.82	0.0358
Cfl1	cofilin 1, non-muscle	-1.72	0.0359
Vkorc1l1	vitamin K epoxide reductase complex, subunit 1-like 1	-1.58	0.0361
Calr	calreticulin	-1.57	0.0364
Copz1	coatomer protein complex, subunit zeta 1	-1.72	0.0369
Fabp9	fatty acid binding protein 9, testis	1.58	0.037
Rock1	Rho-associated coiled-coil containing protein kinase 1	-1.71	0.0375

Mfsd5	major facilitator superfamily domain containing 5	-1.56	0.0376
Gsk3a	glycogen synthase kinase 3 alpha	-1.83	0.0377
Rps19bp1	ribosomal protein S19 binding protein 1	-1.7	0.0378
Suclg1	succinate-CoA ligase, GDP-forming, alpha subunit	-1.53	0.0378
St3gal5	ST3 beta-galactoside alpha-2,3-sialyltransferase 5	-2.18	0.0381
Tmem50a	transmembrane protein 50A	-7.57	0.0381
Fam168a	family with sequence similarity 168, member A	-1.66	0.0383
Atp5g2	ATP synthase, H+ transporting, mitochondrial F0 complex, subunit C2 (subunit 9)	-1.67	0.0385
Cisd1	CDGSH iron sulfur domain 1	-1.76	0.0385
Eif4h	eukaryotic translation initiation factor 4H	-2.52	0.0385
Gm17428	predicted gene, 17428 [Source:MGI Symbol	3.19	0.0385
Tardbp	TAR DNA binding protein	-2.01	0.0385
Hprt	hypoxanthine guanine phosphoribosyl transferase	-2.74	0.0386
Rasgrp1	RAS guanyl releasing protein 1	-1.93	0.0386
Slc12a2	solute carrier family 12, member 2	-1.92	0.0386
Tagln3	transgelin 3	-1.82	0.0387
Cpt1a	carnitine palmitoyltransferase 1a, liver	-2.62	0.0389
Pcp4l1	Purkinje cell protein 4-like 1	-1.53	0.0389
Pten	phosphatase and tensin homolog	-1.99	0.0389
Ncam1	neural cell adhesion molecule 1	-1.63	0.039
Nfkbia	nuclear factor of kappa light polypeptide gene enhancer in B cells inhibitor, alpha	-1.86	0.0394
Gm10181	predicted gene 10181 [Source:MGI Symbol	9.68	0.0395
Dstn	destrin	-4.18	0.0396
Atp1a3	ATPase, Na+/K+ transporting, alpha 3 polypeptide (Atp1a3), mRNA.	-2.36	0.0397
Arl6ip1	ADP-ribosylation factor-like 6 interacting protein 1	-4.49	0.0399
Olfr849	olfactory receptor 849	1.54	0.0399
Rhoa	ras homolog gene family, member A	-1.81	0.04
Adgrf5	adhesion G protein-coupled receptor F5	-2.32	0.0401
Gm14412	predicted gene 14412 [Source:MGI Symbol	-4.19	0.0401
Ghitm	growth hormone inducible transmembrane protein	-5.63	0.0402
Ppm1a	protein phosphatase 1A, magnesium dependent, alpha isoform	-1.77	0.0402
Gm10778	predicted gene 10778 (Gm10778), mRNA.	1.54	0.0403
Gm14325	predicted gene 14325	-1.7	0.0403
Gm14326	predicted gene 14326	-3.83	0.0403
Cdk10	cyclin-dependent kinase 10	-3.17	0.0404
Gabbr2	gamma-aminobutyric acid (GABA) B receptor, 2	-1.9	0.0404
Psd3	pleckstrin and Sec7 domain containing 3	-1.59	0.0404
Gm20917	predicted gene, 20917	1.59	0.0405
Gm3893	predicted gene 3893	-1.51	0.0405

Tspan3	tetraspanin 3	-3.36	0.0407
1110004F10Rik	RIKEN cDNA 1110004F10 gene	-1.87	0.0408
Gm12666	predicted gene 12666	-2.16	0.0408
Tmbim4	transmembrane BAX inhibitor motif containing 4	-1.64	0.0408
Uqcrc2	ubiquinol cytochrome c reductase core protein 2	-2.16	0.0408
Itm2b	integral membrane protein 2B	-9.47	0.041
Calm3	calmodulin 3	-1.66	0.0411
Omg	oligodendrocyte myelin glycoprotein	-1.52	0.0412
Gm3239	predicted gene 3239 [Source:MGI Symbol	1.89	0.0413
Lyz2	lysozyme 2	-1.92	0.0413
Dync1h1	dynein cytoplasmic 1 heavy chain 1	-1.6	0.0414
Gm3317	predicted gene 3317 (Gm3317), mRNA.	1.78	0.0414
Tmem59	transmembrane protein 59	-1.6	0.0419
Ndufb11	NADH dehydrogenase (ubiquinone) 1 beta subcomplex, 11	-4.09	0.0424
Samd8	sterile alpha motif domain containing 8	-1.78	0.0424
Ndufb6	NADH dehydrogenase (ubiquinone) 1 beta subcomplex, 6	-1.55	0.0425
Epb41l1	erythrocyte membrane protein band 4.1 like 1	-1.63	0.0426
Rheb	Ras homolog enriched in brain	-1.89	0.0426
App	amyloid beta (A4) precursor protein	-1.68	0.0427
Sdhb	succinate dehydrogenase complex, subunit B, iron sulfur (Ip)	-1.91	0.0427
Aplp2	amyloid beta (A4) precursor-like protein 2	-1.61	0.043
Gtf3c6	general transcription factor IIIC, polypeptide 6, alpha	-3.85	0.043
Slco1c1	solute carrier organic anion transporter family, member 1c1	-1.75	0.0432
Nr4a2	nuclear receptor subfamily 4, group A, member 2	-1.61	0.0433
Gnb1	guanine nucleotide binding protein (G protein), beta 1	-1.58	0.0435
Anxa6	annexin A6	-1.51	0.0437
Pdhb	pyruvate dehydrogenase (lipoamide) beta	-1.77	0.0441
Sumo3	small ubiquitin-like modifier 3	-1.88	0.0441
Atp6v0a1	ATPase, H+ transporting, lysosomal V0 subunit A1	-1.57	0.0443
Dnttip2	deoxynucleotidyltransferase, terminal, interacting protein 2	-2.5	0.0444
Pdxk	pyridoxal (pyridoxine, vitamin B6) kinase	-1.8	0.0447
Pik3r3	phosphatidylinositol 3 kinase, regulatory subunit, polypeptide 3 (p55)	-1.83	0.0447
Bmi1	Bmi1 polycomb ring finger oncogene	-2.06	0.0448
Gja6	gap junction protein, alpha 6	1.71	0.0455
Ypel5	yippee-like 5 (Drosophila)	-1.72	0.0455
Fam171b	family with sequence similarity 171, member B	-2.25	0.046
Lman2	lectin, mannose-binding 2	-1.5	0.0461
Pabpc1	poly(A) binding protein, cytoplasmic 1	-1.77	0.0463
Rtcb	RNA 2,3-cyclic phosphate and 5-OH ligase	-4.35	0.0464
Sacm1l	SAC1 (suppressor of actin mutations 1, homolog)-like (S. cerevisiae)	-1.54	0.0466

Hsp90b1	heat shock protein 90, beta (Grp94), member 1	-1.65	0.0467
Ptprm	protein tyrosine phosphatase, receptor type, M	-1.64	0.0467
Pcdh7	protocadherin 7	-2.19	0.0468
Tmem14c	transmembrane protein 14C	-1.56	0.0468
Eif1	eukaryotic translation initiation factor 1	-1.64	0.0469
Rhox7b	reproductive homeobox 7B	1.55	0.047
Fxyd1	FXYD domain-containing ion transport regulator 1	-1.75	0.0472
Pcsk2	proprotein convertase subtilisin/kexin type 2	-1.54	0.0472
Pomp	proteasome maturation protein	-3.23	0.0472
Trappc4	trafficking protein particle complex 4	-1.63	0.0472
Carm1	coactivator-associated arginine methyltransferase 1	-1.51	0.0476
Htra1	HtrA serine peptidase 1	-1.61	0.0478
Ephx1	epoxide hydrolase 1, microsomal	-2.13	0.0479
Itpr1	inositol 1,4,5-trisphosphate receptor 1	-1.68	0.0481
Prpf4b	PRP4 pre-mRNA processing factor 4 homolog B (yeast)	-1.6	0.0481
Gm14305	predicted gene 14305	-6.82	0.0482
Pdpk1	3-phosphoinositide dependent protein kinase 1	-1.6	0.0482
Gm14409	predicted gene 14409 [Source:MGI Symbol	-1.72	0.0483
Aff4	AF4/FMR2 family, member 4	-2.01	0.0486
Rnaset2a	ribonuclease T2A	-4.14	0.0487
Bloc1s1	biogenesis of lysosomal organelles complex-1, subunit 1	-2.23	0.0489
Resp18	regulated endocrine-specific protein 18	-5.92	0.0489
Cmtm5	CKLF-like MARVEL transmembrane domain containing 5	-2.81	0.049
Slc25a5	solute carrier family 25 (mitochondrial carrier, adenine nucleotide translocator), member 5	-3.28	0.0491
Lum	lumican	-1.94	0.0494
Ndufa2	NADH dehydrogenase (ubiquinone) 1 alpha subcomplex, 2	-11.54	0.0496
Nme1	NME/NM23 nucleoside diphosphate kinase 1	-2.18	0.0496
Slc4a4	solute carrier family 4 (anion exchanger), member 4	-1.87	0.0498
Slc7a11	solute carrier family 7 (cationic amino acid transporter, y+ system), member 11	-3.94	0.0498

Supplementary Table 8A: HGD+Curc/HGD miRNAs. Non-coding microRNAs differentially expressed by HGD+Curc relative to HGD

Gene Symbol	Name	5p/3p strand	Fold Change	P-val
Mir7008	microRNA 7008	mmu-miR-7008-5p	1.59	0.0004
Mir384	microRNA 384	mmu-miR-384-3p	1.77	0.0035
Mir692-1	microRNA 692-1	mmu-miR-692	-7.25	0.004
Mir7058	microRNA 7058	mmu-miR-7058-5p	1.69	0.0055
Mir6236	microRNA 6236	mmu-miR-6236	-1.94	0.0059
Mir199a-1	microRNA 199a-1	mmu-miR-199a-3p	1.59	0.0063

Mir467c	microRNA 467c	mmu-miR-467c-5p/3p	-4.95	0.009
Mir6917	microRNA 6917	mmu-miR-6917-5p	3.01	0.0092
Mirlet7a-1	microRNA let7a-1	mmu-let-7a-5p	2.46	0.0092
Mir875	microRNA 875	mmu-miR-875-5p	1.55	0.0125
Mir7k	microRNA 7k	mmu-let-7k	1.65	0.0126
Mir133a-2	microRNA 133a-2	mmu-miR-133a-3p	1.63	0.0138
Mir6916	microRNA 6916	mmu-miR-6916-5p	3.21	0.0142
Mir3092	microRNA 3092	mmu-miR-3092-3p	3.75	0.0148
Mir7223	microRNA 7223	mmu-miR-7223-5p	1.56	0.0152
Mir7063	microRNA 7063	mmu-miR-7063-5p	2.35	0.0169
Mir692-3	microRNA 692-3	mmu-miR-692	-3.4	0.017
Mir1941	microRNA 1941	mmu-miR-1941-5p	2.11	0.018
Mir6394	microRNA 6394	mmu-miR-6394	1.75	0.0188
Mir98	microRNA 98	mmu-miR-98-5p	2.44	0.0194
Mir7094-2	microRNA 7094-2	mmu-miR-7094b-2-5p	2	0.0206
Mir665	microRNA 665	mmu-miR-665-3p	-1.65	0.0207
Mir7028	microRNA 7028	mmu-miR-7028-5p	1.74	0.0211
Mir142	microRNA 142	mmu-miR-142a-5p	2.1	0.0217
Mir692-2	microRNA 692-2	mmu-miR-692	-12.24	0.0217
Mir6993	microRNA 6993	mmu-miR-6993-5p	2.73	0.0229
Mir6396	microRNA 6396	mmu-miR-6396	1.57	0.0249
Mir7212	microRNA 7212	mmu-miR-7212-5p	2.53	0.0284
Mir181d	microRNA 181d	mmu-miR-181d-5p	1.56	0.029
Mir28c	microRNA 28c	mmu-miR-28c	1.73	0.0295
Mir7054	microRNA 7054	mmu-miR-7054-5p	1.72	0.0312
Mir1958	microRNA 1958	mmu-miR-1958	-4	0.032
Mir7081	microRNA 7081	mmu-miR-7081-3p	1.62	0.033
Mir378c	microRNA 378c	mmu-miR-378c	-16.41	0.0337
Mir193b	microRNA 193b	mmu-miR-193b-3p	1.75	0.034
Mir3098	microRNA 3098	mmu-miR-3098-5p	2.07	0.0361
Mir6915	microRNA 6915	mmu-miR-6915-5p	2.14	0.0372
Mir7224	microRNA 7224	mmu-miR-7224-3p	-5.97	0.038
Mir667	microRNA 667	mmu-miR-667-3p	-1.97	0.0416
Mir6976	microRNA 6976	mmu-miR-6976-5p	1.75	0.046
Mir6380	microRNA 6380	mmu-miR-6380	-1.6	0.0469
Mir7668	microRNA 7668	mmu-miR-7668-3p	1.53	0.0476

Supplementary Table 8B: HGD+Curc/HGD miRNAs Cytoscape. Non-coding microRNAs differentially expressed by HGD+Curc relative to HGD in Cytosacpe network map and number of target genes.

miRNA nodes	Fold change	p-value	Target gene #
mmu-let-7a-5p/mmu-let-7k/mmu-miR-98-5p	2.46; 1.65; 2.45	0.0092; 0.0126; 0.0194	179
mmu-miR-6394	1.75	0.0188	168
mmu-miR-181d-5p	1.56	0.029	163
mmu-miR-142a-5p	2.1	0.0217	128
mmu-miR-665-3p	-1.65	0.0207	80
mmu-miR-199a-3p	1.59	0.0063	61
mmu-miR-378c	-16.41	0.0337	52
mmu-miR-384-3p	1.77	0.0035	49
mmu-miR-193b-3p	1.75	0.034	44
mmu-miR-875-5p	1.55	0.0125	16

Supplementary Table 9: HGD+Curc/HGD lncRNAs. Long non-coding lncRNAs differentially expressed by HGD+Curc relative to HGD

Gene Symbol	Name	Fold Change	P-val
Gm11681	predicted gene 11681	1.57	0.0008
Gm13189	predicted gene 13189 [Source:MGI Symbol;Acc:MGI:3650860]; novel transcript	3.62	0.0008
Gm16121	predicted gene 16121 [Source:MGI Symbol;Acc:MGI:3802131]; novel transcript	1.6	0.0009
Gm20463	predicted gene 20463 [Source:MGI Symbol;Acc:MGI:5141928]; novel transcript, antisense to Rnf5	1.6	0.0009
1700040F17Rik	PREDICTED: RIKEN cDNA 1700040F17 gene (1700040F17Rik), miscRNA.	1.85	0.001
Gm17361	predicted gene, 17361	1.53	0.0039
Gm17522	predicted gene, 17522	1.53	0.0039
Gm26633	predicted gene, 26633 [Source:MGI Symbol;Acc:MGI:5477127]	1.56	0.0042
4930555M17Rik	RIKEN cDNA 4930555M17 gene [Source:MGI Symbol;Acc:MGI:1925289]; novel transcript, antisense locus	1.67	0.0063
Astx3	amplified spermatogenic transcripts X encoded 3	1.53	0.0065
Gm16084	predicted gene 16084 [Source:MGI Symbol;Acc:MGI:3801938]; putative novel transcript	1.53	0.0075
Gm20675	predicted gene 20675 [Source:MGI Symbol;Acc:MGI:5313122]; novel transcript	1.83	0.0089
Gm21809	predicted gene, 21809 [Source:MGI Symbol;Acc:MGI:5433973]	1.51	0.0089
Gm12753	predicted gene 12753 [Source:MGI Symbol;Acc:MGI:3652140]; novel transcript	1.52	0.0095

6030471H07Rik	RIKEN cDNA 6030471H07 gene [Source:MGI Symbol;Acc:MGI:1925488]; novel transcript	1.55	0.0096
Gm17267	predicted gene, 17267	1.57	0.0098
Cdr1	cerebellar degeneration related antigen 1	-8.53	0.0104
Gm26826	predicted gene, 26826 [Source:MGI Symbol;Acc:MGI:5477320]	2.16	0.0118
Gm15414	predicted gene 15414 [Source:MGI Symbol;Acc:MGI:3705307]; novel transcript	1.51	0.0128
Gm13822	predicted gene 13822 [Source:MGI Symbol;Acc:MGI:3650351]; novel transcript, antisense to Oasl1 and Oasl2	1.63	0.0165
4930583P06Rik	RIKEN cDNA 4930583P06 gene	1.71	0.0182
Gm12204	predicted gene 12204 [Source:MGI Symbol;Acc:MGI:3650262]; novel transcript	1.6	0.0185
C030005K15Rik	RIKEN cDNA C030005K15 gene	1.85	0.02
1700120E14Rik	RIKEN cDNA 1700120E14 gene	1.63	0.0205
Nctc1	non-coding transcript 1	1.51	0.0208
Gm15713	predicted gene 15713	1.74	0.0254
Gm6117	predicted gene 6117 [Source:MGI Symbol;Acc:MGI:3647032]; novel transcript	1.79	0.0257
Gm13175	predicted gene 13175 [Source:MGI Symbol;Acc:MGI:3651286]; novel transcript	1.63	0.0261
4930548K13Rik	RIKEN cDNA 4930548K13 gene	1.63	0.0263
G630093K05Rik	RIKEN cDNA G630093K05 gene; RIKEN cDNA G630093K05 gene (G630093K05Rik), non-coding RNA.	1.62	0.0273
Gm15418	predicted gene 15418 [Source:MGI Symbol;Acc:MGI:3705275]; putative novel transcript	1.51	0.0287
4930414N06Rik	RIKEN cDNA 4930414N06 gene; RIKEN cDNA 4930414N06 gene (4930414N06Rik), non-coding RNA.	1.57	0.0301
4930473O22Rik	RIKEN cDNA 4930473O22 gene; RIKEN cDNA 4930473O22 gene (4930473O22Rik), non-coding RNA.	1.58	0.0325
Gm13335	predicted gene 13335 [Source:MGI Symbol;Acc:MGI:3651482]; novel transcript	-1.97	0.0329
B930095G15Rik	RIKEN cDNA B930095G15 gene	-1.93	0.0334
A430010J10Rik	RIKEN cDNA A430010J10 gene [Source:MGI Symbol;Acc:MGI:2442501]	1.62	0.0363
4930448D08Rik	RIKEN cDNA 4930448D08 gene [Source:MGI Symbol;Acc:MGI:1921921]; novel transcript	1.99	0.0397
AA386476	expressed sequence AA386476 [Source:MGI Symbol;Acc:MGI:3034595]	1.5	0.0474
Gm11986	predicted gene 11986 [Source:MGI Symbol;Acc:MGI:3650604]; novel transccript	1.61	0.0474

Gm15961	predicted gene 15961 [Source:MGI Symbol;Acc:MGI:3802142]; novel transcript	-2.35	0.0484

Supplementary Table 10: HGD+Curc/HGD snoRNAs. Non-coding small nuclear/nucleolar differentially expressed by HGD+Curc relative to HGD

Gene Symbol	Name	Fold Change	P-val
Gm22087	predicted gene, 22087 [Source:MGI Symbol;Acc:MGI:5451864]	1.8	0.0302
Gm22154	predicted gene, 22154 [Source:MGI Symbol;Acc:MGI:5451931]	-2.22	0.0209
Gm22203	predicted gene, 22203 [Source:MGI Symbol;Acc:MGI:5451980]	1.56	0.001
Gm22220	predicted gene, 22220 [Source:MGI Symbol;Acc:MGI:5451997]	-1.58	0.0041
Gm22498	predicted gene, 22289 [Source:MGI Symbol;Acc:MGI:5452066]	1.57	0.0354
Gm22530	predicted gene, 22530 [Source:MGI Symbol;Acc:MGI:5452307]	1.6	0.0309
Gm22663	predicted gene, 22663 [Source:MGI Symbol;Acc:MGI:5452440]	2.01	0.0332
Gm22748	predicted gene, 22748 [Source:MGI Symbol;Acc:MGI:5452525]	-16.42	0.0097
Gm22809	predicted gene, 22809 [Source:MGI Symbol;Acc:MGI:5452586]	1.57	0.0125
Gm22868	predicted gene, 22868 [Source:MGI Symbol;Acc:MGI:5452645]	1.89	0.0034
Gm22947	predicted gene, 22947 [Source:MGI Symbol;Acc:MGI:5452724]	1.54	0.0052
Gm22965	predicted gene, 22965 [Source:MGI Symbol;Acc:MGI:5452742]	1.82	0.0257
Gm23119	predicted gene, 23119 [Source:MGI Symbol;Acc:MGI:5452896]	-4.67	0.0046
Gm23207	predicted gene, 23207 [Source:MGI Symbol;Acc:MGI:5452984]	1.68	0.0328
Gm23219	predicted gene, 23219 [Source:MGI Symbol;Acc:MGI:5452996]	1.82	0.0085
Gm23294	predicted gene, 23294 [Source:MGI Symbol;Acc:MGI:5453071]	1.61	0.0222
Gm23300	predicted gene, 23300 [Source:MGI Symbol;Acc:MGI:5453077]	-1.62	0.0122
Gm23308	predicted gene, 23308 [Source:MGI Symbol;Acc:MGI:5453085]	1.52	0.0343
Gm23513	predicted gene, 23513 [Source:MGI Symbol;Acc:MGI:5453290]	1.55	0.021
Gm23554	predicted gene, 23554 [Source:MGI Symbol;Acc:MGI:5453331]	1.66	0.0103
Gm23607	predicted gene, 23607 [Source:MGI Symbol;Acc:MGI:5453384]	1.53	0.0059
Gm23858	predicted gene, 23858 [Source:MGI Symbol;Acc:MGI:5453635]	2.22	0.0463
Gm24079	predicted gene, 24079 [Source:MGI Symbol;Acc:MGI:5453856]	1.69	0.004
Gm24229	predicted gene, 24229 [Source:MGI Symbol;Acc:MGI:5454006]	2.16	0.0494
Gm24255	predicted gene, 24255 [Source:MGI Symbol;Acc:MGI:5454032]	1.79	0.0119
Gm24256	predicted gene, 24256 [Source:MGI Symbol;Acc:MGI:5454033]	1.86	0.0488
Gm24336	predicted gene, 24336 [Source:MGI Symbol;Acc:MGI:5454113]	-2.36	0.0042
Gm24348	predicted gene, 24348 [Source:MGI Symbol;Acc:MGI:5454125]	1.54	0.015
Gm24357	predicted gene, 24357 [Source:MGI Symbol;Acc:MGI:5454134]	-7.08	0.0297
Gm24384	predicted gene, 24384 [Source:MGI Symbol;Acc:MGI:5454161]	1.68	0.0466
Gm24400	predicted gene, 24400 [Source:MGI Symbol;Acc:MGI:5454177]	-16.58	0.0022
Gm24519	predicted gene, 24519 [Source:MGI Symbol;Acc:MGI:5454296]	1.8	0.0235
Gm24624	predicted gene, 24624 [Source:MGI Symbol;Acc:MGI:5454401]	1.53	0.0481

Gm24626	predicted gene, 24626 [Source:MGI Symbol;Acc:MGI:5454403]	1.55	0.0258
Gm24665	predicted gene, 24665 [Source:MGI Symbol;Acc:MGI:5454442]	1.64	0.0154
Gm24730	predicted gene, 24730 [Source:MGI Symbol;Acc:MGI:5454507]	1.56	0.031
Gm24884	predicted gene, 24884 [Source:MGI Symbol;Acc:MGI:5454661]	1.56	0.0426
Gm25007	predicted gene, 25007 [Source:MGI Symbol;Acc:MGI:5454784]	-1.66	0.0467
Gm25121	predicted gene, 25121 [Source:MGI Symbol;Acc:MGI:5454898]	-1.55	0.0318
Gm25138	predicted gene, 25138 [Source:MGI Symbol;Acc:MGI:5454915]	-3.06	0.0167
Gm25224	predicted gene, 25224 [Source:MGI Symbol;Acc:MGI:5455001]	2.62	0.0051
Gm25226	predicted gene, 25226 [Source:MGI Symbol;Acc:MGI:5455003]	1.66	0.0077
Gm25253	predicted gene, 25253 [Source:MGI Symbol;Acc:MGI:5455030]	1.87	0.0166
Gm25257	predicted gene, 25257 [Source:MGI Symbol;Acc:MGI:5455034]	2.54	0.0336
Gm25402	predicted gene, 25402 [Source:MGI Symbol;Acc:MGI:5455179]	1.53	0.0443
Gm25407	predicted gene, 25407 [Source:MGI Symbol;Acc:MGI:5455184]	1.8	0.0194
Gm25418	predicted gene, 25418 [Source:MGI Symbol;Acc:MGI:5455195]	1.74	0.0014
Gm25539	predicted gene, 25539 [Source:MGI Symbol;Acc:MGI:5455316]	1.5	0.0082
Gm25560	predicted gene, 25560 [Source:MGI Symbol;Acc:MGI:5455337]	1.51	0.0168
Gm25610	predicted gene, 25610 [Source:MGI Symbol;Acc:MGI:5455387]	1.54	0.0273
Gm25630	predicted gene, 25630 [Source:MGI Symbol;Acc:MGI:5455407]	1.71	0.0122
Gm25798	predicted gene, 25798 [Source:MGI Symbol;Acc:MGI:5455575]	3.06	0.005
Gm25824	predicted gene, 25824 [Source:MGI Symbol;Acc:MGI:5455601]	1.51	0.0427
Gm26020	predicted gene, 26020 [Source:MGI Symbol;Acc:MGI:5455797]	1.53	0.0012
Gm26022	predicted gene, 26022 [Source:MGI Symbol;Acc:MGI:5455799]	2.27	0.0007
Gm26072	predicted gene, 26072 [Source:MGI Symbol;Acc:MGI:5455849]	1.69	0.0288
Gm26205	predicted gene, 26205 [Source:MGI Symbol;Acc:MGI:5455982]	-2.58	0.0344
Gm26244	predicted gene, 26244 [Source:MGI Symbol;Acc:MGI:5456021]	-1.54	0.0156
Gm26288	predicted gene, 26288 [Source:MGI Symbol;Acc:MGI:5456065]	1.65	0.0098
Gm26358	predicted gene, 26358 [Source:MGI Symbol;Acc:MGI:5456135]	2.09	0.0256
Gm28020	predicted gene, 28020 [Source:MGI Symbol;Acc:MGI:5531402]	2.08	0.0396
Snord16a	small nucleolar RNA, C/D box 16A	-4.07	0.0013
Snord59a	small nucleolar RNA, C/D box 59A [Source:MGI Symbol;Acc:MGI:5454983]	1.59	0.0103
Snord82	small nucleolar RNA, C/D box 82	4.72	0.0311

Supplementary Table 11: HGD+Curc Diseases CTD neuro. Associated human neurological diseases relative to HGD+Curc DEGs identified in the Comparative Toxicogenomics Database.

Disease Name	Disease Categories	Corrected p-value	Gene count
Nervous System Malformations	Congenital abnormality | Nervous system disease	1.1E-07	24
Malformations of Cortical Development	Congenital abnormality | Nervous system disease	0.000689	12
Heredodegenerative Disorders, Nervous System	Genetic disease (inborn) | Nervous system disease	8.33E-08	25
Mental Disorders	Mental disorder	1.17E-10	56
Neurodevelopmental Disorders	Mental disorder	5.02E-09	40
Intellectual Disability	Mental disorder | Nervous system disease | Signs and symptoms	8.29E-07	24
Mitochondrial Diseases	Metabolic disease	0.00127	11
Synucleinopathies	Metabolic disease | Nervous system disease	0.04358	8
Muscular Diseases	Musculoskeletal disease | Nervous system disease	0.01631	14
Nervous System Diseases	Nervous system disease	6.28E-24	100
Brain Diseases	Nervous system disease	4.69E-16	55
Central Nervous System Diseases	Nervous system disease	3.63E-15	57
Neurodegenerative Diseases	Nervous system disease	2.48E-09	33
Neuromuscular Diseases	Nervous system disease	5.5E-08	29
Epilepsy	Nervous system disease	0.000253	14
Movement Disorders	Nervous system disease	0.00045	15
Parkinsonian Disorders	Nervous system disease	0.00385	10
Peripheral Nervous System Diseases	Nervous system disease	0.00547	13
Basal Ganglia Diseases	Nervous system disease	0.00619	12
Polyneuropathies	Nervous system disease	0.00635	10
Epileptic Syndromes	Nervous system disease	0.01822	9
Cerebellar Diseases	Nervous system disease	0.02451	9
Parkinson Disease	Nervous system disease	0.02952	8
Neurologic Manifestations	Nervous system disease | Signs and symptoms	7.28E-11	49
Neurobehavioral Manifestations	Nervous system disease | Signs and symptoms	6.17E-06	26
Dyskinesias	Nervous system disease | Signs and symptoms	0.0000391	16

Ataxia	Nervous system disease | Signs and symptoms	0.000487	11
Cerebellar Ataxia	Nervous system disease | Signs and symptoms	0.00772	8

Supplementary Table 12: HGD+Curc/HGD vs HGD/LGD Heatmap. Fold changes of common genes across the two key comparisons.

Gene name	HGD / LGD fc	HGD+Curc / HGD fc	Gene name	HGD / LGD fc	HGD+Curc / HGD fc
4930466F19RIK	6.67	-2.11	Lonp2	6.29	-2.26
4930473O22Rik	3.83	1.58	Lrp1	7.08	-1.61
Abhd3	7.2	-2.27	Lynx1	4.55	-1.61
Actg-ps1	8.47	-2.72	Maf	5.86	-2.11
Ano1	5.96	-1.76	Mir1941	6.16	2.11
Ano6	5.49	-1.61	Mir692-1	7.63	-7.25
Apoa1bp	9.22	-3.64	Mir7081	6.3	1.62
Arhgdia	6.51	-1.67	Mir7212	8.03	2.53
Atf2	5.81	-2.1	Mirlet7a-1	5.59	2.46
Atl2	7.59	-2.63	Mlc1	6.46	-2.01
Atp1b2	6.24	-1.59	Mrpl27	5.53	-1.64
Atp2b4	7.35	-2.85	Msl2	6.51	-2.8
Atp6v1c1	6.6	-1.8	Mtch1	7.81	-2.01
AY036118	9.63	-3.66	Mtmr6	6.56	-1.6
Brms1l	6.26	-2.03	Myl9	6.83	-3.22
Camk2n1	6.66	-2.19	Mzt1	7.64	-5.64
Carm1	5.74	-1.51	Napb	6.64	-2.15
Cd248	5.61	-2.55	Ncam1	6.59	-1.63
Cisd1	5.39	-1.76	Ndufa13	9.98	-5.91
Clk3	7.04	-2.53	Ndufb6	8.16	-1.55
Cltb	6.63	-1.5	Ndufb7	6.79	-2.76
Copb2	6.59	-2.37	Nfe2l1	8.57	-1.64
Cox5a	8.18	-6.6	Nlk	6.69	-1.62
Cox5b	10.5	-3.69	Oat	5.23	-1.6
Cox6a1	9.87	-3.48	Olfr692	4.81	1.7
Cox7a2l	6.45	-2.77	Ost4	7.82	-2.7
Cpt1a	6.38	-2.62	Ostc	7.08	-7.03
Crbn	5.45	-2.09	Oxct1	6.52	-1.81
Ctsb	8.04	-2.91	Pabpc1	6.42	-1.77
Cul3	6.06	-1.58	Papd4	5.92	-2.87
Cuta	5.35	-1.53	Pcdh9	7.03	-2.55
Cyfip2	6.72	-2.11	Pcna	5.9	-2.22

D5Ertd683e	3.2	2.06	Pcna-ps2	5.77	-2.23
D8Ertd738e	8.03	-5.55	Pcyox1	7.04	-1.8
Dap3	5.53	-1.77	Pfkm	6.59	-1.6
Dctn2	5.23	-1.95	Pgam1-ps2	7.26	-1.53
Ddn	6.58	-2.22	Pik3c2a	5.62	-1.56
Dennd6a	5.21	-1.69	Plpp3	5.8	-2.45
Dnajc4	5.56	-2.38	Polr2j	6.27	-1.83
Dram2	5.67	-2.07	Ppa1	6.16	-2.61
Drg1	8.72	-6.08	Ppp1r14b	5.88	-2.39
Dtymk	5.2	-1.58	Prpf8	7.08	-3.01
Eef2	8.24	-2.42	Psma6	7.04	-2.2
Eif2b5	5.58	-1.51	Psmb5	8.96	-4.28
Eif5al3-ps	6.45	-3.66	Rab24	6.95	-3.34
Ephx1	7.39	-2.13	Rabac1	8.18	-4.46
Epm2aip1	6.99	-1.69	Raph1	5.58	-1.96
Etnk1	8.15	-6.39	Rassf3	5.92	-1.72
Fads3	5.62	-1.76	Rbm4	5	-1.72
Fam168a	5.34	-1.66	Rbx1	7.68	-4.2
Fam205a1	5.2	-1.57	Resp18	7.86	-5.92
Fam73a	5.6	-1.71	Rheb	6	-1.89
Gabbr2	7.49	-1.9	Rpl10-ps1	11.12	-8.9
Gkn3	6.24	-2.44	Rpl10-ps6	10.4	-5.28
Gm10171	8.83	-1.83	Rpl14-ps1	11.06	-3.9
Gm10592	6.67	-2.11	Rpl18	5.51	-1.62
Gm11273	9.23	-4.04	Rpl18-ps2	9.99	-7.93
Gm11336	5.41	-2.02	Rpl19-ps12	8.14	-2.05
Gm11478	14.39	-16.11	Rplp0	7.35	-2.33
Gm11539	7.35	-3.14	Rplp1-ps1	7.21	-1.71
Gm11628	9.67	-2.92	Rps2-ps13	9.37	-5.21
Gm11633	6.55	-1.64	Rps4x	9.08	-4.37
Gm11675	7.12	-2.3	Rpsa-ps4	9.59	-2.88
Gm11688	8.9	-2.3	Rtn4	6.59	-1.58
Gm11956	5.42	-1.85	Scarb2	6.2	-1.87
Gm12074	5.45	-1.96	Scp2-ps2	6.39	-4.17
Gm12226	5.5	-2.31	Sdhb	5.31	-1.91
Gm12251	8.4	-8.48	Sepw1	8.3	-3.08
Gm12254	8.98	-2.77	Sf3a3	5.6	-1.86
Gm12726	5.91	-2.59	Sgpp2	6.43	-2.75
Gm12816	6.71	-2.76	Slc12a2	5.62	-1.92
Gm12892	5.38	-1.67	Slc25a18	5.2	-1.52
Gm13171	5.38	-1.64	Slc2a13	5.13	-1.57
Gm13249	7.63	-2.03	Slc38a3	6.06	-1.85

Gm13298	5.7	-2.21	Smdt1	8.34	-8.99
Gm13413	7.27	-2.91	Smim13	4.94	-1.64
Gm13573	7.37	-2.33	Snord16a	5.79	-4.07
Gm13680	10.3	-15.02	Snord82	5.25	4.72
Gm13797	8.09	-12.61	Stk39	7.72	-2.72
Gm14150	8	-4.69	Taldo1	5.3	-1.8
Gm14165	7.61	-2.8	Tbc1d9	5.03	-1.55
Gm14292	9.35	-13.89	TC0100000104.mm.1	6.23	-2.95
Gm14450	5.68	-2.39	TC0100000178.mm.1	5.45	-2.1
Gm15028	3.27	1.73	TC0100000555.mm.1	8.31	-16.33
Gm15487	11.31	-4.61	TC0100001808.mm.1	6.23	-5.67
Gm15590	6.21	-3.61	TC0100002672.mm.1	4.5	-1.68
Gm15724	6.63	1.87	TC0100003086.mm.1	7.76	-5.02
Gm16209	7.87	-2.26	TC0200001416.mm.2	11.21	3.09
Gm16418	9.35	-3.31	TC0200001598.mm.2	4.12	1.6
Gm16580	9.69	-9.24	TC0200002802.mm.1	7.68	-4.45
Gm17047	3.71	2.07	TC0200004093.mm.1	8.41	-8.8
Gm17069	3.78	1.6	TC0400000440.mm.2	3.68	2.06
Gm17221	3.67	1.56	TC0400001061.mm.1	6.02	-2.61
Gm17257	5.56	-2.41	TC0400001114.mm.1	8.29	1.58
Gm17428	17.1	3.19	TC0400001488.mm.1	7.79	1.5
Gm17541	12.28	-3.86	TC0400003138.mm.1	4.4	-1.66
Gm1866	6.23	-3.3	TC0400003261.mm.1	5.62	-3.21
Gm19494	8.18	-10.64	TC0400003474.mm.1	10.33	1.56
Gm19496	8.47	-15.39	TC0400003735.mm.2	4.21	1.84
Gm19738	8.14	-3.09	TC0500000141.mm.1	4.84	-1.54
Gm19774	10	-30.91	TC0500001040.mm.1	6.87	-4.47
Gm19886	6.97	-4.59	TC0500001812.mm.1	5.78	-2.21
Gm19933	6.98	-4.96	TC0500002367.mm.1	6.97	-3.55
Gm19974	7.07	-2.41	TC0500003017.mm.1	5.78	-2.43
Gm20349	6.19	-3.44	TC0500003033.mm.2	16.53	1.9
Gm20391	4.43	1.71	TC0500003042.mm.2	13.37	1.54
Gm20675	5.06	1.83	TC0500003353.mm.1	4.73	-2.49
Gm21968	6.67	-2.11	TC0600000156.mm.1	3.59	1.67
Gm22087	4.64	1.8	TC0600000497.mm.2	3.44	1.69
Gm22202	4.07	1.54	TC0600002621.mm.1	5.01	-1.84
Gm22289	10.42	-18.92	TC0600002682.mm.1	6.05	-2.61
Gm22663	9.42	2.01	TC0600003386.mm.1	9.93	1.81
Gm22730	4.89	3.43	TC0700001911.mm.1	6.63	-1.58
Gm22965	6.96	1.82	TC0700004096.mm.1	6.12	-3.84
Gm23119	6.67	-4.67	TC0800001046.mm.1	6.37	-2.22
Gm23662	4.24	1.67	TC0800001957.mm.1	7.42	1.81

Gm24255	6.72	1.79	TC0800001971.mm.1	4.67	2.03
Gm24348	3.43	1.54	TC0800001985.mm.1	3.57	2.05
Gm24357	8.21	-7.08	TC0800002360.mm.1	4.78	-1.7
Gm24400	8.89	-16.58	TC0900001581.mm.1	5.43	-3.09
Gm24519	3.75	1.8	TC0900001929.mm.1	6.49	-2.87
Gm24628	3.44	1.56	TC0900002148.mm.1	7.27	-4.43
Gm25407	3.89	1.8	TC0X00001537.mm.1	5.48	3.29
Gm25418	5.66	1.74	TC0X00003293.mm.1	5.09	-1.53
Gm25630	4.13	1.71	TC1000000237.mm.1	8.29	3.77
Gm25732	6.91	-6.2	TC1000000965.mm.1	5.92	2.33
Gm25911	18.14	-16	TC1000000985.mm.2	14.47	8.53
Gm25992	4.54	-2.58	TC1000001386.mm.1	9.73	1.92
Gm26308	7.71	2.22	TC1000001757.mm.1	4.63	-1.78
Gm26455	4.26	1.69	TC1000001978.mm.1	5.1	1.67
Gm26557	3.81	1.82	TC1000002972.mm.1	9.73	1.92
Gm26701	5.83	-3.02	TC1100000194.mm.1	6.35	2.05
Gm26826	3.51	2.16	TC1100001364.mm.1	6.42	-2.01
Gm26951	4.44	-1.93	TC1100001421.mm.1	5.8	-3.9
Gm27626	15.43	-29.72	TC1100001865.mm.1	4.58	1.8
Gm3208	4.24	1.64	TC1100004012.mm.1	5.67	-2.31
Gm3531	9.02	-3.1	TC1100004086.mm.1	7.92	-2.92
Gm3934	7.03	-2.38	TC1200000713.mm.1	4.55	-2.09
Gm4017	8.34	-5.69	TC1200001267.mm.2	12.43	1.83
Gm4735	9.33	-6.03	TC1200001368.mm.1	6.29	-3.53
Gm4953	6.4	-2.46	TC1200001378.mm.1	4.02	2.15
Gm5139	7.65	-1.67	TC1200002202.mm.1	5.1	-1.87
Gm5239	9.07	-4.63	TC1300000419.mm.1	7.64	-1.68
Gm5265	7.67	-4.3	TC1300001308.mm.1	5.9	-2.23
Gm5943	5.28	-1.79	TC1300001785.mm.1	6.2	-4.63
Gm6055	7.59	-1.78	TC1300001873.mm.1	3.64	1.52
Gm6117	3.83	1.79	TC1300002570.mm.1	4.7	-1.72
Gm6444	7.44	-3.67	TC1400001678.mm.1	5.74	-3.65
Gm6969	8.11	-3.64	TC1400002541.mm.1	6.02	-2.57
Gm7332	6.61	-2.09	TC1500000243.mm.1	4.92	1.7
Gm7381	8.62	-5.73	TC1500000708.mm.1	5.14	-2.43
Gm7497	7.88	-2.99	TC1600001123.mm.1	7.71	-5.06
Gm8420	5.68	-3	TC1600001480.mm.1	8.91	-3.91
Gm8730	7.53	-5.18	TC1700000441.mm.1	9.93	2.14
Gm9103	5.25	-1.73	TC1700000547.mm.1	4.04	2.38
Gm9294	6.27	-2.39	TC1700001338.mm.1	6.3	-3.37
Gm9625	7.6	-3.85	TC1700001387.mm.1	7.54	-5.88
Gm9840	7.45	-5.41	TC1700001452.mm.1	4.37	2.17

Grina	8.9	-3.28	TC1700001629.mm.1	4	1.65
Gstp1	7.2	-2.14	TC1700002099.mm.1	7.95	-2.98
Gstp2	6.96	-1.84	TC1700002364.mm.1	5.45	-2.1
Higd2a	9	-8.94	TC1800000844.mm.1	7.03	-5
Hist1h1c	5.79	-2.88	TC1900000003.mm.1	7.81	-3.68
Hist1h2af	7.54	-4.28	TC1900000146.mm.1	8.19	-2.2
Hist1h2aj	7.76	-7.28	TC1900001403.mm.2	3.33	1.85
Hist1h2al	7.32	-3.45	Tgfb3	6.18	-2.28
Hist1h2an	8.11	-5.89	Tmem14c	4.76	-1.56
Id1	5.88	-1.88	Tmem176b	5.5	-1.59
Ifi27	6.57	-2.14	Tmem30a	7.23	-3.11
Ighv10-1	8.09	2.16	Trappc11	6.08	-1.76
Ighv5-12-4	6.6	2.07	Triqk	5.13	-2.01
Ik	7.78	-3.38	Tsn	5.98	-2
Jag1	5.92	-2.15	Tspyl4	6.18	-2.16
Lancl1	6.32	-2.18	Ttc9	5.42	-1.79
Lin7a	6.06	-2.77	Ttc9b	5.74	-2.24
LOC100861642	10.27	-13.46	Tuba1b	7.7	-2.37
LOC100861649	4.82	-1.82	Tubg1	5.8	-1.88
LOC100861650	7.98	-2.83	Ubc	12.06	-4.86
LOC100861832	5.58	-2.42	Ube2nl	5.55	-2.65
LOC100861833	7.57	-3.59	Uchl1	6.46	-4.06
LOC100861862	6.26	-3.29	Uck2	7.35	-3.86
LOC100862094	8.3	-4.14	Uck2	6.13	-2.81
LOC100862121	5.48	-1.55	Ufd1l	5.68	-1.62
LOC100862145	7.71	-2.26	Uqcr11	6.32	-2.64
LOC100862246	6.94	-4.67	Uqcrc1	6.06	-2.15
LOC100862384	5.83	-1.95	Vamp1	7.47	-2.73
LOC100862392	5.97	-2.22	Vkorc1	7.62	-7.56
LOC432823	12.11	-4.47	Vps28	7.88	-3.19
			Wdr74	5.9	-2.17

Supplementary Table 13: Diet Pellet Composition of Treatment Groups

Component (g/kg)	LGD (Teklad Custom Diets TD.08485)	HGD (Teklad Custom Diets TD.05230)	HGD+ Curcumin
**Casein**	195	195	195
**DL-Methionine**	3	3	3
**Sucrose**	120	341	341

**Corn Starch**	432.99	211.99	209.99
**Maltodextrin**	100	100	100
**Anhydrous Milk-fat**	37.2	37.2	37.2
**Soybean Oil**	12.8	12.8	12.8
**Cellulose**	50	50	50
**Mineral Mix (AIN-76 170915)**	35	35	35
**Calcium Carbonate**	4	4	4
**Vitamin Mix (Teklad 40060)**	10	10	10
**Ethoxyquin (antioxidant)**	0.01	0.01	0.01
**Curcumin**	N/A	N/A	2
